# Global-scale GWAS associates a subset of SNPs with animal-adapted variants in *M. tuberculosis* complex

**DOI:** 10.1186/s12920-023-01695-5

**Published:** 2023-10-24

**Authors:** Evan P. Brenner, Srinand Sreevatsan

**Affiliations:** Department of Pathobiology and Diagnostic Investigation, College of Veterinary Medicine, Michigan State University, 784 Wilson Road, East Lansing, MI 48824 USA

## Abstract

**Background:**

While *Mycobacterium tuberculosis* complex (MTBC) variants are clonal, variant *tuberculosis* is a human-adapted pathogen, and variant *bovis* infects many hosts. Despite nucleotide identity between MTBC variants exceeding 99.95%, it remains unclear what drives these differences. Markers of adaptation into variants were sought by bacterial genome-wide association study of single nucleotide polymorphisms extracted from 6,362 MTBC members from varied hosts and countries.

**Results:**

The search identified 120 genetic loci associated with MTBC variant classification and certain hosts. In many cases, these changes are uniformly fixed in certain variants while absent in others in this dataset, providing good discriminatory power in distinguishing variants by polymorphisms. Multiple changes were seen in genes for cholesterol and fatty acid metabolism, pathways previously proposed to be important for host adaptation, including Mce4F (part of the fundamental cholesterol intake Mce4 pathway), 4 FadD and FadE genes (playing roles in cholesterol and fatty acid utilization), and other targets like Rv3548c and PTPB, genes shown essential for growth on cholesterol by transposon studies.

**Conclusions:**

These findings provide a robust set of genetic loci associated with the split of variant *bovis* and variant *tuberculosis*, and suggest that adaptation to new hosts could involve adjustments in uptake and catabolism of cholesterol and fatty acids, like the proposed specialization to different populations in MTB lineages by alterations to host lipid composition. Future studies are required to elucidate how the associations between cholesterol profiles and pathogen utilization differences between hosts and MTBC variants, as well as the investigation of uncharacterized genes discovered in this study. This information will likely provide an understanding on the diversification of MBO away from humans and specialization towards a broad host range.

**Supplementary Information:**

The online version contains supplementary material available at 10.1186/s12920-023-01695-5.

## Background

The *Mycobacterium tuberculosis* complex (MTBC) has afflicted human and animal health since the dawn of civilization. This ancient pathogen, typified by *M. tuberculosis* variant *tuberculosis* (MTB), infects humans primarily and is considered specialized for this niche [1, 2]. Its subversion of host immune responses, dormancy in granulomas for years or decades, and transmissibility suggest fine adaptation to humans, potentially to per-lineage adaptation to different human populations [3, 4]. MTB infects non-human hosts, including primates, and other animals (such as cattle) more infrequently [5, 6]. On the other hand, *M. tuberculosis* variant *bovis* (MBO) is a generalist pathogen – its host range includes foxes, seals, cattle, cervids, lions, dogs, mustelids, badgers, and others [1, 7–10]. The eponymous bovine reservoir is one of several for MBO [10], including white-tailed deer, elk, or bison in the US and Canada [9, 11, 12], red deer and wild boar populations in Spain [7], European badgers in the UK and Ireland [13, 14], and possums in New Zealand [14]. Despite host range differences, MTBC variants show a rigid population structure [15]. From the initial whole genome sequencing (WGS), researchers were surprised to find MTB and MBO shared 99.95% nucleotide identity, excluding genomic deletions in MBO [16]. Within a few years of the first MBO genome being sequenced, research began on what might drive variant differences, including gene expression (17), omics analysis [8], and metabolism [18, 19], among others. Meaningful variations have been reported, but it remains uncertain how MBO has evolved towards a generalist lifestyle away from a presumed MTB-like specialist ancestor.

To help address this gap in knowledge, WGS datasets were collected for MTBC variants from diverse hosts and countries. Paired-end read SRA datasets with metadata including country and host of isolation, and MTBC variant (n = 6,360 taxa, plus reference and outgroup) were used to create a set of 9,755 SNPs for a bacterial genome-wide association study (bGWAS). This sought to detect loci associated with classification as MTB or MBO, as well as any detectable host-specific markers (e.g., SNPs associated with isolation from cervids). Using RAxML-NG [20], prewas [21], and TreeWAS [22], bGWAS was performed with isolates classified by MBO (1) or not (0).

## Results

**Fig. 1:**
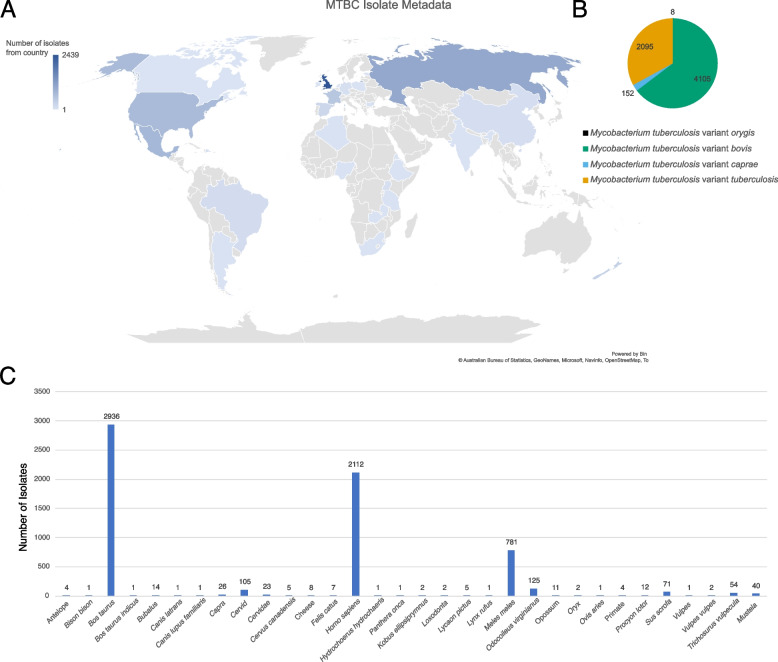
MTBC isolate collection metadata. **A** Geographical distribution of isolates worldwide, where darker colors represent more isolates from that country. **B** MTBC variant makeup of the collection. Values are based on NCBI/ENA SRA designations. **C** Host origin for the collection. *Bos taurus* is the dominant host type, followed by *Homo sapiens* and *Meles meles*

Core SNP extraction was successful for 6,362 isolates. Isolates were from 27 countries (Fig. 1A); included 2,096 MTB (including reference), 4,105 MBO, 152 variant *caprae*, and 8 variant *orygis* (Fig. 1B); and across 30 hosts (Fig. 1C). We additionally added *M. canettii* as the MTBC outgroup (*n* = 1). All sequence data used in this project are publicly available through NCBI and ENA. Accession IDs for all data are recorded in the table Additional file 1, with BioProjects in Column A, and corresponding SRA identifiers in Column B per sequence. The masked core SNP alignment was used for phylogenetic tree generation by RAxML-NG (Additional file 2), which shows splits based on MTBC variant, but is only used for GWAS and not intended for visualization due to its scale. The unmasked core SNP alignment is also provided for reference (Additional file 3). After prewas and maximum likelihood-based ancestral reconstruction, a final set of 7,524 variants over the 6,362 taxa was used for treeWAS input.

**Fig. 2:**
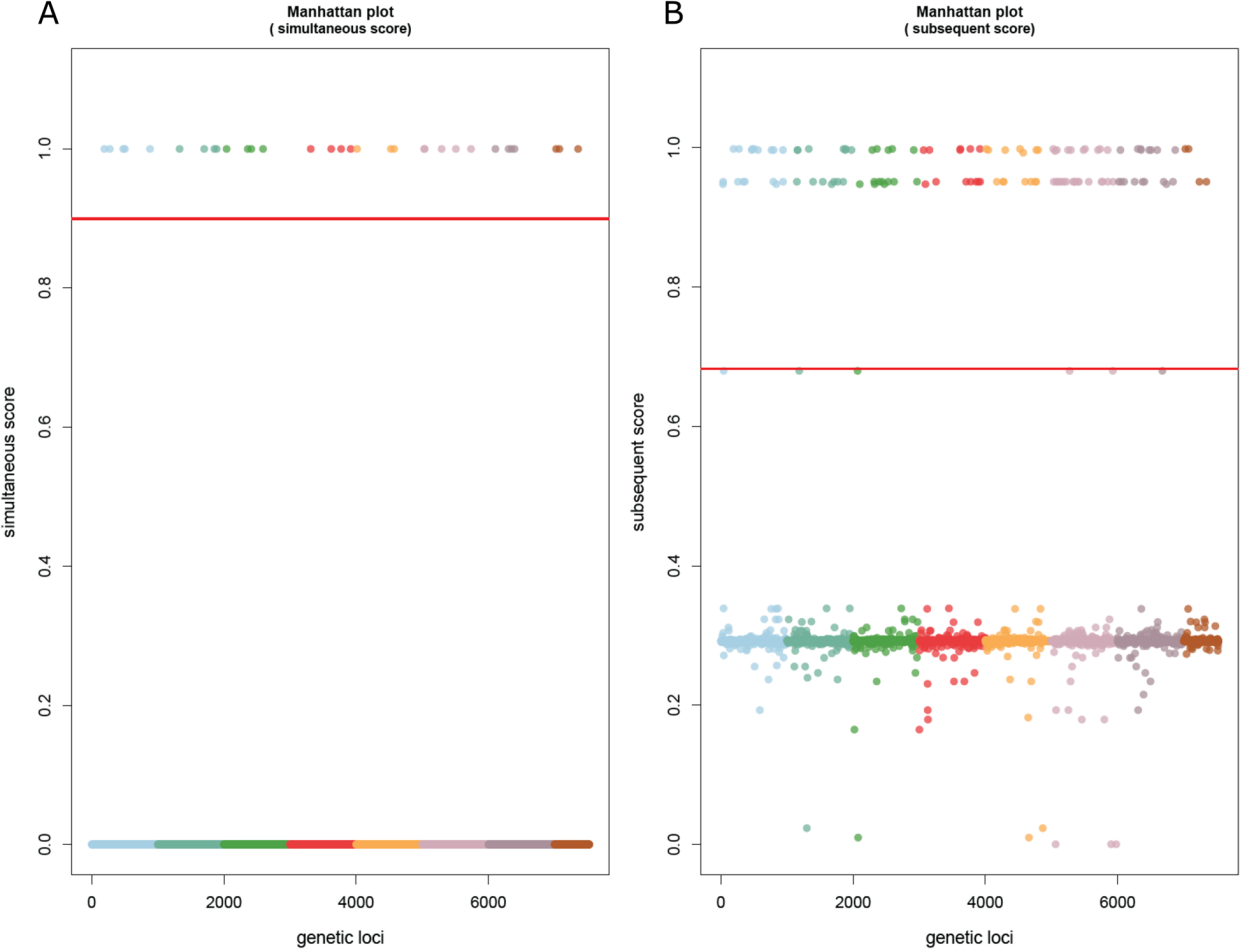
Manhattan plots of bGWAS results for phenotype “*M. tuberculosis* variant *bovis.*” **A** Simultaneous test of association, showing 32 loci ranked to be significant, of which 10 are of dubious quality. **B** Subsequent test of association, showing 120 loci are ranked to be significant. Details for each locus are available in Tables 1 (simultaneous) and 2 (subsequent)

**Fig. 3:**
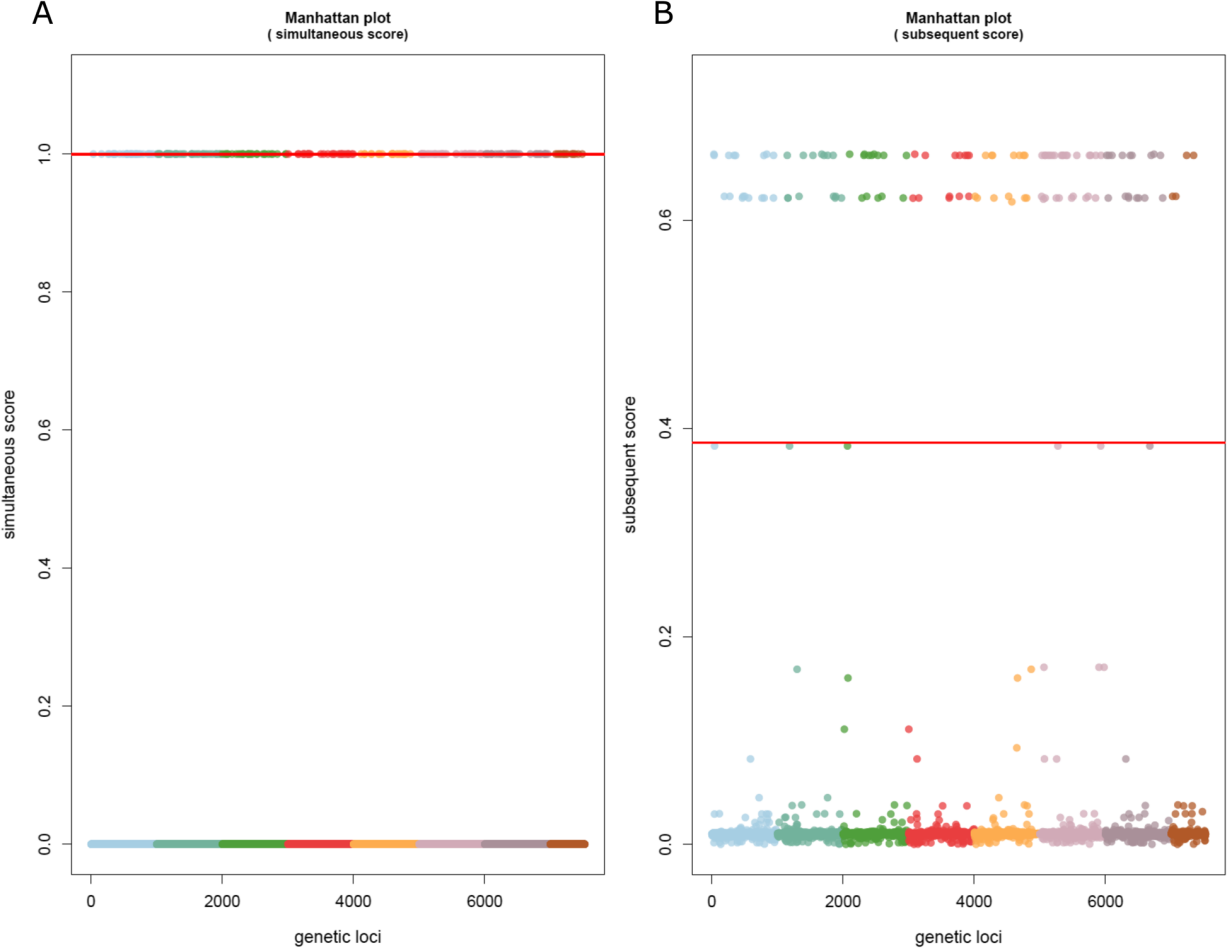
Manhattan plots of bGWAS results for phenotype “*Bovidae*” host. **A** Simultaneous test of association, showing no significantly ranked loci. **B** Subsequent test of association, showing 120 loci are ranked to be significant. The 120 loci identified here are identical to those seen in Table 2 and Fig. 2

**Fig. 4:**
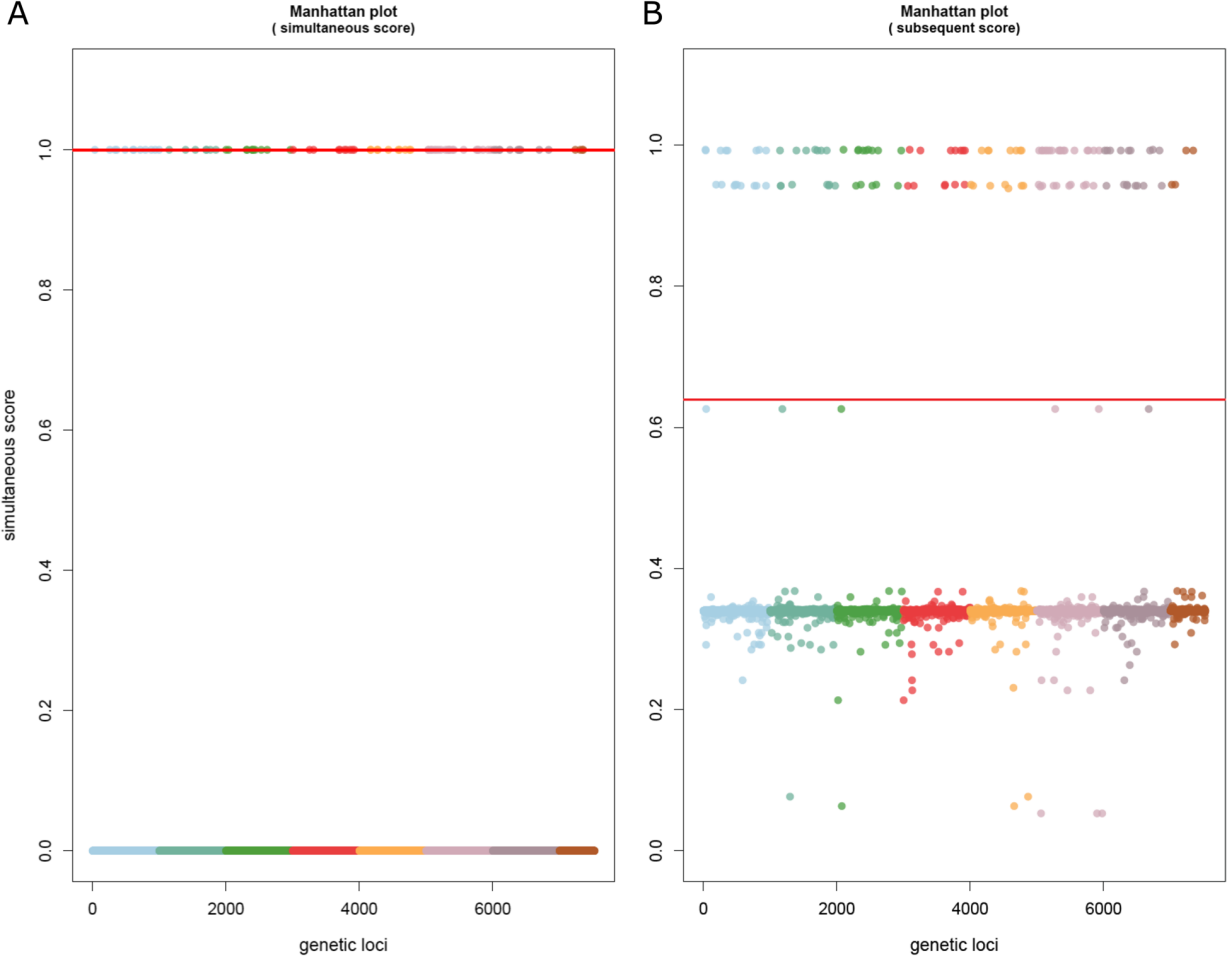
Manhattan plots of bGWAS results for phenotype “*Homo sapiens*” host. **A** Simultaneous test of association, showing no significantly ranked loci. **B** Subsequent test of association, showing 120 loci are ranked to be significant. The 120 loci identified here are identical to those seen in Table 2 and Figs. 2 and 3

TreeWAS runs three tests for statistical significance – the terminal, simultaneous, and subsequent tests. The terminal test identifies broad associations between genotype and phenotype looking only at terminal nodes in the tree; the simultaneous test more stringently identifies deterministic relationships of genotype and phenotype, without necessitating the relationship occur at all branches; the subsequent test utilizes the terminal test but adds ancestral state reconstruction to analyze all nodes of the tree [22]. A thorough explanation of these tests is provided by Dr. Collins on the treeWAS GitHub page [23]. The simultaneous and subsequent tests were used initially, as an ancestral reconstruction was available. When analyzing by phenotype of MBO (1) and Not MBO (0), treeWAS produced significant loci for both simultaneous and subsequent statistical scoring metrics (Fig. 2). Analysis by phenotype of *Bovidae* also produced the same 120 significant loci by the same subsequent test but did not produce loci for the simultaneous test (Fig. 3). Another search by phenotype of *Homo sapiens* produced the same 120 loci again by subsequent test and no loci by simultaneous test (Fig. 4). A phenotype of “non-standard hosts” (where *Homo sapiens* is the standard host for MTB, *Bovidae* for MBO, *Capra* for MCP, and *Oryx* for MOR) yielded no significant hits (Additional file 4). Analysis by phenotype *Cervidae* returned no significance (Additional file 5), but phenotype *Meles meles* (European badger) showed an unusual pattern by subsequent statistical test where nearly all SNPs clustered just around the significance cutoff, yielding hundreds of loci technically exceeding the cutoff yet are tightly clustered with those under it (Additional file 6A). The simultaneous score did not similar hits (Additional file 6B). A similar pattern was seen for *Sus* (Additional file 7). To investigate if these associations reflected geographic effects for *Meles meles,* as all badger samples were from the UK, GWAS analysis was performed by phenotype of UK origin, which yielded no significance by the subsequent test, and a single locus by simultaneous test (Additional file 8). This hit, for a variant present in only two isolates, is a spurious result. For *Meles meles* and *Sus,* it would appear the dataset composition makes it difficult to detect significance against a background already tightly associated with specific genotypes. MCP produced no significant hits (Additional file 9), and MOR was not attempted due to low representation on *M. orygis* samples in the dataset (*n* = 8).

The 32 loci identified by the simultaneous test for MBO are listed in Table 1.

**Table 1:** SNPs significantly associated with classification as MBO by Simultaneous statistical test

SNP	*p*-value	score	G1P1	G0P0	G1P0	G0P1	Locus	Protein	Description	Essentiality Notes from Mycobrowser
147,873	0	1	4100	2250	9	3			Intergenic, upstream of elongation factor G FusA2 (Rv0120c)	n/a
184,727	0	1	4100	2250	9	3	Rv0156	PntAb	Probable NAD(P) transhydrogenase (subunit alpha) PntAb [second part; integral membrane protein] (pyridine nucleotide transhydrogenase subunit alpha) (nicotinamide nucleotide transhydrogenase subunit alpha)	n/a
268,277	0	1	4100	2250	9	3	Rv0224c		Possible methyltransferase (methylase)	In vitro essential per multiple studies (Minato 2019; DeJesus 2017; Sassetti 2003; Griffin 2011)
277,862	0	1	4100	2250	9	3			Intergenic, downstream of FadE4 (Rv0231) and upstream of probable transcriptional regulatory protein (probably TetR/AcrR-family) (Rv0232)	n/a
**438,069**	**0**	**1**	**1**	**2260**	**0**	**4100**	**Rv0359**		**Probable conserved integral membrane protein**	**n/a**
1,234,657	0	1	4100	2250	9	3	Rv1108c	XseA	Probable exodeoxyribonuclease VII (large subunit) XseA (exonuclease VII large subunit)	n/a
**1,390,284**	**0**	**-1**	**0**	**2260**	**2**	**4100**	**Rv1248c**		**Multifunctional alpha-ketoglutarate metabolic enzyme**	**In vitro essential per multiple studies (Minato 2019; Carvalho 2010; Sassetti 2003; Griffin 2011)**
1,478,312	0	1	4100	2250	9	3	Rv1317c	AlkA	Probable bifunctional regulatory protein and DNA repair enzyme AlkA (regulatory protein of adaptative response) (methylphosphotriester-DNA–protein-cysteine S-methyltransferase)	n/a
1,499,291	0	1	4100	2250	9	4	Rv1330c	PncB1	Nicotinic acid phosphoribosyltransferase PncB1	n/a
**1,586,961**	**0**	**1**	**1**	**2260**	**0**	**4100**	**Rv1410c**		**Aminoglycosides/tetracycline-transport integral membrane protein**	**Essential in murine macrophages (Rengarajan 2005) and murine spleen (Sassetti and Rubin 2003)**
1,739,294	0	1	4100	2250	9	3	Rv1536	IleS	Isoleucyl-tRNA synthetase IleS	In vitro essential per multiple studies (Minato 2019; DeJesus 2017; Lamichhane 2003; Griffin 2011)
**1,763,524**	**0**	**1**	**1**	**2260**	**0**	**4100**	**Rv1559**	**IlvA**	**Probable threonine dehydratase IlvA**	**In vitro essential (DeJesus 2017; Griffin 2011), non-essential in rich media (Minato 2019)**
1,830,295	0	1	4100	2250	9	3	Rv1628c		Conserved protein	n/a
**2,314,425**	**0**	**-1**	**0**	**2260**	**2**	**4100**	**Rv2056c**	**RpsN2**	**30S ribosomal protein S14 RpsN2**	**Disruption provides growth advantage (DeJesus 2017)**
2,475,888	0	1	4100	2250	9	3	Rv2210c	IlvE	Branched-chain amino acid transaminase IlvE	In vitro essential (DeJesus 2017; Sassetti 2003; Griffin 2011), non-essential in rich media (Minato 2019)
2,528,773	0	1	4100	2250	9	3	Rv2254c		Probable integral membrane protein	n/a
2,658,676	0	1	4100	2250	9	3	Rv2379c	MbtF	Peptide synthetase MbtF (peptide synthase)	n/a
2,682,593	0	1	4100	2250	9	3	Rv2388c	HemN	Probable oxygen-independent coproporphyrinogen III oxidase HemN (coproporphyrinogenase) (coprogen oxidase)	Essential in murine spleen (Sassetti and Rubin, 2003)
2,912,516	0	1	4100	2250	9	3	Rv2585c		Possible conserved lipoprotein	n/a
2,927,291	0	1	4080	2250	9	21	Rv2598		Conserved hypothetical protein	n/a
3,140,153	0	1	4100	2250	9	4	Rv2833c	UgpB	Probable Sn-glycerol-3-phosphate-binding lipoprotein UgpB	Disruption provides growth advantage (DeJesus 2017)
**3,143,890**	**0**	**-1**	**0**	**2260**	**2**	**4100**	**Rv2837c**		**Conserved protein**	**n/a**
**3,235,485**	**0**	**1**	**1**	**2260**	**0**	**4100**	**Rv2922c**	**Smc**	**Probable chromosome partition protein Smc**	**n/a**
3,371,365	0	1	4100	2250	9	3	Rv3011c	GatA	Probable glutamyl-tRNA(GLN) amidotransferase (subunit A) GatA (Glu-ADT subunit A)	In vitro essential per multiple studies (Minato 2019; DeJesus 2017; Sassetti 2003; Griffin 2011)
3,534,980	0	1	4100	2250	9	3	Rv3166c		Conserved hypothetical protein	n/a
**3,773,023**	**0**	**-1**	**0**	**2260**	**2**	**4100**	**Rv3361c**		**Conserved protein**	**n/a**
3,877,256	0	1	4100	2250	9	3	Rv3456c	RplQ	50S ribosomal protein L17 RplQ	In vitro essential (Minato 2019; Griffin 2011), or mutant shows growth defect (DeJesus 2017)
3,904,490	0	1	4100	2250	9	3	Rv3484	CpsA	Possible conserved protein CpsA	Essential in murine spleen (Sassetti and Rubin, 2003)
**3,922,919**	**0**	**-1**	**0**	**2260**	**1**	**4100**	**Rv3504**	**fadE26**	**Probable acyl-CoA dehydrogenase FadE26**	**n/a**
4,157,578	0	1	4100	2250	9	3	Rv3712		Possible ligase	In vitro essential per multiple studies (Minato 2019; DeJesus 2017; Sassetti 2003; Griffin 2011)
4,171,113	0	1	4100	2250	9	3	Rv3725		Possible oxidoreductase	Disruption provides growth advantage (DeJesus 2017)
**4,281,133**	**0**	**-1**	**0**	**2260**	**2**	**4100**	**Rv3816c**		**Possible acyltransferase**	**n/a**

GWAS results by treeWAS showing single nucleotide polymorphisms (coordinate relative to MTB H37Rv in SNP column) associated with classification of MTBC isolates as M. *tuberculosis* variant *bovis* (MBO). For SNPs within genes or ORFs, the classification and putative function is listed, as well as select information about essentiality by transposon mutagenesis studies from Mycobrowser [https://mycobrowser.epfl.ch/]. Likely false positive calls of association for SNPs with only 1–2 occurrences are highlighted by **bolded rows**

As mentioned for the spurious hit for UK samples, the simultaneous test can report loci as associated even if a SNP is only present in a few isolates. These false positives are included in the data table but are marked by bolding to indicate they are not meaningful. Subtracting these spurious hits, the simultaneous test identifies 22 loci, all of which are also identified by the subsequent test. The 120 loci concordantly identified as associated by *Bovidae*, MBO, and *Homo sapiens* by the subsequent test are listed in Table 2.

**Table 2:** SNPs significantly associated with classification as MBO by Subsequent statistical test

SNP	*p*-value	score	G1P1	G0P0	G1P0	G0P1	Locus	Protein	Description	Essentiality Notes from Mycobrowser	SnpEff Effects
22,264	0	0.948	4100	2080	172	1	Rv0018c	PstP	Involved in regulation (using dephosphorylation of a specific phosphorylated substrate)	Required for survival in murine macrophages (Rengarajan 2005)	Synonymous
23,714	0	0.951	4100	2100	161	1	Rv0019c	FhaB	Conserved protein with FHA domain, FhaB	Required for survival in murine macrophages (Rengarajan 2005)	Synonymous
147,873	0	0.998	4100	2250	9	3			Intergenic, upstream of elongation factor G FusA2 (Rv0120c)		Intergenic
181,672	0	0.951	4100	2100	161	1	Rv0153c	PtbB	Phosphotyrosine protein phosphatase PTPB (protein-tyrosine-phosphatase) (PTPase)	Required for growth on cholesterol (Griffin 2011)	Asp105Gly
184,727	0	0.998	4100	2250	9	3	Rv0156	PntAb	Probable NAD(P) transhydrogenase (subunit alpha) PntAb [second part; integral membrane protein] (pyridine nucleotide transhydrogenase subunit alpha) (nicotinamide nucleotide transhydrogenase subunit alpha)		Tyr2Cys
212,254	0	0.951	4100	2100	161	1			Intergenic, upstream of transmembrane protein (Rv0180)		Intergenic
217,863	0	0.951	4100	2100	160	1	Rv0186	BglS	Possibly involved in degradation [catalytic activity: hydrolysis of terminal, non-reducing beta-D-glucose residues with release of beta-D-glucose]		Pro532Arg
262,160	0	0.997	4100	2250	9	8	Rv0218		Probable conserved transmembrane protein	Essential in murine spleen (Sassetti and Rubin, 2003)	Asp413Asn
268,277	0	0.998	4100	2250	9	3	Rv0224c		Possible methyltransferase (methylase)	In vitro essential per multiple studies (Minato 2019; DeJesus 2017; Sassetti 2003; Griffin 2011)	Phe117Leu
277,862	0	0.998	4100	2250	9	3			Intergenic, downstream of FadE4 (Rv0231) and upstream of probable transcriptional regulatory protein (probably TetR/AcrR-family) (Rv0232)		Intergenic
294,198	0	0.997	4100	2250	9	8	Rv0244c	FadE5	Probable acyl-CoA dehydrogenase FadE5	Required for growth on cholesterol (Griffin 2011)	Glu479Ala
386,060	0	0.997	4100	2250	9	8			Intergenic, upstream of glpQ2 (Rv0317c)		Intergenic
397,386	0	0.951	4100	2100	160	1			Intergenic, downstream of putative dehydrogenase/reductase (Rv0331) and upstream of hypothetical protein (Rv0332)		Intergenic
398,034	0	0.997	4100	2250	9	8	Rv0332		Conserved protein		Glu198Gly
411,100	0	0.948	4100	2090	171	2	Rv0342	IniA	Isoniazid inductible gene protein IniA		Asn88Ser
1,027,445	0	0.997	4100	2250	9	8	Rv0921		Possible resolvase for IS1535		Synonymous
1,029,936	0	0.951	4100	2100	161	1	Rv0923c		Conserved hypothetical protein		Synonymous
1,125,316	0	0.951	4100	2100	161	1	Rv1006		Unknown protein	Disruption provides growth advantage (DeJesus 2017)	Pro535Ser
1,129,160	0	0.997	4100	2250	9	9	Rv1010	KsgA	Probable dimethyladenosine transferase KsgA (S-adenosylmethionine-6-N', N'-adenosyl(rRNA) dimethyltransferase) (16S rRNA dimethylase) (high level kasugamycin resistance protein KsgA) (kasugamycin dimethyltransferase)		Synonymous
1,129,160	0	0.997	4100	2250	9	9	Rv1009	RpfB	Probable resuscitation-promoting factor RpfB		Ala357Val
1,234,657	0	0.998	4100	2250	9	3	Rv1108c	XseA	Probable exodeoxyribonuclease VII (large subunit) XseA (exonuclease VII large subunit)		Synonymous
1,260,537	0	0.951	4100	2100	161	1	Rv1133c	MetE	Probable 5-methyltetrahydropteroyltriglutamate–homocysteine methyltransferase MetE (methionine synthase, vitamin-B12 independent isozyme)	In vitro essential (DeJesus 2017; Sassetti 2003; Griffin 2011), non-essential in rich media (Minato 2019)	Synonymous
1,307,958	0	0.951	4100	2100	161	1	Rv1175c	FadH	Probable NADPH dependent 2,4-dienoyl-CoA reductase FadH (2,4-dienoyl coenzyme A reductase) (4-enoyl-CoA reductase)		Thr90Asn
1,377,140	0	0.948	4100	2080	172	1	Rv1234		Probable transmembrane protein		Glu55Glu
1,393,003	0	0.951	4100	2100	161	1	Rv1248c		Multifunctional alpha-ketoglutarate metabolic enzyme	In vitro essential per multiple studies (Minato 2019; Sassetti 2003; Griffin 2011; Carvalho 2010)	Glu17Ala
1,425,641	0	0.951	4100	2100	161	1	Rv1276c		Conserved hypothetical protein		Thr92Ser
1,458,076	0	0.951	4100	2100	161	1	Rv1301		Conserved protein	In vitro essential (Sassetti 2003; Griffin 2011), non-essential in rich media (Minato 2019)	Synonymous
1,478,312	0	0.998	4100	2250	9	3	Rv1317c	AlkA	Probable bifunctional regulatory protein and DNA repair enzyme AlkA (regulatory protein of adaptative response) (methylphosphotriester-DNA–protein-cysteine S-methyltransferase)		Synonymous
1,496,289	0	0.997	4100	2250	9	8	Rv1328	GlgP	Probable glycogen phosphorylase GlgP		Val576Phe
1,499,291	0	0.998	4100	2250	9	4	Rv1330c	PncB1	Nicotinic acid phosphoribosyltransferase PncB1		Gly423Gly
1,562,049	0	0.997	4100	2250	9	8	Rv1387	PPE20	PPE family protein PPE20		Val94Ala
1,609,445	0	0.948	4100	2080	172	1	Rv1431		Conserved membrane protein		Lys455Gln
1,671,658	0	0.997	4100	2250	9	8	Rv1481		Probable membrane protein	In vitro essential per multiple studies (Minato 2019; DeJesus 2017; Griffin 2011)	Synonymous
1,681,928	0	0.951	4100	2100	161	1	Rv1491c		Conserved membrane protein		Synonymous
1,684,979	0	0.948	4100	2080	172	1	Rv1493	MutB	Probable methylmalonyl-CoA mutase large subunit MutB (MCM)		Synonymous
1,739,294	0	0.998	4100	2250	9	3	Rv1536	IleS	Isoleucyl-tRNA synthetase IleS	In vitro essential per multiple studies (Minato 2019; DeJesus 2017; Griffin 2011; Lamichhane 2003)	Pro926Ala
1,754,572	0	0.951	4100	2100	161	1	Rv1550	FadD11	Probable fatty-acid-CoA ligase FadD11 (fatty-acid-CoA synthetase) (fatty-acid-CoA synthase)		Leu286Ser
1,766,620	0	0.951	4100	2100	161	1	Rv1562c	TreZ	Maltooligosyltrehalose trehalohydrolase TreZ		Ala175Thr
1,794,234	0	0.948	4100	2080	172	1	Rv1593c		Conserved protein		Synonymous
1,804,248	0	0.951	4100	2100	161	1	Rv1604	ImpA	Probable inositol-monophosphatase ImpA (imp)		Synonymous
1,804,315	0	0.997	4100	2250	9	8	Rv1604	ImpA	Probable inositol-monophosphatase ImpA (imp)		Tyr93His
1,830,295	0	0.998	4100	2250	9	3	Rv1628c		Conserved protein		Synonymous
1,834,859	0	0.951	4100	2100	161	1	Rv1630	RpsA	30S ribosomal protein S1 RpsA	In vitro essential per multiple studies (Minato 2019; DeJesus 2017; Griffin 2011; Sassetti 2003)	Ala440Thr
1,971,029	0	0.997	4100	2250	9	8	Rv1744c		Probable membrane protein		Arg121Gln
2,013,589	0	0.951	4100	2100	160	1	Rv1779c		Possible integral membrane protein		Synonymous
2,082,865	0	0.997	4100	2250	10	8	Rv1836c		Conserved protein		Arg591His
2,092,688	0	0.948	4100	2080	172	1	Rv1843c	GuaB1	Probable inosine-5'-monophosphate dehydrogenase GuaB1(imp dehydrogenase) (IMPDH) (IMPD)	Disruption provides growth advantage (DeJesus 2017)	Synonymous
2,104,270	0	0.997	4100	2250	9	8	Rv1856c		Possible oxidoreductase	Disruption provides growth advantage (DeJesus 2017)	Arg185His
2,280,081	0	0.951	4100	2100	161	1	Rv2032	Acg	Conserved protein Acg		Pro318Leu
2,475,116	0	0.997	4100	2250	9	8	Rv2210c	IlvE	Branched-chain amino acid transaminase IlvE	In vitro essential (Sassetti 2003; Griffin 2011; DeJesus 2017), non-essential in rich media (Minato 2019)	Synonymous
2,475,888	0	0.998	4100	2250	9	3	Rv2210c	IlvE	Branched-chain amino acid transaminase IlvE	In vitro essential (Sassetti 2003; Griffin 2011; DeJesus 2017), non-essential in rich media (Minato 2019)	Glu28Ala
2,502,757	0	0.951	4100	2100	161	1	Rv2229c		Conserved protein		Arg239Gln
2,528,773	0	0.998	4100	2250	9	3	Rv2254c		Probable integral membrane protein		Ala68Val
2,529,798	0	0.951	4100	2100	161	1	Rv2256c		Conserved hypothetical protein		Ala26Gly
2,606,813	0	0.951	4100	2100	161	1	Rv2333c	Stp	Integral membrane drug efflux protein Stp		His503Gln
2,646,542	0	0.951	4100	2100	161	1	Rv2364c	Era	Probable GTP-binding protein Era	In vitro essential (Sassetti 2003; Griffin 2011), non-essential in rich media (Minato 2019)	Synonymous
2,658,676	0	0.998	4100	2250	9	3	Rv2379c	MbtF	Peptide synthetase MbtF (peptide synthase)		Ala1137Val
2,659,542	0	0.951	4100	2100	161	1	Rv2379c	MbtF	Peptide synthetase MbtF (peptide synthase)		Synonymous
2,682,593	0	0.998	4100	2250	9	3	Rv2388c	HemN	Probable oxygen-independent coproporphyrinogen III oxidase HemN (coproporphyrinogenase) (coprogen oxidase)	Essential in murine spleen (Sassetti and Rubin, 2003)	Ala184Thr
2,692,875	0	0.997	4100	2250	9	8	Rv2396	AprC	Acid and phagosome regulated protein C, PE-PGRS family protein PE_PGRS41		Ser26Asn
2,760,147	0	0.951	4100	2100	161	1	Rv2458	MmuM	Probable homocysteine S-methyltransferase MmuM (S-methylmethionine:homocysteine methyltransferase) (cysteine methyltransferase)	Disruption provides growth advantage (DeJesus 2017)	Synonymous
2,809,318	0	0.951	4100	2100	161	1	Rv2495c	BkdC	Probable branched-chain keto acid dehydrogenase E2 component BkdC		Arg208Trp
2,812,742	0	0.951	4100	2100	161	1	Rv2498c	CitE	Probable citrate (pro-3S)-lyase (beta subunit) CitE (citrase) (citratase) (citritase) (citridesmolase) (citrase aldolase)		Synonymous
2,817,446	0	0.997	4100	2250	9	8	Rv2502c	AccD1	Probable acetyl-/propionyl-CoA carboxylase (beta subunit) AccD1	Essential in murine spleen (Sassetti and Rubin, 2003)	Phe343Leu
2,912,516	0	0.998	4100	2250	9	3	Rv2585c		Possible conserved lipoprotein		Synonymous
2,927,291	0	0.993	4080	2250	9	21	Rv2598		Conserved hypothetical protein	Disruption provides growth advantage (DeJesus 2017)	Synonymous
2,932,890	0	0.951	4100	2100	161	1	Rv2605c	TesB2	Probable acyl-CoA thioesterase II TesB2 (TEII)		Phe85Leu
2,997,325	0	0.951	4100	2100	161	1	Rv2681		Conserved hypothetical alanine rich protein	Required for growth on cholesterol (Griffin 2011)	Ala196Val
3,032,137	0	0.951	4100	2100	161	1	Rv2720	LexA	Repressor LexA		Val117Ala
3,041,679	0	0.951	4100	2100	161	1	Rv2729c		Probable conserved integral membrane alanine valine and leucine rich protein		Ala266Val
3,042,353	0	0.997	4100	2250	9	8	Rv2729c		Probable conserved integral membrane alanine valine and leucine rich protein		Phe41Leu
3,055,922	0	0.997	4100	2250	9	8	Rv2742c		Conserved hypothetical arginine rich protein		Synonymous
3,140,153	0	0.998	4100	2250	9	4	Rv2833c	UgpB	Probable Sn-glycerol-3-phosphate-binding lipoprotein UgpB		Ser111Ile
3,142,580	0	0.951	4100	2100	161	1	Rv2836c	DinF	Possible DNA-damage-inducible protein F DinF		Pro350Leu
3,152,421	0	0.995	4090	2250	9	13	Rv2845c	ProS	Probable prolyl-tRNA synthetase ProS (proline–tRNA ligase) (PRORS) (global RNA synthesis factor) (proline translase)	Essential in vitro (Minato 2019; DeJesus 2017; Griffin 2011; Sassetti 2003) and in murine spleen (Sassetti and Rubin 2003)	His177Arg
3,157,785	0	0.951	4100	2100	161	1	Rv2849c	CobO	Probable cob(I)alamin adenosyltransferase CobO (corrinoid adenosyltransferase) (corrinoid adotransferase activity)		Trp120Cys
3,158,719	0	0.997	4100	2250	9	8	Rv2850c		Possible magnesium chelatase		Gly446Ser
3,159,237	0	0.951	4100	2100	161	1	Rv2850c		Possible magnesium chelatase		Arg273Gln
3,174,591	0	0.951	4100	2100	161	1	Rv2862c		Conserved hypothetical protein		Arg18Pro
3,189,664	0	0.951	4100	2100	161	1	Rv2879c		Conserved hypothetical protein		Synonymous
3,198,332	0	0.951	4100	2100	161	1	Rv2889c	Tsf	Probable elongation factor Tsf (EF-ts)	In vitro essential (Sassetti 2003; Griffin 2011; DeJesus 2017; Minato 2019)	Thr259Met
3,213,089	0	0.951	4100	2100	161	1	Rv2903c	LepB	Probable signal peptidase I LepB (SPASE I) (leader peptidase I)	In vitro essential (Sassetti 2003; Griffin 2011; DeJesus 2017; Minato 2019)	Asp256Asn
3,223,303	0	0.997	4100	2250	9	8	Rv2914c	PknI	Probable transmembrane serine/threonine-protein kinase I PknI (protein kinase I) (STPK I) (phosphorylase B kinase kinase) (hydroxyalkyl-protein kinase)	Required for growth on cholesterol (Griffin 2011), mutant shows increased growth in THP-1 cells, SCID mice show faster mortality with mutant (Gopalaswamy 2009)	Synonymous
3,235,715	0	0.997	4100	2250	9	8	Rv2922c	Smc	Probable chromosome partition protein Smc		Arg698Gly
3,254,695	0	0.951	4100	2100	161	1	Rv2932	PpsB	Phenolpthiocerol synthesis type-I polyketide synthase PpsB	In vitro essential in CDC1551 (Lamichhane 2003), not in H37Rv (Griffin 2011; DeJesus 2017; Minato 2019)	Synonymous
3,262,628	0	0.951	4100	2100	161	1	Rv2934	PpsD	Phenolpthiocerol synthesis type-I polyketide synthase PpsD		Met127Ile
3,267,715	0	0.951	4100	2100	161	1	Rv2934	PpsD	Phenolpthiocerol synthesis type-I polyketide synthase PpsD		Glu1823Ala
3,282,079	0	0.951	4100	2100	161	1	Rv2940c	Mas	Probable multifunctional mycocerosic acid synthase membrane-associated Mas		Ser213Cys
3,320,554	0	0.951	4100	2100	161	1	Rv2947c	Pks15	Probable polyketide synthase Pks15, involved in the biosynthesis of phenolphthiocerol glycolipids		Synonymous
3,355,417	0	0.997	4100	2250	9	9	Rv2997		Possible alanine rich dehydrogenase		Cys107Ser
3,371,365	0	0.998	4100	2250	9	3	Rv3011c	GatA	Probable glutamyl-tRNA(GLN) amidotransferase (subunit A) GatA (Glu-ADT subunit A)	In vitro essential (Sassetti 2003; Griffin 2011; DeJesus 2017; Minato 2019)	Ala24Thr
3,388,682	0	0.951	4100	2100	161	1	Rv3029c	FixA	Probable electron transfer flavoprotein (beta-subunit) FixA (beta-ETF) (electron transfer flavoprotein small subunit) (ETFSS)	In vitro essential (Sassetti 2003; Griffin 2011), non-essential in rich media (Minato 2019)	Synonymous
3,517,567	0	0.997	4100	2250	9	8	Rv3151	NuoG	Probable NADH dehydrogenase I (chain G) NuoG (NADH-ubiquinone oxidoreductase chain G)		Synonymous
3,534,980	0	0.998	4100	2250	9	3	Rv3166c		Conserved hypothetical protein		Synonymous
3,540,144	0	0.951	4100	2100	161	1	Rv3171c	Hpx	Possible non-heme haloperoxidase Hpx		Thr201Met
3,565,449	0	0.951	4100	2100	161	1	Rv3195		Conserved hypothetical protein		Synonymous
3,594,851	0	0.997	4100	2250	9	8	Rv3218		Conserved protein		Synonymous
3,595,427	0	0.951	4100	2100	161	1	Rv3218		Conserved protein		Synonymous
3,624,710	0	0.951	4100	2100	161	1	Rv3244c	LpqB	Probable conserved lipoprotein LpqB	In vitro essential (Sassetti 2003; Griffin 2011; DeJesus 2017; Minato 2019)	Synonymous
3,664,615	0	0.951	4100	2100	161	1	Rv3282		Conserved hypothetical protein		Ala133Ser
3,678,929	0	0.997	4100	2250	9	8	Rv3296	Lhr	Probable ATP-dependent helicase Lhr (large helicase-related protein)		Val719Met
3,690,854	0	0.951	4100	2100	161	1	Rv3303c	LpdA	NAD(P)H quinone reductase LpdA		Thr29Ala
3,770,588	0	0.951	4100	2100	161	1	Rv3356c	FolD	Probable bifunctional protein FolD: methylenetetrahydrofolate dehydrogenase + methenyltetrahydrofolate cyclohydrolase	In vitro essential (Sassetti 2003; Griffin 2011; DeJesus 2017; Minato 2019)	Gln21Pro
3,857,161	0	0.951	4100	2100	161	1	Rv3437		Possible conserved transmembrane protein	Disruption provides growth advantage (DeJesus 2017)	Leu84Pro
3,877,256	0	0.998	4100	2250	9	3	Rv3456c	RplQ	50S ribosomal protein L17 RplQ	In vitro essential (Minato 2019; Griffin 2011)	Synonymous
3,904,490	0	0.998	4100	2250	9	3	Rv3484	CpsA	Possible conserved protein CpsA	Essential in murine spleen (Sassetti and Rubin, 2003)	Synonymous
3,907,958	0	0.997	4100	2250	9	8	Rv3488		Conserved hypothetical protein		Gly98Arg
3,912,636	0	0.951	4100	2100	162	1	Rv3494c	Mce4F	Mce-family protein Mce4F	Required for growth on cholesterol (Griffin 2011)	Asp245Gly
3,924,350	0	0.951	4100	2100	161	1	Rv3505	FadE27	Probable acyl-CoA dehydrogenase FadE27		Ala218Val
3,977,910	0	0.997	4100	2250	9	8	Rv3538		Probable dehydrogenase. Possible 2-enoyl acyl-CoA hydratase		Synonymous
3,987,645	0	0.997	4100	2250	9	8	Rv3548c		Probable short-chain type dehydrogenase/reductase	Required for growth on cholesterol (Griffin 2011)	Met218Val
4,004,604	0	0.997	4100	2250	9	8	Rv3563	FadE32	Probable acyl-CoA dehydrogenase FadE32	Required for growth on cholesterol (Griffin 2011), essential in murine spleen (Sassetti and Rubin, 2003)	Gln105Arg
4,034,908	0	0.951	4100	2100	161	1	Rv3593	LpqF	Probable conserved lipoprotein LpqF	In vitro essential (Sassetti 2003; Griffin 2011; Minato 2019)	Asn186Ser
4,047,039	0	0.948	4100	2080	172	1	Rv3604c		Probable conserved transmembrane protein rich in alanine and arginine and proline	In vitro essential (Sassetti 2003; Griffin 2011; Minato 2019)	Val153Gly
4,083,511	0	0.951	4100	2100	161	1	Rv3645		Probable conserved transmembrane protein	In vitro essential (DeJesus 2017; Griffin 2011)	Synonymous
4,090,661	0	0.997	4100	2250	9	8	Rv3649		Probable helicase	Essential in murine spleen (Sassetti and Rubin, 2003)	Synonymous
4,157,578	0	0.998	4100	2250	9	3	Rv3712		Possible ligase	In vitro essential (Sassetti 2003; Griffin 2011; DeJesus 2017; Minato 2019)	Gly200Ser
4,171,113	0	0.998	4100	2250	9	3	Rv3725		Possible oxidoreductase	Disruption provides growth advantage (DeJesus 2017)	Synonymous
4,242,970	0	0.951	4100	2100	161	1	Rv3793	EmbC	Integral membrane indolylacetylinositol arabinosyltransferase EmbC (arabinosylindolylacetylinositol synthase)	In vitro essential (Sassetti 2003; Goude 2008; Griffin 2011; DeJesus 2017; Minato 2019)	Synonymous
4,278,968	0	0.951	4100	2100	161	1	Rv3813c		Conserved protein	n/a	Met83Thr

GWAS results by treeWAS showing single nucleotide polymorphisms (coordinate relative to MTB H37Rv in SNP column) associated with classification of MTBC isolates as M. *tuberculosis* variant *bovis* (MBO). For SNPs within genes or ORFs, the classification and putative function is listed, as well as select information about essentiality by transposon mutagenesis studies from Mycobrowser [https://mycobrowser.epfl.ch/]

The subset of loci called by both tests in MBO (*n* = 22) are presented in Table 3.

**Table 3:** SNPs concordantly significantly associated with classification as MBO by Subsequent and Simultaneous statistical tests

SNP	*p*-value	score	G1P1	G0P0	G1P0	G0P1	Locus	Protein	Description	Essentiality Notes from Mycobrowser
147,873	0	1	4100	2250	9	3			Intergenic, upstream of elongation factor G FusA2 (Rv0120c)	n/a
184,727	0	1	4100	2250	9	3	Rv0156	PntAb	Probable NAD(P) transhydrogenase (subunit alpha) PntAb [second part; integral membrane protein] (pyridine nucleotide transhydrogenase subunit alpha) (nicotinamide nucleotide transhydrogenase subunit alpha)	n/a
268,277	0	1	4100	2250	9	3	Rv0224c		Possible methyltransferase (methylase)	In vitro essential per multiple studies (Minato 2019; DeJesus 2017; Sassetti 2003; Griffin 2011)
277,862	0	1	4100	2250	9	3			Intergenic, downstream of FadE4 (Rv0231) and upstream of probable transcriptional regulatory protein (probably TetR/AcrR-family) (Rv0232)	n/a
1,234,657	0	1	4100	2250	9	3	Rv1108c	XseA	Probable exodeoxyribonuclease VII (large subunit) XseA (exonuclease VII large subunit)	n/a
1,478,312	0	1	4100	2250	9	3	Rv1317c	AlkA	Probable bifunctional regulatory protein and DNA repair enzyme AlkA (regulatory protein of adaptative response) (methylphosphotriester-DNA–protein-cysteine S-methyltransferase)	
1,499,291	0	1	4100	2250	9	4	Rv1330c	PncB1	Nicotinic acid phosphoribosyltransferase PncB1	n/a
1,739,294	0	1	4100	2250	9	3	Rv1536	IleS	Isoleucyl-tRNA synthetase IleS	In vitro essential per multiple studies (Minato 2019; DeJesus 2017; Lamichhane 2003; Griffin 2011)
1,830,295	0	1	4100	2250	9	3	Rv1628c		Conserved protein	n/a
2,475,888	0	1	4100	2250	9	3	Rv2210c	IlvE	Branched-chain amino acid transaminase IlvE	In vitro essential (DeJesus 2017; Sassetti 2003; Griffin 2011), non-essential in rich media (Minato 2019)
2,528,773	0	1	4100	2250	9	3	Rv2254c		Probable integral membrane protein	n/a
2,658,676	0	1	4100	2250	9	3	Rv2379c	MbtF	Peptide synthetase MbtF (peptide synthase)	n/a
2,682,593	0	1	4100	2250	9	3	Rv2388c	HemN	Probable oxygen-independent coproporphyrinogen III oxidase HemN (coproporphyrinogenase) (coprogen oxidase)	Essential in murine spleen (Sassetti and Rubin, 2003)
2,912,516	0	1	4100	2250	9	3	Rv2585c		Possible conserved lipoprotein	n/a
2,927,291	0	1	4080	2250	9	21	Rv2598		Conserved hypothetical protein	n/a
3,140,153	0	1	4100	2250	9	4	Rv2833c	UgpB	Probable Sn-glycerol-3-phosphate-binding lipoprotein UgpB	Disruption provides growth advantage (DeJesus 2017)
3,371,365	0	1	4100	2250	9	3	Rv3011c	GatA	Probable glutamyl-tRNA(GLN) amidotransferase (subunit A) GatA (Glu-ADT subunit A)	In vitro essential per multiple studies (Minato 2019; DeJesus 2017; Sassetti 2003; Griffin 2011)
3,534,980	0	1	4100	2250	9	3	Rv3166c		Conserved hypothetical protein	n/a
3,877,256	0	1	4100	2250	9	3	Rv3456c	RplQ	50S ribosomal protein L17 RplQ	In vitro essential (Minato 2019; Griffin 2011), or mutant shows growth defect (DeJesus 2017)
3,904,490	0	1	4100	2250	9	3	Rv3484	CpsA	Possible conserved protein CpsA	Essential in murine spleen (Sassetti and Rubin, 2003)
4,157,578	0	1	4100	2250	9	3	Rv3712		Possible ligase	In vitro essential per multiple studies (Minato 2019; DeJesus 2017; Sassetti 2003; Griffin 2011)
4,171,113	0	1	4100	2250	9	3	Rv3725		Possible oxidoreductase	Disruption provides growth advantage (DeJesus 2017)

GWAS results by treeWAS showing single nucleotide polymorphisms (coordinate relative to MTB H37Rv in SNP column) associated with classification of MTBC isolates as M. *tuberculosis* variant *bovis* (MBO). For SNPs within genes or ORFs, the classification and putative function is listed, as well as select information about essentiality by transposon mutagenesis studies from Mycobrowser [https://mycobrowser.epfl.ch/]. This list is a subset of only variants called in both Tables 1 and 2

After GWAS, several apparent genotypic edge cases arose. FadD11 was highlighted as significantly associated by a single non-synonymous variant, FadD11 L286S, which appeared fixed in MBO and MCP, while MTB and MOR showed WT nearly exclusively. Of 4,105 MBO isolates and 152 MCP isolates, only 1 MBO isolate showed WT at this position, suggesting a reversion in this genome. Likewise, of 2,087 MTB and 8 MOR isolates, only 9 MTB isolates showed L286S. These genotypic exceptions were checked further: a Chinese cattle isolate SRR16278270 for the 1 MBO outlier, and 9 UK MTB isolates from humans for the MTB outliers (Table 4).

**Table 4:** SNP typing improperly labeled MTBC isolates by MBO-lineage markers

Lineage Marker	Bovis		
**Accession ID**	**C1427476T**	**A2831482G**	**C3624593T**
ERR017796	✓	✓	✓
ERR026636	✓	✓	✓
ERR046747	✓	✓	✓
ERR046748	✓	✓	✓
ERR046749	✓	✓	✓
ERR046954	✓	✓	✓
ERR046961	✓	✓	✓
ERR046989	✓	✓	✓
ERR387001*	✓	✓	✓
SRR16278270	X	X	X

Nine pathogen isolates from humans (underlined) were classified when deposited into NCBI as MTB, but their genotypes by GWAS did not align with other MTB isolates. These isolates were checked for three MBO lineage-determining SNPs as reported by Coll et al*. *[24], and all 9 were found to possess these SNPs, indicating a misclassification in the database. This was confirmed by SNP-IT [25], which also reported one isolate was a BCG strain (starred). Conversely, one isolate (SRR16278270) was classified as MBO, but was shown not to possess any MBO lineage-determining SNPs, supporting a misclassification of an MTB isolate as MBO

^*^BCG strain

These 10 isolates’ VCF files were checked against the SNP barcode [24], with lineage-determining positions searched per VCF through Unix command “grep” and the SNP coordinate. The MBO isolate bore no MBO lineage-determining SNPs, and instead was cleanly typable as MTB lineage 2.2.1 (Table 5). Evidently, this isolate is a case of bovine MTB being incorrectly identified as MBO when uploaded to NCBI. Likewise, of the 9 human MTB cases that stood out, all bore the 3 lineage-determining SNPs for MBO (Table 4), and no MTB lineage-determining markers. In these cases, 9 cases of human MBO were misclassified as MTB. These results were validated using SNP-IT software [25], which also typed ERR387001 as a BCG strain, suggesting a case of BCG-osis misdiagnosed or mislabeled as TB. After correcting these calls, there is a perfect divide between MTB/MOR and MBO/MCP, with 100% of isolates bearing the WT for MTB/MOR, and 100% of isolates bearing the SNP for MBO/MCP. This discrepancy may have affected robustness of GWAS based on phenotype of “MBO variant.” However, it is noted that comparisons for “host *Bovidae”* and “host *Homo sapiens”* are unaffected by this, and all 3 analyses produced perfect concordance of their 120 associated loci by subsequent tests, suggesting this mislabeling had minimal influence. In summary, a set of 120 discriminatory loci was identified, which was also identified by bGWAS of phenotypes classified as a host of *Bovidae* (1) or not (0), and *Homo sapiens* (1) or not (0).

**Table 5:** SNP typing improperly labeled MBO isolate by MTB-lineage markers

Lineage Marker	Lineage 2	Lineage 2	Lineage 2	Lineage 2	Lineage 2.2	Lineage 2.2	Lineage 2.2	Lineage 2.2.1	Lineage 2.2.1
Accession ID	G497491A	C811753T	A1834177C	T2543395C	C1849051T	G2505085A	C2775361T	C797736T	C3498198T
SRR16278270	✓	✓	✓	✓	✓	✓	✓	✓	✓

Isolate SRR16278270 was deposited in NCBI as an MBO isolate from cattle, but its genotype by GWAS did not align with other MBO isolates. This isolate was checked for MTB lineage-determining SNPs as reported by Coll et al*. *[24], and was found to contain all SNPs for MTB lineage 2.2.1 and none for MBO (Table 4), indicating a misclassification in the database. This was confirmed by SNP-IT [25]

## Discussion

Despite remarkable similarity between MTB and MBO, evidence of clear divides was present in SNPs highlighted as associated by treeWAS analysis.

The Fad family of proteins are important in MTBC, with MTB known to carry 36 FadD and FadE loci [26, 27]. GWAS identified SNPs in FadD11, FadE5, FadE27, and FadE32 associated with differentiation into MBO. These genes are involved in fatty acid and cholesterol handling inside the environment of the host [28]. Mycobacterial reliance on cholesterol is known to be critical for pathogenesis, and MTB features around 80 genes involved with cholesterol balance and metabolism [29]. Disruption of cholesterol import is severely disruptive to infection and persistence [29, 30].

Cholesterol intake in MTBC requires a functional Mce4 system [30]. A missense mutation (A734G, Asp254Gly) in Mce4F was seen in all MBO/MCP isolates, and only a single MTB isolate from Russia (ERR108427) which bore a unique SNP signature: G3836739A (lineage 4.8), and G1759252T (lineage 4.9). No other SNPs for lineage 4 or any other lineage were identified. A literature search turned up Congo type MTB that can present lineage 4.8 and 4.9 SNPs, but only in combination with 4.7 SNPs that were absent in this sample [31]. Except this atypical MTB specimen, another split by this *mce4f* SNP separated MTB/MOR and MBO/MCP*.*

Catabolized cholesterol products fuel core acyl-CoA metabolism pathway, as well as polyketide synthesis, a pathway already known to differ between MBO and MTB [32]. GWAS identified two separate missense mutations in *ppsD*, and synonymous changes in *ppsB* and *pks15*, all polyketide synthase genes. Genes annotated in roles of cholesterol and fatty acid metabolism or in pathways downstream of these processes are among all loci identified by the MBO subsequent tests are shown in Table 6. These associated SNPs are scattered across lipid metabolic pathways and include members whose exact function is unclear. Ten out of fourteen of these SNPs are non-synonymous.

**Table 6:** Genes identified by GWAS associated with fatty acid and cholesterol metabolism

SNP	*p*-value	score	G1P1	G0P0	G1P0	G0P1	Locus	Protein	Change	Description	Notes
181,672	0	0.951	4100	2100	161	1	Rv0153c	PtbB	Asp105Gly	Phosphotyrosine protein phosphatase PTPB (protein-tyrosine-phosphatase) (PTPase)	Required for growth on cholesterol (Griffin 2011)
294,198	0	0.997	4100	2250	9	8	Rv0244c	FadE5	Glu479Ala	Probable acyl-CoA dehydrogenase FadE5	Required for growth on cholesterol (Griffin 2011)
1,684,979	0	0.948	4100	2080	172	1	Rv1493	MutB	Synonymous	Probable methylmalonyl-CoA mutase large subunit MutB (MCM)	Downstream in cholesterol to propionyl-CoA metabolic pathways (Wilburn 2018)
1,754,572	0	0.951	4100	2100	161	1	Rv1550	FadD11	Leu286Ser	Probable fatty-acid-CoA ligase FadD11 (fatty-acid-CoA synthetase) (fatty-acid-CoA synthase)	
2,997,325	0	0.951	4100	2100	161	1	Rv2681		Ala196Val	Conserved hypothetical alanine rich protein	Required for growth on cholesterol (Griffin 2011)
3,223,303	0	0.997	4100	2250	9	8	Rv2914c	PknI	Synonymous	Probable transmembrane serine/threonine-protein kinase I PknI (protein kinase I) (STPK I) (phosphorylase B kinase kinase) (hydroxyalkyl-protein kinase)	Required for growth on cholesterol (Griffin 2011), mutant shows increased growth in THP-1 cells, SCID mice show faster mortality with mutant (Gopalaswamy 2009)
3,912,636	0	0.951	4100	2100	162	1	Rv3494c	Mce4F	Asp245Gly	Mce-family protein Mce4F	Required for growth on cholesterol (Griffin 2011)
3,924,350	0	0.951	4100	2100	161	1	Rv3505	FadE27	Ala218Val	Probable acyl-CoA dehydrogenase FadE27	
3,987,645	0	0.997	4100	2250	9	8	Rv3548c		Met218Val	Probable short-chain type dehydrogenase/reductase	Required for growth on cholesterol (Griffin 2011)
4,004,604	0	0.997	4100	2250	9	8	Rv3563	FadE32	Gln105Arg	Probable acyl-CoA dehydrogenase FadE32	Required for growth on cholesterol (Griffin 2011), essential in murine spleen (Sassetti and Rubin, 2003)
3,254,695	0	0.951	4100	2100	161	1	Rv2932	PpsB	Synonymous	Phenolpthiocerol synthesis type-I polyketide synthase PpsB	In vitro essential in CDC1551 (Lamichhane 2003), not in H37Rv (Griffin 2011; DeJesus 2017; Minato 2019)
3,262,628	0	0.951	4100	2100	161	1	Rv2934	PpsD	Met127Ile	Phenolpthiocerol synthesis type-I polyketide synthase PpsD	
3,267,715	0	0.951	4100	2100	161	1	Rv2934	PpsD	Glu1823Ala	Phenolpthiocerol synthesis type-I polyketide synthase PpsD	
3,320,554	0	0.951	4100	2100	161	1	Rv2947c	Pks15	Synonymous	Probable polyketide synthase Pks15, involved in the biosynthesis of phenolphthiocerol glycolipids	

A subset of genes in the pathways of lipid and cholesterol intake, metabolism, and utilization were identified with SNPs by GWAS, with a split roughly between one genotype in MTB ± MOR and a divergent genotype in MBO ± MCP. Columns 2–5 indicate presence of genotype **G** (SNP = 1, WT = 0) and phenotype **P** (MBO classification = 1, non-MBO classification = 0). The misclassification of 9 MBO isolates as MTB, and 1 MTB isolate as MBO (Tables 4, 5) are evident in the G1P0 and G0P1 columns for many variants

Other identified loci with functions separate from cholesterol and lipid metabolism include AccD1, involved in leucine degradation [33], which bore a fixed SNP of Phe343Leu. SNP 1739294 in the essential isoleucyl-tRNA synthetase IleS causes a Pro926Ala substitution, SNP 3152421 in the essential prolyl-tRNA synthetase ProS yields His177Arg, SNP 3371365 in the essential glutamyl-tRNA amidotransferase subunit GatA causes Ala24Thr, and a synonymous change is seen at 1,260,537 for methionine synthesis gene MetE. Related to translational machinery, SNP 3198332 causes Thr259Met in essential elongation factor Tsf. SNP 1129160 impacts both RpfB (Resuscitation promoting factor B) and KsgA (a dimethyladenosine transferase) genes, resulting in RpfB Ala357Val and a synonymous mutation in KsgA. RpfB is thought to be involved in the transition from dormancy to active replication, and is co-transcribed with *ksgA* and *ispE*, genes involved in ribosome maturation and cell wall synthesis, respectively [34]. After accounting for mislabeling cases in deposited isolates, these SNPs are all fixed and exclusive in this dataset either in MBO and MCP, or MBO alone.

MTBC physiology and function remain uncertain, and 48/120 loci identified are in genes annotated only by locus identifier and generically, like “conserved protein” or “possible oxidoreductase” even after PE/PPE gene filtering, removing a largely uncharacterized family comprising ~ 10% of MTBC genes. Even among genes with fuller annotations, nearly all include “probable” in their descriptions. The genes presented in Table 6 are not comprehensive and given the uncertainty in function across many loci, other important genes both inside and outside lipid metabolism almost certainly exist in the bGWAS output of Table 2. Loci associated with adaptation towards new hosts and lifestyles are useful then to highlight for characterization, as it narrows the still vast pool of MTBC genes with uncertain functions towards a subset of genes with fixed changes in some variants.

Any associated loci may signify adaptive roles in differentiation from a specialist infection by MTB and a pathogen with a much broader host range, like MBO. It is well-reported that members of the MTBC are clonal, and not only is horizontal gene transfer vanishingly rare, mutation rates in members of this complex are low (~ 2 × 10^–10^ mutations per cell division) [35]. Given 99.95% genetic identity between MTB and MBO, < 2000 polymorphisms differentiate divergent variants (disregarding RDs/large sequence polymorphisms), and fixed changes are an even smaller subset. While this research cannot draw conclusions about how specific changes might alter metabolism or virulence to better reflect new host environs, it does highlight multiple SNPs across multiple genes in MTBC metabolic pathways. Metabolic differences are known to exist between variants and even between lineages of MTB, including in lipid profile [36, 37]. While these data are only associations, they may support findings by Griffin et al*.* reporting cholesterol utilization in MTB is key to host adaptation [29]. MTB drives macrophages to import lipids for utilization as an energy and carbon source [38–41]. The human cholesterol profile is LDL-dominant [42], as is the guinea pig [42], a model of tuberculosis that better recapitulates human disease [43] vs. the mouse model [5, 43, 44], an animal model with an HDL-dominant cholesterol profile and a lower overall cholesterol load [45, 46]. The MBO bovine host is HDL-dominant, for comparison [47]. Others have reported MTB infections are influenced differently by HDL vs. LDL cholesterol [48]. MTB is known to exploit lipid-rich “foamy macrophages,” and research has shown MTB trehalose dimycolate and other factors are associated with lipid droplet and foamy macrophage formation [38–40]. Foamy macrophage formation is associated with higher levels and intake of circulating LDL cholesterol, but recent research found MTB-infected macrophages have different lipid profiles from foamy macrophages characterized in atherosclerosis and other diseases [45], indicating disease-specific responses lead to buildup of certain lipids [41]. Finally, higher HDL levels are known to counteract foamy macrophage formation through classical LDL intake, HDL suppresses TNFα production in MTB-infected macrophages, and mice are more resistant to foamy macrophage formation, compared to humans, guinea pigs, rabbit, or primate models [42, 45, 48]. Variation in use of cholesterol and fatty acids is known to exist between MTBC variants and lineages. Finally, though research is more limited in this area, studies have demonstrated mice are more susceptible to disease and death by MBO infection than by MTB [49, 50]. Biological reality is undoubtedly far more complex, but from existing literature, host lipid profiles differ, lipid availability and sequestration are key to MTB virulence, animal models with a lipid profile closer to humans better reproduce “classic” granulomatous tuberculosis by MTB as seen in humans, and MTB lineages and MTBC variants utilize lipids differentially. Cholesterol/fatty acid metabolic pathways associated by bGWAS showing variant-specific between MTB and MBO are suggestive of a potential contributor towards host adaptation.

Adaptation to specific hosts was not detectable with this approach, which could be improved through routine sequencing of isolates from non-standard host types, which are currently rare and geographically biased. While efforts were taken to select from countries worldwide, the dataset itself is necessarily a biased sampling as well – very few isolates of the vast number of infections worldwide are ever sequenced, and of those that are, even fewer meet the inclusion criteria for metadata and sequencing coverage. Furthermore, in many clinical MTB genomes utilized in this work, evolutionary pressures imparted by antibiotic use are a powerful influence on SNP distribution, and drug-resistance associated SNPs were not filtered from the input dataset. This adds an additional layer of bias in that antibiotic selection pressures vary wildly across MTBC members. For future studies and adaptations of these findings, it is critical to note that our use of MTB H37Rv as a reference to study the entire MTBC and its evolutionary history is necessarily missing data. While the use of this reference as a standard is common practice and the H37Rv coordinates are typically how MTB SNPs are defined, the H37Rv lineage is a modern one, and any regions of difference absent in H37Rv but present in other MTBC members will not be SNP-called by our method, excluding potentially tens of thousands of base pairs. Future studies should consider using more “ancestral” variants like *M. canettii*, performing an MBO-specific bGWAS to try to identify host adaptation within the variant, or even by constructing an MTBC pangenome to call SNPs against for the maximum possible number of informative protein-coding changes. We hope our research serves as a launching point for these studies, for more routine sequencing and better metadata classification of isolates across the MTBC, and for better understanding of MTBC divergence, including through generation of time-measured phylogenies to estimate SNP occurrences through evolutionary history.

## Conclusions

Analysis of the genetic differences between MTBC isolates and their association with classification as certain variants or isolation from certain hosts by GWAS was performed. The 120 SNPs identified through this analysis provide a trove of genes and pathways implicated in adaptation towards a generalist lifecycle, including loci across cholesterol and fatty acid uptake, catabolism, and downstream processing pathways, important for central metabolism in MTBC organisms and critical for pathogenesis [28, 36, 38, 39, 51]. The understanding of host adaptation in *Mycobacterium* is a major outstanding knowledge gap. While tremendous work has been performed by groups worldwide, the mechanisms of how exactly MTBC members present different and infect different hosts is unresolved. Detail transcriptomic profiling has been performed in cattle infections by MTB and MBO, which demonstrate that macrophages have very distinct response profiles to each variant, as well as what appears to be a fine-tuned engagement with the bovine innate immune system for MBO [8]. It is known that MTB is attenuated in cattle [8], and that cellular-level differences manifest in overall disease course differences in other hosts like mice [49]. As discussed above, one rare aspect of TB pathogenesis is its critical utilization of host cholesterol for survival and success [30], and prior research within MTB hypothesizes human population-specific cholesterol utilization adaptation has occurred across MTB lineages [4]. Multiple studies have found that cholesterol utilization is in fact fundamental to pathogenesis, as covered in the review by Moopanar and Mvubu [40]. While the findings in this work are only suggestive, they again highlight cholesterol and fatty acid-associated genetic pathways, and future research in the lab should assess how loci identified herein may functionally differ in the context of different host pathways for cholesterol metabolism. Available lipid and cholesterol pools in bovine, murine, and other non-human hosts, differences between host cholesterol and lipid profiles, and the possible lineage-specific adaptation identified in the loci in this analysis could contribute to the elusive mechanisms of pathogen host preferences.

In summary, these SNPs reliably differentiate MTBC variants, and importantly, they may inform research into genes that differ between variants, narrowing the pool of uncharacterized proteins to study in the MTBC.

## Methods

Existing datasets were collected, including those where bGWAS was performed to answer other questions. Dong et al. (2022) genome-sequenced 74 Chinese cattle MBO isolates, and performed bGWAS analysis on 3,227 total MBO isolates from around the world [52]. Additionally, sequences used by Coll et al*.* in designing the MTBC SNP barcode are a validated set of primarily human MTB isolates [24]. Both datasets were included in this analysis, along with many smaller sets. FASTQ download URLs were acquired through SRA-Explorer [53], formatted for Globus-CLI [54, 55] and downloaded to MSU’s High Performance Computing Center (HPCC) for processing. After transfer, single-end read data were excluded, and Snippy [56] run with default parameters (BWA base quality = 13, minimum SNP coverage = 10 reads, minimum VCF SNP quality = 100), paired-end read input, and MTB H37Rv as the reference genome (AL123456.3). A complete list of accession IDs for all sequences used are provided in Additional file 1. Rare cases of genomes with unusually high numbers of SNPs (> 3,000) were excluded, as were genomes with alignment coverage < 90% of the reference length (cutoff < 3.96mbp). Additionally, metadata were compiled for all isolates (Additional file 1), and only datasets with host, MTBC variant, and country of isolation were included. Taxonomic classification by Kraken 2 [57] revealed several isolates primarily (> > 50%) contained plant or insect genomes, and were excluded, at which point remaining samples contained classified reads mapping primarily to *Mycobacterium*. Remaining paired-end read sets were selected (n = 6,360) to build the final “snippy-core” core SNP alignment, along with the H37Rv reference and *M. canettii* (GCF_000253375.1) as an outgroup, while masking PE/PPE genes using the H37Rv-specific.bed file provided by default in Snippy. Core SNPs were used for phylogenetic tree generation in RAxML-NG [20] (substitution model GTR + G selected by ModelTest-NG [58]; bootstrap analysis: seed = 774,900,118, bootstrap trees = 300; for tree search analysis: seed = 4,949,250,770, 50 parsimony-based, 50 random-based starting trees for tree search; applying bootstrap support to best ML tree: –consense MRE). On a Windows 10 desktop PC, RStudio (2022.07.2 + 576) [59], R (v4.0.5) [60], and the R package vcfR (v1.12.0) [61] were used to generate a vcfR object for import with prewas [21]. In prewas (v1.1.1), the VCF object containing variant calls was processed with an input tree generated from RAxML-NG, the H37Rv GFF3 file, and with ancestral reconstruction flag set to TRUE, for maximum likelihood-based reconstruction through the ape R package it uses. On an HPCC cluster, a Conda [62] environment was created containing GCC (v11.2.0) [63], OpenMPI (v4.1.1) [64], and R (v4.1.2). The R package devtools (v2.4.5) [65] was installed, and used to install prewas (v1.1.1) [66] and treeWAS (v1.1) [67] from Github. The RData object containing prewas output from the desktop PC was uploaded to HPCC and used for ancestral reconstruction state, binary variant matrix, and phylogenetic tree inputs, along with binary metadata phenotype matrices. All other parameters were left at their defaults. For MTBC lineage determination, the Coll et al*.* SNP barcode and SNP-IT tool were used [24, 25]. SnpEff (v4.2) was used to annotate variants separately [68]. TreeWAS generated default Manhattan plots and distribution graphics, and text output was collected in.csv files.

### Supplementary Information

**Additional file 1.**

**Additional file 2.**

**Additional file 3.**

**Additional file 4.**

**Additional file 5.**

**Additional file 6.**

**Additional file 7.**

**Additional file 8.**

**Additional file 9.**

## Data Availability

All sequence data used in this project are publicly available through NCBI and ENA. Accession IDs for all data are recorded in the table Additional file 1, with BioProjects in Column A, and corresponding SRA identifiers in Column B per sequence.

## References

[1] Mostowy S, Inwald J, Gordon S, Martin C, Warren R, Kremer K (2005). Revisiting the evolution of Mycobacterium bovis. J Bacteriol.

[2] Stucki D, Brites D, Jeljeli L, Coscolla M, Liu Q, Trauner A (2016). Mycobacterium tuberculosis lineage 4 comprises globally distributed and geographically restricted sublineages. Nat Genet.

[3] Freschi L, Vargas R, Husain A, Kamal SMM, Skrahina A, Tahseen S (2021). Population structure, biogeography and transmissibility of mycobacterium tuberculosis. Nat Commun..

[4] Gagneux S, DeRiemer K, Van T, Kato-Maeda M, De Jong BC, Narayanan S (2006). Variable host-pathogen compatibility in mycobacterium tuberculosis. Proc Natl Acad Sci U S A.

[5] Kaushal D, Mehra S, Didier PJ, Lackner AA. The non-human primate model of tuberculosis. J Med Primatol. 2012;41(3):191–201. https://onlinelibrary.wiley.com/doi/10.1111/j.1600-0684.2012.00536.x 10.1111/j.1600-0684.2012.00536.x PMC3961469 22429048

[6] Lombard JE, Patton EA, Gibbons-Burgener SN, Klos RF, Tans-Kersten JL, Carlson BW, et al. Human-to-Cattle Mycobacterium tuberculosis Complex Transmission in the United States. Front Vet Sci. 2021;8(July):1–11. https://www.frontiersin.org/articles/10.3389/fvets.2021.691192/full 10.3389/fvets.2021.691192 PMC8311018 34322536

[7] Naranjo V, Gortazar C, Vicente J, de la Fuente J (2008). Evidence of the role of European wild boar as a reservoir of mycobacterium tuberculosis complex. Vet Microbiol.

[8] Malone KM, Rue-Albrecht K, Magee DA, Conlon K, Schubert OT, Nalpas NC, et al. Comparative ’omics analyses differentiate mycobacterium tuberculosis and mycobacterium bovis and reveal distinct macrophage responses to infection with the human and bovine tubercle bacilli. Microb Genomics. 2018;4(3). 10.1099/mgen.0.000163 PMC5885015 29557774

[9] Wobeser G (2009). Bovine tuberculosis in Canadian wildlife: an updated history. Can Vet J La Rev Vet Can..

[10] Ayele WY, Neill SD, Zinsstag J, Weiss MG, Pavlik I (2004). Bovine tuberculosis: an old disease but a new threat to Africa. Int J Tuberc Lung Dis..

[11] VerCauteren KC, Lavelle MJ, Campa H. Persistent spillback of bovine tuberculosis from white-tailed deer to cattle in Michigan, USA: Status, Strategies, and Needs. Front Vet Sci. 2018;5(NOV):1–13. https://www.frontiersin.org/article/10.3389/fvets.2018.00301/full 10.3389/fvets.2018.00301 PMC6281989 30555834

[12] Sunstrum J, Shoyinka A, Power LE, Maxwell D, Stobiersky MG, Signs K (2019). Zoonotic Mycobacterium bovis disease in deer hunters – Michigan, 2002–2017. Morb Mortal Wkly Rep.

[13] Gormley E, Corner LAL (2018). Pathogenesis of Mycobacterium bovis Infection: The Badger model as a paradigm for understanding tuberculosis in animals. Front Vet Sci..

[14] Buddle BM, Vordermeier HM, Chambers MA, de Klerk-Lorist LM. Efficacy and safety of BCG vaccine for control of tuberculosis in domestic livestock and wildlife. Front Vet Sci. 2018 Oct 26;5(OCT):1–17. https://www.frontiersin.org/article/10.3389/fvets.2018.00259/full 10.3389/fvets.2018.00259 PMC6214331 30417002

[15] Zimpel CK, Patané JSL, Guedes ACP, de Souza RF, Silva-Pereira TT, Camargo NCS (2020). Global distribution and evolution of Mycobacterium bovis lineages. Front Microbiol.

[16] Garnier T, Eiglmeier K, Camus JC, Medina N, Mansoor H, Pryor M (2003). The complete genome sequence of Mycobacterium bovis. Proc Natl Acad Sci U S A.

[17] Rehren G, Walters S, Fontan P, Smith I, Zárraga AM (2007). Differential gene expression between Mycobacterium bovis and Mycobacterium tuberculosis. Tuberculosis..

[18] Sohaskey CD, Modesti L (2009). Differences in nitrate reduction between mycobacterium tuberculosis and mycobacterium bovis are due to differential expression of both narGHJI and narK2. FEMS Microbiol Lett.

[19] Lofthouse EK, Wheeler PR, Beste DJV, Khatri BL, Wu H, Mendum TA (2013). Systems-based approaches to probing metabolic variation within the mycobacterium tuberculosis complex. PLoS ONE.

[20] Kozlov AM, Darriba D, Flouri T, Morel B, Stamatakis A (2019). RAxML-NG: a fast, scalable and user-friendly tool for maximum likelihood phylogenetic inference. Bioinformatics.

[21] Saund K, Lapp Z, Thiede SN, Pirani A, Snitkin ES (2020). Prewas: data pre-processing for more informative bacterial gwas. Microb Genomics.

[22] Collins C, Didelot X (2018). A phylogenetic method to perform genome-wide association studies in microbes that accounts for population structure and recombination. McHardy AC, editor. PLOS Comput Biol..

[23] Collins C. How treeWAS works: Tests of Association [Internet]. GitHub repo for treeWAS. 2018. Available from: https://github.com/caitiecollins/treeWAS/wiki/1.-How-treeWAS-Works#tests-of-association

[24] Coll F, McNerney R, Guerra-Assunção JA, Glynn JR, Perdigão J, Viveiros M (2014). A robust SNP barcode for typing mycobacterium tuberculosis complex strains. Nat Commun.

[25] Lipworth S, Jajou R, De Neeling A, Bradley P, Van Der Hoek W, Maphalala G (2019). SNP-IT tool for identifying subspecies and associated lineages of mycobacterium tuberculosis complex. Emerg Infect Dis.

[26] Glickman MS, Jacobs WR (2001). Microbial pathogenesis of mycobacterium tuberculosis: Dawn of a discipline. Cell.

[27] Wipperman MF, Yang M, Thomas ST, Sampson NS (2013). Shrinking the fadE proteome of mycobacterium tuberculosis: insights into cholesterol metabolism through identification of an α2β2 heterotetrameric acyl coenzyme a dehydrogenase family. J Bacteriol.

[28] Fieweger, Wilburn, Van der Ven. Comparing the Metabolic Capabilities of Bacteria in the Mycobacterium tuberculosis Complex. Microorganisms. 2019;7(6):177. https://www.mdpi.com/2076-2607/7/6/177 10.3390/microorganisms7060177 PMC6617402 31216777

[29] Griffin JE, Gawronski JD, DeJesus MA, Ioerger TR, Akerley BJ, Sassetti CM (2011). High-resolution phenotypic profiling defines genes essential for mycobacterial growth and cholesterol catabolism. PLoS Pathog.

[30] Pandey AK, Sassetti CM (2008). Mycobacterial persistence requires the utilization of host cholesterol. Proc Natl Acad Sci U S A.

[31] Malm S, Linguissi LSG, Tekwu EM, Vouvoungui JC, Kohl TA, Beckert P (2017). New mycobacterium tuberculosis complex sublineage, Brazzaville. Congo Emerg Infect Dis.

[32] Marri PR, Bannantine JP, Golding GB (2006). Comparative genomics of metabolic pathways in mycobacterium species: gene duplication, gene decay and lateral gene transfer. FEMS Microbiol Rev.

[33] Ehebauer MT, Zimmermann M, Jakobi AJ, Noens EE, Laubitz D, Cichocki B (2015). Characterization of the Mycobacterial Acyl-CoA Carboxylase Holo Complexes Reveals Their Functional Expansion into Amino Acid Catabolism. Schnappinger D, editor. PLOS Pathog..

[34] Schwenk S, Moores A, Nobeli I, McHugh TD, Arnvig KB (2018). Cell-wall synthesis and ribosome maturation are co-regulated by an RNA switch in mycobacterium tuberculosis. Nucleic Acids Res.

[35] Ford CB, Lin PL, Chase MR, Shah RR, Iartchouk O, Galagan J (2011). Use of whole genome sequencing to estimate the mutation rate of mycobacterium tuberculosis during latent infection. Nat Genet.

[36] Moopanar K, Mvubu NE. Lineage-specific differences in lipid metabolism and its impact on clinical strains of Mycobacterium tuberculosis. Microb Pathog. 2020;146(April):104250. https://linkinghub.elsevier.com/retrieve/pii/S0882401020305374 10.1016/j.micpath.2020.104250 32407863

[37] Gonzalo-Asensio J, Malaga W, Pawlik A, Astarie-Dequeker C, Passemar C, Moreau F (2014). Evolutionary history of tuberculosis shaped by conserved mutations in the PhoPR virulence regulator. Proc Natl Acad Sci U S A.

[38] Muñoz S, Rivas-Santiago B, Enciso JA. Mycobacterium tuberculosis Entry into Mast Cells Through Cholesterol-rich Membrane Microdomains. Scand J Immunol [Internet]. 2009 Sep;70(3):256–63. https://onlinelibrary.wiley.com/doi/https://onlinelibrary.wiley.com/doi/10.1111/j.1365-3083.2009.02295.x 10.1111/j.1365-3083.2009.02295.x 19703015

[39] Kim MJ, Wainwright HC, Locketz M, Bekker LG, Walther GB, Dittrich C (2010). Caseation of human tuberculosis granulomas correlates with elevated host lipid metabolism. EMBO Mol Med.

[40] Moopanar K, Mvubu NE. Lineage-specific differences in lipid metabolism and its impact on clinical strains of Mycobacterium tuberculosis. Microb Pathog. 2020;146(April). 10.1016/j.micpath.2020.104250 32407863

[41] Guerrini V, Prideaux B, Blanc L, Bruiners N, Arrigucci R, Singh S (2018). Storage lipid studies in tuberculosis reveal that foam cell biogenesis is disease-specific. PLoS Pathog.

[42] Fernandez ML, Volek JS (2006). Guinea pigs: a suitable animal model to study lipoprotein metabolism, atherosclerosis and inflammation. Nutr Metab.

[43] Orme IM, Ordway DJ. Mouse and Guinea Pig Models of Tuberculosis. In: Tuberculosis and the Tubercle Bacillus [Internet]. Washington, DC: ASM Press; 2017. p. 143–62. Available from: http://doi.wiley.com/10.1128/9781555819569.ch7

[44] Cooper AM (2015). Mouse model of tuberculosis. Cold Spring Harb Perspect Med.

[45] Oppi S, Lüscher TF, Stein S (2019). Mouse models for atherosclerosis research—Which is my line?. Front Cardiovasc Med.

[46] Gordon SM, Li H, Zhu X, Shah AS, Lu LJ, Davidson WS. A Comparison of the Mouse and Human Lipoproteome: Suitability of the Mouse Model for Studies of Human Lipoproteins. J Proteome Res. 2015;14(6):2686–95. https://pubs.acs.org/doi/10.1021/acs.jproteome.5b00213 10.1021/acs.jproteome.5b00213 PMC4712022 25894274

[47] Duran MJ, Kannampuzha-Francis J, Nydam D, Behling-Kelly E (2021). Characterization of particle size distribution of plasma lipoproteins in dairy cattle using high-resolution polyacrylamide electrophoresis. Front Anim Sci.

[48] Inoue M, Niki M, Ozeki Y, Nagi S, Chadeka EA, Yamaguchi T (2018). High-density lipoprotein suppresses tumor necrosis factor alpha production by mycobacteria-infected human macrophages. Sci Rep..

[49] Dong H, Lv Y, Sreevatsan S, Zhao D, Zhou X (2017). Differences in pathogenicity of three animal isolates of mycobacterium species in a mouse model. PLoS ONE.

[50] Medina E, Ryan L, LaCourse R, North RJ (2006). Superior virulence of Mycobacterium bovis over mycobacterium tuberculosis (Mtb) for Mtb-resistant and Mtb-susceptible mice is manifest as an ability to cause extrapulmonary disease. Tuberculosis.

[51] Gatfield J, Pieters J (2000). Essential role for cholesterol in entry of mycobacteria into macrophages. Science (80-)..

[52] Dong Y, Feng Y, Ou X, Liu C, Fan W, Zhao Y, et al. Genomic analysis of diversity, biogeography, and drug resistance in Mycobacterium bovis. Transbound Emerg Dis. 2022;69(5):e2769–78. Available from: https://onlinelibrary.wiley.com/doi/10.1111/tbed.14628 10.1111/tbed.14628 35695307

[53] Ewels P. SRA-Explorer [Internet]. Available from: https://sra-explorer.info/

[54] Foster I (2011). Globus online: accelerating and democratizing science through cloud-based services. IEEE Internet Comput..

[55] Allen B, Bresnahan J, Childers L, Foster I, Kandaswamy G, Kettimuthu R, et al. Software as a service for data scientists. Commun ACM. 2012;55(2):81–8. https://dl.acm.org/doi/10.1145/2076450.2076468

[56] Seemann T. snippy: fast bacterial variant calling from NGS reads [Internet]. 2015. Available from: https://github.com/tseemann/snippy

[57] Wood DE, Lu J, Langmead B. Improved metagenomic analysis with Kraken 2. bioRxiv. 2019;1–13. 10.1186/s13059-019-1891-0 PMC6883579 31779668

[58] Darriba D, Posada D, Kozlov AM, Stamatakis A, Morel B, Flouri T (2020). ModelTest-NG: a new and scalable tool for the selection of DNA and protein evolutionary models. Mol Biol Evol..

[59] Allaire J. RStudio: integrated development for R [Internet]. RStudio Team. Boston, MA; 2012. Available from: www.rstudio.com

[60] RDC T. A Language and Environment for Statistical Computing. Vienna: R Foundation for Statistical Computing. 2010. Available from: https://www.r-project.org/

[61] Knaus BJ, Grünwald NJ. vcfr: a package to manipulate and visualize variant call format data in R. Mol Ecol Resour. 2017;17(1):44–53. https://onlinelibrary.wiley.com/doi/10.1111/1755-0998.12549 10.1111/1755-0998.12549 27401132

[62] Anaconda. Anaconda Software Distribution. [Internet]. Computer software. 2016. p. Vers. 2–2.4.0. Available from: https://continuum.io/

[63] GCC Team. GCC, the GNU Compiler Collection [Internet]. 2013. Available from: http://gcc.gnu.org/

[64] Gabriel E, Fagg GE, Bosilca G, Angskun T, Dongarra JJ, Squyres JM, et al. Open MPI: Goals, Concept, and Design of a Next Generation MPI Implementation. In: Lecture Notes in Computer Science (including subseries Lecture Notes in Artificial Intelligence and Lecture Notes in Bioinformatics) [Internet]. 2004. p. 97–104. Available from: http://link.springer.com/10.1007/978-3-540-30218-6_19

[65] Wickham H, Hester J, Chang W. Tools to make developing R packages easier - Package “devtools” [Internet]. 2021. Available from: https://devtools.r-lib.org/, https://github.com/r-lib/devtools

[66] Saund K, Lapp Z, Thiede SN, Pirani A, Snitkin ES. Prewas: Data pre-processing for more informative bacterial gwas [Internet]. Vol. 6, Microbial Genomics. GitHub; 2020. p. 1–8. Available from: https://github.com/Snitkin-Lab-Umich/prewas 10.1099/mgen.0.000368 PMC7371116 32310745

[67] Collins C, Didelot X. treeWAS: A phylogenetic tree-based approach to genome-wide association studies in microbes [Internet]. GitHub; 2022. Available from: https://github.com/caitiecollins/treeWAS

[68] Cingolani P, Platts A, Wang LL, Coon M, Nguyen T, Wang L, et al. A program for annotating and predicting the effects of single nucleotide polymorphisms, SnpEff. Fly (Austin) [Internet]. 2012 Apr 27;6(2):80–92. Available from: http://www.tandfonline.com/doi/abs/10.4161/fly.19695 10.4161/fly.19695 PMC3679285 22728672

