# Supplementary material for: Global-scale GWAS associates a subset of SNPs with animal-adapted variants in M. tuberculosis complex
Source: BMC Med Genomics. 2023 Oct 24;16:260. doi: 10.1186/s12920-023-01695-5 (PMC10598944; doi:10.1186/s12920-023-01695-5)
Supplement: Supplementary file 2 — Additional file 2. [file 12920_2023_1695_MOESM2_ESM.pdf]

Supp 2. GWAS Tree – NWK file

((ERR6139637:0.00000100,(ERR6149933:0.00000100,(ERR6140773:0.00010800,(ERR6149425:0
.00000100,(ERR6143852:0.00000100,(ERR6140351:0.00000100,(ERR6149427:0.00000100,(ERR
6149469:0.00000100,(((((SRR14296393:0.00108100,SRR15296958:0.00064800)0.0333:0.00000
100,((((((((SRR1791766:0.00021600,((SRR1791974:0.00000100,SRR1791975:0.00000100)0.3967
:0.00064800,SRR1791998:0.00010800)0.4483:0.00010800)0.5344:0.00064800,SRR13268339:0.
00064800)0.4058:0.00000100,(SRR1792066:0.00021600,SRR1791763:0.00010800)0.3167:0.00
021600)0.2750:0.00000100,((((((((((SRR1791829:0.00000100,SRR1791762:0.00010800)0.3967:
0.00021600,SRR1791809:0.00054000)0.3983:0.00010800,(((((((((((((((((((((((SRR1792099:0.000
00100,SRR1792113:0.00000100)0.3533:0.00043200,SRR1791725:0.00000100)0.3783:0.000108
00,((SRR1791781:0.00010800,((SRR1791729:0.00000100,(SRR1792031:0.00000100,SRR179203
0:0.00000100)0.3467:0.00010800)0.3683:0.00000100,SRR1791724:0.00000100)0.5178:0.0006
4800)0.3908:0.00000100,(((((((((((((((((((((((((SRR1791740:0.00000100,SRR1791771:0.00000100
)0.2967:0.00010800,((((SRR1791782:0.00000100,SRR1791783:0.00000100)0.1200:0.00000100,
SRR1791743:0.00000100)0.2133:0.00000100,SRR1791787:0.00000100)0.3044:0.00000100,SRR
1791786:0.00000100)0.3833:0.00010800)0.2833:0.00000100,((((((((SRR1791836:0.00000100,S
RR1791850:0.00000100)0.0533:0.00000100,SRR1791879:0.00010800)0.0667:0.00000100,SRR1
791837:0.00000100)0.1433:0.00000100,SRR1791734:0.00000100)0.2117:0.00000100,SRR1791
833:0.00000100)0.2813:0.00000100,SRR1791853:0.00000100)0.3433:0.00000100,SRR1791851:
0.00000100)0.4114:0.00000100,SRR1791835:0.00000100)0.4679:0.00021600)0.2867:0.000001
00,(((((((SRR1791886:0.00000100,((SRR1792333:0.00000100,SRR1792313:0.00000100)0.2767:0
.00010800,SRR1791887:0.00000100)0.3200:0.00000100)0.4067:0.00010800,SRR1792446:0.000
00100)0.3800:0.00000100,SRR12554471:0.00000100)0.4080:0.00000100,SRR1792450:0.00043
200)0.4606:0.00010800,SRR1791877:0.00000100)0.4738:0.00010800,SRR1792074:0.00010800
)0.4521:0.00000100,SRR1791796:0.00010800)0.4641:0.00010800)0.2580:0.00000100,SRR1791
892:0.00043200)0.2665:0.00000100,SRR1792158:0.00032400)0.2699:0.00000100,(((SRR17917
67:0.00021600,SRR1792422:0.00032400)0.1167:0.00000100,SRR1792039:0.00010800)0.2550:0
.00000100,SRR5657418:0.00010800)0.3378:0.00010800)0.3068:0.00000100,((SRR1791966:0.0
0000100,SRR1791943:0.00000100)0.2200:0.00000100,SRR1791962:0.00000100)0.3917:0.0002
1600)0.3254:0.00000100,SRR1791806:0.00032400)0.3331:0.00000100,SRR1791704:0.0000010
0)0.3420:0.00000100,(((SRR1791750:0.00000100,SRR1791748:0.00000100)0.1900:0.00000100,
SRR1791720:0.00000100)0.3467:0.00000100,SRR1791749:0.00000100)0.5022:0.00032400)0.3
663:0.00000100,SRR1791939:0.00021600)0.3702:0.00010800,SRR1792349:0.00021600)0.3618:
0.00000100,((((((((((((((((((((((((((SRR1792365:0.00000100,SRR1791856:0.00000100)0.0267:0.00
000100,SRR1792359:0.00000100)0.0400:0.00000100,SRR1792360:0.00000100)0.0456:0.00000
100,SRR1792362:0.00000100)0.0533:0.00000100,SRR1792366:0.00000100)0.0773:0.00000100,
SRR1791840:0.00000100)0.0967:0.00000100,SRR1791813:0.00000100)0.1052:0.00000100,SRR
1792348:0.00000100)0.1271:0.00000100,SRR1792417:0.00000100)0.1485:0.00000100,SRR179
2368:0.00000100)0.1687:0.00000100,SRR1791808:0.00000100)0.1945:0.00000100,SRR179237
1:0.00000100)0.2189:0.00000100,SRR1792364:0.00000100)0.2477:0.00000100,SRR1791814:0.
00000100)0.2774:0.00000100,SRR1792416:0.00000100)0.3044:0.00000100,SRR1791794:0.000
00100)0.3354:0.00000100,SRR1791849:0.00000100)0.3665:0.00000100,SRR1792361:0.000001
00)0.3948:0.00000100,SRR1792414:0.00000100)0.4221:0.00000100,SRR1792367:0.00000100)

0.4475:0.00000100,SRR1792413:0.00000100)0.4679:0.00000100,SRR1792415:0.00000100)0.49
20:0.00010800,SRR1791758:0.00000100)0.4983:0.00010800,SRR1791898:0.00010800)0.4842:0
.00000100,SRR1791897:0.00000100)0.4779:0.00000100,(SRR1791904:0.00000100,SRR1791895
:0.00010800)0.2867:0.00010800)0.4844:0.00000100,SRR1791896:0.00000100)0.4971:0.00021
600)0.2838:0.00000100,(((SRR1791709:0.00000100,SRR1791732:0.00000100)0.1367:0.000001
00,SRR1791916:0.00010800)0.2083:0.00000100,SRR1791744:0.00000100)0.3100:0.00010800)
0.2719:0.00000100,((((((((SRR1792016:0.00000100,SRR1791890:0.00000100)0.1700:0.0000010
0,SRR1791811:0.00010800)0.3650:0.00010800,SRR1792035:0.00010800)0.3056:0.00000100,SR
R1792345:0.00032400)0.3025:0.00000100,SRR1791917:0.00021600)0.3207:0.00000100,SRR17
91893:0.00010800)0.3550:0.00000100,SRR1791838:0.00000100)0.4071:0.00000100,SRR17921
69:0.00021600)0.4604:0.00021600)0.2563:0.00000100,(((((((((((((((SRR1791846:0.00000100,SR
R1792353:0.00000100)0.0700:0.00000100,SRR1791742:0.00000100)0.1383:0.00000100,SRR17
91844:0.00000100)0.1878:0.00000100,SRR1792026:0.00000100)0.2700:0.00000100,SRR17918
62:0.00000100)0.3247:0.00000100,SRR1791845:0.00000100)0.3794:0.00010800,(((((((((SRR179
1920:0.00000100,SRR1791932:0.00000100)0.0500:0.00000100,SRR1791918:0.00000100)0.088
3:0.00000100,SRR1791919:0.00000100)0.1111:0.00000100,SRR1791934:0.00000100)0.1633:0.
00000100,SRR1791905:0.00000100)0.2060:0.00000100,SRR1791922:0.00000100)0.2528:0.000
00100,SRR1791914:0.00000100)0.3081:0.00000100,SRR1791908:0.00000100)0.3438:0.000001
00,SRR1791921:0.00000100)0.3826:0.00010800)0.3075:0.00000100,(SRR1792384:0.00010800,
SRR1792354:0.00000100)0.2433:0.00010800)0.3156:0.00000100,SRR1791843:0.00010800)0.3
256:0.00000100,SRR1791755:0.00043200)0.3337:0.00000100,SRR1792319:0.00000100)0.3440:
0.00000100,SRR1791735:0.00010800)0.3567:0.00000100,SRR1792322:0.00000100)0.3638:0.00
000100,SRR1791831:0.00021600)0.3738:0.00000100,SRR1792321:0.00000100)0.3852:0.00010
800)0.2726:0.00000100,SRR1792088:0.00000100)0.2759:0.00000100,(SRR1791769:0.0000010
0,(SRR1791989:0.00000100,SRR1791915:0.00000100)0.3033:0.00010800)0.3733:0.00010800)0
.2849:0.00000100,((SRR1792489:0.00021600,SRR1791754:0.00075600)0.2533:0.00010800,SRR
1791924:0.00054000)0.1317:0.00000100)0.2966:0.00000100,((((((((((((((((SRR1792082:0.00000
100,SRR1792075:0.00000100)0.0600:0.00000100,SRR1791957:0.00000100)0.0617:0.00000100,
SRR1791944:0.00000100)0.0778:0.00000100,SRR1792087:0.00000100)0.0817:0.00000100,SRR
1792079:0.00000100)0.1053:0.00000100,SRR1792080:0.00000100)0.1094:0.00000100,SRR179
2085:0.00000100)0.1424:0.00000100,SRR1792081:0.00000100)0.1563:0.00000100,SRR179210
6:0.00000100)0.1819:0.00000100,SRR1792083:0.00000100)0.2170:0.00000100,SRR1791906:0.
00010800)0.2361:0.00000100,SRR1792084:0.00000100)0.2600:0.00000100,SRR1792091:0.000
00100)0.2882:0.00000100,SRR1792086:0.00000100)0.3124:0.00000100,SRR1792093:0.000001
00)0.3349:0.00000100,SRR1792092:0.00000100)0.3554:0.00010800)0.3596:0.00000100,(SRR1
792040:0.00010800,SRR1791706:0.00000100)0.2533:0.00010800)0.3683:0.00000100,SRR1791
903:0.00043200)0.3720:0.00000100,SRR1791973:0.00021600)0.3755:0.00000100,SRR1791702:
0.00021600)0.3794:0.00000100,SRR1791900:0.00032400)0.3828:0.00000100)0.4018:0.000001
00)0.4131:0.00000100,SRR1792309:0.00032400)0.4169:0.00000100,((SRR1791991:0.00000100
,SRR1791992:0.00000100)0.3100:0.00000100,SRR1791990:0.00000100)0.5050:0.00043200)0.4
268:0.00000100,(SRR1791778:0.00064800,SRR1792357:0.00021600)0.0067:0.00000100)0.433
2:0.00000100,SRR1791798:0.00000100)0.4366:0.00000100,SRR1792150:0.00000100)0.4399:0.
00000100,SRR1792288:0.00010800)0.4436:0.00000100,SRR1792059:0.00000100)0.4469:0.000
00100,SRR1792151:0.00000100)0.4504:0.00000100,SRR1792013:0.00021600)0.4533:0.000001

00,SRR1791703:0.00010800)0.4564:0.00000100,(SRR1792506:0.00000100,SRR1792504:0.0000
0100)0.4300:0.00032400)0.4628:0.00000100,SRR1791695:0.00010800)0.4654:0.00000100,SRR
1791761:0.00032400)0.4683:0.00000100,(SRR1791993:0.00000100,SRR1792033:0.00000100)0
.3600:0.00021600)0.4742:0.00000100,SRR1792250:0.00010800)0.4779:0.00000100,SRR179229
6:0.00000100)0.4807:0.00000100,(SRR1791953:0.00032400,SRR1791790:0.00075600)0.0267:0
.00000100)0.4863:0.00000100,SRR1792029:0.00000100)0.4890:0.00000100,SRR14296143:0.00
010800)0.4920:0.00000100,SRR1792369:0.00010800)0.4950:0.00000100,SRR1791791:0.00000
100)0.4976:0.00032400)0.4896:0.00000100,((((((((SRR1792142:0.00000100,((((((((((((((((((((((((((
((((((((((((((((((((((((((SRR1791982:0.00000100,(((((((((SRR1792177:0.00000100,SRR1792180:0.00
000100)0.0700:0.00000100,SRR1792181:0.00000100)0.1017:0.00000100,SRR1792183:0.00000
100)0.1322:0.00000100,SRR1792178:0.00000100)0.1475:0.00000100,SRR1792182:0.00000100
)0.1920:0.00000100,SRR1792166:0.00000100)0.2422:0.00000100,SRR1792168:0.00000100)0.2
862:0.00000100,SRR1792167:0.00000100)0.3158:0.00000100,SRR1792179:0.00000100)0.3552:
0.00010800)0.3230:0.00000100,SRR1792191:0.00000100)0.2967:0.00000100,SRR1792115:0.00
000100)0.2742:0.00000100,SRR1792094:0.00000100)0.2562:0.00000100,SRR1792102:0.00000
100)0.2410:0.00000100,SRR1792048:0.00000100)0.2287:0.00000100,SRR1791947:0.00000100
)0.2165:0.00000100,SRR1791935:0.00000100)0.2059:0.00000100,SRR1792097:0.00000100)0.1
963:0.00000100,SRR1791980:0.00000100)0.1874:0.00000100,SRR1791985:0.00000100)0.1790:
0.00000100,SRR1792010:0.00000100)0.1746:0.00000100,SRR1792049:0.00000100)0.1686:0.00
000100,SRR1791997:0.00000100)0.1649:0.00000100,SRR1792090:0.00000100)0.1599:0.00000
100,SRR1792044:0.00000100)0.1569:0.00000100,SRR1792119:0.00000100)0.1538:0.00000100,
SRR1792145:0.00000100)0.1509:0.00000100,SRR1792105:0.00000100)0.1476:0.00000100,SRR
1792038:0.00000100)0.1475:0.00000100,SRR1792067:0.00000100)0.1471:0.00000100,SRR179
2006:0.00000100)0.1474:0.00000100,SRR1792095:0.00000100)0.1479:0.00000100,SRR179212
4:0.00000100)0.1495:0.00000100,SRR1791984:0.00000100)0.1519:0.00000100,SRR1792043:0.
00000100)0.1549:0.00000100,((SRR1792429:0.00000100,(((SRR1792339:0.00000100,SRR17923
41:0.00000100)0.1700:0.00000100,SRR1792338:0.00000100)0.3067:0.00000100,SRR1792340:0
.00000100)0.4667:0.00021600)0.4250:0.00000100,SRR1792334:0.00000100)0.4713:0.0002160
0)0.1901:0.00000100,SRR1792143:0.00000100)0.1994:0.00000100,SRR1792231:0.00000100)0.
2108:0.00000100,SRR1792007:0.00000100)0.2214:0.00000100,SRR1792076:0.00000100)0.231
0:0.00000100,SRR1792062:0.00000100)0.2443:0.00000100,SRR1792061:0.00000100)0.2571:0.
00000100,SRR1791977:0.00043200)0.2681:0.00000100,SRR1792114:0.00000100)0.2800:0.000
00100,SRR1792103:0.00000100)0.2918:0.00000100,SRR1792144:0.00000100)0.3020:0.000001
00,SRR1791994:0.00000100)0.3115:0.00000100,SRR1792192:0.00000100)0.3214:0.00000100,S
RR1791979:0.00000100)0.3307:0.00000100,SRR1792116:0.00000100)0.3415:0.00000100,SRR1
792123:0.00000100)0.3514:0.00000100,SRR1792244:0.00000100)0.3609:0.00000100,SRR1792
009:0.00000100)0.3701:0.00000100,SRR1791983:0.00000100)0.3783:0.00000100,SRR1791995:
0.00000100)0.3862:0.00000100,SRR1792199:0.00000100)0.3934:0.00000100,SRR1791967:0.00
000100)0.4020:0.00000100,SRR1791996:0.00000100)0.4088:0.00000100,SRR1792100:0.00000
100)0.4160:0.00000100,SRR1792104:0.00000100)0.4232:0.00000100,SRR1792149:0.00010800
)0.4286:0.00000100)0.4350:0.00000100,SRR1792121:0.00000100)0.4421:0.00000100,SRR1792
070:0.00000100)0.4502:0.00000100,SRR1791981:0.00000100)0.4582:0.00010800,SRR1791861:
0.00010800)0.4564:0.00000100,(SRR1791728:0.00000100,SRR1791773:0.00054000)0.3933:0.0
0021600)0.4560:0.00000100,SRR1791822:0.00032400)0.4573:0.00000100,SRR1792281:0.0003

2400)0.4590:0.00010800)0.3782:0.00000100,(((((((SRR11462879:0.00000100,SRR11462871:0.0
0000100)0.0700:0.00000100,SRR11462903:0.00000100)0.1567:0.00000100,SRR11462913:0.00
021600)0.1944:0.00000100,SRR11462927:0.00000100)0.2783:0.00000100,SRR11462924:0.000
00100)0.3407:0.00000100,SRR11462914:0.00000100)0.4017:0.00000100,SRR11462920:0.0000
0100)0.4414:0.00021600)0.3715:0.00000100,(((((((((SRR5657518:0.00000100,((SRR14296421:0
.00000100,SRR14296288:0.00000100)0.1633:0.00000100,SRR14296191:0.00000100)0.3183:0.0
0000100)0.4378:0.00021600,((((((((((SRR1792196:0.00000100,SRR1791765:0.00000100)0.0567:
0.00000100,SRR1791928:0.00010800)0.0483:0.00000100,SRR1792194:0.00000100)0.0800:0.00
000100,SRR1792305:0.00010800)0.0950:0.00000100,SRR1791949:0.00010800)0.1113:0.00000
100,SRR1792036:0.00043200)0.1028:0.00000100,SRR1792447:0.00010800)0.1114:0.00000100,
SRR1792198:0.00000100)0.1358:0.00000100,SRR1792195:0.00000100)0.1596:0.00000100,SRR
1791907:0.00032400)0.1593:0.00000100)0.1840:0.00000100,SRR1791739:0.00010800)0.1922:
0.00000100,((((SRR1792283:0.00000100,SRR1791745:0.00010800)0.1300:0.00000100,SRR1791
847:0.00010800)0.2033:0.00000100,SRR1791956:0.00000100)0.3344:0.00000100,SRR1792328:
0.00000100)0.4358:0.00021600)0.2288:0.00000100,((((((((((((((((SRR12554457:0.00000100,SRR
12554460:0.00000100)0.0367:0.00000100,((SRR12554468:0.00000100,SRR12554454:0.000001
00)0.1733:0.00000100,SRR12554469:0.00000100)0.3300:0.00010800)0.2092:0.00000100,SRR1
2554458:0.00000100)0.1900:0.00000100,SRR12554467:0.00000100)0.1822:0.00000100,SRR12
554459:0.00000100)0.1929:0.00000100,SRR12554463:0.00000100)0.2150:0.00000100,SRR125
54464:0.00000100)0.2370:0.00000100,SRR12554456:0.00000100)0.2680:0.00000100,SRR1255
4461:0.00000100)0.3058:0.00000100,SRR12554466:0.00000100)0.3397:0.00000100,SRR12554
470:0.00000100)0.3836:0.00000100,SRR12554455:0.00000100)0.4152:0.00000100,SRR125544
62:0.00000100)0.4493:0.00000100,SRR12554465:0.00010800)0.4719:0.00010800,(SRR179214
1:0.00000100,SRR1792132:0.00000100)0.3900:0.00043200)0.4700:0.00000100,SRR1791945:0.
00000100)0.4881:0.00021600)0.4250:0.00000100,(SRR1791952:0.00032400,SRR1791927:0.00
000100)0.2733:0.00010800)0.4420:0.00000100,SRR1792307:0.00010800)0.4525:0.00000100,S
RR5657483:0.00000100)0.4625:0.00010800,((SRR1791788:0.00021600,(SRR1792020:0.000216
00,SRR1792072:0.00000100)0.3433:0.00010800)0.3167:0.00000100,SRR16278265:0.00043200
)0.3989:0.00010800)0.4576:0.00010800)0.3835:0.00000100,((((((((SRR11462901:0.00000100,((
((((((((SRR11462921:0.00000100,SRR11462874:0.00010800)0.0400:0.00000100,SRR11462904:0
.00000100)0.0750:0.00000100,SRR11462935:0.00010800)0.0933:0.00000100,SRR11462923:0.0
0010800)0.1267:0.00000100,SRR11462870:0.00010800)0.1660:0.00000100,SRR11462865:0.00
000100)0.2100:0.00000100,SRR11462931:0.00010800)0.2419:0.00000100,SRR11462912:0.000
10800)0.2908:0.00000100,SRR11462891:0.00000100)0.3504:0.00000100,SRR11462869:0.0000
0100)0.3883:0.00000100)0.4327:0.00010800,(((((SRR11462926:0.00000100,(SRR11462918:0.0
0000100,SRR11462936:0.00000100)0.3067:0.00010800)0.2117:0.00000100,(((SRR11462940:0.
00000100,SRR11462872:0.00000100)0.1333:0.00000100,SRR11462883:0.00000100)0.2983:0.0
0000100,SRR11462919:0.00010800)0.4011:0.00010800)0.3456:0.00000100,SRR11462864:0.00
021600)0.3629:0.00000100,SRR11462902:0.00021600)0.3854:0.00000100,SRR11462892:0.000
00100)0.4252:0.00010800)0.4957:0.00021600,SRR11535173:0.00021600)0.4795:0.00000100,((
SRR11535174:0.00000100,SRR11535176:0.00021600)0.1867:0.00000100,SRR11535175:0.0000
0100)0.4650:0.00032400)0.4513:0.00000100,((SRR14049337:0.00000100,SRR14049338:0.0000
0100)0.2333:0.00000100,SRR14049339:0.00000100)0.4900:0.00043200)0.4639:0.00000100,SR
R14049343:0.00054000)0.4749:0.00000100,SRR11535171:0.00032400)0.4869:0.00000100,SRR

11535177:0.00032400)0.4975:0.00021600)0.4214:0.00000100,(((((SRR1792358:0.00000100,(SR
R5657394:0.00021600,SRR1791864:0.00032400)0.2367:0.00000100)0.4067:0.00010800,SRR17
91752:0.00010800)0.4189:0.00010800,SRR1791785:0.00054000)0.3717:0.00000100,((SRR1791
885:0.00000100,SRR1791958:0.00000100)0.2067:0.00000100,SRR1791881:0.00000100)0.4667:
0.00021600)0.4562:0.00000100,SRR1791826:0.00032400)0.4983:0.00032400)0.4347:0.000001
00,((((((SRR5216979:0.00000100,(SRR5216954:0.00000100,SRR5216910:0.00010800)0.1967:0.
00000100)0.3467:0.00010800,SRR5216937:0.00000100)0.2767:0.00000100,SRR5216711:0.000
21600)0.2567:0.00000100,(SRR5216850:0.00000100,(SRR5216841:0.00000100,SRR5216978:0.
00010800)0.3600:0.00021600)0.3883:0.00010800)0.3148:0.00000100,((SRR5216855:0.000108
00,SRR5216809:0.00000100)0.3167:0.00010800,SRR5216758:0.00000100)0.3917:0.00010800)
0.4273:0.00000100,SRR5216958:0.00000100)0.4652:0.00000100,SRR5216719:0.00010800)0.49
17:0.00032400)0.4508:0.00000100,((((((SRR11462873:0.00000100,SRR11462906:0.00010800)0
.2267:0.00010800,(SRR11462930:0.00010800,SRR11462939:0.00000100)0.2833:0.00010800)0.
3456:0.00000100,SRR11462877:0.00032400)0.4542:0.00010800,SRR11462887:0.00021600)0.4
467:0.00000100,SRR11462884:0.00000100)0.5089:0.00054000,(SRR11462899:0.00010800,SRR
11462894:0.00000100)0.3900:0.00021600)0.4942:0.00021600)0.4624:0.00000100,(((ERR44509
43:0.00000100,(((((((ERR4450940:0.00000100,ERR4450942:0.00000100)0.0600:0.00000100,ER
R4450953:0.00000100)0.1350:0.00000100,ERR4450944:0.00000100)0.1844:0.00000100,ERR44
50955:0.00000100)0.2342:0.00000100,ERR4450954:0.00000100)0.2827:0.00000100,ERR44509
49:0.00000100)0.3400:0.00000100,ERR4450948:0.00000100)0.4086:0.00000100)0.4588:0.000
00100,ERR4450941:0.00000100)0.5063:0.00032400,SRR14049340:0.00032400)0.4897:0.00010
800)0.4761:0.00000100)0.4849:0.00000100,SRR1792172:0.00010800)0.4863:0.00000100,((SRR
14049336:0.00010800,SRR14049341:0.00021600)0.1400:0.00000100,SRR14049335:0.0003240
0)0.3150:0.00010800)0.4901:0.00000100,SRR11462878:0.00000100)0.4915:0.00000100,(SRR1
791707:0.00086400,SRR14049334:0.00032400)0.0033:0.00000100)0.4941:0.00000100)0.4964:
0.00000100,SRR11462917:0.00000100)0.4975:0.00000100,(SRR1792051:0.00000100,SRR17920
60:0.00000100)0.4233:0.00021600)0.4998:0.00032400,((
((
((
((
((
((
((
((
(((SRR1791770:0.00010700,SRR1791777:0.00054100)0.350
0:0.00140600,ERR4188374:0.00010800)0.1817:0.00000100,((
((
((
((SRR5216967:0.000001
00,((SRR5216754:0.00000100,SRR5216731:0.00000100)0.0533:0.00000100,SRR5216814:0.000
00100)0.1000:0.00000100)0.1533:0.00000100,SRR5216947:0.00000100)0.1850:0.00000100,SR
R5216949:0.00000100)0.2353:0.00000100,SRR5216934:0.00000100)0.2778:0.00000100,SRR52
16781:0.00000100)0.3133:0.00000100,SRR5216691:0.00021600)0.3329:0.00000100,(SRR5216
914:0.00000100,SRR5216797:0.00000100)0.3833:0.00032400)0.3870:0.00000100,SRR5216883:
0.00000100)0.4242:0.00000100,SRR5216768:0.00010800)0.4642:0.00000100,SRR5216929:0.00

010800)0.4990:0.00010700,(SRR5216936:0.00000100,SRR5216961:0.00000100)0.3633:0.0004
3300)0.5098:0.00162300,(((((ERR4188404:0.00000100,ERR3468537:0.00000100)0.1200:0.0000
0100,ERR3468663:0.00000100)0.2000:0.00000100,ERR4188083:0.00000100)0.2911:0.0000010
0,ERR4187970:0.00000100)0.3608:0.00000100,ERR3468648:0.00000100)0.4340:0.00021600)0.
3683:0.00000100,((
(((SRR8
073657:0.00000100,SRR8073646:0.00000100)0.0000:0.00000100,SRR1792401:0.00000100)0.0
017:0.00000100,SRR5817713:0.00000100)0.0033:0.00000100,SRR1792037:0.00000100)0.0067:
0.00000100,SRR1792471:0.00000100)0.0080:0.00000100,SRR1792461:0.00000100)0.0089:0.00
000100,SRR5817694:0.00000100)0.0105:0.00000100,SRR1791854:0.00000100)0.0129:0.00000
100,SRR1792399:0.00000100)0.0130:0.00000100,SRR1792057:0.00000100)0.0147:0.00000100,
(SRR8073672:0.00000100,SRR8073656:0.00000100)0.3600:0.00021600)0.0389:0.00000100,SR
R8073654:0.00010800)0.0372:0.00000100,SRR1792423:0.00010800)0.0362:0.00000100,SRR17
92397:0.00000100)0.0347:0.00000100,SRR8073630:0.00000100)0.0346:0.00000100,SRR17920
96:0.00000100)0.0337:0.00000100,SRR8073628:0.00000100)0.0335:0.00000100,SRR1792440:0
.00000100)0.0333:0.00000100,SRR1792436:0.00000100)0.0327:0.00000100,SRR1792439:0.000
00100)0.0322:0.00000100,SRR1792112:0.00000100)0.0315:0.00000100,SRR1792458:0.000001
00)0.0309:0.00000100,SRR1792291:0.00000100)0.0300:0.00000100,SRR1792412:0.00000100)
0.0296:0.00000100,(((((((((((((((((((((((((((((((SRR1792510:0.00000100,SRR5817670:0.00000100)0
.0167:0.00000100,SRR5817697:0.00000100)0.0167:0.00000100,SRR5817715:0.00000100)0.024
4:0.00000100,SRR1791828:0.00000100)0.0333:0.00000100,SRR8073652:0.00000100)0.0420:0.
00000100,SRR1792187:0.00000100)0.0517:0.00000100,SRR1792497:0.00000100)0.0581:0.000
00100,SRR5817690:0.00021600)0.0550:0.00000100,SRR1791929:0.00000100)0.0615:0.000001
00,SRR1791930:0.00000100)0.0690:0.00000100,SRR5817675:0.00000100)0.0767:0.00000100,S
RR5817707:0.00000100)0.0833:0.00000100,SRR5817669:0.00000100)0.0910:0.00000100,SRR5
817689:0.00000100)0.0998:0.00000100,SRR5817700:0.00000100)0.1102:0.00000100,SRR1792
069:0.00000100)0.1208:0.00000100,SRR5817717:0.00000100)0.1359:0.00000100,SRR1791940:
0.00000100)0.1515:0.00000100,SRR5817674:0.00000100)0.1653:0.00000100,SRR1791819:0.00
000100)0.1793:0.00000100,SRR1792050:0.00000100)0.1957:0.00000100,SRR8073606:0.00000
100)0.2091:0.00000100,SRR5817696:0.00000100)0.2232:0.00000100,SRR1792508:0.00000100
)0.2374:0.00000100,SRR1792058:0.00000100)0.2489:0.00000100,SRR1792509:0.00000100)0.2
578:0.00000100,SRR8073659:0.00000100)0.2691:0.00000100,SRR1791818:0.00000100)0.2786:
0.00000100,SRR5817709:0.00000100)0.2909:0.00000100,SRR1791931:0.00000100)0.3004:0.00
000100,SRR1791925:0.00000100)0.3115:0.00010800)0.1738:0.00000100,SRR1791839:0.00000
100)0.1710:0.00000100,SRR1792463:0.00000100)0.1683:0.00000100,SRR3091238:0.00000100
)0.1657:0.00000100,SRR1791874:0.00000100)0.1630:0.00000100,SRR3091235:0.00000100)0.1
606:0.00000100,SRR3091255:0.00000100)0.1581:0.00000100,SRR1792331:0.00000100)0.1558:
0.00000100,SRR1792011:0.00010800)0.1536:0.00000100,SRR5817695:0.00000100)0.1515:0.00
000100,SRR8073622:0.00000100)0.1495:0.00000100,SRR5817677:0.00000100)0.1474:0.00000
100,SRR1792390:0.00000100)0.1453:0.00000100,SRR8073605:0.00000100)0.1435:0.00000100,
SRR1792405:0.00000100)0.1417:0.00000100,SRR5817719:0.00000100)0.1399:0.00000100,SRR
1792378:0.00000100)0.1383:0.00000100,SRR3091248:0.00000100)0.1366:0.00000100,SRR179
2389:0.00000100)0.1349:0.00000100,SRR3091239:0.00000100)0.1333:0.00000100,SRR179238
2:0.00000100)0.1316:0.00000100,SRR1792262:0.00000100)0.1303:0.00000100,SRR1792466:0.

00000100)0.1290:0.00000100,SRR1791738:0.00000100)0.1278:0.00000100,SRR1792394:0.000
00100)0.1264:0.00000100,SRR8073670:0.00000100)0.1251:0.00000100,SRR3091241:0.000001
00)0.1239:0.00000100,SRR5817691:0.00000100)0.1227:0.00000100,SRR8073624:0.00000100)
0.1218:0.00000100,SRR1792392:0.00000100)0.1206:0.00000100,SRR1792469:0.00000100)0.11
97:0.00000100,SRR1792055:0.00000100)0.1187:0.00000100,SRR1792419:0.00000100)0.1178:0
.00000100,SRR1791693:0.00000100)0.1167:0.00000100,SRR1792243:0.00000100)0.1157:0.000
00100,SRR1792323:0.00000100)0.1147:0.00000100,SRR5817706:0.00000100)0.1138:0.000001
00,SRR5817671:0.00000100)0.1129:0.00000100,SRR3091251:0.00000100)0.1119:0.00000100,S
RR3091242:0.00000100)0.1113:0.00000100,(SRR8073679:0.00010800,SRR8073618:0.0001080
0)0.1900:0.00010800)0.1095:0.00000100,SRR8073666:0.00000100)0.1086:0.00000100,SRR179
1894:0.00000100)0.1081:0.00000100,SRR1792398:0.00000100)0.1074:0.00000100,SRR179185
7:0.00000100)0.1071:0.00000100,SRR3091243:0.00000100)0.1064:0.00000100,SRR1792380:0.
00000100)0.1061:0.00000100,SRR3091245:0.00000100)0.1057:0.00000100,SRR1792294:0.000
10800)0.1052:0.00000100,SRR1792483:0.00000100)0.1048:0.00000100,SRR8073610:0.000001
00)0.1040:0.00000100,SRR1792406:0.00000100)0.1036:0.00000100,SRR5817710:0.00000100)
0.1037:0.00000100,SRR5817678:0.00000100)0.1034:0.00000100,SRR5817668:0.00000100)0.10
32:0.00000100,SRR1792437:0.00000100)0.1028:0.00000100,SRR3091237:0.00000100)0.1027:0
.00000100,SRR3091247:0.00000100)0.1026:0.00000100,SRR3091258:0.00000100)0.1024:0.000
00100,SRR3091254:0.00000100)0.1026:0.00000100,SRR8073609:0.00000100)0.1027:0.000001
00,SRR8073647:0.00000100)0.1032:0.00000100,SRR5817704:0.00000100)0.1037:0.00000100,S
RR3091257:0.00000100)0.1042:0.00000100,SRR1792434:0.00000100)0.1046:0.00000100,SRR1
792460:0.00000100)0.1052:0.00000100,SRR1791716:0.00000100)0.1058:0.00000100,SRR1792
019:0.00000100)0.1064:0.00000100,SRR3091240:0.00000100)0.1070:0.00000100,SRR3091232:
0.00000100)0.1078:0.00000100,SRR8073667:0.00000100)0.1088:0.00000100,SRR1792373:0.00
000100)0.1105:0.00000100,SRR1792377:0.00000100)0.1120:0.00000100,(((SRR5817673:0.000
10800,SRR5817708:0.00000100)0.1200:0.00000100,SRR5817712:0.00000100)0.2217:0.000001
00,SRR5817702:0.00000100)0.3111:0.00010800)0.1188:0.00000100,SRR3091250:0.00000100)
0.1207:0.00000100,SRR1791719:0.00000100)0.1231:0.00000100,SRR5817687:0.00000100)0.12
47:0.00000100,SRR1792351:0.00000100)0.1271:0.00000100,SRR8073617:0.00000100)0.1298:0
.00000100,SRR1792470:0.00000100)0.1322:0.00000100,SRR1792418:0.00010800)0.1350:0.000
00100,SRR1792053:0.00000100)0.1379:0.00000100,SRR1792395:0.00000100)0.1408:0.000001
00,SRR3091246:0.00000100)0.1441:0.00000100,SRR3091244:0.00000100)0.1473:0.00000100,S
RR5817720:0.00000100)0.1500:0.00000100,SRR5817699:0.00000100)0.1532:0.00000100,SRR1
792370:0.00000100)0.1566:0.00000100,SRR1792286:0.00000100)0.1600:0.00000100,SRR1792
352:0.00000100)0.1638:0.00000100,SRR1792242:0.00000100)0.1671:0.00000100,SRR1792420:
0.00000100)0.1701:0.00000100,SRR1792459:0.00000100)0.1729:0.00000100,SRR1792186:0.00
000100)0.1757:0.00000100,SRR8073615:0.00000100)0.1794:0.00000100,SRR1792400:0.00000
100)0.1824:0.00000100,SRR1792393:0.00000100)0.1852:0.00000100,SRR1792257:0.00000100
)0.1885:0.00000100,SRR5817685:0.00000100)0.1914:0.00000100,SRR1791863:0.00000100)0.1
940:0.00000100,SRR3091249:0.00000100)0.1971:0.00000100,SRR1792404:0.00000100)0.2000:
0.00000100,SRR8073619:0.00000100)0.2030:0.00000100,SRR1792464:0.00000100)0.2062:0.00
000100,(((SRR5817703:0.00000100,SRR5817698:0.00000100)0.1233:0.00000100,SRR1791694:
0.00000100)0.2033:0.00000100,SRR1791812:0.00000100)0.2878:0.00010800)0.2159:0.000001
00,SRR8073623:0.00000100)0.2187:0.00000100,SRR1792335:0.00000100)0.2222:0.00000100,S

RR8073601:0.00000100)0.2252:0.00000100,SRR1792408:0.00000100)0.2277:0.00000100,SRR3091233:0.00000100)0.2307:0.00000100,SRR1792000:0.00000100)0.2339:0.00000100,SRR8073640:0.00000100)0.2376:0.00000100,SRR3091236:0.00000100)0.2405:0.00000100,SRR1791718:0.00000100)0.2434:0.00000100,SRR1792388:0.00000100)0.2467:0.00000100,SRR1792021:0.00000100)0.2493:0.00000100,SRR1792451:0.00000100)0.2522:0.00000100,SRR1792376:0.00000100)0.2549:0.00000100,SRR1792290:0.00000100)0.2580:0.00000100,SRR1792467:0.00000100)0.2608:0.00000100,SRR1792344:0.00000100)0.2630:0.00000100,SRR4019487:0.00000100)0.2659:0.00000100,SRR1792428:0.00010800)0.2687:0.00000100,SRR1791986:0.00000100)0.2716:0.00000100,SRR1792456:0.00000100)0.2745:0.00000100,(SRR1791825:0.00000100,SRR1791817:0.00000100)0.2667:0.00010800)0.2801:0.00000100,SRR8073632:0.00000100)0.2826:0.00000100,SRR1792381:0.00000100)0.2858:0.00000100,SRR8073655:0.00000100)0.2884:0.00000100,SRR1792306:0.00000100)0.2915:0.00000100,SRR1792022:0.00000100)0.2942:0.00000100,SRR1792025:0.00000100)0.2973:0.00000100,SRR5817716:0.00000100)0.3002:0.00000100,SRR1792379:0.00000100)0.3029:0.00000100,SRR1792391:0.00000100)0.3053:0.00000100,SRR8073614:0.00000100)0.3083:0.00000100,SRR1792375:0.00000100)0.3110:0.00000100,SRR8073669:0.00000100)0.3143:0.00000100,SRR1792289:0.00000100)0.3173:0.00000100,SRR1792385:0.00000100)0.3200:0.00000100,SRR8073681:0.00000100)0.3228:0.00000100,SRR1792325:0.00000100)0.3249:0.00000100,SRR3091256:0.00000100)0.3278:0.00000100,SRR8073643:0.00010800)0.3304:0.00000100,SRR1792023:0.00010800)0.3329:0.00000100,SRR8073664:0.00000100)0.3355:0.00000100,SRR3091253:0.00000100)0.3379:0.00000100,SRR1792056:0.00000100)0.3405:0.00000100,SRR8073616:0.00000100)0.3436:0.00000100,SRR1792462:0.00000100)0.3460:0.00000100,SRR1792465:0.00000100)0.3480:0.00000100,SRR8073673:0.00000100)0.3506:0.00000100,SRR1792387:0.00000100)0.3529:0.00000100,SRR1791803:0.00000100)0.3553:0.00000100,SRR8073645:0.00000100)0.3579:0.00000100,SRR1792396:0.00000100)0.3606:0.00000100,SRR8073676:0.00000100)0.3630:0.00000100,SRR3091252:0.00000100)0.3652:0.00000100,SRR8073603:0.00000100)0.3676:0.00000100,SRR1792403:0.00000100)0.3703:0.00000100,SRR1792501:0.00000100)0.3732:0.00000100,SRR1792468:0.00000100)0.3756:0.00000100,SRR1792433:0.00000100)0.3778:0.00000100,SRR1791810:0.00000100)0.3802:0.00000100,SRR8073642:0.00000100)0.3824:0.00000100,SRR3091234:0.00000100)0.3847:0.00000100,SRR1792324:0.00000100)0.3872:0.00000100,SRR8073607:0.00000100)0.3897:0.00000100,(SRR5817718:0.00000100,SRR8073626:0.00000100)0.2233:0.00010800)0.3939:0.00000100,SRR1792407:0.00000100)0.3964:0.00000100,SRR1792012:0.00000100)0.3987:0.00000100,SRR1792098:0.00000100)0.4008:0.00000100,SRR1792032:0.00000100)0.4037:0.00000100,SRR1791731:0.00000100)0.4057:0.00000100,SRR1792402:0.00000100)0.4076:0.00000100,SRR1792386:0.00000100)0.4098:0.00000100,SRR1792185:0.00000100)0.4119:0.00000100,SRR1792374:0.00000100)0.4143:0.00000100,SRR1792024:0.00000100)0.4166:0.00010800,(((SRR8073641:0.00000100,SRR1791730:0.00000100)0.0067:0.00000100,SRR8073668:0.00000100)0.0133:0.00000100,SRR5817692:0.00000100)0.0189:0.00000100,SRR8073620:0.00000100)0.0175:0.00000100,((((((SRR1792457:0.00000100,SRR8073635:0.00000100)0.0433:0.00000100,SRR8073648:0.00000100)0.0900:0.00000100,SRR5817701:0.00000100)0.1322:0.00000100,SRR1791699:0.00000100)0.1967:0.00000100,SRR1791757:0.00000100)0.2473:0.00000100,SRR8073650:0.00000100)0.2806:0.00000100,SRR1792160:0.00000100)0.3152:0.00010800)0.1919:0.00000100,((((SRR8073674:0.00000100,SRR8073671:0.00000100)0.2867:0.00010800,SRR8073680:0.00000100)0.2517:0.00000100,SRR8073651:0.00000100)0.2989:0.00000100,SRR8073665:0.00

000100)0.3408:0.00010800)0.1737:0.00000100,SRR1792018:0.00010800)0.1656:0.00000100,S
RR1792383:0.00000100)0.1575:0.00000100,SRR1792507:0.00000100)0.1505:0.00000100,SRR5
817686:0.00000100)0.1437:0.00000100,SRR8073663:0.00000100)0.1380:0.00000100,SRR8073
625:0.00000100)0.1330:0.00000100,SRR8073633:0.00000100)0.1282:0.00000100,SRR1791775:
0.00000100)0.1247:0.00000100,SRR8073639:0.00000100)0.1214:0.00000100,SRR8073604:0.00
000100)0.1172:0.00000100,((((((SRR8073662:0.00000100,SRR1791698:0.00000100)0.0700:0.0
0000100,SRR8073636:0.00000100)0.1417:0.00000100,SRR8073653:0.00000100)0.1744:0.0000
0100,SRR1791772:0.00000100)0.2167:0.00000100,SRR1792272:0.00000100)0.2713:0.0000010
0,SRR1792271:0.00000100)0.3117:0.00010800)0.1193:0.00000100,SRR8073608:0.00000100)0.
1173:0.00000100,SRR1792089:0.00000100)0.1166:0.00000100,SRR8073675:0.00000100)0.115
4:0.00000100,SRR8073660:0.00000100)0.1147:0.00000100,SRR1792253:0.00000100)0.1156:0.
00000100,SRR1792267:0.00010800)0.1164:0.00000100,SRR8073678:0.00000100)0.1173:0.000
00100,SRR1792427:0.00000100)0.1197:0.00000100,SRR8073627:0.00000100)0.1236:0.000001
00,SRR8073677:0.00000100)0.1264:0.00000100,SRR8073638:0.00010800)0.1298:0.00000100,S
RR5817688:0.00000100)0.1339:0.00000100,SRR1791902:0.00000100)0.1401:0.00000100,SRR8
073644:0.00000100)0.1472:0.00000100,SRR5817676:0.00010800)0.1539:0.00000100,SRR1792
256:0.00000100)0.1607:0.00000100,SRR5817705:0.00000100)0.1683:0.00000100,SRR1792255:
0.00000100)0.1766:0.00000100,SRR1792424:0.00000100)0.1844:0.00000100,SRR1792503:0.00
000100)0.1922:0.00000100,SRR8073629:0.00000100)0.2007:0.00000100,SRR1792266:0.00000
100)0.2073:0.00000100,SRR5817683:0.00000100)0.2147:0.00000100,SRR8073631:0.00000100
)0.2211:0.00000100,SRR8073658:0.00000100)0.2280:0.00000100,SRR5817693:0.00000100)0.2
341:0.00000100,SRR8073621:0.00000100)0.2413:0.00000100,SRR8073613:0.00010800)0.2475:
0.00000100,SRR5817680:0.00000100)0.2541:0.00000100,SRR5817711:0.00000100)0.2611:0.00
000100,SRR1792252:0.00000100)0.2679:0.00000100,SRR5817714:0.00000100)0.2747:0.00000
100,SRR1792431:0.00000100)0.2815:0.00000100,SRR1792425:0.00000100)0.2876:0.00000100,
SRR1792265:0.00000100)0.2937:0.00000100,SRR4019492:0.00021600)0.2986:0.00000100,SRR
8073649:0.00000100)0.3035:0.00000100,SRR1792054:0.00000100)0.3094:0.00000100,SRR581
7684:0.00000100)0.3151:0.00000100,SRR8073637:0.00000100)0.3204:0.00000100,SRR807363
4:0.00000100)0.3256:0.00000100,SRR8073612:0.00010800)0.3305:0.00000100,SRR5817672:0.
00010800)0.3350:0.00000100,SRR5817681:0.00000100)0.3412:0.00000100,SRR5817679:0.000
00100)0.3468:0.00000100,SRR5817682:0.00000100)0.3513:0.00000100,SRR8073602:0.000108
00)0.3544:0.00000100,SRR8073611:0.00000100)0.3585:0.00000100,SRR1792430:0.00000100)
0.3630:0.00000100,SRR1792426:0.00000100)0.3677:0.00000100,SRR1792452:0.00000100)0.37
29:0.00010800)0.4545:0.00000100,((((((((((((((((((((((SRR11462889:0.00010800,SRR11462911:0
.00000100)0.0200:0.00000100,SRR11462888:0.00000100)0.0350:0.00000100,SRR11462876:0.0
0010800)0.0511:0.00000100,SRR11462933:0.00010800)0.0675:0.00000100,SRR11462867:0.00
000100)0.0693:0.00000100,SRR11462910:0.00010800)0.0744:0.00000100,((((SRR11462895:0.0
0010800,SRR11462862:0.00000100)0.1133:0.00000100,SRR11462866:0.00010800)0.1850:0.00
000100,SRR11462915:0.00000100)0.2522:0.00000100,SRR11462900:0.00000100)0.3242:0.000
10800)0.1761:0.00000100,SRR11462916:0.00010800)0.1825:0.00000100,SRR11462896:0.0000
0100)0.1856:0.00000100,SRR11462905:0.00010800)0.2000:0.00000100,SRR11462890:0.00000
100)0.2158:0.00000100,SRR11462909:0.00000100)0.2396:0.00000100,SRR11462880:0.000108
00)0.2618:0.00000100,SRR11462875:0.00000100)0.2865:0.00000100,SRR11462860:0.0002160
0)0.3025:0.00000100,SRR11462897:0.00000100)0.3252:0.00000100,SRR11462925:0.00000100

)0.3448:0.00000100,SRR11462908:0.00010800)0.3638:0.00000100,SRR11462929:0.00000100)0.3806:0.00000100,SRR11462881:0.00000100)0.3972:0.00000100,SRR11462922:0.00000100)0.4143:0.00000100,SRR11462886:0.00010800)0.4285:0.00000100,SRR11462898:0.00000100)0.4433:0.00021600)0.4882:0.00043200,((SRR14296277:0.00000100,SRR14296257:0.00000100)0.2367:0.00010800,((((((((((((((((((((((((((((((SRR14296292:0.00000100,SRR5657405:0.00000100)0.0033:0.00000100,((((SRR5657481:0.00010800,SRR14296308:0.00010800)0.0633:0.00000100,SRR14296272:0.00000100)0.1317:0.00000100,(SRR14296304:0.00000100,SRR14296156:0.00000100)0.3767:0.00021600)0.3108:0.00000100,SRR5657493:0.00000100)0.3627:0.00010800)0.2614:0.00000100,SRR14296331:0.00000100)0.2329:0.00000100,SRR14296163:0.00000100)0.2111:0.00000100,SRR14296201:0.00010800)0.1950:0.00000100,SRR14296426:0.00000100)0.1800:0.00000100,((((((SRR14296396:0.00000100,SRR14296402:0.00000100)0.0567:0.00000100,SRR14296398:0.00000100)0.1017:0.00000100,(SRR14296199:0.00000100,SRR14296400:0.00000100)0.2767:0.00010800)0.1967:0.00000100,SRR14296198:0.00000100)0.2273:0.00000100,SRR5657443:0.00000100)0.2717:0.00000100,SRR14296378:0.00010800)0.3038:0.00000100,SRR5657444:0.00000100)0.3408:0.00010800)0.1975:0.00000100,SRR14296296:0.00010800)0.1940:0.00000100,SRR14296320:0.00010800)0.1908:0.00000100,SRR5657463:0.00000100)0.1914:0.00000100,SRR14296193:0.00000100)0.1918:0.00000100,SRR5657494:0.00010800)0.1924:0.00000100,SRR14296369:0.00000100)0.1994:0.00000100,SRR5657402:0.00000100)0.2053:0.00000100,SRR14296141:0.00000100)0.2138:0.00000100,SRR14296151:0.00000100)0.2249:0.00000100,(SRR14296368:0.00000100,SRR14296091:0.00000100)0.2767:0.00010800)0.2454:0.00000100,SRR5657445:0.00000100)0.2543:0.00000100,SRR14296107:0.00000100)0.2626:0.00000100,SRR14296194:0.00010800)0.2706:0.00000100,SRR14296401:0.00000100)0.2805:0.00000100,SRR5657383:0.00000100)0.2894:0.00000100,SRR14296307:0.00000100)0.2970:0.00000100,SRR14296289:0.00000100)0.3052:0.00000100,SRR14296329:0.00000100)0.3133:0.00000100,SRR14296428:0.00000100)0.3192:0.00000100,SRR14296353:0.00010800)0.3270:0.00000100,SRR14296231:0.00000100)0.3352:0.00000100,SRR14296330:0.00000100)0.3416:0.00000100,SRR5657462:0.00010800)0.3468:0.00010800)0.3318:0.00000100,SRR14296315:0.00000100)0.3248:0.00000100,SRR1792063:0.00010800)0.3181:0.00000100,SRR14296187:0.00000100)0.3116:0.00000100,((((((((((((SRR14296386:0.00000100,SRR14296422:0.00000100)0.1200:0.00000100,SRR14296429:0.00000100)0.2383:0.00000100,SRR14296382:0.00000100)0.3500:0.00000100,SRR14296225:0.00000100)0.4283:0.00021600,((SRR14296264:0.00000100,(((((SRR14296100:0.00000100,SRR14296216:0.00000100)0.1233:0.00000100,SRR1791865:0.00000100)0.1783:0.00000100,SRR14296254:0.00000100)0.2844:0.00000100,SRR14296209:0.00000100)0.3717:0.00000100,SRR14296409:0.00000100)0.4247:0.00010800)0.4067:0.00000100,SRR5657431:0.00000100)0.4162:0.00010800)0.2978:0.00000100,(((((((SRR14296111:0.00021600,(SRR14296427:0.00000100,SRR14296410:0.00000100)0.2800:0.00010800)0.1917:0.00000100,SRR14296243:0.00000100)0.1756:0.00000100,SRR5657507:0.00010800)0.1967:0.00000100,SRR14296125:0.00010800)0.2280:0.00000100,SRR14296404:0.00000100)0.2817:0.00000100,SRR14296403:0.00010800)0.3276:0.00000100,SRR14296405:0.00000100)0.3588:0.00010800)0.2811:0.00000100,SRR14296349:0.00000100)0.2897:0.00000100,((SRR14296282:0.00000100,SRR14296283:0.00000100)0.2433:0.00000100,SRR14296284:0.00000100)0.4533:0.00032400)0.3149:0.00000100,SRR14296203:0.00000100)0.3228:0.00000100,SRR14296154:0.00000100)0.3314:0.00000100,SRR1429642

5:0.00000100)0.3404:0.00000100,SRR14296397:0.00000100)0.3464:0.00000100,SRR14296381:0.00021600)0.3511:0.00010800)0.2265:0.00000100,(((((((((((((((((((((((((((SRR14296280:0.00000100,SRR1792448:0.00000100)0.1000:0.00000100,SRR14296270:0.00021600)0.1750:0.00000100,SRR5657446:0.00000100)0.2556:0.00000100,SRR14296376:0.00000100)0.3217:0.00010800,SRR5657419:0.00000100)0.2627:0.00000100,SRR14296379:0.00010800)0.2372:0.00000100,SRR14296363:0.00000100)0.2129:0.00000100,SRR14296212:0.00000100)0.1967:0.00000100,SRR14296251:0.00000100)0.1833:0.00000100,SRR14296414:0.00000100)0.1767:0.00000100,SRR1792455:0.00000100)0.1761:0.00000100,SRR14296119:0.00000100)0.1736:0.00000100,SRR5657395:0.00000100)0.1797:0.00000100,SRR14296313:0.00021600)0.1898:0.00000100,SRR14296250:0.00000100)0.2051:0.00000100,SRR5657487:0.00000100)0.2208:0.00000100,SRR14296362:0.00000100)0.2412:0.00000100,SRR14296097:0.00000100)0.2628:0.00000100,SRR5657468:0.00010800)0.2863:0.00000100,SRR14296146:0.00000100)0.3062:0.00000100,SRR14296249:0.00000100)0.3289:0.00000100,SRR5657505:0.00021600)0.3485:0.00000100,SRR14296359:0.00000100)0.3664:0.00000100,SRR5657475:0.00000100)0.3867:0.00000100,SRR1792071:0.00000100)0.4044:0.00000100,SRR5657420:0.00000100)0.4176:0.00000100,SRR5657368:0.00010800)0.4294:0.00021600)0.1845:0.00000100,SRR5657382:0.00000100)0.1829:0.00000100,(((((((((((((SRR5657375:0.00000100,SRR5657500:0.00000100)0.0433:0.00000100,SRR14296344:0.00000100)0.0467:0.00000100,SRR14296278:0.00000100)0.0611:0.00000100,SRR14296323:0.00000100)0.0775:0.00000100,SRR14296087:0.00000100)0.0973:0.00000100,SRR14296188:0.00000100)0.1139:0.00000100,SRR5657458:0.00000100)0.1381:0.00000100,SRR5657524:0.00000100)0.1746:0.00000100,SRR5657439:0.00000100)0.2056:0.00000100,SRR14296190:0.00000100)0.2343:0.00000100,SRR5657388:0.00000100)0.2576:0.00000100,SRR14296103:0.00000100)0.2869:0.00000100,SRR5657378:0.00000100)0.3026:0.00010800)0.1645:0.00000100,((SRR14296298:0.00000100,SRR14296294:0.00000100)0.1533:0.00000100,SRR5657452:0.00000100)0.3000:0.00010800)0.1610:0.00000100,SRR5657509:0.00000100)0.1598:0.00000100,(((((SRR14296102:0.00000100,SRR14296419:0.00021600)0.0800:0.00000100,SRR14296372:0.00021600)0.1617:0.00000100,SRR14296274:0.00000100)0.2567:0.00000100,SRR5657411:0.00010800)0.3025:0.00000100,SRR14296166:0.00021600)0.3500:0.00010800)0.1536:0.00000100,SRR5657495:0.00000100)0.1524:0.00000100,SRR5657412:0.00000100)0.1513:0.00000100,(((((((((((((((((((SRR14296213:0.00000100,SRR14296175:0.00000100)0.0233:0.00000100,SRR14296340:0.00000100)0.0317:0.00000100,SRR14296364:0.00000100)0.0456:0.00000100,SRR14296291:0.00000100)0.0558:0.00000100,SRR14296133:0.00000100)0.0660:0.00000100,SRR5657498:0.00000100)0.0811:0.00000100,SRR14296117:0.00010800)0.0848:0.00000100,SRR5657373:0.00000100)0.1017:0.00000100,SRR14296099:0.00000100)0.1204:0.00000100,SRR14296132:0.00000100)0.1433:0.00000100,SRR14296121:0.00000100)0.1642:0.00000100,SRR14296174:0.00000100)0.1844:0.00000100,SRR5657503:0.00000100)0.2064:0.00000100,SRR14296098:0.00000100)0.2248:0.00000100,SRR5657496:0.00000100)0.2473:0.00000100,SRR14296321:0.00000100)0.2635:0.00000100,SRR14296399:0.00000100)0.2780:0.00000100,SRR5657374:0.00000100)0.2902:0.00000100,SRR5657502:0.00000100)0.3060:0.00010800)0.1359:0.00000100,SRR14296185:0.00000100)0.1351:0.00000100,SRR14296324:0.00000100)0.1343:0.00000100,((SRR5657392:0.00000100,SRR14296411:0.00010800)0.1800:0.00000100,(SRR1792411:0.00000100,SRR1792442:0.00000100)0.2833:0.00010800)0.4389:0.00021600)0.1315:0.00000100,(((SRR14296371:0.00000100,SRR14296131:0.00000100)0.1067:0.00000100,SRR5657404:0.00000100)0.2300:0.00000100,SRR14296215:0.00000100)0.2944:0.00010800)0.1287:0.00000100,SRR14296208:0.00010800)0.1280:0.00000

100,SRR5657380:0.00000100)0.1273:0.00000100,(((SRR14296352:0.00000100,SRR14296365:0.
00000100)0.3167:0.00010800,SRR14296139:0.00000100)0.3317:0.00000100,SRR14296167:0.0
0000100)0.4222:0.00021600)0.1256:0.00000100,SRR5657457:0.00010800)0.1250:0.00000100,
((((((SRR14296159:0.00000100,SRR14296140:0.00010800)0.0767:0.00000100,SRR14296161:0.
00000100)0.1417:0.00000100,SRR14296101:0.00000100)0.2222:0.00000100,SRR14296204:0.0
0000100)0.2992:0.00000100,SRR14296124:0.00000100)0.3553:0.00000100,SRR14296128:0.00
000100)0.4172:0.00000100,SRR14296180:0.00000100)0.4652:0.00032400)0.1215:0.00000100,
SRR5657504:0.00000100)0.1209:0.00000100,((SRR5657430:0.00000100,SRR14296357:0.00000
100)0.2300:0.00000100,SRR14296207:0.00000100)0.4100:0.00021600)0.1197:0.00000100,SRR
14296302:0.00010800)0.1192:0.00000100,(((((((((SRR5657470:0.00000100,SRR14296388:0.00
000100)0.0567:0.00000100,SRR14296222:0.00000100)0.0917:0.00000100,SRR14296266:0.000
00100)0.1400:0.00000100,SRR14296262:0.00000100)0.1742:0.00000100,SRR14296416:0.0000
0100)0.2193:0.00000100,SRR14296202:0.00021600)0.2517:0.00000100,SRR14296170:0.00010
800)0.3114:0.00000100,SRR5657376:0.00000100)0.3542:0.00000100,SRR14296263:0.0000010
0)0.3926:0.00000100,SRR5657387:0.00000100)0.4270:0.00021600)0.1163:0.00000100,SRR565
7408:0.00000100)0.1158:0.00000100,SRR5657485:0.00010800)0.1153:0.00000100,SRR565748
0:0.00000100)0.1148:0.00000100,(SRR14296358:0.00010800,SRR5657520:0.00000100)0.2467:
0.00010800)0.1143:0.00000100,((SRR5657399:0.00000100,SRR5657448:0.00000100)0.1600:0.
00000100,SRR5657449:0.00000100)0.3000:0.00010800)0.1135:0.00000100,((SRR5657427:0.00
000100,SRR14296162:0.00000100)0.1900:0.00000100,SRR5657386:0.00000100)0.3133:0.0001
0800)0.1124:0.00000100,(SRR1792441:0.00043200,((SRR1791873:0.00000100,SRR1791859:0.0
0000100)0.2267:0.00000100,SRR1791888:0.00000100)0.4533:0.00021600)0.4044:0.00010800)
0.1121:0.00000100,SRR14296155:0.00000100)0.1117:0.00000100,SRR5657511:0.00000100)0.1
113:0.00000100,SRR5657491:0.00000100)0.1108:0.00000100,SRR14296265:0.00000100)0.110
4:0.00000100,SRR5657474:0.00000100)0.1099:0.00000100,SRR14296144:0.00000100)0.1095:0
.00000100,(((SRR14296348:0.00000100,SRR14296295:0.00000100)0.0900:0.00000100,SRR565
7369:0.00000100)0.2033:0.00000100,SRR5657459:0.00000100)0.2911:0.00010800)0.1086:0.0
0000100,(SRR14296235:0.00000100,SRR14296293:0.00000100)0.1933:0.00010800)0.1081:0.0
0000100,SRR5657377:0.00000100)0.1078:0.00000100,SRR14296366:0.00021600)0.1076:0.000
00100,SRR14296281:0.00000100)0.1073:0.00000100,SRR14296392:0.00000100)0.1071:0.0000
0100,(((((((SRR14296173:0.00010800,SRR14296300:0.00000100)0.0500:0.00000100,SRR14296
192:0.00000100)0.0750:0.00000100,SRR14296200:0.00000100)0.1178:0.00000100,SRR142963
19:0.00000100)0.1592:0.00000100,SRR14296406:0.00000100)0.1940:0.00000100,SRR5657366:
0.00000100)0.2339:0.00000100,SRR14296095:0.00010800)0.2743:0.00000100,SRR14296322:0.
00000100)0.3008:0.00010800)0.1072:0.00000100,SRR14296413:0.00000100)0.1069:0.0000010
0,SRR14296182:0.00000100)0.1067:0.00000100,SRR5657497:0.00000100)0.1065:0.00000100,S
RR14296129:0.00000100)0.1062:0.00000100,SRR14296275:0.00000100)0.1060:0.00000100,SR
R14296138:0.00000100)0.1057:0.00000100,SRR1792355:0.00000100)0.1055:0.00000100,SRR5
657519:0.00000100)0.1052:0.00000100,SRR14296242:0.00000100)0.1049:0.00000100,SRR142
96253:0.00032400)0.1050:0.00000100,SRR14296314:0.00000100)0.1047:0.00000100,SRR5657
425:0.00021600)0.1047:0.00000100,((((SRR5657379:0.00000100,SRR5657384:0.00000100)0.08
33:0.00000100,SRR5657438:0.00000100)0.1700:0.00000100,SRR14296116:0.00000100)0.2544:
0.00000100,SRR14296297:0.00000100)0.3233:0.00010800)0.1043:0.00000100,(((SRR14296337
:0.00000100,SRR14296338:0.00000100)0.2100:0.00000100,SRR14296339:0.00000100)0.3600:0

.00010800,SRR5657447:0.00000100)0.3933:0.00010800)0.1051:0.00000100,(((SRR14296327:0.
00000100,((SRR14296326:0.00000100,SRR14296150:0.00000100)0.1100:0.00000100,SRR1429
6351:0.00000100)0.2100:0.00000100)0.3233:0.00000100,SRR14296325:0.00000100)0.4192:0.
00010800,SRR14296273:0.00000100)0.4127:0.00010800)0.1059:0.00000100,SRR5657517:0.00
000100)0.1058:0.00000100,SRR1791751:0.00010800)0.1059:0.00000100,SRR14296112:0.0000
0100)0.1060:0.00000100,SRR14296195:0.00000100)0.1060:0.00000100,SRR5657442:0.000001
00)0.1061:0.00000100,((SRR5657397:0.00000100,SRR14296153:0.00010800)0.1600:0.0000010
0,SRR5657396:0.00000100)0.2917:0.00010800)0.1069:0.00000100,SRR5657450:0.00000100)0.
1070:0.00000100,SRR5657490:0.00000100)0.1072:0.00000100,SRR5657441:0.00000100)0.107
5:0.00000100,SRR5657522:0.00000100)0.1077:0.00000100,SRR14296157:0.00000100)0.1080:0
.00000100,(((SRR5657499:0.00000100,SRR5657506:0.00000100)0.1367:0.00000100,(SRR14296
104:0.00000100,SRR14296171:0.00000100)0.3000:0.00010800)0.3344:0.00000100,SRR565747
1:0.00000100)0.4417:0.00021600)0.1098:0.00000100,((SRR14296236:0.00000100,SRR1429642
3:0.00000100)0.3400:0.00032400,SRR14296246:0.00000100)0.3267:0.00010800)0.1113:0.000
00100,(SRR14296391:0.00000100,SRR14296115:0.00000100)0.2367:0.00010800)0.1121:0.000
00100,SRR14296137:0.00000100)0.1128:0.00000100,SRR5657365:0.00000100)0.1133:0.00000
100,SRR5657398:0.00000100)0.1142:0.00000100,SRR5657469:0.00000100)0.1146:0.00000100,
(SRR14296387:0.00000100,SRR1791976:0.00000100)0.2300:0.00010800)0.1157:0.00000100,S
RR14296342:0.00010800)0.1160:0.00000100,SRR14296214:0.00000100)0.1167:0.00000100,SR
R5657421:0.00000100)0.1172:0.00000100,SRR14296148:0.00000100)0.1178:0.00000100,SRR1
4296345:0.00000100)0.1182:0.00000100,SRR14296377:0.00000100)0.1189:0.00000100,SRR14
296232:0.00010800)0.1197:0.00000100,SRR5657437:0.00000100)0.1201:0.00000100,((SRR565
7360:0.00000100,SRR14296271:0.00000100)0.1867:0.00000100,SRR5657410:0.00000100)0.30
83:0.00010800)0.1222:0.00000100,SRR5657413:0.00010800)0.1229:0.00000100,SRR14296383:
0.00000100)0.1235:0.00000100,SRR5657422:0.00000100)0.1239:0.00000100,SRR14296303:0.0
0043200)0.1248:0.00000100,SRR14296299:0.00000100)0.1254:0.00000100,SRR5657453:0.000
00100)0.1261:0.00000100,SRR14296341:0.00000100)0.1270:0.00000100,SRR14296309:0.0000
0100)0.1276:0.00000100,SRR5657527:0.00000100)0.1283:0.00000100,SRR14296407:0.000001
00)0.1292:0.00000100,SRR14296346:0.00000100)0.1297:0.00000100,SRR5657456:0.00010800
)0.1306:0.00000100,SRR14296086:0.00000100)0.1312:0.00000100,SRR14296347:0.00000100)
0.1320:0.00000100,SRR14296226:0.00000100)0.1328:0.00000100,SRR14296258:0.00000100)0.
1336:0.00000100,SRR14296415:0.00000100)0.1342:0.00000100,SRR14296241:0.00000100)0.1
348:0.00000100,SRR14296218:0.00000100)0.1355:0.00000100,SRR5657393:0.00000100)0.136
2:0.00000100,((SRR14296152:0.00000100,SRR14296424:0.00000100)0.1567:0.00000100,SRR1
4296234:0.00000100)0.2900:0.00010800)0.1386:0.00000100,SRR14296189:0.00000100)0.1394
:0.00000100,SRR5657409:0.00000100)0.1402:0.00000100,SRR5657436:0.00000100)0.1409:0.0
0000100,SRR14296228:0.00000100)0.1415:0.00000100,SRR14296147:0.00000100)0.1422:0.00
000100,SRR5657432:0.00000100)0.1430:0.00000100,SRR5657466:0.00000100)0.1437:0.00000
100,SRR14296217:0.00000100)0.1446:0.00000100,((SRR14296395:0.00000100,SRR14296279:0
.00000100)0.2533:0.00000100,SRR14296239:0.00000100)0.4883:0.00032400)0.1470:0.000001
00,SRR5657426:0.00000100)0.1478:0.00000100,(((SRR14296390:0.00000100,SRR14296160:0.0
0000100)0.2500:0.00010800,SRR5657489:0.00000100)0.2300:0.00000100,SRR14296127:0.000
00100)0.2956:0.00010800)0.1504:0.00000100,SRR14296385:0.00000100)0.1510:0.00000100,S
RR14296230:0.00000100)0.1518:0.00000100,SRR5657403:0.00000100)0.1525:0.00000100,SRR

5657415:0.00000100)0.1533:0.00000100,SRR14296176:0.00021600)0.1542:0.00000100,SRR56
57476:0.00000100)0.1550:0.00000100,SRR5657406:0.00010800)0.1558:0.00000100,SRR14296
316:0.00000100)0.1567:0.00000100,SRR5657429:0.00000100)0.1574:0.00000100,SRR1429613
5:0.00000100)0.1582:0.00000100,SRR5657510:0.00000100)0.1591:0.00000100,SRR14296123:0
.00000100)0.1600:0.00000100,SRR14296145:0.00010800)0.1610:0.00000100,SRR5657461:0.00
000100)0.1617:0.00000100,SRR5657467:0.00000100)0.1625:0.00000100,SRR14296255:0.0000
0100)0.1635:0.00000100,(SRR14296227:0.00021600,SRR5657363:0.00010800)0.3367:0.00021
600)0.1652:0.00000100,SRR5657417:0.00000100)0.1661:0.00000100,SRR14296237:0.0000010
0)0.1668:0.00000100,SRR14296179:0.00000100)0.1677:0.00000100,SRR14296245:0.00010800
)0.1683:0.00000100,SRR1792028:0.00021600)0.1691:0.00000100,SRR14296355:0.00000100)0.
1698:0.00000100,(SRR14296233:0.00000100,SRR14296211:0.00000100)0.2067:0.00010800)0.
1714:0.00000100,SRR14296350:0.00000100)0.1722:0.00000100,SRR14296261:0.00010800)0.1
731:0.00000100,SRR1791715:0.00000100)0.1738:0.00000100,SRR5657482:0.00010800)0.1746:
0.00000100,SRR14296219:0.00000100)0.1751:0.00000100,(SRR14296276:0.00000100,SRR5657
460:0.00000100)0.2200:0.00010800)0.1769:0.00000100,SRR14296106:0.00010800)0.1778:0.0
0000100,SRR14296197:0.00010800)0.1784:0.00000100,(SRR14296229:0.00000100,SRR142963
35:0.00000100)0.2267:0.00010800)0.1798:0.00000100,SRR5657364:0.00000100)0.1806:0.000
00100,SRR1791705:0.00000100)0.1815:0.00000100,((SRR14296089:0.00000100,SRR14296286:
0.00010800)0.1533:0.00000100,SRR14296168:0.00000100)0.2800:0.00010800)0.1835:0.00000
100,SRR5657464:0.00000100)0.1846:0.00000100,SRR14296311:0.00010800)0.1854:0.0000010
0,SRR14296164:0.00000100)0.1865:0.00000100,SRR14296285:0.00010800)0.1872:0.00000100,
SRR5657385:0.00000100)0.1879:0.00000100,SRR14296361:0.00000100)0.1886:0.00000100,SR
R14296178:0.00000100)0.1895:0.00000100,SRR14296287:0.00010800)0.1900:0.00000100,((SR
R14296367:0.00010800,SRR14296252:0.00000100)0.1600:0.00000100,SRR14296384:0.000001
00)0.3100:0.00010800)0.1921:0.00000100,SRR14296267:0.00010800)0.1928:0.00000100,SRR5
657371:0.00000100)0.1937:0.00000100,SRR14296096:0.00000100)0.1944:0.00000100,SRR142
96328:0.00000100)0.1949:0.00000100,SRR14296268:0.00010800)0.1958:0.00000100,SRR1429
6210:0.00000100)0.1968:0.00000100,SRR5657423:0.00000100)0.1977:0.00000100,SRR142961
96:0.00010800)0.1984:0.00000100,SRR14296290:0.00000100)0.1990:0.00000100,SRR5657488:
0.00000100)0.1998:0.00000100,SRR5657492:0.00010800)0.2004:0.00000100,SRR14296312:0.0
0000100)0.2012:0.00000100,SRR14296256:0.00000100)0.2021:0.00000100,((SRR14296333:0.0
0000100,SRR14296220:0.00010800)0.3167:0.00010800,SRR14296240:0.00000100)0.3483:0.00
010800)0.2044:0.00000100,SRR14296105:0.00021600)0.2050:0.00000100,SRR14296134:0.000
00100)0.2057:0.00000100,SRR5657454:0.00000100)0.2065:0.00000100,SRR14296221:0.00010
800)0.2073:0.00000100,SRR5657372:0.00010800)0.2080:0.00000100,SRR5657472:0.00000100
)0.2086:0.00000100,(SRR5657477:0.00000100,SRR5657400:0.00010800)0.2633:0.00010800)0.
2097:0.00000100,SRR14296259:0.00000100)0.2107:0.00000100,(SRR14296343:0.00000100,SR
R14296334:0.00000100)0.2133:0.00010800)0.2123:0.00000100,SRR14296088:0.00000100)0.2
132:0.00000100,SRR5657479:0.00000100)0.2142:0.00000100,SRR14296336:0.00000100)0.215
0:0.00000100,SRR5657508:0.00010800)0.2158:0.00000100,SRR14296260:0.00000100)0.2164:0
.00000100,SRR5657389:0.00000100)0.2172:0.00000100,SRR14296238:0.00000100)0.2180:0.00
000100,SRR14296305:0.00000100)0.2187:0.00000100,SRR5657407:0.00000100)0.2193:0.0000
0100,SRR14296373:0.00000100)0.2202:0.00000100,SRR14296149:0.00000100)0.2208:0.00000
100,SRR1791759:0.00000100)0.2214:0.00000100,SRR5657416:0.00010800)0.2221:0.00000100,

SRR14296181:0.00010800)0.2228:0.00000100,SRR14296380:0.00000100)0.2236:0.00000100,S
RR5657381:0.00000100)0.2246:0.00000100,SRR5657428:0.00010800)0.2253:0.00000100,SRR1
4296374:0.00000100)0.2260:0.00000100,SRR5657414:0.00000100)0.2268:0.00000100,SRR142
96136:0.00000100)0.2274:0.00000100,SRR14296301:0.00000100)0.2285:0.00000100,SRR1429
6120:0.00000100)0.2292:0.00000100,SRR5657501:0.00000100)0.2297:0.00000100,SRR565745
1:0.00000100)0.2304:0.00000100,SRR5657523:0.00010800)0.2311:0.00000100,(SRR14296318:
0.00000100,SRR14296223:0.00000100)0.3567:0.00032400)0.2321:0.00000100,(SRR14296169:
0.00000100,SRR14296165:0.00000100)0.2633:0.00010800)0.2337:0.00000100,SRR5657526:0.0
0000100)0.2346:0.00000100,SRR14296317:0.00010800)0.2352:0.00000100,SRR5657367:0.000
00100)0.2360:0.00000100,SRR14296332:0.00000100)0.2369:0.00000100,SRR14296247:0.0000
0100)0.2377:0.00000100,SRR14296370:0.00010800)0.2387:0.00000100,SRR5657486:0.000001
00)0.2393:0.00000100,SRR5657455:0.00010800)0.2400:0.00000100,SRR14296354:0.00000100
)0.2408:0.00000100,SRR5657473:0.00000100)0.2415:0.00000100,SRR14296183:0.00000100)0.
2422:0.00000100,SRR14296310:0.00000100)0.2431:0.00000100,SRR14296360:0.00000100)0.2
439:0.00000100,SRR14296205:0.00000100)0.2447:0.00000100,SRR5657465:0.00000100)0.245
4:0.00000100,SRR5657528:0.00000100)0.2462:0.00000100,SRR5657435:0.00000100)0.2469:0.
00000100,SRR14296093:0.00000100)0.2478:0.00000100,SRR5657433:0.00000100)0.2486:0.00
000100,SRR5657370:0.00000100)0.2495:0.00000100,SRR14296110:0.00000100)0.2502:0.0000
0100,SRR14296092:0.00010800)0.2508:0.00000100,SRR5657484:0.00010800)0.2515:0.000001
00,SRR5657401:0.00010800)0.2522:0.00000100,SRR14296418:0.00000100)0.2531:0.00000100,
SRR14296094:0.00000100)0.2541:0.00000100,SRR14296306:0.00010800)0.2549:0.00000100,S
RR14296206:0.00000100)0.2557:0.00000100,SRR5657514:0.00000100)0.2564:0.00000100,SRR
14296172:0.00010800)0.2572:0.00000100,SRR14296248:0.00000100)0.2580:0.00000100,SRR1
4296130:0.00010800)0.2587:0.00000100,SRR14296244:0.00000100)0.2594:0.00000100,SRR14
296186:0.00000100)0.2604:0.00000100,SRR14296177:0.00000100)0.2611:0.00000100,SRR565
7516:0.00010800)0.2621:0.00000100,SRR5657529:0.00000100)0.2631:0.00000100,SRR142961
42:0.00021600)0.2640:0.00000100,SRR14296356:0.00000100)0.2650:0.00000100,SRR1429611
8:0.00010800)0.2658:0.00000100,SRR5657478:0.00000100)0.2668:0.00000100,SRR5657525:0.
00000100)0.2679:0.00000100,SRR14296184:0.00000100)0.2687:0.00000100,SRR14296224:0.0
0000100)0.2698:0.00000100)0.5284:0.00032400,((((((((SRR1792159:0.00000100,SRR1792146:
0.00000100)0.1000:0.00000100,SRR1792157:0.00000100)0.1783:0.00000100,SRR1792152:0.00
000100)0.2978:0.00000100,SRR1792014:0.00000100)0.3800:0.00000100,SRR1792107:0.00000
100)0.4580:0.00010800,SRR1791889:0.00000100)0.4422:0.00000100,SRR1792494:0.00021600
)0.4705:0.00000100,SRR1791780:0.00000100)0.5267:0.00075600,(SRR4019490:0.00000100,SR
R1792350:0.00000100)0.4400:0.00075600)0.4900:0.00010800)0.5242:0.00000100,(((((((((((((((
(((((((ERR3468759:0.00000100,ERR3468755:0.00000100)0.0100:0.00000100,ERR3468599:0.00
000100)0.0267:0.00000100,ERR3468482:0.00010800)0.0356:0.00000100,(((((((ERR4187812:0.0
0000100,ERR4187803:0.00000100)0.0467:0.00000100,ERR4187712:0.00000100)0.1017:0.0000
0100,ERR4187772:0.00000100)0.1589:0.00000100,ERR4187809:0.00000100)0.2275:0.0000010
0,ERR4187829:0.00000100)0.2800:0.00000100,ERR4187997:0.00000100)0.3350:0.00000100,E
RR4187806:0.00000100)0.3752:0.00010800)0.2642:0.00000100,ERR3468552:0.00000100)0.24
83:0.00000100,ERR3468598:0.00000100)0.2415:0.00000100,ERR3468596:0.00000100)0.2362:
0.00000100,ERR3468541:0.00000100)0.2351:0.00000100,ERR3468591:0.00000100)0.2358:0.0
0000100,ERR3468773:0.00000100)0.2410:0.00000100,ERR3468533:0.00000100)0.2520:0.0000

0100,ERR3468597:0.00000100)0.2663:0.00000100,ERR4187973:0.00000100)0.2845:0.0000010
0,ERR3468605:0.00000100)0.3071:0.00000100,ERR3468766:0.00000100)0.3323:0.00000100,E
RR3468679:0.00000100)0.3523:0.00000100,ERR3468564:0.00000100)0.3742:0.00000100,ERR3
468574:0.00000100)0.3960:0.00000100,ERR4187699:0.00000100)0.4168:0.00000100,ERR4188
383:0.00000100)0.4351:0.00000100,ERR3468590:0.00000100)0.4489:0.00000100,ERR3468702
:0.00010800)0.4618:0.00000100,ERR3468742:0.00000100)0.4796:0.00000100,ERR3468647:0.0
0000100)0.4967:0.00021600)0.5276:0.00021600,((((((((((SRR1792312:0.00000100,((((((((SRR17
92318:0.00000100,SRR1792311:0.00000100)0.0333:0.00000100,SRR1792317:0.00000100)0.07
33:0.00000100,SRR1792320:0.00000100)0.1089:0.00000100,SRR1792316:0.00000100)0.1433:0
.00000100,SRR1792326:0.00000100)0.1807:0.00000100,SRR1792314:0.00010800)0.2022:0.000
00100,SRR1792342:0.00000100)0.2381:0.00000100,SRR1792315:0.00000100)0.2800:0.000001
00)0.3322:0.00000100,SRR1792332:0.00000100)0.3740:0.00000100,SRR1792327:0.00000100)
0.4088:0.00000100,SRR1792337:0.00000100)0.4475:0.00010800,(SRR1791923:0.00000100,SRR
1791938:0.00000100)0.3067:0.00010800)0.4257:0.00000100,(((SRR1792302:0.00000100,SRR1
792300:0.00000100)0.1300:0.00000100,SRR1792301:0.00000100)0.2733:0.00000100,SRR1792
298:0.00000100)0.3811:0.00010800)0.4630:0.00000100,SRR1791697:0.00010800)0.4786:0.00
000100,SRR1792443:0.00043200)0.4952:0.00010800,(SRR1792240:0.00000100,SRR1792034:0.
00010800)0.3200:0.00010800)0.5042:0.00000100,SRR1791807:0.00032400)0.5210:0.00075700
,(((((SRR1791760:0.00010800,SRR1791954:0.00010800)0.4000:0.00032400,(SRR1792297:0.000
00100,SRR1792282:0.00000100)0.4200:0.00032400)0.2856:0.00000100,(SRR1791955:0.00043
200,SRR1791746:0.00064800)0.0867:0.00000100)0.3287:0.00000100,(SRR1791799:0.0000010
0,SRR1791800:0.00000100)0.4133:0.00032400)0.4643:0.00000100,SRR1792241:0.00032400)0.
5329:0.00097300)0.5095:0.00021600)0.5274:0.00043200,ERR4187627:0.00010800)0.5268:0.0
0000100,(((((((((((((((SRR16278283:0.00000100,((SRR16278286:0.00000100,SRR16278262:0.000
00100)0.0700:0.00000100,SRR16278219:0.00000100)0.1383:0.00000100)0.2067:0.00000100,S
RR16278246:0.00000100)0.2817:0.00000100,SRR16278288:0.00000100)0.3473:0.00000100,SR
R16278255:0.00000100)0.4178:0.00000100,SRR16278290:0.00000100)0.4781:0.00010800,SRR
16278279:0.00021600)0.4929:0.00021600,(((((SRR5216727:0.00000100,SRR5216763:0.000108
00)0.4000:0.00021600,((((SRR5216799:0.00000100,SRR5216779:0.00000100)0.1033:0.0000010
0,SRR5216928:0.00000100)0.2017:0.00000100,SRR5216698:0.00000100)0.3122:0.00000100,SR
R5216884:0.00000100)0.4167:0.00010800)0.3956:0.00000100,(SRR5216970:0.00000100,SRR5
216893:0.00000100)0.3433:0.00010800)0.4679:0.00000100,SRR5216854:0.00010800)0.5148:0
.00032400,(((((SRR5216759:0.00010800,SRR5216724:0.00000100)0.0733:0.00000100,(SRR5216
774:0.00010800,SRR5216709:0.00000100)0.3033:0.00010800)0.2778:0.00000100,SRR5216906:
0.00000100)0.3683:0.00000100,SRR5216741:0.00010800)0.4460:0.00000100,SRR5216891:0.00
000100)0.5067:0.00054000)0.4877:0.00021600)0.3321:0.00000100,(((((((SRR5216700:0.00000
100,SRR5216956:0.00021600)0.0533:0.00000100,SRR5216912:0.00032400)0.0867:0.00000100,
SRR5216882:0.00000100)0.1267:0.00000100,SRR5216764:0.00021600)0.1908:0.00000100,SRR
5216876:0.00010800)0.2507:0.00000100,SRR5216715:0.00000100)0.2944:0.00000100,SRR521
6903:0.00000100)0.3371:0.00010800)0.2632:0.00000100,(((((((((((((((((((((((SRR5216834:0.0001
0800,SRR5216894:0.00000100)0.1000:0.00000100,SRR5216863:0.00000100)0.1767:0.0000010
0,(SRR5216982:0.00000100,SRR5216887:0.00000100)0.3100:0.00010800)0.3133:0.00000100,S
RR5216864:0.00000100)0.3707:0.00010800,SRR5216743:0.00021600)0.3200:0.00000100,(((((((
(SRR5216704:0.00000100,SRR5216802:0.00000100)0.0567:0.00000100,SRR5216916:0.0000010

0)0.1033:0.00000100,SRR5216911:0.00000100)0.1600:0.00000100,SRR5216909:0.00000100)0.
2100:0.00000100,SRR5216923:0.00000100)0.2513:0.00000100,SRR5216721:0.00000100)0.300
0:0.00000100,SRR5216981:0.00000100)0.3500:0.00000100,SRR5216702:0.00000100)0.3900:0.
00010800)0.2731:0.00000100,SRR5216821:0.00000100)0.2617:0.00000100,SRR5216830:0.000
00100)0.2520:0.00000100,(SRR5216980:0.00000100,SRR5216744:0.00000100)0.4000:0.00032
400)0.2481:0.00000100,SRR5216818:0.00032400)0.2490:0.00000100,((SRR5216699:0.0000010
0,SRR5216840:0.00000100)0.1867:0.00000100,SRR5216795:0.00000100)0.3417:0.00010800)0.
2616:0.00000100,SRR5216692:0.00021600)0.2753:0.00000100,(SRR5216966:0.00000100,SRR5
216846:0.00000100)0.3033:0.00010800)0.2965:0.00000100,SRR5216720:0.00010800)0.3093:0
.00000100,SRR5216769:0.00000100)0.3214:0.00000100,SRR5216845:0.00000100)0.3344:0.000
00100,SRR5216838:0.00000100)0.3457:0.00000100,SRR5216861:0.00000100)0.3578:0.000001
00,SRR5216839:0.00000100)0.3676:0.00000100,SRR5216926:0.00021600)0.3762:0.00000100,S
RR5216693:0.00010800)0.3865:0.00000100,SRR5216794:0.00010800)0.3941:0.00000100,SRR5
216796:0.00021600)0.4019:0.00010800)0.2760:0.00000100,((((((((SRR5216793:0.00010800,((S
RR5216847:0.00000100,SRR5216913:0.00000100)0.3533:0.00010800,SRR5216829:0.00010800
)0.4167:0.00010800)0.3144:0.00000100,SRR5216751:0.00000100)0.2833:0.00000100,SRR5216
742:0.00000100)0.2800:0.00000100,SRR5216853:0.00000100)0.2939:0.00000100,SRR5216825:
0.00000100)0.3233:0.00000100,SRR5216908:0.00000100)0.3717:0.00000100,SRR5216889:0.00
000100)0.4137:0.00000100,SRR5216983:0.00000100)0.4587:0.00021600)0.2942:0.00000100,((
((SRR5216740:0.00010800,SRR5216965:0.00010800)0.2367:0.00000100,SRR5216943:0.000001
00)0.4167:0.00021600,((((((((((((((SRR5216713:0.00000100,SRR5216788:0.00000100)0.0333:0
.00000100,SRR5216707:0.00000100)0.0467:0.00000100,SRR5216773:0.00000100)0.0756:0.000
00100,SRR5216772:0.00000100)0.0967:0.00000100,SRR5216805:0.00000100)0.1253:0.000001
00,SRR5216736:0.00000100)0.1428:0.00000100,SRR5216881:0.00000100)0.1667:0.00000100,(
SRR5216880:0.00000100,SRR5216748:0.00000100)0.2867:0.00010800)0.2026:0.00000100,SRR
5216941:0.00000100)0.2300:0.00000100,SRR5216783:0.00000100)0.2630:0.00000100,SRR521
6718:0.00000100)0.2994:0.00000100,SRR5216940:0.00000100)0.3359:0.00000100,SRR521695
9:0.00000100)0.3624:0.00000100,SRR5216865:0.00010800)0.3900:0.00000100,SRR5216787:0.
00010800)0.4150:0.00000100,SRR5216798:0.00000100)0.4396:0.00010800)0.4187:0.00000100
,(SRR5216976:0.00032400,SRR5216892:0.00032400)0.2567:0.00010800)0.4261:0.00010800)0.
3535:0.00000100,(((SRR11418335:0.00010800,(SRR15296925:0.00000100,SRR11418328:0.000
00100)0.2933:0.00010800)0.2450:0.00000100,(SRR11418313:0.00000100,SRR15296942:0.000
10800)0.3233:0.00010800)0.3192:0.00000100,SRR11418331:0.00010800)0.3427:0.00010800)0
.3680:0.00000100,SRR5216952:0.00000100)0.3709:0.00000100,(SRR16278238:0.00010800,SRR
16278222:0.00000100)0.3167:0.00043200)0.3740:0.00000100,(SRR5216706:0.00010800,SRR5
216835:0.00000100)0.4300:0.00043200)0.3788:0.00010800)0.4699:0.00000100,ERR3439546:0
.00000100)0.4694:0.00000100,(((
ERR5162099:0.00000100,ERR4188358:0.00000100)0.0100:0.00000100,ERR4187623:0.0000010
0)0.0117:0.00000100,ERR4187703:0.00000100)0.0133:0.00000100,ERR4187878:0.00000100)0.
0125:0.00000100,(((((ERR4188110:0.00000100,ERR4187967:0.00000100)0.0900:0.00000100,ER
R4187963:0.00000100)0.1400:0.00000100,ERR4187999:0.00000100)0.2056:0.00000100,ERR41
88154:0.00000100)0.2550:0.00000100,ERR4187885:0.00000100)0.3113:0.00010800)0.1637:0.
00000100,ERR4188151:0.00000100)0.1512:0.00000100,ERR4188231:0.00000100)0.1394:0.000
00100,ERR3468695:0.00000100)0.1300:0.00000100,ERR4188226:0.00000100)0.1226:0.000001

00,ERR4188146:0.00000100)0.1158:0.00000100,ERR5162114:0.00000100)0.1098:0.00000100,
ERR4188424:0.00000100)0.1039:0.00000100,ERR4187976:0.00000100)0.0993:0.00000100,ERR
4188329:0.00000100)0.0942:0.00000100,ERR4187849:0.00000100)0.0913:0.00000100,ERR418
7846:0.00000100)0.0883:0.00000100,ERR4188419:0.00000100)0.0856:0.00000100,ERR418811
9:0.00000100)0.0825:0.00000100,ERR4188313:0.00000100)0.0806:0.00000100,ERR4187910:0.
00000100)0.0787:0.00000100,ERR4188423:0.00000100)0.0763:0.00000100,ERR4187789:0.000
00100)0.0746:0.00000100,ERR3468734:0.00000100)0.0727:0.00000100,ERR4187762:0.000001
00)0.0717:0.00000100,ERR5162125:0.00000100)0.0708:0.00000100,ERR4187871:0.00000100)
0.0702:0.00000100,ERR4187791:0.00000100)0.0696:0.00000100,ERR4187650:0.00000100)0.0
697:0.00000100,ERR4187759:0.00000100)0.0702:0.00000100,ERR3468692:0.00000100)0.0702
:0.00000100,ERR4187782:0.00000100)0.0711:0.00000100,ERR4188355:0.00000100)0.0709:0.0
0000100,ERR4187786:0.00000100)0.0721:0.00000100,ERR4188356:0.00000100)0.0722:0.0000
0100,ERR5162062:0.00000100)0.0727:0.00000100,ERR4188143:0.00000100)0.0743:0.0000010
0,ERR4187984:0.00000100)0.0763:0.00000100,ERR5162066:0.00000100)0.0775:0.00000100,E
RR3439536:0.00000100)0.0814:0.00000100,ERR4188422:0.00000100)0.0847:0.00000100,ERR4
187752:0.00000100)0.0884:0.00000100,ERR4188217:0.00000100)0.0933:0.00000100,ERR4187
764:0.00000100)0.0978:0.00000100,ERR4187790:0.00000100)0.1035:0.00000100,ERR4187746
:0.00000100)0.1090:0.00000100,ERR4188361:0.00000100)0.1156:0.00000100,ERR4188005:0.0
0000100)0.1200:0.00000100,ERR3468611:0.00010800)0.1253:0.00000100,ERR4188141:0.0000
0100)0.1323:0.00000100,ERR5162080:0.00000100)0.1387:0.00000100,ERR3468703:0.0000010
0)0.1465:0.00000100,ERR5162077:0.00000100)0.1546:0.00000100,ERR4188029:0.00010800)0.
1607:0.00000100,ERR3468654:0.00000100)0.1685:0.00000100,ERR4188435:0.00000100)0.173
7:0.00000100,ERR4188060:0.00010800)0.1796:0.00000100,ERR3439227:0.00000100)0.1875:0.
00000100,ERR4187785:0.00000100)0.1927:0.00000100,ERR4187914:0.00000100)0.1992:0.000
00100,ERR5162070:0.00000100)0.2053:0.00000100,ERR4188004:0.00000100)0.2113:0.000001
00,ERR4187906:0.00000100)0.2173:0.00000100,ERR4188127:0.00000100)0.2215:0.00000100,
ERR5162065:0.00000100)0.2265:0.00000100,ERR4187779:0.00000100)0.2318:0.00000100,ERR
4187783:0.00000100)0.2366:0.00000100,ERR4188113:0.00000100)0.2419:0.00000100,ERR516
2058:0.00000100)0.2469:0.00000100,ERR4188410:0.00000100)0.2518:0.00000100,ERR418832
6:0.00000100)0.2568:0.00000100,ERR4188297:0.00000100)0.2612:0.00000100,ERR4187888:0.
00000100)0.2658:0.00000100,ERR4187903:0.00000100)0.2705:0.00000100,ERR4188360:0.000
00100)0.2750:0.00000100,ERR4187780:0.00000100)0.2788:0.00000100,ERR4188429:0.000108
00)0.2831:0.00000100,ERR4188063:0.00000100)0.2867:0.00000100,ERR4188248:0.00000100)
0.2907:0.00000100,ERR3468700:0.00000100)0.2942:0.00000100,ERR4187899:0.00000100)0.2
983:0.00000100,ERR3468628:0.00000100)0.3025:0.00000100,ERR3439228:0.00000100)0.3060
:0.00000100,ERR4187852:0.00000100)0.3096:0.00000100,ERR4187902:0.00000100)0.3129:0.0
0000100,ERR4188140:0.00000100)0.3163:0.00000100,ERR4188390:0.00000100)0.3192:0.0001
0800)0.4324:0.00000100,ERR5162131:0.00000100)0.4320:0.00000100,ERR4188366:0.0000010
0)0.4316:0.00000100,ERR3439326:0.00000100)0.4313:0.00000100,ERR3439334:0.00000100)0.
4309:0.00000100,ERR3468581:0.00000100)0.4305:0.00000100,ERR3439486:0.00000100)0.430
1:0.00000100,ERR3468465:0.00010800)0.4298:0.00000100,ERR4188412:0.00000100)0.4294:0.
00000100,ERR4188086:0.00000100)0.4290:0.00000100,ERR4188227:0.00000100)0.4286:0.000
00100,ERR3439500:0.00000100)0.4283:0.00000100,(ERR4188009:0.00000100,ERR4187947:0.0
0000100)0.1967:0.00010800)0.4275:0.00000100)0.4202:0.00000100,ERR4187745:0.00000100)

0.4198:0.00000100,ERR3439290:0.00000100)0.4195:0.00000100,(((((SRR1792343:0.00000100,
SRR1792330:0.00000100)0.2600:0.00000100,SRR1792329:0.00000100)0.3900:0.00010800,(((((
(((((((SRR1792270:0.00000100,(((((((SRR1792276:0.00000100,SRR1792273:0.00000100)0.0600:
0.00000100,SRR1792254:0.00000100)0.1117:0.00000100,SRR1792275:0.00000100)0.1711:0.00
000100,SRR1792268:0.00000100)0.2300:0.00000100,SRR1792274:0.00000100)0.3040:0.00000
100,SRR1792278:0.00000100)0.3739:0.00000100,SRR1792260:0.00010800)0.4262:0.00000100
)0.4775:0.00010800,SRR1792363:0.00010800)0.4719:0.00000100,SRR1792001:0.00021600)0.4
850:0.00000100,SRR1791727:0.00000100)0.5279:0.00032400,((((SRR4019484:0.00000100,SRR
1792077:0.00000100)0.0800:0.00000100,SRR1791901:0.00000100)0.1683:0.00000100,SRR179
2045:0.00000100)0.2744:0.00000100,SRR1792042:0.00000100)0.3692:0.00010800)0.3856:0.0
0000100,SRR1791823:0.00000100)0.3690:0.00000100,SRR1791756:0.00000100)0.3546:0.0000
0100,SRR1792347:0.00000100)0.3477:0.00000100,((((SRR1791913:0.00000100,SRR1792310:0.
00054000)0.2967:0.00010800,SRR5657391:0.00000100)0.2733:0.00000100,SRR5657390:0.000
00100)0.3911:0.00000100,SRR1791926:0.00021600)0.4742:0.00021600)0.3829:0.00000100,SR
R1792162:0.00010800)0.4027:0.00000100,SRR1791878:0.00000100)0.4203:0.00000100,SRR17
92064:0.00021600)0.4381:0.00000100)0.4859:0.00000100,((SRR1792065:0.00000100,SRR1792
046:0.00000100)0.3867:0.00021600,SRR1791723:0.00010800)0.4817:0.00021600)0.5264:0.00
075700,((SRR1791816:0.00032400,SRR1791801:0.00021600)0.3933:0.00043200,(SRR15296946
:0.00021600,SRR1792438:0.00043200)0.3800:0.00032400)0.4578:0.00021600)0.5243:0.00097
300)0.4076:0.00000100,ERR3468519:0.00000100)0.4072:0.00000100,ERR3468483:0.00000100
)0.4069:0.00000100,ERR3439223:0.00000100)0.4066:0.00000100,ERR4187887:0.00000100)0.4
062:0.00000100,ERR3439274:0.00000100)0.4059:0.00000100,ERR3468538:0.00000100)0.4056
:0.00000100,ERR4187682:0.00000100)0.4053:0.00000100,ERR4187769:0.00000100)0.4049:0.0
0000100,ERR3439297:0.00000100)0.4046:0.00000100,(((S
RR5216695:0.00000100,SRR5216827:0.00021600)0.0033:0.00000100,(((SRR5216904:0.000108
00,((((((SRR5216732:0.00000100,SRR5216747:0.00010800)0.0533:0.00000100,SRR5216786:0.
00000100)0.1183:0.00000100,(SRR5216760:0.00010800,SRR5216716:0.00000100)0.2767:0.00
010800)0.2108:0.00000100,SRR5216938:0.00010800)0.2367:0.00000100,SRR5216833:0.00010
800)0.2794:0.00000100,(SRR5216878:0.00000100,SRR5216739:0.00000100)0.3000:0.0001080
0)0.3854:0.00000100,SRR5216975:0.00010800)0.4256:0.00000100)0.4600:0.00010800,(SRR52
16824:0.00000100,SRR5216890:0.00000100)0.3167:0.00010800)0.4794:0.00010800,SRR52167
23:0.00010800)0.4749:0.00010800)0.4122:0.00000100,SRR5216752:0.00010800)0.3869:0.000
00100,SRR5216791:0.00000100)0.3641:0.00000100,((((((((SRR5216895:0.00000100,SRR521681
0:0.00000100)0.2933:0.00010800,(SRR5216745:0.00000100,SRR5216778:0.00021600)0.2767:0
.00010800)0.2500:0.00000100,SRR5216785:0.00000100)0.2467:0.00000100,SRR5216866:0.000
00100)0.2787:0.00000100,SRR5216822:0.00000100)0.3211:0.00000100,SRR5216924:0.000216
00)0.3676:0.00000100,SRR5216776:0.00000100)0.4104:0.00010800,((((((((((((SRR5216735:0.0
0010800,SRR5216694:0.00000100)0.0367:0.00000100,SRR5216948:0.00000100)0.0533:0.0000
0100,SRR5216935:0.00010800)0.0800:0.00000100,SRR5216766:0.00000100)0.1083:0.0000010
0,SRR5216734:0.00000100)0.1400:0.00000100,SRR5216784:0.00000100)0.1739:0.00000100,SR
R5216780:0.00000100)0.2110:0.00000100,SRR5216701:0.00000100)0.2567:0.00000100,SRR52
16710:0.00000100)0.3056:0.00000100,SRR5216915:0.00000100)0.3443:0.00000100,SRR52169
69:0.00000100)0.3745:0.00000100,SRR5216777:0.00000100)0.4153:0.00000100,SRR5216800:0
.00010800)0.4436:0.00010800)0.5142:0.00043200)0.3000:0.00000100,(((((((((SRR5216757:0.0

0021600,(((((SRR5216874:0.00000100,SRR5216756:0.00000100)0.0733:0.00000100,SRR521696
8:0.00010800)0.1083:0.00000100,SRR5216792:0.00000100)0.1778:0.00000100,SRR5216922:0.
00043200)0.2158:0.00000100,SRR5216856:0.00010800)0.2793:0.00000100)0.3344:0.00000100
,SRR5216765:0.00000100)0.4095:0.00000100,SRR5216870:0.00000100)0.4692:0.00000100,SRR
5216896:0.00000100)0.5211:0.00021600,SRR5216728:0.00010800)0.5147:0.00010800,SRR521
6964:0.00021600)0.5000:0.00000100,SRR5216951:0.00054000)0.5100:0.00021600,(((((((((((((S
RR5216902:0.00000100,SRR5216869:0.00000100)0.3833:0.00021600,((SRR5216770:0.0006480
0,SRR5216953:0.00000100)0.1533:0.00000100,SRR5216729:0.00000100)0.3633:0.00010800)0.
3033:0.00000100,(SRR5216816:0.00000100,SRR5216919:0.00010800)0.3233:0.00010800)0.31
61:0.00000100,SRR5216858:0.00021600)0.3329:0.00000100,SRR5216900:0.00010800)0.3688:0
.00000100,SRR5216984:0.00010800)0.3993:0.00010800,((SRR5216753:0.00010800,SRR521682
3:0.00021600)0.1533:0.00000100,SRR5216898:0.00021600)0.3433:0.00010800)0.3467:0.0000
0100,((((SRR5216955:0.00010800,SRR5216812:0.00000100)0.1233:0.00000100,SRR5216963:0.
00000100)0.2167:0.00000100,SRR5216985:0.00010800)0.3089:0.00000100,SRR5216857:0.000
10800)0.3642:0.00010800)0.3747:0.00000100,SRR5216977:0.00032400)0.3920:0.00000100,SR
R5216960:0.00010800)0.4086:0.00000100,(SRR5216804:0.00032400,SRR5216872:0.00000100)
0.2267:0.00010800)0.4402:0.00000100,SRR5216972:0.00010800)0.4580:0.00000100,SRR52168
79:0.00043200)0.4725:0.00021600)0.3587:0.00000100,(((((((((((((((SRR5216917:0.00000100,(SR
R5216843:0.00000100,SRR5216873:0.00000100)0.2000:0.00000100)0.3650:0.00010800,(((SRR
5216737:0.00064800,SRR5216815:0.00054000)0.1400:0.00000100,(SRR5216730:0.00000100,S
RR5216803:0.00010800)0.0800:0.00000100)0.1722:0.00000100,SRR5216945:0.00000100)0.22
58:0.00000100)0.3638:0.00000100,SRR5216905:0.00021600)0.3975:0.00010800,(SRR5216942:
0.00021600,SRR5216897:0.00032400)0.2833:0.00010800)0.3407:0.00000100,SRR5216931:0.00
010800)0.3312:0.00000100,SRR5216696:0.00010800)0.3253:0.00000100,SRR5216749:0.00021
600)0.3338:0.00000100,SRR5216717:0.00010800)0.3412:0.00000100,SRR5216813:0.00010800
)0.3578:0.00000100,SRR5216974:0.00000100)0.3850:0.00000100,SRR5216868:0.00000100)0.4
153:0.00000100,SRR5216973:0.00010800)0.4448:0.00000100,SRR5216726:0.00000100)0.4719:
0.00000100,SRR5216901:0.00000100)0.4978:0.00000100,SRR5216930:0.00010800)0.5195:0.00
043200)0.3870:0.00000100,(((((SRR5216789:0.00000100,SRR5216806:0.00000100)0.2033:0.00
000100,SRR5216867:0.00000100)0.3817:0.00010800,SRR5216746:0.00000100)0.3656:0.00000
100,SRR5216907:0.00000100)0.4467:0.00000100,SRR5216819:0.00000100)0.5047:0.00043200
)0.4056:0.00010800)0.3099:0.00000100,(((((SRR5216755:0.00000100,((SRR5216871:0.0000010
0,(SRR5216750:0.00000100,SRR5216950:0.00000100)0.2667:0.00010800)0.2183:0.00000100,S
RR5216703:0.00010800)0.2122:0.00000100)0.2725:0.00000100,SRR5216790:0.00000100)0.32
53:0.00000100,SRR5216712:0.00000100)0.3989:0.00010800,((SRR5216849:0.00000100,SRR52
16808:0.00010800)0.3433:0.00010800,SRR5216933:0.00000100)0.4050:0.00010800)0.3996:0.
00000100,SRR5216842:0.00010800)0.4200:0.00010800)0.3265:0.00000100,((((((SRR5216807:0
.00000100,SRR5216851:0.00000100)0.1200:0.00000100,SRR5216826:0.00010800)0.1583:0.000
00100,SRR5216761:0.00000100)0.2422:0.00000100,SRR5216708:0.00000100)0.3317:0.000001
00,SRR5216925:0.00000100)0.4340:0.00000100,SRR5216921:0.00000100)0.5106:0.00032400)
0.3482:0.00000100,SRR5216828:0.00010800)0.3510:0.00000100,SRR5216971:0.00000100)0.35
26:0.00000100,SRR5216885:0.00000100)0.3557:0.00000100,SRR5216932:0.00000100)0.3581:0
.00000100,SRR5216920:0.00000100)0.3615:0.00000100,SRR5216725:0.00000100)0.3645:0.000
00100,SRR5216899:0.00010800)0.3679:0.00000100,SRR5216852:0.00010800)0.3714:0.000001

00,SRR5216962:0.00000100)0.3748:0.00000100,SRR5216714:0.00010800)0.3795:0.00000100,S
RR5216859:0.00000100)0.3835:0.00000100,SRR5216705:0.00000100)0.3875:0.00000100,SRR5
216939:0.00000100)0.3907:0.00000100,SRR5216944:0.00010800)0.3943:0.00000100,SRR5216
762:0.00010800)0.3981:0.00000100,SRR5216820:0.00000100)0.4017:0.00000100,SRR5216918:
0.00010800)0.4049:0.00000100,SRR5216733:0.00000100)0.4087:0.00000100,(SRR5216877:0.0
0000100,SRR5216860:0.00000100)0.2900:0.00010800)0.4159:0.00000100,SRR5216848:0.0000
0100)0.4193:0.00000100,SRR5216832:0.00000100)0.4227:0.00000100,SRR5216738:0.0001080
0)0.4268:0.00000100,SRR5216697:0.00000100)0.4306:0.00000100,SRR5216957:0.00000100)0.
4342:0.00000100,SRR5216767:0.00000100)0.4379:0.00000100,SRR5216690:0.00000100)0.442
2:0.00000100,SRR5216831:0.00000100)0.4459:0.00000100,SRR5216722:0.00000100)0.4493:0.
00000100,SRR5216946:0.00000100)0.4532:0.00000100,SRR5216811:0.00010800)0.4569:0.000
00100,SRR5216888:0.00000100)0.4602:0.00000100,SRR5216927:0.00000100)0.4638:0.000001
00,SRR5216837:0.00000100)0.4675:0.00000100,SRR5216782:0.00010800)0.4713:0.00000100,S
RR5216836:0.00021600)0.4747:0.00000100,SRR5216862:0.00000100)0.4782:0.00000100,SRR5
216775:0.00010800)0.4811:0.00000100,SRR5216844:0.00000100)0.4847:0.00000100,SRR5216
771:0.00000100)0.4883:0.00000100,SRR5216886:0.00000100)0.4912:0.00000100,SRR5216801:
0.00000100)0.4941:0.00000100,SRR5216875:0.00000100)0.4968:0.00000100,SRR5216817:0.00
010800)0.4997:0.00010800,(((SRR1792487:0.00032400,((ERR4352205:0.00000100,ERR4352204
:0.00000100)0.3833:0.00021600,ERR4352203:0.00010800)0.4583:0.00010800)0.4989:0.00032
400,(ERR4187894:0.00000100,ERR4187696:0.00000100)0.3933:0.00075600)0.3380:0.0000010
0,((((SRR5657521:0.00000100,((((SRR14296375:0.00000100,SRR14296122:0.00000100)0.3700:
0.00010800,SRR14296394:0.00021600)0.2767:0.00000100,((SRR1792175:0.00000100,SRR1429
6158:0.00000100)0.2067:0.00000100,SRR1792176:0.00000100)0.3667:0.00010800)0.3920:0.0
0000100,SRR14296269:0.00010800)0.4439:0.00000100)0.5167:0.00021600,SRR5657361:0.000
10800)0.5213:0.00010800,SRR1791911:0.00032400)0.5141:0.00010800,SRR1791871:0.000216
00)0.5193:0.00021600)0.3750:0.00000100)0.5147:0.00075600)0.3588:0.00000100,ERR418828
0:0.00000100)0.3586:0.00000100,ERR3468710:0.00000100)0.3583:0.00000100,ERR4187668:0.
00000100)0.3581:0.00000100,ERR5162048:0.00000100)0.3579:0.00000100,ERR3468532:0.000
00100)0.3576:0.00000100,ERR3439484:0.00000100)0.3574:0.00000100,ERR3468470:0.000001
00)0.3571:0.00000100,(((SRR14296126:0.00010800,(SRR1791970:0.00010800,SRR1791883:0.0
0021600)0.2600:0.00000100)0.4567:0.00021600,SRR1791737:0.00021600)0.4267:0.00000100,
SRR1791937:0.00021600)0.4850:0.00032400)0.3560:0.00000100,ERR3468587:0.00000100)0.3
558:0.00000100,ERR4188076:0.00000100)0.3555:0.00000100,ERR5162141:0.00000100)0.3553
:0.00000100,ERR4187933:0.00000100)0.3551:0.00000100,ERR4188262:0.00000100)0.3548:0.0
0000100,((((((ERR4187670:0.00000100,ERR4188027:0.00000100)0.1100:0.00000100,(ERR4188
092:0.00000100,ERR4188180:0.00000100)0.3500:0.00021600)0.2478:0.00000100,ERR4187974
:0.00000100)0.2900:0.00000100,ERR4187684:0.00000100)0.3520:0.00000100,ERR4188420:0.0
0000100)0.4194:0.00000100,ERR5162052:0.00000100)0.4776:0.00032400)0.3531:0.00000100,
ERR4188170:0.00000100)0.3528:0.00000100,ERR4187848:0.00000100)0.3526:0.00000100,ERR
4188229:0.00000100)0.3524:0.00000100,ERR3439362:0.00000100)0.3521:0.00000100,ERR418
7614:0.00000100)0.3519:0.00000100,ERR4187905:0.00000100)0.3517:0.00000100,ERR418832
7:0.00000100)0.3514:0.00000100,ERR4187646:0.00000100)0.3512:0.00000100,ERR3468525:0.
00000100)0.3509:0.00000100,ERR4187823:0.00000100)0.3507:0.00000100,ERR4187952:0.000
00100)0.3505:0.00000100,ERR3468782:0.00000100)0.3502:0.00000100,ERR4188182:0.000001

00)0.3500:0.00000100,ERR4188018:0.00000100)0.3498:0.00000100,ERR4188201:0.00000100)
0.3495:0.00000100,ERR4188171:0.00000100)0.3493:0.00000100,ERR4188023:0.00000100)0.3
491:0.00000100,ERR4188013:0.00000100)0.3489:0.00000100,ERR3439553:0.00010800)0.3486
:0.00000100,ERR3439237:0.00000100)0.3484:0.00000100,ERR4187728:0.00000100)0.3482:0.0
0000100,ERR3468419:0.00000100)0.3479:0.00000100,ERR4187919:0.00000100)0.3477:0.0000
0100,ERR4187664:0.00000100)0.3475:0.00000100,ERR4188251:0.00000100)0.3472:0.0000010
0,ERR4187690:0.00000100)0.3470:0.00000100,ERR3468585:0.00000100)0.3468:0.00000100,E
RR4187982:0.00000100)0.3466:0.00000100,ERR3439339:0.00000100)0.3463:0.00000100,ERR3
439300:0.00000100)0.3461:0.00000100,ERR3468650:0.00000100)0.3459:0.00000100,ERR3439
467:0.00000100)0.3457:0.00000100,ERR4187954:0.00000100)0.3454:0.00000100,ERR4188075
:0.00000100)0.3452:0.00000100,ERR3439333:0.00000100)0.3450:0.00000100,ERR4187673:0.0
0000100)0.3448:0.00000100,ERR5162112:0.00000100)0.3445:0.00000100,ERR4187859:0.0000
0100)0.3443:0.00000100,ERR4188084:0.00000100)0.3441:0.00000100,ERR3439356:0.0000010
0)0.3438:0.00000100,ERR4188052:0.00000100)0.3436:0.00000100,ERR4187835:0.00000100)0.
3434:0.00000100,ERR4188059:0.00000100)0.3432:0.00000100,ERR3468501:0.00000100)0.342
9:0.00000100,ERR4188219:0.00000100)0.3427:0.00000100,ERR3468588:0.00000100)0.3425:0.
00000100,ERR3439322:0.00000100)0.3423:0.00000100,((SRR1791784:0.00075600,(ERR017796
:0.00000100,ERR026636:0.00000100)0.3900:0.00075600)0.3917:0.00000100,SRR1792279:0.00
064800)0.4922:0.00043200)0.3415:0.00000100,ERR4188067:0.00000100)0.3413:0.00000100,E
RR4188307:0.00000100)0.3411:0.00000100,ERR4188351:0.00000100)0.3409:0.00000100,ERR4
188348:0.00000100)0.3406:0.00000100,ERR4187740:0.00000100)0.3404:0.00000100,ERR3439
547:0.00000100)0.3402:0.00000100,ERR4188282:0.00000100)0.3400:0.00000100,ERR4188206
:0.00000100)0.3397:0.00000100,ERR3439433:0.00000100)0.3395:0.00000100,ERR4187957:0.0
0000100)0.3393:0.00000100,ERR3439493:0.00000100)0.3391:0.00000100,ERR4187613:0.0000
0100)0.3389:0.00000100,ERR4188373:0.00000100)0.3386:0.00000100,ERR3439425:0.0000010
0)0.3384:0.00000100,ERR4188203:0.00000100)0.3382:0.00000100,ERR3439349:0.00000100)0.
3380:0.00000100,ERR3468430:0.00000100)0.3378:0.00000100,ERR3439562:0.00000100)0.337
6:0.00000100,ERR4188008:0.00000100)0.3373:0.00000100,ERR4188362:0.00000100)0.3371:0.
00000100,ERR3468721:0.00000100)0.3369:0.00000100,ERR4187865:0.00000100)0.3367:0.000
00100,ERR4188091:0.00000100)0.3365:0.00000100,ERR4187731:0.00000100)0.3363:0.000001
00,ERR3439305:0.00000100)0.3361:0.00000100,ERR3439258:0.00000100)0.3358:0.00000100,
ERR3439552:0.00000100)0.3356:0.00000100,ERR3439390:0.00000100)0.3354:0.00000100,ERR
4187795:0.00000100)0.3352:0.00000100,ERR4188249:0.00000100)0.3350:0.00000100,ERR516
2136:0.00000100)0.3348:0.00000100,ERR4188145:0.00000100)0.3346:0.00000100,ERR346856
5:0.00000100)0.3344:0.00000100,ERR4187732:0.00000100)0.3342:0.00000100,ERR4188428:0.
00000100)0.3339:0.00000100,ERR3468421:0.00000100)0.3337:0.00000100,ERR4188007:0.000
00100)0.3335:0.00000100,ERR4187617:0.00000100)0.3333:0.00000100,ERR3468592:0.000001
00)0.3331:0.00000100,ERR3468661:0.00000100)0.3329:0.00000100,ERR4187792:0.00000100)
0.3327:0.00000100,ERR3439591:0.00000100)0.3325:0.00000100,ERR4188153:0.00000100)0.3
323:0.00000100,ERR3439230:0.00000100)0.3321:0.00000100,ERR5162135:0.00000100)0.3319
:0.00000100,ERR5162111:0.00000100)0.3317:0.00000100,ERR3439408:0.00000100)0.3315:0.0
0000100,ERR4188269:0.00000100)0.3313:0.00000100,ERR4188378:0.00000100)0.3311:0.0000
0100,ERR4188310:0.00000100)0.3309:0.00000100,ERR3468506:0.00000100)0.3307:0.0000010
0,ERR4188026:0.00000100)0.3305:0.00000100,ERR3439455:0.00000100)0.3303:0.00000100,E

RR3468750:0.00000100)0.3301:0.00000100,ERR3468480:0.00000100)0.3299:0.00000100,ERR5162067:0.00000100)0.3297:0.00000100,ERR3468435:0.00010800)0.3295:0.00000100,ERR3439499:0.00000100)0.3293:0.00000100,ERR3439487:0.00000100)0.3291:0.00000100,ERR4187981:0.00000100)0.3289:0.00000100,ERR5162085:0.00000100)0.3287:0.00000100,ERR3468527:0.00000100)0.3285:0.00000100,ERR4188364:0.00000100)0.3282:0.00000100,ERR4187838:0.00000100)0.3281:0.00000100,(ERR3439502:0.00000100,ERR3439377:0.00000100)0.2167:0.00010800)0.3277:0.00000100,ERR4187768:0.00000100)0.3275:0.00000100,ERR4187625:0.00000100)0.3273:0.00000100,ERR3439238:0.00000100)0.3271:0.00000100,ERR5162104:0.00000100)0.3269:0.00000100,ERR3439528:0.00000100)0.3267:0.00000100,ERR4188232:0.00000100)0.3265:0.00000100,ERR3468697:0.00000100)0.3263:0.00000100,ERR3439232:0.00000100)0.3261:0.00000100,ERR4187934:0.00000100)0.3259:0.00000100,ERR4187612:0.00000100)0.3257:0.00000100,ERR3468491:0.00010800)0.3255:0.00000100,ERR3468625:0.00010800)0.3253:0.00000100,ERR3439485:0.00000100)0.3251:0.00000100,ERR3439426:0.00010800)0.3250:0.00000100,ERR4187830:0.00000100)0.3248:0.00000100,ERR4187927:0.00000100)0.3246:0.00000100,ERR4187900:0.00000100)0.3244:0.00000100,ERR4188056:0.00000100)0.3242:0.00000100,ERR3439428:0.00000100)0.3240:0.00000100,ERR5162145:0.00000100)0.3238:0.00000100,ERR3439415:0.00000100)0.3236:0.00000100,ERR3439271:0.00000100)0.3234:0.00000100,ERR3468434:0.00000100)0.3232:0.00000100,ERR4188271:0.00010800)0.3230:0.00000100,ERR3468545:0.00000100)0.3228:0.00000100,(((((ERR5162116:0.00000100,ERR4188147:0.00000100)0.0667:0.00000100,ERR4188116:0.00000100)0.1183:0.00000100,ERR4188114:0.00000100)0.2111:0.00000100,ERR4188111:0.00000100)0.2600:0.00000100,ERR4188108:0.00000100)0.3027:0.00010800)0.3218:0.00000100,ERR5162108:0.00000100)0.3217:0.00000100,ERR3468554:0.00000100)0.3215:0.00000100,ERR4187640:0.00000100)0.3213:0.00000100,ERR3468582:0.00000100)0.3211:0.00000100,ERR3439389:0.00000100)0.3209:0.00000100,ERR3468664:0.00000100)0.3207:0.00000100,ERR3439330:0.00000100)0.3205:0.00000100,ERR4188042:0.00000100)0.3204:0.00000100,ERR4188185:0.00000100)0.3202:0.00000100,ERR4187686:0.00000100)0.3200:0.00000100,ERR4188302:0.00000100)0.3198:0.00000100,ERR4187901:0.00000100)0.3196:0.00000100,ERR4187988:0.00000100)0.3195:0.00000100,ERR3468531:0.00000100)0.3193:0.00000100,ERR3468427:0.00000100)0.3191:0.00000100,(ERR3468713:0.00000100,ERR3439298:0.00000100)0.2233:0.00010800)0.3188:0.00000100,ERR4188338:0.00000100)0.3186:0.00000100,ERR3439267:0.00000100)0.3184:0.00000100,ERR4188370:0.00000100)0.3182:0.00000100,ERR3439507:0.00000100)0.3180:0.00000100,ERR3439603:0.00000100)0.3179:0.00000100,ERR3468481:0.00000100)0.3177:0.00000100,ERR4187990:0.00000100)0.3175:0.00000100,ERR4187868:0.00000100)0.3173:0.00000100,ERR4188058:0.00000100)0.3171:0.00000100,(((SRR1791700:0.00010800,(SRR1792372:0.00000100,SRR1792356:0.00000100)0.2633:0.00010800)0.3017:0.00000100,SRR1791834:0.00000100)0.4189:0.00000100,SRR1791942:0.00021600)0.5250:0.00108000)0.3164:0.00000100,ERR4188074:0.00000100)0.3163:0.00000100,ERR4188357:0.00000100)0.3161:0.00000100,ERR3439360:0.00000100)0.3159:0.00000100,ERR3468748:0.00000100)0.3157:0.00000100,ERR3439535:0.00000100)0.3155:0.00000100,ERR3439359:0.00000100)0.3153:0.00000100,ERR3439404:0.00000100)0.3152:0.00000100,ERR3468472:0.00000100)0.3150:0.00000100,ERR3468641:0.00000100)0.3148:0.00000100,ERR4187676:0.00000100)0.3146:0.00000100,ERR4187633:0.00000100)0.3144:0.00000100,ERR3468681:0.00000100)0.3143:0.00000100,ERR4188354:0.00000100)0.3141:0.00000100,ERR3439314:0.00000100)0.3139:0.00000100,ERR4188183:0.00000100)0.3138:0.00000100,ERR4188256:0.00000100)0.3136:0.00000100,ERR4188293:0.

00000100)0.3134:0.00000100,ERR3439296:0.00000100)0.3132:0.00000100,ERR3439323:0.000
00100)0.3131:0.00000100,ERR5162084:0.00000100)0.3129:0.00000100,ERR4188095:0.000001
00)0.3127:0.00000100,ERR4187773:0.00000100)0.3125:0.00000100,ERR3439541:0.00000100)
0.3124:0.00000100,ERR3468450:0.00000100)0.3122:0.00000100,ERR4187864:0.00000100)0.3
120:0.00000100,ERR3468620:0.00000100)0.3119:0.00000100,ERR4188121:0.00000100)0.3117
:0.00000100,ERR3439400:0.00000100)0.3116:0.00000100,ERR4188296:0.00000100)0.3114:0.0
0000100,ERR3439456:0.00000100)0.3112:0.00000100,ERR4187950:0.00000100)0.3111:0.0000
0100,ERR3439327:0.00000100)0.3109:0.00000100,ERR4188152:0.00000100)0.3107:0.0000010
0,ERR4188235:0.00000100)0.3106:0.00000100,ERR3439593:0.00000100)0.3104:0.00000100,E
RR4188032:0.00000100)0.3102:0.00000100,ERR4188068:0.00000100)0.3101:0.00000100,ERR4
188408:0.00000100)0.3099:0.00000100,ERR5162139:0.00000100)0.3097:0.00000100,ERR3468
497:0.00000100)0.3096:0.00000100,ERR4188034:0.00000100)0.3094:0.00000100,ERR4188332
:0.00000100)0.3092:0.00000100,ERR3439399:0.00000100)0.3091:0.00000100,ERR4188244:0.0
0000100)0.3089:0.00000100,ERR5162064:0.00000100)0.3087:0.00000100,ERR3468423:0.0000
0100)0.3086:0.00000100,SRR15296929:0.00043200)0.3084:0.00000100,ERR5162120:0.000001
00)0.3083:0.00000100,ERR4187657:0.00000100)0.3081:0.00000100,ERR4187941:0.00000100)
0.3080:0.00000100,ERR3468439:0.00000100)0.3078:0.00000100,((ERR4187824:0.00000100,ER
R3468686:0.00000100)0.1300:0.00000100,ERR4187884:0.00000100)0.2450:0.00010800)0.307
4:0.00000100,ERR4187660:0.00000100)0.3072:0.00000100,ERR4188336:0.00000100)0.3070:0.
00000100,ERR5162101:0.00000100)0.3069:0.00000100,ERR4188341:0.00000100)0.3067:0.000
00100,ERR4187831:0.00000100)0.3065:0.00000100,ERR4188131:0.00000100)0.3064:0.000001
00,ERR3468457:0.00000100)0.3062:0.00000100,ERR4187608:0.00000100)0.3061:0.00000100,
ERR3468689:0.00000100)0.3059:0.00000100,ERR3439325:0.00000100)0.3057:0.00000100,ERR
3439605:0.00000100)0.3056:0.00000100,ERR4187798:0.00000100)0.3054:0.00000100,ERR343
9570:0.00000100)0.3053:0.00000100,ERR3439599:0.00000100)0.3051:0.00000100,ERR343930
9:0.00000100)0.3049:0.00000100,ERR3439243:0.00000100)0.3048:0.00000100,ERR3468712:0.
00000100)0.3046:0.00000100,(ERR4187960:0.00000100,ERR4187931:0.00000100)0.2233:0.00
010800)0.3043:0.00000100,ERR3439257:0.00000100)0.3042:0.00000100,ERR4187616:0.00000
100)0.3040:0.00000100,ERR4188039:0.00000100)0.3039:0.00000100,ERR4187825:0.00000100
)0.3037:0.00000100,ERR3439353:0.00000100)0.3036:0.00000100,ERR3439365:0.00000100)0.3
034:0.00000100,ERR5162072:0.00000100)0.3033:0.00000100,ERR4188093:0.00000100)0.3031
:0.00000100,ERR3439315:0.00000100)0.3029:0.00000100,ERR3468500:0.00010800)0.3028:0.0
0000100,ERR4188359:0.00000100)0.3027:0.00000100,ERR4187861:0.00000100)0.3025:0.0000
0100,ERR4187757:0.00000100)0.3024:0.00000100,ERR3439469:0.00000100)0.3023:0.0000010
0,ERR3468711:0.00000100)0.3021:0.00000100,ERR4187781:0.00000100)0.3020:0.00000100,E
RR5162117:0.00000100)0.3018:0.00000100,ERR3468539:0.00000100)0.3016:0.00000100,ERR4
187986:0.00000100)0.3015:0.00000100,ERR3439375:0.00000100)0.3013:0.00000100,ERR3468
499:0.00000100)0.3012:0.00000100,ERR4187851:0.00000100)0.3010:0.00000100,ERR4188319
:0.00000100)0.3009:0.00000100,ERR3468534:0.00000100)0.3008:0.00000100,ERR4188169:0.0
0000100)0.3006:0.00000100,ERR4188273:0.00000100)0.3005:0.00000100,ERR3439548:0.0000
0100)0.3003:0.00000100,ERR3439521:0.00000100)0.3002:0.00000100,ERR3439414:0.0000010
0)0.3000:0.00000100,ERR3439542:0.00000100)0.2999:0.00000100,ERR3439577:0.00000100)0.
2997:0.00000100,ERR4187898:0.00000100)0.2996:0.00000100,ERR3468489:0.00000100)0.299
4:0.00000100,ERR4188304:0.00000100)0.2993:0.00000100,ERR3439421:0.00000100)0.2992:0.

00000100,ERR3439483:0.00000100)0.2990:0.00000100,ERR4187983:0.00010800)0.2989:0.000
00100,ERR4187951:0.00000100)0.2987:0.00000100,ERR3468487:0.00000100)0.2986:0.000001
00,ERR4188106:0.00000100)0.2985:0.00000100,ERR3439357:0.00000100)0.2983:0.00000100,
ERR4187858:0.00000100)0.2982:0.00000100,ERR4187972:0.00000100)0.2981:0.00000100,ERR
046748:0.00000100)0.2979:0.00000100,ERR3468595:0.00000100)0.2978:0.00000100,ERR3468
456:0.00000100)0.2976:0.00000100,ERR4187948:0.00000100)0.2975:0.00000100,ERR4188176
:0.00000100)0.2973:0.00000100,ERR3439261:0.00000100)0.2972:0.00000100,ERR4187904:0.0
0000100)0.2970:0.00000100,ERR4187766:0.00000100)0.2969:0.00000100,ERR3439272:0.0000
0100)0.2968:0.00000100,ERR5162079:0.00000100)0.2966:0.00000100,ERR5087442:0.0000010
0)0.2965:0.00000100,ERR3468652:0.00000100)0.2964:0.00000100,ERR4188118:0.00000100)0.
2962:0.00000100,ERR4188220:0.00000100)0.2961:0.00000100,ERR4187725:0.00000100)0.295
9:0.00000100,ERR4187663:0.00000100)0.2958:0.00000100,ERR3439293:0.00000100)0.2956:0.
00000100,ERR3439328:0.00000100)0.2955:0.00000100,ERR4187722:0.00000100)0.2954:0.000
00100,ERR3468493:0.00000100)0.2952:0.00000100,ERR4188041:0.00000100)0.2951:0.000001
00,ERR5162094:0.00000100)0.2950:0.00000100,ERR4188190:0.00000100)0.2948:0.00000100,
ERR3468426:0.00000100)0.2947:0.00000100,ERR3439470:0.00000100)0.2945:0.00000100,ERR
4188340:0.00000100)0.2944:0.00000100,ERR4187998:0.00000100)0.2943:0.00000100,ERR346
8775:0.00000100)0.2941:0.00000100,ERR3468476:0.00000100)0.2940:0.00000100,ERR418834
4:0.00000100)0.2938:0.00000100,ERR3468675:0.00000100)0.2937:0.00000100,ERR4188073:0.
00000100)0.2935:0.00000100,ERR3439233:0.00000100)0.2934:0.00000100,ERR5162049:0.000
00100)0.2933:0.00000100,ERR3468440:0.00000100)0.2931:0.00000100,ERR3439498:0.000001
00)0.2930:0.00000100,ERR4188054:0.00000100)0.2929:0.00000100,ERR4188224:0.00000100)
0.2927:0.00000100,ERR3439287:0.00000100)0.2926:0.00000100,ERR5162138:0.00000100)0.2
925:0.00000100,ERR4187672:0.00000100)0.2923:0.00000100,ERR3439556:0.00000100)0.2922
:0.00000100,ERR4188193:0.00000100)0.2921:0.00000100,ERR3439457:0.00000100)0.2920:0.0
0000100,ERR4187874:0.00000100)0.2918:0.00000100,ERR4188367:0.00000100)0.2917:0.0000
0100,ERR4188253:0.00000100)0.2916:0.00000100,ERR5162123:0.00000100)0.2914:0.0000010
0,ERR4187662:0.00000100)0.2913:0.00000100,ERR4187647:0.00000100)0.2912:0.00000100,E
RR3468594:0.00000100)0.2911:0.00000100,ERR5162093:0.00000100)0.2910:0.00000100,ERR3
439561:0.00000100)0.2908:0.00000100,ERR5162086:0.00000100)0.2907:0.00000100,ERR5162
100:0.00000100)0.2906:0.00000100,ERR3439352:0.00000100)0.2905:0.00000100,ERR4187815
:0.00000100)0.2903:0.00000100,ERR3468729:0.00000100)0.2902:0.00000100,ERR3468536:0.0
0000100)0.2901:0.00000100,ERR3439575:0.00000100)0.2900:0.00000100,ERR4188433:0.0000
0100)0.2899:0.00000100,ERR3468425:0.00010800)0.2898:0.00000100,ERR3439337:0.0000010
0)0.2896:0.00000100,ERR4187827:0.00000100)0.2895:0.00000100,ERR4187908:0.00000100)0.
2894:0.00000100,ERR3439283:0.00000100)0.2893:0.00000100,ERR3468730:0.00000100)0.289
2:0.00000100,ERR4187855:0.00010800)0.2891:0.00000100,ERR3439482:0.00000100)0.2890:0.
00000100,ERR3468603:0.00000100)0.2888:0.00000100,ERR3468546:0.00000100)0.2887:0.000
00100,ERR4187653:0.00000100)0.2886:0.00000100,ERR4188291:0.00000100)0.2885:0.000001
00,ERR4188046:0.00000100)0.2884:0.00000100,ERR4187811:0.00000100)0.2883:0.00000100,
ERR4188012:0.00000100)0.2881:0.00000100,ERR5162088:0.00000100)0.2880:0.00000100,ERR
3439361:0.00000100)0.2879:0.00000100,ERR3468575:0.00000100)0.2878:0.00000100,ERR343
9545:0.00000100)0.2877:0.00000100,ERR4187702:0.00000100)0.2876:0.00000100,ERR418810
0:0.00000100)0.2875:0.00000100)0.2873:0.00000100,ERR4187750:0.00000100)0.2871:0.0000

0100,ERR4188184:0.00000100)0.2871:0.00000100,ERR4187637:0.00000100)0.2870:0.00000100,ERR4187770:0.00000100)0.2869:0.00000100,ERR4187748:0.00000100)0.2868:0.00000100,ERR4187813:0.00000100)0.2867:0.00000100,ERR3468632:0.00000100)0.2866:0.00000100,ERR4188345:0.00000100)0.2865:0.00000100,ERR3439571:0.00000100)0.2864:0.00000100,ERR3439585:0.00000100)0.2863:0.00000100,ERR3439460:0.00000100)0.2862:0.00000100,ERR3439234:0.00000100)0.2861:0.00000100,ERR4188343:0.00000100)0.2860:0.00000100,ERR3439519:0.00000100)0.2859:0.00000100,ERR3439594:0.00000100)0.2858:0.00000100,ERR3439409:0.00000100)0.2857:0.00000100,ERR3468696:0.00000100)0.2856:0.00000100,ERR4187847:0.00000100)0.2855:0.00000100,ERR3439524:0.00000100)0.2854:0.00000100,ERR4188339:0.00000100)0.2853:0.00000100,ERR4187704:0.00000100)0.2852:0.00000100,ERR4187805:0.00000100)0.2851:0.00000100,ERR3439544:0.00000100)0.2850:0.00000100,ERR3468473:0.00000100)0.2850:0.00000100,ERR4188382:0.00000100)0.2849:0.00000100,ERR3468672:0.00010800)0.2848:0.00000100,ERR4187706:0.00000100)0.2847:0.00000100,ERR4188097:0.00000100)0.2846:0.00000100,ERR3439358:0.00000100)0.2846:0.00000100,ERR3468791:0.00000100)0.2845:0.00000100,ERR3468441:0.00000100)0.2844:0.00000100,ERR3439410:0.00000100)0.2843:0.00000100,ERR3439543:0.00000100)0.2842:0.00000100,ERR4187775:0.00000100)0.2841:0.00000100,ERR4188038:0.00000100)0.2841:0.00000100,ERR5162078:0.00000100)0.2840:0.00000100,ERR3468737:0.00000100)0.2839:0.00000100,ERR4187656:0.00000100)0.2838:0.00000100,ERR4187996:0.00000100)0.2837:0.00000100,ERR3468743:0.00000100)0.2836:0.00000100,ERR4188198:0.00000100)0.2836:0.00000100,ERR046747:0.00000100)0.2835:0.00000100,ERR4188126:0.00000100)0.2834:0.00000100,ERR3439351:0.00000100)0.2833:0.00000100,ERR4187922:0.00000100)0.2832:0.00000100,ERR3439394:0.00000100)0.2832:0.00000100,ERR3468477:0.00000100)0.2831:0.00000100,ERR5162076:0.00000100)0.2830:0.00000100,ERR4187659:0.00000100)0.2829:0.00000100,ERR3468475:0.00000100)0.2829:0.00000100,ERR4188099:0.00000100)0.2828:0.00000100,ERR4187788:0.00000100)0.2827:0.00000100,ERR3439368:0.00010800)0.2826:0.00000100,ERR4187648:0.00000100)0.2826:0.00000100,ERR4188088:0.00000100)0.2825:0.00000100,ERR4188406:0.00000100)0.2824:0.00000100,ERR4188087:0.00000100)0.2823:0.00000100,ERR5162087:0.00000100)0.2823:0.00000100,ERR3439438:0.00000100)0.2822:0.00000100,((ERR3439497:0.00000100,ERR4187801:0.00000100)0.2400:0.00000100,ERR4188286:0.00000100)0.4450:0.00032400)0.2821:0.00000100,ERR4187842:0.00000100)0.2820:0.00000100,ERR3468471:0.00000100)0.2820:0.00000100,ERR4187926:0.00000100)0.2819:0.00000100,ERR3439367:0.00000100)0.2818:0.00000100,ERR4187977:0.00000100)0.2818:0.00000100,ERR4188384:0.00000100)0.2817:0.00000100,ERR3468763:0.00000100)0.2816:0.00000100,ERR3468559:0.00000100)0.2816:0.00000100,ERR3468676:0.00000100)0.2815:0.00000100,ERR3439569:0.00000100)0.2815:0.00000100,ERR3439247:0.00000100)0.2814:0.00000100,ERR4187765:0.00000100)0.2814:0.00000100,ERR4188363:0.00000100)0.2813:0.00000100,ERR4187760:0.00000100)0.2813:0.00000100,ERR3468694:0.00000100)0.2812:0.00000100,ERR046749:0.00000100)0.2812:0.00000100,ERR3439396:0.00000100)0.2811:0.00000100,ERR5162044:0.00000100)0.2810:0.00000100,ERR3439558:0.00000100)0.2810:0.00000100,ERR3468436:0.00000100)0.2809:0.00000100,ERR4188385:0.00000100)0.2809:0.00000100,ERR4187918:0.00000100)0.2808:0.00000100,ERR4188015:0.00000100)0.2808:0.00000100,ERR4188245:0.00000100)0.2807:0.00000100,ERR4188334:0.00000100)0.2807:0.00000100,ERR3439403:0.00000100)0.2806:0.00000100,ERR5162121:0.00000100)0.2806:0.00000100,ERR3468478:0.00000100)0.2805:0.00000100,ERR3439596:0.00000100)0.2805:0.00000100,ERR4187683:0.00000100)0.2805:0.00000100,ERR3439587:0.0000

0100)0.2804:0.00000100,ERR3439496:0.00000100)0.2804:0.00000100,ERR3439379:0.0000010
0)0.2804:0.00000100,ERR4188272:0.00000100)0.2803:0.00000100,ERR3468723:0.00000100)0.
2803:0.00000100,ERR4188270:0.00000100)0.2803:0.00000100,ERR3439248:0.00000100)0.280
2:0.00000100,ERR4188237:0.00000100)0.2802:0.00000100,ERR3439572:0.00000100)0.2802:0.
00000100,ERR5162075:0.00000100)0.2801:0.00000100,ERR3468461:0.00000100)0.2801:0.000
00100,ERR4188380:0.00000100)0.2801:0.00000100,ERR4187856:0.00000100)0.2800:0.000001
00,ERR3439224:0.00000100)0.2800:0.00000100,ERR4188047:0.00000100)0.2799:0.00000100,
ERR3468746:0.00000100)0.2799:0.00000100,ERR3439506:0.00000100)0.2799:0.00000100,ERR
3439567:0.00000100)0.2798:0.00000100,ERR4188368:0.00000100)0.2798:0.00000100,ERR516
2103:0.00000100)0.2798:0.00000100,ERR4188069:0.00000100)0.2798:0.00000100,ERR418841
1:0.00000100)0.2797:0.00000100,ERR4187678:0.00000100)0.2797:0.00000100,ERR3468709:0.
00000100)0.2797:0.00000100,ERR3439478:0.00000100)0.2796:0.00000100,ERR4187711:0.000
00100)0.2796:0.00000100,ERR3468741:0.00000100)0.2796:0.00000100,ERR3468691:0.000001
00)0.2796:0.00000100,ERR4188409:0.00000100)0.2796:0.00000100,ERR3439563:0.00000100)
0.2795:0.00000100,ERR3439583:0.00000100)0.2795:0.00000100,ERR3439262:0.00000100)0.2
795:0.00000100,ERR4188137:0.00000100)0.2795:0.00000100,ERR3439289:0.00000100)0.2794
:0.00000100,ERR4188399:0.00000100)0.2794:0.00000100,ERR3468698:0.00010800)0.2794:0.0
0000100,ERR4187991:0.00000100)0.2794:0.00000100,ERR3439529:0.00000100)0.2794:0.0000
0100,ERR4187738:0.00000100)0.2794:0.00000100,ERR3439418:0.00010800)0.2794:0.0000010
0,ERR3439595:0.00000100)0.2793:0.00000100,ERR4188003:0.00000100)0.2793:0.00000100,E
RR4187609:0.00000100)0.2793:0.00000100,ERR3439549:0.00000100)0.2793:0.00000100,ERR3
468715:0.00000100)0.2793:0.00000100,ERR3439601:0.00000100)0.2792:0.00000100,ERR4188
223:0.00000100)0.2792:0.00000100,ERR4187605:0.00000100)0.2792:0.00000100,ERR4187763
:0.00000100)0.2792:0.00000100,ERR4188150:0.00000100)0.2791:0.00000100,ERR4187909:0.0
0000100)0.2791:0.00000100,ERR5162063:0.00000100)0.2791:0.00000100,ERR4188022:0.0000
0100)0.2791:0.00000100,ERR3439304:0.00000100)0.2791:0.00000100,ERR3468610:0.0000010
0)0.2791:0.00000100,ERR4188168:0.00000100)0.2790:0.00000100,ERR3439370:0.00000100)0.
2790:0.00000100,ERR3439559:0.00000100)0.2790:0.00000100,ERR3468492:0.00000100)0.279
0:0.00000100,ERR3439491:0.00000100)0.2790:0.00000100,ERR3439523:0.00000100)0.2790:0.
00000100,ERR4187860:0.00000100)0.2790:0.00000100,ERR3439380:0.00000100)0.2789:0.000
00100,ERR3468502:0.00000100)0.2789:0.00000100,ERR4187994:0.00000100)0.2789:0.000001
00,ERR3439303:0.00000100)0.2789:0.00000100,ERR4188369:0.00000100)0.2789:0.00000100,
ERR3439286:0.00000100)0.2788:0.00000100,ERR4187975:0.00000100)0.2788:0.00000100,ERR
3468658:0.00000100)0.2788:0.00000100,ERR5162098:0.00000100)0.2788:0.00000100,ERR418
7949:0.00000100)0.2788:0.00000100,ERR3468579:0.00000100)0.2788:0.00000100,ERR516214
4:0.00000100)0.2788:0.00000100,ERR4187987:0.00000100)0.2788:0.00000100,ERR3439402:0.
00000100)0.2788:0.00000100,ERR4188325:0.00000100)0.2788:0.00000100,ERR4188044:0.000
00100)0.2788:0.00000100,ERR4187843:0.00000100)0.2788:0.00000100,ERR4188389:0.000001
00)0.2788:0.00000100,ERR4188405:0.00000100)0.2788:0.00000100,ERR4187717:0.00000100)
0.2788:0.00000100,ERR4187866:0.00000100)0.2788:0.00000100,ERR3439530:0.00000100)0.2
788:0.00000100,ERR3468542:0.00000100)0.2788:0.00000100,ERR4187666:0.00000100)0.2788
:0.00000100,ERR3468448:0.00000100)0.2788:0.00000100,ERR3468688:0.00010800)0.2788:0.0
0000100,ERR4188236:0.00000100)0.2788:0.00000100,ERR3439458:0.00000100)0.2788:0.0000
0100,ERR3468460:0.00000100)0.2788:0.00000100,(ERR3439335:0.00000100,ERR3439371:0.00

000100)0.2333:0.00010800)0.2788:0.00000100,ERR3468747:0.00000100)0.2788:0.00000100,E
RR3439275:0.00000100)0.2788:0.00000100,ERR4188277:0.00000100)0.2788:0.00000100,ERR3
439295:0.00000100)0.2788:0.00000100,ERR3468523:0.00000100)0.2788:0.00000100,ERR4188
318:0.00000100)0.2788:0.00000100,ERR3468623:0.00000100)0.2788:0.00000100,ERR4188432
:0.00010800)0.2788:0.00000100,ERR4188123:0.00000100)0.2788:0.00000100,ERR3439550:0.0
0000100)0.2788:0.00000100,ERR3439579:0.00000100)0.2789:0.00000100,SRR15296939:0.000
10800)0.2788:0.00000100,ERR4188120:0.00000100)0.2789:0.00000100,ERR4187879:0.000001
00)0.2789:0.00000100,ERR4188064:0.00000100)0.2789:0.00000100,ERR4188430:0.00000100)
0.2789:0.00000100,ERR4187700:0.00000100)0.2789:0.00000100,ERR4188349:0.00000100)0.2
789:0.00000100,ERR4187944:0.00000100)0.2789:0.00000100,ERR3439584:0.00000100)0.2789
:0.00000100,ERR4188031:0.00000100)0.2790:0.00000100,ERR4187808:0.00000100)0.2790:0.0
0000100,ERR4188288:0.00000100)0.2790:0.00000100,ERR4187930:0.00000100)0.2790:0.0000
0100,ERR3468449:0.00000100)0.2790:0.00000100,ERR4187845:0.00000100)0.2790:0.0000010
0,ERR3439225:0.00000100)0.2790:0.00000100,ERR3439313:0.00000100)0.2790:0.00000100,E
RR5162115:0.00010800)0.2791:0.00000100,ERR3439580:0.00000100)0.2791:0.00000100,ERR3
439436:0.00000100)0.2791:0.00000100,ERR3439241:0.00000100)0.2791:0.00000100,ERR4187
778:0.00000100)0.2791:0.00000100,ERR4187742:0.00000100)0.2791:0.00000100,ERR5162082
:0.00000100)0.2791:0.00000100,ERR3439372:0.00000100)0.2792:0.00000100,ERR4188195:0.0
0000100)0.2792:0.00000100,ERR4188078:0.00000100)0.2792:0.00000100,ERR4187920:0.0000
0100)0.2792:0.00000100,ERR3439437:0.00000100)0.2792:0.00000100,ERR3468451:0.0000010
0)0.2793:0.00000100,ERR3439516:0.00000100)0.2793:0.00000100,ERR3468511:0.00000100)0.
2793:0.00000100,ERR4187665:0.00000100)0.2793:0.00000100,ERR3439515:0.00000100)0.279
4:0.00000100,ERR3439244:0.00000100)0.2794:0.00000100,ERR4188125:0.00000100)0.2794:0.
00000100,ERR3468674:0.00000100)0.2794:0.00000100,ERR3468510:0.00000100)0.2795:0.000
00100,ERR3439264:0.00000100)0.2795:0.00000100,ERR4188035:0.00000100)0.2795:0.000001
00,ERR4187836:0.00000100)0.2795:0.00000100,ERR3439471:0.00000100)0.2796:0.00000100,
ERR4187694:0.00000100)0.2797:0.00000100,ERR4187875:0.00000100)0.2797:0.00000100,ERR
4187649:0.00000100)0.2797:0.00000100,ERR046954:0.00000100)0.2797:0.00000100,ERR3439
341:0.00000100)0.2797:0.00000100,ERR4187629:0.00000100)0.2798:0.00000100,ERR3468464
:0.00000100)0.2798:0.00000100,ERR4188000:0.00000100)0.2798:0.00000100,ERR046961:0.00
010800)0.2799:0.00000100,ERR4188250:0.00000100)0.2799:0.00000100,ERR3468606:0.00000
100)0.2799:0.00000100,ERR3468717:0.00000100)0.2800:0.00000100,ERR4188117:0.00000100
)0.2800:0.00000100,ERR4188311:0.00000100)0.2800:0.00000100,ERR4187707:0.00000100)0.2
801:0.00000100,ERR3439565:0.00000100)0.2801:0.00000100,ERR3468720:0.00000100)0.2801
:0.00000100,ERR3468518:0.00000100)0.2802:0.00000100,ERR4188267:0.00000100)0.2802:0.0
0000100,ERR3439239:0.00000100)0.2802:0.00000100,ERR3468462:0.00000100)0.2803:0.0000
0100,ERR3468495:0.00000100)0.2803:0.00000100,ERR3439364:0.00000100)0.2803:0.0000010
0,ERR4187810:0.00000100)0.2804:0.00000100,ERR4187611:0.00000100)0.2804:0.00000100,E
RR3439537:0.00000100)0.2804:0.00000100,ERR4188164:0.00000100)0.2805:0.00000100,ERR3
468668:0.00000100)0.2805:0.00000100,ERR3439604:0.00000100)0.2805:0.00000100,ERR5162
143:0.00000100)0.2806:0.00000100,ERR4188376:0.00000100)0.2806:0.00000100,ERR3439592
:0.00000100)0.2807:0.00000100,ERR4187895:0.00000100)0.2807:0.00000100,ERR3439420:0.0
0000100)0.2807:0.00000100,ERR4187681:0.00000100)0.2808:0.00000100,ERR4188261:0.0000
0100)0.2808:0.00000100,ERR3439479:0.00000100)0.2809:0.00000100,ERR4187896:0.0000010

0)0.2809:0.00000100,ERR3439539:0.00000100)0.2810:0.00000100,ERR3468736:0.00000100)0.
2810:0.00000100,ERR4187876:0.00000100)0.2810:0.00000100,ERR4187822:0.00000100)0.281
1:0.00000100,ERR4187615:0.00000100)0.2811:0.00000100,ERR3439376:0.00000100)0.2812:0.
00000100,ERR4188305:0.00000100)0.2812:0.00000100,ERR3439382:0.00000100)0.2813:0.000
00100,ERR3439299:0.00000100)0.2813:0.00000100,ERR3468515:0.00000100)0.2814:0.000001
00,ERR4187821:0.00000100)0.2814:0.00000100,ERR4187828:0.00000100)0.2814:0.00000100,
ERR3439260:0.00000100)0.2815:0.00000100,ERR5162089:0.00000100)0.2815:0.00000100,ERR
3439307:0.00000100)0.2815:0.00000100,ERR5162059:0.00000100)0.2816:0.00000100,ERR418
8211:0.00000100)0.2816:0.00000100,ERR4188214:0.00000100)0.2817:0.00000100,ERR418800
1:0.00000100)0.2818:0.00000100,ERR3439531:0.00000100)0.2818:0.00000100,ERR3468728:0.
00000100)0.2818:0.00000100,ERR4187932:0.00000100)0.2819:0.00000100,ERR4187741:0.000
00100)0.2820:0.00000100,ERR3439431:0.00000100)0.2820:0.00000100,ERR4188175:0.000001
00)0.2821:0.00000100,ERR4188139:0.00000100)0.2822:0.00000100,ERR4188276:0.00000100)
0.2822:0.00000100,ERR3439340:0.00000100)0.2823:0.00000100,ERR3468442:0.00000100)0.2
824:0.00000100,ERR3468569:0.00000100)0.2824:0.00000100,ERR3468572:0.00000100)0.2825
:0.00000100,ERR5162047:0.00000100)0.2826:0.00000100,ERR3439466:0.00000100)0.2827:0.0
0000100,ERR4188425:0.00000100)0.2828:0.00000100,ERR3439454:0.00000100)0.2829:0.0000
0100,ERR4187606:0.00000100)0.2829:0.00000100,ERR3439600:0.00000100)0.2830:0.0000010
0,ERR4187923:0.00000100)0.2830:0.00000100,ERR3468520:0.00000100)0.2831:0.00000100,E
RR4188157:0.00000100)0.2831:0.00000100,ERR4188109:0.00000100)0.2832:0.00000100,ERR3
439568:0.00000100)0.2833:0.00000100,ERR5162083:0.00000100)0.2834:0.00000100,ERR4187
723:0.00000100)0.2834:0.00000100,ERR3468509:0.00000100)0.2835:0.00000100,ERR3468463
:0.00000100)0.2836:0.00000100,ERR4188347:0.00000100)0.2836:0.00000100,ERR3468768:0.0
0000100)0.2837:0.00000100,ERR4187787:0.00000100)0.2838:0.00000100,ERR4188002:0.0000
0100)0.2838:0.00000100,ERR4187644:0.00000100)0.2839:0.00000100,ERR3439250:0.0000010
0)0.2839:0.00000100,ERR3439573:0.00000100)0.2840:0.00000100,ERR3468761:0.00000100)0.
2840:0.00000100,ERR4188051:0.00000100)0.2841:0.00000100,(ERR4188312:0.00000100,ERR3
439424:0.00000100)0.2467:0.00010800)0.2842:0.00000100,ERR3439518:0.00000100)0.2843:0
.00000100,ERR3468731:0.00000100)0.2844:0.00000100,ERR4188242:0.00000100)0.2844:0.00
000100,ERR4188434:0.00000100)0.2845:0.00000100,ERR3468660:0.00000100)0.2846:0.00000
100,ERR4187979:0.00000100)0.2846:0.00000100,ERR4187886:0.00000100)0.2847:0.00000100
,ERR3468562:0.00000100)0.2848:0.00000100,ERR3468474:0.00000100)0.2849:0.00000100,ER
R3468438:0.00000100)0.2849:0.00000100,ERR4188134:0.00000100)0.2850:0.00000100,ERR41
88306:0.00000100)0.2851:0.00000100,ERR3468571:0.00000100)0.2852:0.00000100,ERR41876
89:0.00000100)0.2853:0.00000100,ERR4188316:0.00000100)0.2854:0.00000100,ERR3468547:
0.00000100)0.2854:0.00000100,(ERR3439240:0.00000100,ERR3439251:0.00000100)0.2300:0.0
0010800)0.2856:0.00000100,ERR4187814:0.00000100)0.2857:0.00000100,ERR3468783:0.0000
0100)0.2858:0.00000100,ERR3468437:0.00000100)0.2859:0.00000100,ERR3439468:0.0000010
0)0.2859:0.00000100,ERR4188205:0.00000100)0.2860:0.00000100,ERR3468548:0.00000100)0.
2861:0.00000100,ERR4187697:0.00000100)0.2862:0.00000100,ERR4188259:0.00000100)0.286
3:0.00000100,ERR3439472:0.00000100)0.2864:0.00000100,ERR3439319:0.00000100)0.2865:0.
00000100,ERR3468494:0.00010800)0.2866:0.00000100,ERR5087443:0.00000100)0.2866:0.000
00100,ERR3468617:0.00000100)0.2867:0.00000100,ERR3439288:0.00000100)0.2868:0.000001
00,ERR3439222:0.00000100)0.2869:0.00000100,ERR4187799:0.00000100)0.2870:0.00000100,

ERR3439465:0.00000100)0.2870:0.00000100,ERR4187915:0.00000100)0.2871:0.00000100,ERR
4188070:0.00000100)0.2872:0.00000100,ERR4188228:0.00000100)0.2873:0.00000100,ERR343
9413:0.00000100)0.2874:0.00000100,ERR3439590:0.00000100)0.2875:0.00000100,ERR516213
7:0.00000100)0.2875:0.00000100,ERR3468568:0.00000100)0.2877:0.00000100,ERR3439366:0.
00000100)0.2878:0.00000100,ERR4188101:0.00000100)0.2879:0.00000100,ERR5162056:0.000
00100)0.2880:0.00000100,ERR3439412:0.00000100)0.2881:0.00000100,ERR4188266:0.000001
00)0.2882:0.00000100,ERR3439226:0.00000100)0.2883:0.00000100,ERR3468751:0.00000100)
0.2884:0.00000100,ERR3439263:0.00000100)0.2885:0.00000100,ERR3468455:0.00000100)0.2
886:0.00000100,ERR3468567:0.00000100)0.2886:0.00000100,ERR4188333:0.00043200)0.2887
:0.00000100,ERR3468429:0.00000100)0.2888:0.00000100,ERR4188335:0.00000100)0.2889:0.0
0000100,ERR4187721:0.00000100)0.2890:0.00000100,ERR3468576:0.00000100)0.2891:0.0000
0100,ERR4188328:0.00000100)0.2892:0.00000100,ERR4187832:0.00000100)0.2892:0.0000010
0,ERR4188191:0.00000100)0.2893:0.00000100,ERR3468514:0.00000100)0.2894:0.00000100,E
RR4187882:0.00000100)0.2895:0.00000100,ERR3439388:0.00000100)0.2896:0.00000100,ERR3
468526:0.00000100)0.2897:0.00000100,ERR3468444:0.00000100)0.2898:0.00000100,ERR4187
945:0.00000100)0.2899:0.00000100,ERR5162097:0.00000100)0.2900:0.00000100,ERR3468770
:0.00000100)0.2900:0.00000100,ERR3468680:0.00000100)0.2901:0.00000100,ERR3468667:0.0
0000100)0.2902:0.00000100,ERR3468484:0.00000100)0.2903:0.00000100,ERR4187710:0.0000
0100)0.2904:0.00000100,ERR5162060:0.00010800)0.2905:0.00000100,ERR3468516:0.0000010
0)0.2906:0.00000100,ERR3439554:0.00000100)0.2907:0.00000100,ERR3468454:0.00000100)0.
2908:0.00000100,ERR4188016:0.00000100)0.2909:0.00000100,ERR4187958:0.00000100)0.291
0:0.00000100,ERR4188090:0.00000100)0.2912:0.00000100,ERR4188279:0.00000100)0.2913:0.
00000100,ERR4188030:0.00000100)0.2914:0.00000100,ERR3439373:0.00000100)0.2915:0.000
00100,ERR4188020:0.00000100)0.2916:0.00000100,ERR4187912:0.00000100)0.2916:0.000001
00,ERR3468540:0.00000100)0.2917:0.00000100,ERR4188194:0.00000100)0.2919:0.00000100,
ERR4187980:0.00000100)0.2920:0.00000100,ERR3468422:0.00000100)0.2921:0.00000100,ERR
4187677:0.00000100)0.2922:0.00000100,ERR5162107:0.00000100)0.2923:0.00000100,ERR418
8392:0.00000100)0.2924:0.00000100,ERR4188172:0.00000100)0.2925:0.00000100,ERR516205
4:0.00000100)0.2926:0.00000100,ERR3439434:0.00000100)0.2927:0.00000100,ERR3439242:0.
00000100)0.2928:0.00000100,ERR3439525:0.00000100)0.2929:0.00000100,ERR3439476:0.000
00100)0.2930:0.00000100,ERR4188079:0.00000100)0.2931:0.00000100,ERR4187620:0.000001
00)0.2932:0.00000100,ERR4187638:0.00010800)0.2933:0.00000100,ERR3468433:0.00000100)
0.2934:0.00000100,ERR4187739:0.00000100)0.2936:0.00000100,ERR4188401:0.00000100)0.2
937:0.00000100,ERR4188159:0.00000100)0.2938:0.00000100,ERR5162096:0.00000100)0.2939
:0.00000100,SRR17156534:0.00064800)0.2940:0.00000100,ERR3468563:0.00000100)0.2940:0.
00000100,ERR3468553:0.00000100)0.2941:0.00000100,ERR4188303:0.00000100)0.2943:0.000
00100,ERR4187687:0.00000100)0.2944:0.00000100,ERR3468570:0.00000100)0.2945:0.000001
00,ERR4188300:0.00000100)0.2946:0.00000100,ERR4187679:0.00000100)0.2947:0.00000100,
ERR3439423:0.00000100)0.2948:0.00000100,ERR3468666:0.00000100)0.2949:0.00000100,ERR
5162092:0.00000100)0.2950:0.00000100,ERR3468616:0.00000100)0.2952:0.00000100,ERR346
8580:0.00000100)0.2953:0.00000100,ERR4188085:0.00000100)0.2954:0.00000100,ERR343925
4:0.00000100)0.2955:0.00000100,ERR3468447:0.00000100)0.2956:0.00000100,ERR4188233:0.
00000100)0.2957:0.00000100,ERR5162071:0.00000100)0.2959:0.00000100,ERR3439451:0.000
00100)0.2959:0.00000100,ERR5162146:0.00000100)0.2961:0.00000100,ERR3468543:0.000001

00)0.2962:0.00000100,ERR4188136:0.00010800)0.2963:0.00000100,ERR4187818:0.00000100)
0.2965:0.00000100,ERR3439494:0.00000100)0.2966:0.00000100,ERR3439576:0.00000100)0.2
967:0.00000100,ERR3468496:0.00000100)0.2968:0.00000100,ERR3468735:0.00000100)0.2969
:0.00000100,ERR4188210:0.00000100)0.2971:0.00000100,ERR3439510:0.00000100)0.2972:0.0
0000100,ERR4187985:0.00000100)0.2974:0.00000100,ERR4188192:0.00000100)0.2975:0.0000
0100,ERR3439369:0.00010800)0.2976:0.00000100,ERR3439245:0.00000100)0.2978:0.0000010
0,ERR4188320:0.00000100)0.2979:0.00000100,ERR4188238:0.00000100)0.2980:0.00000100,E
RR4187854:0.00000100)0.2981:0.00000100,ERR3468560:0.00000100)0.2982:0.00000100,ERR3
439564:0.00000100)0.2984:0.00000100,ERR4187929:0.00000100)0.2985:0.00000100,ERR3439
439:0.00000100)0.2986:0.00000100,ERR3439386:0.00000100)0.2987:0.00000100,ERR3439310
:0.00000100)0.2989:0.00000100,ERR4188094:0.00000100)0.2990:0.00000100,ERR4188301:0.0
0000100)0.2991:0.00000100,ERR4187619:0.00000100)0.2993:0.00000100,ERR3468452:0.0000
0100)0.2994:0.00000100,ERR4188212:0.00000100)0.2995:0.00000100,ERR3439527:0.0000010
0)0.2996:0.00000100,ERR4187705:0.00000100)0.2998:0.00000100,ERR3468765:0.00000100)0.
2999:0.00000100,ERR4187961:0.00000100)0.3001:0.00000100,ERR4187992:0.00000100)0.300
2:0.00000100,ERR4188055:0.00000100)0.3004:0.00000100,ERR4188036:0.00000100)0.3005:0.
00000100,ERR4188057:0.00000100)0.3007:0.00000100,ERR4188161:0.00000100)0.3008:0.000
00100,ERR4187610:0.00000100)0.3009:0.00000100,ERR3468738:0.00000100)0.3011:0.000001
00,ERR4187736:0.00000100)0.3012:0.00000100,ERR4188309:0.00000100)0.3014:0.00000100,
ERR4188298:0.00000100)0.3015:0.00000100,ERR4187642:0.00000100)0.3017:0.00000100,ERR
3439557:0.00000100)0.3018:0.00000100,ERR3468767:0.00000100)0.3020:0.00000100,ERR343
9520:0.00000100)0.3021:0.00000100,ERR3468602:0.00000100)0.3022:0.00000100,ERR346867
3:0.00000100)0.3024:0.00000100,ERR3439586:0.00000100)0.3025:0.00000100,ERR3439555:0.
00000100)0.3027:0.00000100,ERR4188098:0.00000100)0.3028:0.00000100,ERR3439464:0.000
00100)0.3029:0.00000100,ERR4188274:0.00000100)0.3031:0.00000100,ERR4188200:0.000001
00)0.3033:0.00000100,ERR4188436:0.00000100)0.3035:0.00000100,ERR4187804:0.00000100)
0.3036:0.00000100,ERR4187968:0.00000100)0.3037:0.00000100,ERR3439345:0.00000100)0.3
039:0.00000100,ERR3439302:0.00000100)0.3040:0.00000100,ERR3468677:0.00000100)0.3042
:0.00000100,ERR4188386:0.00000100)0.3043:0.00000100,ERR4188221:0.00000100)0.3045:0.0
0000100,ERR3468424:0.00000100)0.3046:0.00000100,ERR4188148:0.00000100)0.3048:0.0000
0100,ERR5162147:0.00000100)0.3049:0.00000100,ERR4187726:0.00000100)0.3051:0.0000010
0,ERR4188061:0.00000100)0.3052:0.00000100,ERR4188292:0.00000100)0.3054:0.00000100,E
RR3439378:0.00010800)0.3055:0.00000100,ERR5162053:0.00000100)0.3057:0.00000100,ERR3
468722:0.00000100)0.3058:0.00000100,ERR3439477:0.00000100)0.3060:0.00000100,ERR4187
873:0.00000100)0.3061:0.00000100,ERR4188268:0.00000100)0.3063:0.00000100,ERR4188426
:0.00000100)0.3064:0.00000100,ERR4188379:0.00000100)0.3066:0.00000100,ERR4187966:0.0
0000100)0.3068:0.00000100,ERR3439602:0.00000100)0.3069:0.00000100,ERR5162142:0.0000
0100)0.3071:0.00000100,ERR4188105:0.00000100)0.3072:0.00000100,ERR4187925:0.0000010
0)0.3074:0.00000100,ERR4187962:0.00000100)0.3076:0.00000100,ERR3439354:0.00000100)0.
3078:0.00000100,ERR4188413:0.00000100)0.3079:0.00000100,ERR3468781:0.00010800)0.308
1:0.00000100,ERR3468613:0.00000100)0.3082:0.00000100,ERR4187844:0.00000100)0.3084:0.
00000100,ERR4187776:0.00000100)0.3086:0.00000100,ERR4187891:0.00000100)0.3087:0.000
00100,ERR4187761:0.00000100)0.3089:0.00000100,ERR4187820:0.00000100)0.3090:0.000001
00,ERR4187959:0.00000100)0.3092:0.00000100,ERR3468507:0.00000100)0.3094:0.00000100,

ERR4188294:0.00000100)0.3096:0.00000100,ERR3468774:0.00010800)0.3097:0.00000100,ERR4188129:0.00000100)0.3099:0.00000100,ERR4187943:0.00000100)0.3101:0.00000100,ERR4188128:0.00010800)0.3102:0.00000100,ERR4188421:0.00000100)0.3104:0.00000100,ERR5162119:0.00000100)0.3106:0.00000100,ERR3439473:0.00000100)0.3107:0.00000100,ERR4188124:0.00010800)0.3109:0.00000100,ERR3439252:0.00000100)0.3110:0.00000100,ERR4188122:0.00000100)0.3112:0.00000100,ERR3468649:0.00000100)0.3114:0.00000100,ERR3439581:0.00000100)0.3116:0.00000100,ERR3439422:0.00000100)0.3117:0.00000100,ERR4188186:0.00000100)0.3119:0.00000100,ERR3439348:0.00000100)0.3121:0.00000100,ERR3439385:0.00000100)0.3122:0.00000100,ERR4187924:0.00000100)0.3125:0.00000100,ERR3439492:0.00000100)0.3126:0.00000100,SRR15296932:0.00075600)0.3128:0.00000100,ERR3439269:0.00000100)0.3130:0.00000100,ERR4188295:0.00000100)0.3132:0.00000100,ERR3439355:0.00000100)0.3133:0.00000100,ERR3468479:0.00000100)0.3135:0.00000100,ERR3439582:0.00000100)0.3137:0.00000100,ERR4187833:0.00000100)0.3139:0.00000100,ERR3439513:0.00000100)0.3141:0.00000100,ERR4188115:0.00000100)0.3142:0.00000100,ERR4188197:0.00000100)0.3144:0.00000100,ERR3468512:0.00000100)0.3146:0.00000100,ERR4188066:0.00000100)0.3149:0.00000100,ERR3439273:0.00000100)0.3150:0.00000100,((ERR4188135:0.00000100,ERR4188130:0.00000100)0.1300:0.00000100,ERR4188132:0.00000100)0.3017:0.00010800)0.3156:0.00000100,ERR4187794:0.00000100)0.3158:0.00000100,ERR3468706:0.00000100)0.3159:0.00000100,ERR3468645:0.00000100)0.3161:0.00000100,ERR4188065:0.00000100)0.3163:0.00000100,ERR3468604:0.00000100)0.3165:0.00000100,ERR3468621:0.00000100)0.3166:0.00000100,ERR4188337:0.00000100)0.3168:0.00000100,ERR5162113:0.00000100)0.3170:0.00000100,ERR5162081:0.00000100)0.3172:0.00000100,ERR5162130:0.00000100)0.3174:0.00000100,ERR4187733:0.00000100)0.3175:0.00000100,ERR3468566:0.00010800)0.3177:0.00000100,ERR4188045:0.00000100)0.3179:0.00000100,ERR3439505:0.00000100)0.3181:0.00000100,ERR3468629:0.00000100)0.3183:0.00000100,ERR3468635:0.00000100)0.3184:0.00000100,ERR3439461:0.00000100)0.3186:0.00000100,ERR3439560:0.00000100)0.3188:0.00000100,ERR3468428:0.00000100)0.3190:0.00000100,ERR4187691:0.00000100)0.3192:0.00000100,ERR4187989:0.00000100)0.3194:0.00000100,ERR4188006:0.00000100)0.3196:0.00000100,ERR3468784:0.00000100)0.3198:0.00000100,ERR4188025:0.00000100)0.3200:0.00000100,ERR4187729:0.00000100)0.3202:0.00000100,ERR5162091:0.00000100)0.3204:0.00000100,ERR3468459:0.00000100)0.3206:0.00000100,ERR4188283:0.00000100)0.3208:0.00000100,ERR3439480:0.00000100)0.3210:0.00000100,ERR4187708:0.00000100)0.3212:0.00000100,ERR4187892:0.00000100)0.3213:0.00000100,ERR4187872:0.00000100)0.3215:0.00000100,ERR4187862:0.00000100)0.3217:0.00000100,ERR3468682:0.00000100)0.3219:0.00000100,ERR3439236:0.00000100)0.3221:0.00000100,ERR3439488:0.00000100)0.3223:0.00000100,ERR5162095:0.00010800)0.3225:0.00000100,ERR3468704:0.00000100)0.3227:0.00000100,ERR3468634:0.00000100)0.3230:0.00000100,ERR4188102:0.00000100)0.3231:0.00000100,ERR3468558:0.00000100)0.3233:0.00000100,ERR4188371:0.00000100)0.3235:0.00000100,ERR5162128:0.00000100)0.3237:0.00000100,ERR4187680:0.00000100)0.3239:0.00000100,ERR3439481:0.00010800)0.3241:0.00000100,ERR4188398:0.00000100)0.3243:0.00000100,ERR3468485:0.00000100)0.3245:0.00000100,ERR4188142:0.00000100)0.3247:0.00000100,ERR3439463:0.00000100)0.3249:0.00000100,ERR3468656:0.00000100)0.3251:0.00000100,ERR4187635:0.00000100)0.3253:0.00000100,ERR3439495:0.00000100)0.3255:0.00000100,ERR4188103:0.00000100)0.3257:0.00000100,ERR4188199:0.00000100)0.3259:0.00000100,ERR3439342:0.00000100)0.3261:0.00000100,ERR3439324:0.00000100)0.3263:0.00000100,ERR3468517:0.0000

0100)0.3265:0.00000100,ERR4188040:0.00000100)0.3267:0.00000100,ERR3439284:0.0000010
0)0.3269:0.00000100,ERR4188264:0.00000100)0.3271:0.00000100,ERR4188308:0.00000100)0.
3273:0.00000100,ERR4188397:0.00000100)0.3274:0.00000100,ERR5162050:0.00000100)0.327
6:0.00000100,ERR4188028:0.00000100)0.3278:0.00000100,ERR3439336:0.00000100)0.3280:0.
00000100,ERR3468749:0.00000100)0.3282:0.00000100,ERR3439405:0.00000100)0.3284:0.000
00100,ERR4187889:0.00000100)0.3286:0.00000100,ERR3439397:0.00000100)0.3288:0.000001
00,ERR3439526:0.00000100)0.3290:0.00000100,ERR4187693:0.00000100)0.3292:0.00000100,
ERR3439316:0.00000100)0.3294:0.00000100,ERR3468453:0.00000100)0.3296:0.00000100,ERR
4187837:0.00000100)0.3298:0.00000100,ERR3468788:0.00000100)0.3300:0.00000100,ERR418
8138:0.00010800)0.3302:0.00000100,ERR3439429:0.00010800)0.3304:0.00000100,ERR516212
9:0.00000100)0.3306:0.00000100,ERR4187675:0.00000100)0.3308:0.00000100,ERR3468466:0.
00000100)0.3310:0.00000100,ERR4187969:0.00000100)0.3312:0.00000100,ERR4188037:0.000
00100)0.3314:0.00000100,ERR3468573:0.00000100)0.3316:0.00000100,ERR4188174:0.000001
00)0.3318:0.00000100,ERR5162140:0.00000100)0.3320:0.00000100,ERR3439517:0.00000100)
0.3322:0.00000100,ERR4188173:0.00000100)0.3324:0.00000100,ERR4187755:0.00000100)0.3
326:0.00000100,ERR3468586:0.00000100)0.3328:0.00000100,ERR3439450:0.00010800)0.3330
:0.00000100,ERR3468431:0.00000100)0.3332:0.00000100,ERR3439430:0.00000100)0.3334:0.0
0000100,ERR3439540:0.00000100)0.3337:0.00000100,ERR4188402:0.00000100)0.3339:0.0000
0100,ERR4188427:0.00010800)0.3341:0.00000100,ERR3439332:0.00000100)0.3343:0.0000010
0,ERR5087441:0.00000100)0.3345:0.00000100,ERR3468468:0.00000100)0.3347:0.00000100,E
RR3439448:0.00000100)0.3349:0.00000100,ERR5162069:0.00000100)0.3352:0.00000100,ERR3
439344:0.00000100)0.3354:0.00000100,ERR4188162:0.00000100)0.3356:0.00000100,ERR4188
104:0.00000100)0.3358:0.00000100,ERR3439308:0.00000100)0.3360:0.00000100,ERR4188049
:0.00000100)0.3362:0.00000100,ERR4188437:0.00000100)0.3364:0.00000100,ERR3439231:0.0
0000100)0.3367:0.00000100,ERR4188077:0.00000100)0.3368:0.00000100,ERR4188112:0.0000
0100)0.3371:0.00000100,ERR4188216:0.00000100)0.3373:0.00000100,ERR3439383:0.0000010
0)0.3375:0.00000100,ERR3439392:0.00000100)0.3378:0.00000100,ERR4187797:0.00000100)0.
3380:0.00000100,ERR3439419:0.00000100)0.3382:0.00000100,ERR3468432:0.00000100)0.338
4:0.00000100,ERR3439512:0.00000100)0.3386:0.00000100,ERR4187743:0.00000100)0.3389:0.
00000100,ERR3468557:0.00000100)0.3391:0.00000100,ERR3468772:0.00000100)0.3393:0.000
00100,ERR3439321:0.00000100)0.3396:0.00000100,ERR4188177:0.00000100)0.3398:0.000001
00,ERR4188017:0.00010800)0.3400:0.00000100,ERR4187937:0.00000100)0.3402:0.00000100,
ERR3468792:0.00000100)0.3404:0.00000100,ERR4187719:0.00000100)0.3407:0.00000100,ERR
4188260:0.00000100)0.3409:0.00000100,ERR4188278:0.00000100)0.3411:0.00000100,ERR343
9278:0.00000100)0.3413:0.00000100,ERR3439320:0.00000100)0.3415:0.00000100,ERR343951
4:0.00000100)0.3418:0.00000100,ERR3439338:0.00000100)0.3420:0.00000100,ERR4187784:0.
00000100)0.3422:0.00000100,ERR4188418:0.00000100)0.3425:0.00000100,ERR3439312:0.000
00100)0.3427:0.00000100,ERR4188196:0.00000100)0.3429:0.00000100,ERR4188246:0.000001
00)0.3431:0.00000100,ERR3439532:0.00000100)0.3433:0.00000100,ERR3468707:0.00000100)
0.3436:0.00000100,ERR3468627:0.00000100)0.3438:0.00000100,ERR3468467:0.00010800)0.3
440:0.00000100,ERR4188394:0.00000100)0.3443:0.00000100,ERR3468626:0.00000100)0.3445
:0.00000100,ERR3468583:0.00000100)0.3447:0.00000100,ERR3439363:0.00000100)0.3450:0.0
0000100,ERR4187655:0.00000100)0.3452:0.00000100,ERR3439331:0.00000100)0.3454:0.0000
0100,ERR4187735:0.00000100)0.3457:0.00000100,ERR3468458:0.00000100)0.3459:0.0000010

0,ERR5162110:0.00000100)0.3461:0.00000100,ERR3439292:0.00000100)0.3464:0.00000100,E
RR3439346:0.00000100)0.3466:0.00000100,ERR4187654:0.00000100)0.3468:0.00000100,ERR4
188417:0.00000100)0.3471:0.00000100,ERR3439475:0.00000100)0.3473:0.00000100,ERR3468
760:0.00000100)0.3476:0.00000100,ERR3439318:0.00000100)0.3478:0.00000100,ERR4188407
:0.00000100)0.3480:0.00000100,ERR3439534:0.00000100)0.3483:0.00000100,ERR4187651:0.0
0000100)0.3485:0.00000100,ERR5162124:0.00000100)0.3488:0.00000100,ERR3439538:0.0001
0800)0.3490:0.00000100,ERR4188258:0.00000100)0.3492:0.00000100,ERR3468794:0.0000010
0)0.3495:0.00000100,ERR3468505:0.00000100)0.3497:0.00000100,ERR4188315:0.00000100)0.
3500:0.00000100,ERR4187913:0.00000100)0.3502:0.00000100,ERR4187928:0.00000100)0.350
5:0.00000100,ERR3439291:0.00000100)0.3507:0.00000100,ERR4187621:0.00000100)0.3509:0.
00000100,ERR3468796:0.00000100)0.3512:0.00000100,ERR3468529:0.00000100)0.3514:0.000
00100,ERR4187940:0.00000100)0.3517:0.00000100,ERR3439249:0.00000100)0.3519:0.000001
00,ERR3468678:0.00000100)0.3521:0.00000100,ERR4188081:0.00000100)0.3524:0.00000100,
ERR3439566:0.00000100)0.3527:0.00000100,ERR4188024:0.00000100)0.3529:0.00000100,ERR
4188314:0.00000100)0.3531:0.00000100,ERR4188415:0.00000100)0.3534:0.00000100,ERR346
8593:0.00000100)0.3536:0.00000100,ERR3468589:0.00000100)0.3539:0.00000100,ERR418785
3:0.00000100)0.3542:0.00000100,ERR4187863:0.00054000)0.3544:0.00000100,ERR3468651:0.
00000100)0.3546:0.00000100,ERR3439490:0.00000100)0.3549:0.00000100,ERR4187727:0.000
00100)0.3551:0.00000100,ERR4188240:0.00000100)0.3554:0.00000100,ERR4188393:0.000001
00)0.3556:0.00000100,ERR4187658:0.00000100)0.3559:0.00000100,ERR3439270:0.00000100)
0.3561:0.00000100,ERR4188281:0.00000100)0.3564:0.00000100,ERR4187695:0.00000100)0.3
566:0.00000100,ERR4188317:0.00000100)0.3569:0.00000100,ERR4187911:0.00000100)0.3571
:0.00000100,ERR4187834:0.00000100)0.3574:0.00000100,ERR4188239:0.00000100)0.3576:0.0
0000100,ERR4188165:0.00000100)0.3579:0.00000100,ERR4188346:0.00000100)0.3581:0.0000
0100,ERR4188400:0.00000100)0.3584:0.00000100,ERR3468469:0.00000100)0.3586:0.0000010
0,ERR4187618:0.00000100)0.3589:0.00000100,ERR3439311:0.00000100)0.3591:0.00000100,E
RR4187754:0.00000100)0.3594:0.00000100,ERR4187758:0.00000100)0.3597:0.00000100,ERR3
439589:0.00000100)0.3599:0.00000100,ERR3439459:0.00000100)0.3602:0.00000100,ERR4187
965:0.00000100)0.3605:0.00000100,ERR4188082:0.00000100)0.3607:0.00000100,ERR4187897
:0.00000100)0.3609:0.00000100,ERR4188107:0.00000100)0.3612:0.00000100,ERR4187737:0.0
0010800)0.3614:0.00000100,ERR4187692:0.00000100)0.3617:0.00000100,ERR4188225:0.0000
0100)0.3619:0.00000100,ERR3439551:0.00000100)0.3622:0.00000100,ERR4188263:0.0000010
0)0.3625:0.00000100,ERR4187867:0.00000100)0.3627:0.00000100,ERR4187701:0.00000100)0.
3630:0.00000100,ERR4188188:0.00000100)0.3633:0.00000100,ERR4188156:0.00000100)0.363
5:0.00000100,ERR3439411:0.00000100)0.3638:0.00000100,ERR3439246:0.00000100)0.3641:0.
00000100,ERR4187850:0.00000100)0.3644:0.00000100,ERR3468490:0.00000100)0.3646:0.000
00100,ERR4188252:0.00000100)0.3649:0.00000100,ERR4187938:0.00000100)0.3652:0.000001
00,ERR4187978:0.00000100)0.3654:0.00000100,ERR3439306:0.00000100)0.3657:0.00000100,
ERR3439449:0.00000100)0.3660:0.00000100,ERR4188377:0.00000100)0.3663:0.00000100,ERR
4187767:0.00000100)0.3666:0.00000100,ERR3468790:0.00000100)0.3668:0.00000100,ERR346
8513:0.00000100)0.3671:0.00000100,ERR4188431:0.00000100)0.3674:0.00000100,ERR418789
0:0.00000100)0.3676:0.00000100,ERR5162132:0.00000100)0.3679:0.00000100,ERR4188096:0.
00000100)0.3682:0.00000100,ERR4188275:0.00000100)0.3685:0.00000100,ERR4187622:0.000
00100)0.3688:0.00000100,ERR3439255:0.00000100)0.3691:0.00000100,ERR3468443:0.000108

00)0.3694:0.00000100,ERR5162102:0.00000100)0.3697:0.00000100,ERR3468504:0.00000100)0.3699:0.00000100,ERR3439395:0.00000100)0.3702:0.00000100,ERR4187893:0.00000100)0.3705:0.00000100,ERR4187826:0.00000100)0.3708:0.00000100,ERR4187807:0.00000100)0.3711:0.00000100,ERR4899138:0.00000100)0.3714:0.00000100,ERR5162105:0.00000100)0.3717:0.00000100,ERR3468498:0.00000100)0.3720:0.00000100,ERR4188388:0.00000100)0.3723:0.00000100,ERR4187946:0.00000100)0.3725:0.00000100,ERR4187639:0.00000100)0.3728:0.00000100,ERR3468608:0.00000100)0.3731:0.00000100,ERR5162109:0.00000100)0.3734:0.00000100,ERR4187669:0.00000100)0.3737:0.00000100,ERR4188403:0.00000100)0.3740:0.00000100,ERR3439598:0.00000100)0.3743:0.00000100,ERR046989:0.00000100)0.3745:0.00000100,ERR3468556:0.00000100)0.3748:0.00000100,ERR4188043:0.00000100)0.3751:0.00000100,ERR3439256:0.00000100)0.3754:0.00000100,ERR4188071:0.00000100)0.3757:0.00000100,ERR3468521:0.00000100)0.3760:0.00000100,ERR4188365:0.00000100)0.3763:0.00000100,ERR3439347:0.00000100)0.3766:0.00000100,ERR3439533:0.00000100)0.3768:0.00000100,ERR3468555:0.00000100)0.3771:0.00000100,ERR3468637:0.00000100)0.3774:0.00000100,ERR3468420:0.00000100)0.3777:0.00000100,ERR3468795:0.00000100)0.3781:0.00000100,ERR3439259:0.00000100)0.3784:0.00000100,ERR4188331:0.00000100)0.3787:0.00000100,ERR4188321:0.00000100)0.3789:0.00000100,ERR3468446:0.00000100)0.3792:0.00000100,ERR3439578:0.00000100)0.3795:0.00000100,ERR3439508:0.00000100)0.3799:0.00000100,ERR3439597:0.00000100)0.3802:0.00000100,ERR4188209:0.00000100)0.3805:0.00000100,ERR4187643:0.00000100)0.3808:0.00000100,ERR4188243:0.00000100)0.3811:0.00000100,ERR3439266:0.00010800)0.3814:0.00000100,ERR3468699:0.00000100)0.3816:0.00000100,ERR4187802:0.00000100)0.3819:0.00000100,ERR4188299:0.00000100)0.3822:0.00000100,ERR4188387:0.00000100)0.3825:0.00000100,ERR5162074:0.00000100)0.3828:0.00000100,ERR3439427:0.00000100)0.3832:0.00000100,ERR4188324:0.00000100)0.3835:0.00000100,ERR3468508:0.00010800)0.3838:0.00000100,ERR4187674:0.00000100)0.3841:0.00000100,ERR3468600:0.00000100)0.3844:0.00000100,ERR4187936:0.00000100)0.3847:0.00000100)0.5353:0.00075600)0.5353:0.00000100)0.5354:0.00000100)0.5355:0.00000100)0.5355:0.00000100)0.5356:0.00000100)0.5357:0.00000100)0.5357:0.00000100)0.5358:0.00000100,((SRR16278249:0.00000100,SRR16278224:0.00000100)0.1200:0.00000100,SRR16278252:0.00010800)0.1633:0.00000100,SRR16278292:0.00000100)0.2889:0.00000100,SRR16278266:0.00000100)0.4375:0.00000100,SRR16278282:0.00000100)0.5567:0.00118900,((((((((((((((((SRR16278220:0.00000100,(((((((((((((((((SRR16278261:0.00000100,SRR16278273:0.00000100)0.0167:0.00000100,SRR16278274:0.00000100)0.0283:0.00000100,SRR16278229:0.00000100)0.0311:0.00000100,SRR16278248:0.00000100)0.0383:0.00000100,SRR16278272:0.00000100)0.0440:0.00000100,SRR16278240:0.00000100)0.0550:0.00000100,SRR16278278:0.00000100)0.0667:0.00000100,SRR16278247:0.00000100)0.0792:0.00000100,SRR16278225:0.00000100)0.0904:0.00000100,SRR16278226:0.00000100)0.1020:0.00000100,SRR16278236:0.00000100)0.1106:0.00000100,SRR16278268:0.00000100)0.1175:0.00000100,SRR16278281:0.00000100)0.1228:0.00000100,SRR16278267:0.00000100)0.1295:0.00000100,SRR16278263:0.00000100)0.1391:0.00000100,SRR16278227:0.00000100)0.1477:0.00000100,SRR16278231:0.00000100)0.1592:0.00000100)0.1693:0.00000100,SRR16278221:0.00000100)0.1826:0.00000100,SRR16278257:0.00000100)0.2002:0.00000100,SRR16278245:0.00000100)0.2189:0.00000100,((((SRR16278277:0.00000100,SRR16278242:0.00000100)0.1000:0.00000100,SRR16278256:0.00000100)0.1833:0.00000100,SRR16278284:0.00000100)0.2800:0.00000100,SRR16278259:0.00000100)0.3658:0.00010800)0

.2795:0.00000100,((((((((SRR16278271:0.00000100,SRR16278289:0.00000100)0.0600:0.000001
00,SRR16278276:0.00000100)0.0700:0.00000100,SRR16278223:0.00000100)0.1100:0.0000010
0,SRR16278291:0.00000100)0.1658:0.00000100,SRR16278237:0.00000100)0.2127:0.00000100,
SRR16278235:0.00000100)0.2700:0.00000100,SRR16278232:0.00000100)0.3295:0.00000100,S
RR16278287:0.00000100)0.3738:0.00010800)0.4408:0.00000100,SRR16278233:0.00000100)0.
4552:0.00000100,SRR16278241:0.00000100)0.4721:0.00000100,SRR16278285:0.00000100)0.4
865:0.00000100,SRR16278239:0.00000100)0.5030:0.00000100,SRR16278228:0.00000100)0.51
88:0.00000100,SRR16278244:0.00000100)0.5294:0.00000100,SRR16278258:0.00000100)0.543
2:0.00000100,SRR16278260:0.00000100)0.5547:0.00064800,SRR16278230:0.00054000)0.5457:
0.00000100,(((((SRR16278253:0.00000100,SRR16278243:0.00000100)0.1000:0.00000100,SRR1
6278269:0.00000100)0.2167:0.00000100,SRR16278254:0.00000100)0.3222:0.00000100,SRR16
278234:0.00000100)0.4633:0.00000100,SRR16278264:0.00000100)0.5447:0.00064800)0.5523:
0.00032400)0.5538:0.00075700,((ERR6142329:0.00000100,(ERR6140775:0.00000100,(ERR6149
413:0.00000100,(ERR6149470:0.00000100,((((SRR8063651:0.00021600,SRR8063660:0.0004320
0)0.4867:0.00043200,((((((((SRR8065078:0.00021600,SRR8065082:0.00032400)0.3133:0.00010
800,(SRR8065085:0.00000100,SRR8065086:0.00000100)0.5033:0.00032400)0.3189:0.0000010
0,SRR8065070:0.00064800)0.3142:0.00000100,(SRR8065079:0.00000100,SRR8065071:0.00000
100)0.3767:0.00010800)0.4239:0.00000100,SRR8065069:0.00010800)0.5014:0.00000100,SRR8
065087:0.00032400)0.5521:0.00021600,SRR8065080:0.00043200)0.5511:0.00021600,(SRR806
5073:0.00064800,(SRR8065081:0.00000100,SRR8065074:0.00000100)0.4400:0.00097300)0.51
67:0.00032400)0.5583:0.00086500)0.4971:0.00000100,((((SRR8063663:0.00000100,((((((SRR80
63666:0.00000100,SRR8063654:0.00000100)0.1033:0.00000100,SRR8063653:0.00000100)0.16
33:0.00000100,SRR8063659:0.00000100)0.2189:0.00000100,SRR8063664:0.00000100)0.2867:0
.00000100,SRR8063665:0.00000100)0.3840:0.00000100,SRR8063661:0.00000100)0.4761:0.000
00100)0.5395:0.00010800,SRR8063656:0.00010800)0.5563:0.00054000,((SRR8063655:0.00000
100,SRR8063662:0.00000100)0.3833:0.00021600,SRR8063652:0.00075600)0.4417:0.00010800
)0.5542:0.00043200,(SRR1791710:0.00000100,SRR1791712:0.00000100)0.4167:0.00151300)0.
5115:0.00000100)0.5552:0.00043200,(SRR16278251:0.00021600,SRR16278280:0.00000100)0.
4367:0.00129700)0.5198:0.00000100)0.5030:0.00000100)0.4873:0.00000100)0.4725:0.000001
00)0.4586:0.00000100,((((((((((((((SRR1792479:0.00000100,SRR1792481:0.00000100)0.3200:0.
00010800,(SRR1792474:0.00000100,SRR1792488:0.00000100)0.1833:0.00000100)0.3578:0.00
000100,(SRR1792482:0.00000100,SRR1792161:0.00000100)0.3100:0.00010800)0.4793:0.0000
0100,SRR1792493:0.00010800)0.5450:0.00151400,((
((ERR6337402:0.00000100,ERR6338275:0.00000100)0.0067:0.0
0000100,ERR6337348:0.00000100)0.0133:0.00000100,ERR6337409:0.00000100)0.0144:0.0000
0100,ERR6337360:0.00000100)0.0167:0.00000100,(((((((ERR6336658:0.00000100,ERR6337375:
0.00000100)0.0700:0.00000100,ERR6336662:0.00000100)0.0950:0.00000100,ERR6337383:0.0
0000100)0.1389:0.00000100,ERR6336657:0.00000100)0.1992:0.00000100,ERR6337205:0.0000
0100)0.2567:0.00000100,ERR6337203:0.00000100)0.3139:0.00000100,ERR6336684:0.0000010
0)0.3552:0.00010800)0.2158:0.00000100,(((((((((((ERR6338360:0.00000100,ERR6338274:0.000
00100)0.0367:0.00000100,ERR6338300:0.00000100)0.0650:0.00000100,ERR6338279:0.000001
00)0.0833:0.00000100,ERR6338364:0.00000100)0.1050:0.00000100,ERR6338278:0.00000100)
0.1353:0.00000100,ERR6338305:0.00000100)0.1583:0.00000100,ERR6337390:0.00000100)0.1
910:0.00000100,ERR6338293:0.00000100)0.2267:0.00000100,ERR6338301:0.00000100)0.2711

:0.00000100,ERR6337388:0.00000100)0.3060:0.00000100,ERR6338359:0.00000100)0.3445:0.0
0000100,ERR6338297:0.00000100)0.3742:0.00010800)0.2223:0.00000100,((((((((((((((((ERR633
6683:0.00000100,ERR6338363:0.00000100)0.0400:0.00000100,ERR6336827:0.00000100)0.041
7:0.00000100,ERR6337204:0.00000100)0.0622:0.00000100,ERR6337364:0.00000100)0.0717:0.
00000100,ERR6336810:0.00000100)0.0887:0.00000100,ERR6337400:0.00000100)0.1033:0.000
00100,ERR6338282:0.00010800)0.1152:0.00000100,ERR6338369:0.00000100)0.1383:0.000001
00,ERR6337413:0.00000100)0.1526:0.00000100,ERR6336663:0.00000100)0.1787:0.00000100,
ERR6337339:0.00000100)0.2130:0.00000100,ERR6337386:0.00000100)0.2492:0.00000100,ERR
6337389:0.00000100)0.2833:0.00000100,ERR6336682:0.00000100)0.3160:0.00000100,ERR633
7392:0.00000100)0.3400:0.00000100,ERR6337069:0.00000100)0.3629:0.00000100,ERR633661
9:0.00000100)0.3867:0.00010800)0.2067:0.00000100,(ERR6338296:0.00000100,ERR6338307:0
.00000100)0.2600:0.00010800)0.2007:0.00000100,ERR6358027:0.00000100)0.1964:0.0000010
0,((ERR6337377:0.00000100,ERR6337396:0.00000100)0.1933:0.00000100,ERR6337355:0.0000
0100)0.3333:0.00010800)0.1893:0.00000100,ERR6338281:0.00000100)0.1858:0.00000100,ERR
6337414:0.00000100)0.1825:0.00000100,ERR6337068:0.00000100)0.1792:0.00000100,ERR633
7408:0.00000100)0.1763:0.00000100,ERR6336618:0.00000100)0.1735:0.00000100,ERR633740
3:0.00000100)0.1710:0.00000100,ERR6357083:0.00000100)0.1682:0.00000100,ERR6336660:0.
00000100)0.1656:0.00000100,ERR6338367:0.00000100)0.1631:0.00000100,ERR6338299:0.000
00100)0.1611:0.00000100,ERR6338361:0.00000100)0.1592:0.00000100,ERR6337353:0.000001
00)0.1570:0.00000100,ERR6336828:0.00000100)0.1547:0.00000100,ERR6337342:0.00000100)
0.1531:0.00000100,ERR6338368:0.00000100)0.1511:0.00000100,ERR6336659:0.00000100)0.1
495:0.00000100,ERR6336661:0.00000100)0.1483:0.00000100,ERR6337404:0.00000100)0.1478
:0.00000100,ERR6338292:0.00000100)0.1468:0.00000100,ERR6337410:0.00000100)0.1463:0.0
0000100,((ERR6337395:0.00000100,ERR6338352:0.00000100)0.1900:0.00000100,ERR6337394:
0.00000100)0.3083:0.00010800)0.1481:0.00000100,ERR6337359:0.00000100)0.1494:0.000001
00,ERR6338287:0.00000100)0.1505:0.00000100,ERR6337349:0.00000100)0.1520:0.00000100,
ERR6338295:0.00000100)0.1539:0.00000100,ERR6336686:0.00000100)0.1563:0.00000100,ERR
6337206:0.00000100)0.1583:0.00000100,ERR6338354:0.00000100)0.1612:0.00000100,ERR633
7345:0.00000100)0.1652:0.00000100,ERR6338291:0.00000100)0.1706:0.00000100,ERR633735
6:0.00000100)0.1770:0.00000100,ERR6338273:0.00000100)0.1831:0.00000100,ERR6337350:0.
00000100)0.1904:0.00000100,ERR6338270:0.00000100)0.1982:0.00000100,ERR6337381:0.000
00100)0.2055:0.00000100,ERR6337366:0.00000100)0.2134:0.00000100,ERR6338271:0.000001
00)0.2218:0.00000100,ERR6338351:0.00000100)0.2290:0.00000100,ERR6338298:0.00000100)
0.2378:0.00000100,ERR6336617:0.00000100)0.2459:0.00000100,ERR6338290:0.00000100)0.2
537:0.00000100,ERR6338280:0.00000100)0.2615:0.00000100,ERR6338277:0.00000100)0.2692
:0.00000100,ERR6337341:0.00000100)0.2773:0.00000100,ERR6338284:0.00000100)0.2851:0.0
0000100,ERR6337344:0.00000100)0.2930:0.00000100,ERR6337379:0.00000100)0.2999:0.0000
0100,ERR6337397:0.00000100)0.3073:0.00000100,ERR6338294:0.00000100)0.3155:0.0000010
0,ERR6337361:0.00000100)0.3221:0.00000100,ERR6337412:0.00000100)0.3290:0.00000100,E
RR6337393:0.00000100)0.3349:0.00000100,ERR6337346:0.00000100)0.3423:0.00000100,ERR6
336809:0.00000100)0.3492:0.00000100,ERR6338269:0.00000100)0.3568:0.00000100,ERR6338
357:0.00000100)0.3629:0.00000100,ERR6338283:0.00000100)0.3688:0.00000100,ERR6338286
:0.00000100)0.3746:0.00000100,ERR6338276:0.00000100)0.3817:0.00000100,ERR6337401:0.0
0000100)0.3888:0.00000100,ERR6338272:0.00000100)0.3950:0.00000100,ERR6337398:0.0000

0100)0.4013:0.00000100,ERR6337352:0.00000100)0.4073:0.00000100,ERR6338362:0.0000010
0)0.4133:0.00000100,ERR6338304:0.00000100)0.4192:0.00000100,ERR6338289:0.00000100)0.
4244:0.00000100,ERR6337385:0.00010800)0.4296:0.00000100,ERR6338306:0.00000100)0.434
6:0.00000100,ERR6336616:0.00000100)0.4398:0.00000100,ERR6337343:0.00000100)0.4452:0.
00000100,ERR6337357:0.00000100)0.4502:0.00000100,ERR6337347:0.00000100)0.4554:0.000
00100,ERR6337340:0.00000100)0.4607:0.00000100,ERR6337411:0.00000100)0.4662:0.000001
00,ERR6338366:0.00000100)0.4708:0.00000100,ERR6338285:0.00000100)0.4760:0.00000100,
ERR6337384:0.00000100)0.4807:0.00000100,ERR6338355:0.00000100)0.4853:0.00000100,ERR
6337399:0.00000100)0.4908:0.00000100,ERR6338302:0.00010800)0.4951:0.00000100,ERR633
7351:0.00000100)0.4996:0.00000100,ERR6338353:0.00000100)0.5046:0.00000100,ERR633740
5:0.00010800)0.5086:0.00000100,ERR6337406:0.00000100)0.5134:0.00000100,ERR6338356:0.
00000100)0.5184:0.00000100,ERR6337391:0.00000100)0.5219:0.00000100,ERR6338288:0.000
00100)0.5260:0.00000100,ERR6338358:0.00000100)0.5301:0.00000100,ERR6338303:0.000001
00)0.5344:0.00000100,ERR6337202:0.00000100)0.5383:0.00000100,ERR6336685:0.00000100)
0.5420:0.00000100,ERR6337067:0.00000100)0.5457:0.00000100,ERR6337378:0.00000100)0.5
493:0.00000100,ERR6338365:0.00000100)0.5537:0.00064800)0.5293:0.00000100,(((((((((((((((((
(((ERR6201807:0.0000
0100,((((((((((((((((((((ERR6201842:0.00000100,ERR6201896:0.00000100)0.0200:0.00000100,ERR
6210148:0.00000100)0.0350:0.00000100,ERR6210150:0.00000100)0.0400:0.00000100,ERR619
9027:0.00000100)0.0550:0.00000100,ERR6201838:0.00000100)0.0667:0.00000100,ERR620185
2:0.00000100)0.0806:0.00000100,ERR6209542:0.00010800)0.0886:0.00000100,ERR6201828:0.
00000100)0.0967:0.00000100,((ERR6198390:0.00000100,ERR6201872:0.00000100)0.1933:0.00
000100,ERR6201801:0.00000100)0.3467:0.00010800)0.1488:0.00000100,ERR6201865:0.00000
100)0.1553:0.00000100,ERR6201885:0.00000100)0.1769:0.00000100,ERR6201814:0.00000100
)0.2007:0.00000100,ERR6210236:0.00000100)0.2251:0.00000100,ERR6201830:0.00000100)0.2
446:0.00000100,ERR6201832:0.00000100)0.2659:0.00000100,ERR6210161:0.00000100)0.2852
:0.00000100,ERR6201841:0.00000100)0.3002:0.00000100,ERR6201868:0.00000100)0.3158:0.0
0000100,ERR6201845:0.00000100)0.3278:0.00000100,ERR6201861:0.00000100)0.3414:0.0001
0800)0.3268:0.00000100,(((((((((((((((((((((ERR6210188:0.00000100,ERR6209352:0.00000100)0.
0267:0.00000100,ERR6210130:0.00000100)0.0333:0.00000100,ERR6210230:0.00000100)0.055
6:0.00000100,ERR6209353:0.00000100)0.0742:0.00000100,ERR6210201:0.00000100)0.0827:0.
00000100,ERR6209345:0.00000100)0.1006:0.00000100,ERR6209492:0.00000100)0.1114:0.000
00100,ERR6209343:0.00000100)0.1271:0.00000100,ERR6209423:0.00000100)0.1511:0.000001
00,ERR6209576:0.00000100)0.1687:0.00000100,ERR6209346:0.00000100)0.1936:0.00000100,
ERR6209527:0.00000100)0.2228:0.00000100,ERR6209573:0.00000100)0.2595:0.00000100,ERR
6201875:0.00000100)0.2960:0.00000100,ERR6209524:0.00000100)0.3260:0.00000100,ERR621
0127:0.00000100)0.3592:0.00000100,ERR6210128:0.00000100)0.3935:0.00000100,ERR620191
5:0.00000100)0.4220:0.00000100,ERR6210158:0.00000100)0.4484:0.00000100,ERR6209444:0.
00000100)0.4722:0.00000100,ERR6210195:0.00000100)0.4922:0.00021600)0.2727:0.0000010
0,ERR6209323:0.00000100)0.2668:0.00000100,ERR6198401:0.00000100)0.2613:0.00000100,(((
((((ERR6209445:0.00000100,ERR6210267:0.00010800)0.3067:0.00010800,ERR6210272:0.00000
100)0.1950:0.00000100,ERR6210216:0.00000100)0.2000:0.00000100,ERR6210258:0.00000100
)0.2542:0.00000100,ERR6210149:0.00000100)0.2973:0.00000100,ERR6210252:0.00000100)0.3
478:0.00000100,ERR6210169:0.00010800)0.3781:0.00010800)0.2341:0.00000100,ERR6201897

:0.00000100)0.2299:0.00000100,ERR6201858:0.00000100)0.2260:0.00000100,((((ERR6209044:
0.00000100,ERR6209491:0.00000100)0.1133:0.00000100,ERR6201891:0.00000100)0.1817:0.0
0000100,ERR6208976:0.00000100)0.2856:0.00000100,ERR6209424:0.00000100)0.3550:0.0001
0800)0.2126:0.00000100,ERR6201909:0.00000100)0.2096:0.00000100,ERR6198291:0.0000010
0)0.2067:0.00000100,ERR6201836:0.00000100)0.2037:0.00000100,ERR6210207:0.00000100)0.
2008:0.00000100,ERR6201820:0.00000100)0.1980:0.00000100,(((((((((((ERR6201819:0.000001
00,ERR6209278:0.00000100)0.0300:0.00000100,ERR6201879:0.00000100)0.0550:0.00000100,
ERR6198449:0.00000100)0.0800:0.00000100,ERR6210212:0.00000100)0.1000:0.00000100,ERR
6201818:0.00000100)0.1327:0.00000100,ERR6209309:0.00000100)0.1583:0.00000100,ERR620
1910:0.00000100)0.1957:0.00000100,ERR6201809:0.00000100)0.2354:0.00000100,ERR619839
8:0.00000100)0.2711:0.00000100,ERR6201833:0.00000100)0.3027:0.00000100,ERR6209301:0.
00000100)0.3388:0.00000100,ERR6198395:0.00000100)0.3731:0.00010800)0.1870:0.0000010
0,ERR6201870:0.00000100)0.1851:0.00000100,(((((((((ERR6201854:0.00000100,ERR6201853:0
.00000100)0.0267:0.00000100,ERR6201960:0.00000100)0.0633:0.00000100,ERR6201978:0.00
000100)0.0867:0.00000100,ERR6209443:0.00000100)0.1358:0.00000100,ERR6201821:0.00000
100)0.1647:0.00000100,ERR6209349:0.00000100)0.2133:0.00000100,ERR6210268:0.00000100
)0.2381:0.00000100,ERR6210175:0.00000100)0.2908:0.00000100,ERR6201829:0.00000100)0.3
226:0.00000100,ERR6209422:0.00000100)0.3493:0.00010800)0.1774:0.00000100,ERR6209344
:0.00000100)0.1763:0.00000100,ERR6210223:0.00000100)0.1751:0.00000100,ERR6201882:0.0
0000100)0.1740:0.00000100,ERR6201863:0.00000100)0.1734:0.00000100,ERR6210259:0.0000
0100)0.1725:0.00000100,ERR6209350:0.00000100)0.1715:0.00000100,ERR6201812:0.0000010
0)0.1709:0.00000100,ERR6198389:0.00000100)0.1706:0.00000100,ERR6202401:0.00000100)0.
1699:0.00000100,ERR6201794:0.00000100)0.1701:0.00000100,ERR6201877:0.00000100)0.170
1:0.00000100,ERR6198210:0.00000100)0.1710:0.00000100,ERR6201855:0.00000100)0.1715:0.
00000100,ERR6201866:0.00000100)0.1719:0.00000100,ERR6209348:0.00010800)0.1732:0.000
00100,ERR6201894:0.00000100)0.1739:0.00000100,ERR6201857:0.00000100)0.1754:0.000001
00,ERR6201907:0.00000100)0.1774:0.00000100,ERR6201886:0.00000100)0.1795:0.00000100,(
ERR6202040:0.00000100,ERR6201906:0.00000100)0.2800:0.00010800)0.1848:0.00000100,ERR
6201899:0.00010800)0.1872:0.00000100,ERR6201871:0.00000100)0.1893:0.00000100,ERR620
1945:0.00000100)0.1922:0.00000100,ERR6201937:0.00000100)0.1951:0.00000100,ERR620187
4:0.00000100)0.1983:0.00000100,ERR6198394:0.00000100)0.2016:0.00000100,ERR6198379:0.
00000100)0.2052:0.00000100,ERR6201908:0.00000100)0.2092:0.00000100,ERR6201798:0.000
00100)0.2125:0.00000100,ERR6201849:0.00000100)0.2169:0.00000100,ERR6201847:0.000001
00)0.2213:0.00000100,((ERR6201898:0.00000100,ERR6201895:0.00000100)0.1533:0.00000100
,ERR6201893:0.00000100)0.3250:0.00010800)0.2325:0.00000100,ERR6198376:0.00010800)0.2
363:0.00000100,ERR6201823:0.00000100)0.2400:0.00000100,ERR6201901:0.00000100)0.2434
:0.00000100,ERR6210270:0.00000100)0.2478:0.00000100,ERR6201862:0.00000100)0.2510:0.0
0000100,ERR6201878:0.00000100)0.2549:0.00000100,ERR6198204:0.00000100)0.2590:0.0000
0100,ERR6201902:0.00010800)0.2633:0.00000100,ERR6201883:0.00000100)0.2673:0.0000010
0,ERR6206178:0.00000100)0.2714:0.00000100,ERR6201811:0.00000100)0.2753:0.00000100,E
RR6201800:0.00000100)0.2792:0.00000100,ERR6201888:0.00000100)0.2831:0.00000100,ERR6
201839:0.00000100)0.2865:0.00000100,ERR6201831:0.00000100)0.2905:0.00000100,ERR6209
335:0.00000100)0.2939:0.00000100,ERR6210129:0.00000100)0.2979:0.00000100,ERR6210147
:0.00000100)0.3019:0.00000100,ERR6201972:0.00000100)0.3053:0.00000100,ERR6209526:0.0

0000100)0.3094:0.00000100,ERR6203392:0.00000100)0.3133:0.00000100,ERR6198207:0.0000
0100)0.3173:0.00000100,ERR6208726:0.00000100)0.3212:0.00000100,ERR6201804:0.0000010
0)0.3249:0.00000100,ERR6210269:0.00000100)0.3286:0.00000100,ERR6201826:0.00000100)0.
3327:0.00000100,ERR6201889:0.00000100)0.3360:0.00000100,ERR6198443:0.00000100)0.339
7:0.00000100,ERR6201860:0.00010800)0.3434:0.00000100,ERR6201986:0.00000100)0.3470:0.
00000100,ERR6202004:0.00000100)0.3501:0.00000100,ERR6201837:0.00000100)0.3534:0.000
00100,ERR6198445:0.00000100)0.3571:0.00000100,ERR6201998:0.00000100)0.3604:0.000001
00,ERR6201869:0.00000100)0.3638:0.00000100,ERR6201815:0.00000100)0.3674:0.00000100,
ERR6201835:0.00000100)0.3707:0.00000100,ERR6198448:0.00000100)0.3738:0.00000100,ERR
6198216:0.00000100)0.3770:0.00000100,ERR6201810:0.00000100)0.3798:0.00000100,ERR619
8377:0.00000100)0.3837:0.00000100,ERR6201903:0.00000100)0.3867:0.00000100,ERR621027
1:0.00000100)0.3895:0.00000100,ERR6198400:0.00000100)0.3933:0.00000100,ERR6198391:0.
00000100)0.3965:0.00000100,ERR6209351:0.00000100)0.3997:0.00000100,ERR6209493:0.000
00100)0.4024:0.00000100,ERR6198442:0.00000100)0.4053:0.00000100,ERR6201844:0.000001
00)0.4079:0.00000100,ERR6204677:0.00010800)0.4108:0.00000100,ERR6201890:0.00000100)
0.4138:0.00000100,ERR6198378:0.00000100)0.4165:0.00000100,ERR6201797:0.00000100)0.4
196:0.00000100,ERR6201848:0.00000100)0.4222:0.00000100,ERR6201817:0.00000100)0.4249
:0.00000100,ERR6201825:0.00000100)0.4274:0.00000100,ERR6201808:0.00000100)0.4299:0.0
0000100,ERR6201813:0.00010800)0.4320:0.00000100,ERR6201806:0.00000100)0.4347:0.0000
0100,ERR6210266:0.00000100)0.4374:0.00000100,ERR6198399:0.00000100)0.4399:0.0000010
0,ERR6198117:0.00000100)0.4422:0.00000100,ERR6201822:0.00000100)0.4446:0.00000100,E
RR6201884:0.00000100)0.4468:0.00000100,ERR6198349:0.00000100)0.4489:0.00000100,SRR1
5296934:0.00010800)0.4507:0.00000100,ERR6201851:0.00000100)0.4531:0.00000100,ERR619
8447:0.00000100)0.4562:0.00021600,(((SRR5486086:0.00000100,(SRR5486084:0.00000100,((S
RR5486079:0.00010800,SRR5486082:0.00000100)0.1433:0.00000100,(SRR5486077:0.0000010
0,SRR5486078:0.00010800)0.3233:0.00010800)0.4344:0.00010800)0.3950:0.00000100)0.4260:
0.00000100,SRR5486080:0.00010800)0.4889:0.00000100,SRR5486074:0.00010800)0.5514:0.00
162100)0.4392:0.00000100)0.3048:0.00000100,(((((((((((((((((((ERR027454:0.00000100,((((((((((
((ERR229973:0.00086600,ERR133
980:0.00097500)0.0000:0.00000100,(((ERR386905:0.0014
0900,ERR067679:0.00010800)0.0000:0.00000100,((
((((((((((ERR046780:0.00000100,ERR046779:0.00000100)0.2500:0.00075800,ERR229971:0.0002
1600)0.1283:0.00000100,(((((((ERR386850:0.00000100,ERR351918:0.00000100)0.3033:0.00097
400,ERR067584:0.00021600)0.1517:0.00000100,((((((((((((((((((((ERR403312:0.00140800,ERR133
981:0.00173300)0.0067:0.00000100,((ERR067725:0.00054100,ERR4
03402:0.00119100)0.0033:0.00000100,(((ERR386991:0.
00216700,ERR040122:0.00205800)0.0000:0.00000100,((
(((((((((((((((ERR158599:0.00097500,ERR133964:0.00064900)0.0000:0.00000100,(((((((((((((((((((((((((((((
(((((((ERR403355:0.00162500,(ERR067655:0.00075800,ERR229945:0.00065000)0.2667:0.00021
600)0.1333:0.00000100,(((ERR039333:0.00000100,E
RR039334:0.00000100)0.2833:0.00097400,((ERR133899:0.00000100,ERR234650:0.00010800)0.
1167:0.00000100,ERR229995:0.00010800)0.2083:0.00010800)0.1483:0.00000100,(((((((((((((((((((
((((((((((((((ERR403340:0.00054100,ERR234580:0.00108300)0.0000:0.00000100,(((((((ERR133906
:0.00010800,Reference:0.00390800)0.0067:0.00000100,((((((((((((((((((((((ERR403334:0.00000100,

ERR351937:0.00000100)0.2300:0.00000100,ERR386869:0.00000100)0.3733:0.00140800,(((ERR
386883:0.00043300,ERR017786:0.00000100)0.1400:0.00000100,ERR019860:0.00000100)0.303
3:0.00000100,ERR386982:0.00000100)0.4022:0.00043300)0.2261:0.00000100,((((((((((((((((((((
(((((((((((((((((((ERR387038:0.00510500,((ERR133817:0.00000100,ERR067614:0.00010800)0.12
33:0.00000100,ERR133813:0.00000100)0.2317:0.00010800)0.1544:0.00000100,((((((((((((((((((
(((((((((((((((((((ERR133971:0.00000100,ERR133965:0.00000100)0.2567:0.00086600,ERR40334
3:0.00075800)0.1283:0.00000100,ERR133922:0.00000100)0.0856:0.00000100,((((((((ERR40335
6:0.00043300,((ERR386859:0.00000100,ERR351927:0.00000100)0.3100:0.00054100,ERR38702
5:0.00129900)0.3867:0.00292900)0.2589:0.00000100,(((((((((((((((((((((((ERR386876:0.000974
00,(ERR027447:0.00010800,ERR234668:0.00010800)0.1333:0.00010800)0.0683:0.00000100,(((
(((((((((((((((((((ERR351846:0.00000100,ERR386778:0.00000100)0.2567:0.00086600,(ERR38678
3:0.00000100,ERR351851:0.00000100)0.2633:0.00108300)0.1500:0.00000100,(((((((((ERR3869
60:0.00000100,ERR387019:0.00000100)0.2067:0.00000100,ERR403341:0.00000100)0.3583:0.0
0043300,ERR403405:0.00086600)0.3500:0.00000100,ERR386903:0.00108200)0.3950:0.001733
00,ERR386909:0.00054100)0.3160:0.00000100,((((((((((((((((((((ERR067726:0.00162500,(ERR13
7208:0.00000100,ERR234661:0.00010800)0.2067:0.00010800)0.1033:0.00000100,(((((((((((((((
(((ERR403246:0.00260100,(ERR067690:0.00010800,ERR067591:0.00043300)0.1767:0.00010800
)0.0900:0.00000100,(((((((((((((((((((((((((((((ERR046959:0.00000100,ERR046972:0.00000100)0.
0633:0.00000100,ERR046958:0.00000100)0.1567:0.00000100,ERR046994:0.00000100)0.2133:
0.00000100,ERR046974:0.00000100)0.2667:0.00000100,ERR046992:0.00000100)0.3027:0.000
00100,ERR046957:0.00010800)0.3367:0.00000100,ERR046993:0.00000100)0.3729:0.00000100
,ERR046990:0.00000100)0.4108:0.00086600,(((((ERR046947:0.00000100,ERR046966:0.000108
00)0.0800:0.00000100,ERR046949:0.00000100)0.1733:0.00000100,ERR046963:0.00000100)0.2
533:0.00000100,ERR046938:0.00000100)0.3467:0.00000100,ERR046967:0.00000100)0.3980:0.
00097400)0.2440:0.00000100,((((((((((((((((((((((((((ERR386980:0.00205900,(ERR067589:0.000
00100,ERR403412:0.00032500)0.1500:0.00010800)0.0750:0.00000100,((((((((ERR386954:0.000
86600,ERR158606:0.00010800)0.0000:0.00000100,((((ERR386912:0.00086600,(((ERR386987:0.
00086600,((((((ERR386889:0.00010800,((ERR386893:0.00010800,(ERR386782:0.00000100,ERR3
51850:0.00000100)0.2600:0.00010800)0.2533:0.00000100,(ERR386823:0.00000100,ERR35189
1:0.00000100)0.2500:0.00010800)0.3800:0.00010800)0.3887:0.00010800,(ERR158610:0.00021
600,ERR387020:0.00010800)0.2933:0.00021600)0.4043:0.00010800,ERR387012:0.00032500)0.
4046:0.00010800,(ERR387026:0.00000100,ERR386938:0.00000100)0.2500:0.00010800)0.3820:
0.00000100,ERR403374:0.00043300)0.3970:0.00000100,ERR386900:0.00010800)0.4147:0.000
21600)0.4049:0.00000100,ERR387055:0.00097400)0.4164:0.00043300,ERR403257:0.00119200
)0.4062:0.00010800)0.3808:0.00000100,ERR108489:0.00010800)0.3584:0.00000100,(((((((((((((
(ERR072021:0.00000100,ERR072022:0.00000100)0.2867:0.00108300,(((ERR067724:0.00000100
,ERR229965:0.00000100)0.1433:0.00000100,ERR108511:0.00000100)0.2650:0.00000100,ERR2
27983:0.00000100)0.3233:0.00021600)0.2120:0.00000100,((((((((((ERR403384:0.00108300,((E
RR046865:0.00000100,ERR046864:0.00000100)0.2600:0.00054100,(((ERR351904:0.00000100,E
RR386832:0.00000100)0.2133:0.00000100,ERR386836:0.00000100)0.3033:0.00000100,ERR351
900:0.00000100)0.3867:0.00086600)0.3960:0.00141000)0.3306:0.00000100,((((((((((((((((((((((((
((ERR386935:0.00151500,((ERR484744:0.00032400,ERR387005:0.00021600)0.2533:0.00021600
,ERR386888:0.00097400)0.3033:0.00000100)0.3744:0.00021400,(ERR351922:0.00000100,ERR3
86854:0.00000100)0.3367:0.00097600)0.4067:0.00260500,((((((ERR019871:0.00000100,ERR01

7797:0.00000100)0.2000:0.00010800,((ERR386950:0.00021600,ERR403395:0.00021600)0.0767
:0.00000100,ERR387041:0.00021600)0.1117:0.00000100)0.2567:0.00000100,ERR386930:0.000
10800)0.3053:0.00000100,(ERR386897:0.00021600,ERR403414:0.00000100)0.2200:0.0001080
0)0.3990:0.00010800,ERR403337:0.00086600)0.3988:0.00000100,ERR038741:0.00108200)0.41
04:0.00043300)0.2533:0.00000100,(ERR403387:0.00162500,ERR386924:0.00151600)0.1600:0.
00010800)0.2239:0.00000100,(((ERR403265:0.00108300,(ERR144579:0.00043300,(((ERR06774
0:0.00000100,(ERR108440:0.00000100,ERR067648:0.00000100)0.2600:0.00010800)0.2767:0.0
0000100,ERR144626:0.00021600)0.3400:0.00000100,ERR133929:0.00010800)0.3967:0.000325
00)0.3667:0.00010800)0.3056:0.00000100,((((((((((((((((((ERR046777:0.00000100,ERR047016:0.
00000100)0.0833:0.00000100,ERR386886:0.00000100)0.1617:0.00000100,ERR046778:0.00000
100)0.1978:0.00000100,ERR047005:0.00000100)0.2667:0.00000100,ERR046776:0.00000100)0.
3213:0.00000100,ERR047008:0.00000100)0.3839:0.00000100,ERR047006:0.00000100)0.4162:
0.00097400,ERR387013:0.00075800)0.3646:0.00000100,((((((((ERR040129:0.00000100,ERR040
118:0.00000100)0.1933:0.00000100,ERR040136:0.00000100)0.3467:0.00010800,ERR040140:0.
00010800)0.4033:0.00086600,ERR040141:0.00043300)0.3833:0.00010800,ERR038743:0.00054
100)0.3707:0.00000100,ERR040139:0.00064900)0.4056:0.00195100,(((((((((ERR040112:0.00000
100,ERR040113:0.00000100)0.1400:0.00000100,ERR040116:0.00000100)0.2783:0.00000100,E
RR040117:0.00000100)0.3956:0.00043300,((ERR038744:0.00010800,((ERR038745:0.00000100,
ERR038737:0.00000100)0.1767:0.00000100,ERR040134:0.00000100)0.2917:0.00000100)0.350
0:0.00021600,(ERR046871:0.00000100,ERR046870:0.00000100)0.2600:0.00043300)0.3400:0.0
0010800)0.3141:0.00000100,(ERR040120:0.00000100,ERR040142:0.00021600)0.2300:0.00021
600)0.3373:0.00010800,(((ERR046836:0.00000100,ERR046837:0.00000100)0.2700:0.00140800,
ERR403332:0.00054100)0.1583:0.00000100,(((ERR046884:0.00000100,ERR046885:0.00000100)
0.3033:0.00054100,(ERR386867:0.00000100,ERR351935:0.00000100)0.2900:0.00054100)0.206
7:0.00000100,ERR387033:0.00032500)0.1792:0.00000100)0.1790:0.00000100)0.3379:0.00000
100,(ERR387016:0.00043300,ERR038736:0.00086600)0.0500:0.00000100)0.3687:0.00000100,(
ERR039330:0.00000100,ERR046732:0.00000100)0.2700:0.00054100)0.3899:0.00032500,((((ER
R228033:0.00000100,ERR234685:0.00000100)0.1767:0.00000100,ERR228034:0.00000100)0.37
00:0.00032500,ERR133872:0.00021600)0.3967:0.00054200,ERR403382:0.00065000)0.3717:0.0
0010800)0.3830:0.00021600)0.3069:0.00000100,ERR133875:0.00054100)0.2983:0.00000100)0
.2390:0.00000100,ERR484747:0.00000100)0.2338:0.00000100,ERR137266:0.00021600)0.2288:
0.00000100,SRR16278270:0.00043300)0.2240:0.00000100,(((((((((((((((((((ERR351923:0.000001
00,ERR403369:0.00000100)0.1833:0.00000100,ERR403408:0.00000100)0.3133:0.00000100,ER
R386855:0.00000100)0.4167:0.00119200,(((((((((((((((((ERR038298:0.00000100,ERR046772:0.00
000100)0.0733:0.00000100,ERR046769:0.00000100)0.0850:0.00000100,ERR046940:0.0000010
0)0.1200:0.00000100,ERR046914:0.00000100)0.1417:0.00000100,ERR040088:0.00010800)0.15
07:0.00000100,ERR039323:0.00000100)0.1794:0.00000100,ERR046771:0.00000100)0.2086:0.0
0000100,ERR040108:0.00000100)0.2367:0.00000100,ERR040087:0.00000100)0.2622:0.000001
00,ERR046729:0.00000100)0.2990:0.00000100,ERR046770:0.00000100)0.3248:0.00000100,ER
R046942:0.00000100)0.3572:0.00000100,ERR040090:0.00000100)0.3836:0.00000100,ERR0467
73:0.00000100)0.4014:0.00043300,(((((((((ERR386789:0.00000100,ERR351901:0.00000100)0.08
67:0.00000100,ERR351857:0.00000100)0.1717:0.00000100,ERR386786:0.00000100)0.2333:0.0
0000100,ERR351854:0.00000100)0.2950:0.00000100,ERR386833:0.00000100)0.3427:0.000001
00,ERR403352:0.00000100)0.3789:0.00010800,ERR040133:0.00032400)0.3533:0.00000100,ER

R040126:0.00032400)0.3575:0.00000100,ERR040091:0.00032500)0.3678:0.00010800)0.3678:0
.00010800,((((((ERR019854:0.00000100,ERR017780:0.00000100)0.2967:0.00043300,(((((((((((((((
((((((((((((((((((((ERR038300:0.00010800,((((((((ERR351905:0.00000100,ERR386837:0.00000100)0
.0633:0.00000100,ERR072094:0.00000100)0.0967:0.00000100,ERR046970:0.00000100)0.1256:
0.00000100,ERR386792:0.00000100)0.1725:0.00000100,ERR046969:0.00000100)0.2080:0.000
00100,ERR046968:0.00000100)0.2339:0.00000100,ERR351860:0.00000100)0.2629:0.00000100
,ERR046965:0.00000100)0.2879:0.00010800)0.2619:0.00000100,ERR038266:0.00000100)0.239
7:0.00000100,ERR387048:0.00000100)0.2245:0.00000100,(ERR046857:0.00000100,ERR046858
:0.00000100)0.3400:0.00032400)0.2000:0.00000100,ERR040106:0.00000100)0.1898:0.000001
00,ERR046775:0.00000100)0.1802:0.00000100,ERR386894:0.00000100)0.1740:0.00000100,((E
RR403375:0.00010800,ERR025833:0.00000100)0.0967:0.00000100,ERR025834:0.00000100)0.2
400:0.00010800)0.1588:0.00000100,(ERR046982:0.00000100,ERR046983:0.00000100)0.2100:0
.00010800)0.1551:0.00000100,(ERR351864:0.00000100,ERR386796:0.00000100)0.3400:0.0003
2500)0.1546:0.00000100,ERR046889:0.00000100)0.1569:0.00000100,ERR387034:0.00021600)
0.1584:0.00000100,ERR038273:0.00000100)0.1609:0.00000100,ERR403349:0.00010800)0.167
0:0.00000100,ERR040089:0.00021600)0.1752:0.00000100,ERR046860:0.00000100)0.1829:0.00
000100,ERR046936:0.00000100)0.1926:0.00000100,(((ERR386793:0.00000100,ERR351861:0.00
000100)0.1333:0.00000100,ERR351862:0.00000100)0.2183:0.00000100,ERR386794:0.0000010
0)0.2822:0.00010800)0.2368:0.00000100,ERR038264:0.00000100)0.2484:0.00000100,ERR0401
07:0.00000100)0.2608:0.00000100,ERR046854:0.00000100)0.2693:0.00000100,ERR038299:0.0
0000100)0.2788:0.00000100,ERR046847:0.00000100)0.2898:0.00000100,ERR038272:0.000001
00)0.3010:0.00000100,ERR046866:0.00000100)0.3115:0.00000100,ERR038271:0.00000100)0.3
196:0.00000100,ERR038270:0.00000100)0.3274:0.00000100,ERR046845:0.00000100)0.3372:0.
00000100,ERR038269:0.00000100)0.3466:0.00000100,ERR387057:0.00000100)0.3556:0.00000
100,ERR046921:0.00000100)0.3635:0.00000100,ERR046867:0.00000100)0.3708:0.00000100,E
RR387047:0.00000100)0.3770:0.00000100,ERR046861:0.00000100)0.3823:0.00000100,ERR038
265:0.00000100)0.3902:0.00021600)0.3814:0.00000100,(ERR387002:0.00010800,(ERR386838:
0.00000100,ERR351906:0.00000100)0.2367:0.00021600)0.2983:0.00010800)0.3745:0.0000010
0,(ERR040093:0.00021600,ERR040086:0.00000100)0.2633:0.00032400)0.3759:0.00000100,((ER
R072065:0.00000100,ERR072077:0.00000100)0.2100:0.00000100,ERR072080:0.00000100)0.36
33:0.00043300)0.3824:0.00000100,ERR387062:0.00043300)0.3851:0.00010800)0.3993:0.0005
4100,(ERR351845:0.00000100,ERR386777:0.00000100)0.3433:0.00140900)0.3998:0.00043300)
0.3828:0.00000100,(((ERR229991:0.00000100,ERR137191:0.0000
0100)0.2633:0.00064900,(ERR403350:0.00130000,((ERR046895:0.00000100,ERR046846:0.0000
0100)0.1967:0.00097500,ERR386933:0.00119100)0.3200:0.00043300)0.3667:0.00043300)0.22
47:0.00000100,(((ERR133818:0.00010800,ERR229941:0.00021600)0.2933:0.00075800,ERR1084
49:0.00054100)0.3117:0.00000100,ERR229938:0.00064900)0.3656:0.00021600)0.1633:0.0000
0100,ERR133987:0.00010800)0.1470:0.00000100,(((((ERR067627:0.00000100,(((((((ERR133934:
0.00000100,((((ERR403274:0.00021600,ERR067682:0.00000100)0.0767:0.00000100,ERR403317
:0.00021600)0.1967:0.00000100,ERR067640:0.00010800)0.3033:0.00000100,ERR133828:0.000
10800)0.3675:0.00000100)0.4227:0.00043300,ERR137213:0.00054100)0.3944:0.00010800,(((E
RR158612:0.00000100,ERR027469:0.00000100)0.2867:0.00010800,ERR067756:0.00010800)0.4
167:0.00065000,(ERR386828:0.00000100,ERR351896:0.00000100)0.2967:0.00064900)0.3750:0
.00010800)0.3288:0.00000100,(ERR137261:0.00054100,ERR386884:0.00086600)0.0867:0.0000

0100)0.3482:0.00000100,((ERR229956:0.00000100,ERR229948:0.00000100)0.2833:0.00065000
,ERR228000:0.00032400)0.4067:0.00054100)0.3950:0.00021600,(((ERR228057:0.00000100,ERR
137197:0.00000100)0.2500:0.00032500,ERR403260:0.00021600)0.3450:0.00054100,(ERR1339
09:0.00000100,ERR403261:0.00000100)0.2800:0.00119100)0.3575:0.00010800)0.3589:0.0000
0100,(ERR108448:0.00054100,(((ERR133881:0.00064900,ERR144629:0.00043300)0.2233:0.000
10800,ERR133835:0.00108300)0.2917:0.00000100,ERR133836:0.00054100)0.3956:0.00010800
)0.4100:0.00054100)0.3947:0.00021600)0.3801:0.00000100,ERR133812:0.00010800)0.3665:0.
00000100,((ERR072035:0.00000100,(((ERR047011:0.00000100,ERR072019:0.00000100)0.1500:
0.00000100,ERR047009:0.00000100)0.2383:0.00000100,ERR046998:0.00000100)0.3333:0.000
00100)0.3858:0.00032500,(ERR351919:0.00000100,ERR386851:0.00000100)0.3000:0.0007580
0)0.3461:0.00010800)0.2936:0.00000100,(ERR047884:0.00401700,(((ERR386842:0.00000100,E
RR351910:0.00000100)0.2100:0.00000100,ERR351908:0.00000100)0.3350:0.00000100,ERR386
840:0.00000100)0.4089:0.00314700)0.3642:0.00010700)0.2573:0.00000100,(((((((((((((((((((((((((
((((((((((((((((((((((((((((((((((((ERR046763:0.00000100,ERR038284:0.00000100)0.0300:0.00000100,
ERR038254:0.00000100)0.0700:0.00000100,ERR046759:0.00000100)0.0922:0.00000100,ERR38
7037:0.00000100)0.1208:0.00000100,ERR046762:0.00000100)0.1367:0.00000100,ERR038255:
0.00000100)0.1583:0.00000100,ERR046755:0.00000100)0.1962:0.00000100,ERR038283:0.000
00100)0.2363:0.00000100,ERR386926:0.00000100)0.2559:0.00000100,ERR072045:0.00000100
)0.2797:0.00000100,ERR072050:0.00000100)0.3076:0.00010800,ERR040098:0.00000100)0.283
3:0.00000100,ERR046768:0.00000100)0.2636:0.00000100,ERR046820:0.00000100)0.2479:0.00
000100,ERR072042:0.00000100)0.2333:0.00000100,ERR040096:0.00010800)0.2194:0.0000010
0,ERR046760:0.00000100)0.2073:0.00000100,ERR072051:0.00000100)0.1972:0.00000100,ERR
040105:0.00000100)0.1874:0.00000100,ERR046925:0.00000100)0.1802:0.00000100,ERR04675
1:0.00000100)0.1725:0.00000100,ERR072046:0.00000100)0.1670:0.00000100,ERR040099:0.00
000100)0.1622:0.00000100,ERR040102:0.00000100)0.1564:0.00000100,ERR072041:0.0000010
0)0.1525:0.00000100,ERR025837:0.00000100)0.1485:0.00000100,ERR046926:0.00000100)0.14
48:0.00000100,ERR046876:0.00000100)0.1429:0.00000100,ERR386975:0.00010800)0.1403:0.0
0000100,ERR040103:0.00000100)0.1402:0.00000100,ERR040100:0.00010800)0.1411:0.000001
00,ERR386997:0.00000100)0.1429:0.00000100,ERR046766:0.00000100)0.1444:0.00000100,ER
R046877:0.00000100)0.1476:0.00000100,ERR046879:0.00000100)0.1510:0.00000100,ERR0400
95:0.00000100)0.1538:0.00000100,ERR046767:0.00000100)0.1576:0.00000100,ERR046920:0.0
0000100)0.1612:0.00000100,ERR403386:0.00000100)0.1682:0.00000100,ERR046756:0.000001
00)0.1737:0.00000100,ERR072044:0.00000100)0.1807:0.00000100,ERR387050:0.00000100)0.1
861:0.00000100,ERR387028:0.00000100)0.1918:0.00000100,ERR040094:0.00000100)0.1973:0.
00000100,ERR072048:0.00000100)0.2028:0.00000100,ERR046761:0.00000100)0.2088:0.00000
100,ERR046878:0.00000100)0.2137:0.00000100,ERR072047:0.00000100)0.2200:0.00000100,E
RR046764:0.00000100)0.2248:0.00000100,ERR046765:0.00000100)0.2303:0.00000100,ERR046
758:0.00000100)0.2352:0.00000100,ERR046752:0.00000100)0.2402:0.00000100,ERR046753:0.
00000100)0.2458:0.00000100,ERR046927:0.00000100)0.2514:0.00000100,ERR046928:0.00000
100)0.2553:0.00000100,ERR403388:0.00000100)0.2603:0.00000100,ERR046754:0.00000100)0.
2650:0.00000100,ERR040101:0.00000100)0.2693:0.00000100,ERR025838:0.00000100)0.2746:
0.00000100,ERR040097:0.00000100)0.2792:0.00000100,ERR040104:0.00000100)0.2832:0.000
10800)0.1981:0.00000100)0.1789:0.00000100,(((((((((((((((ERR386872:0.00000100,ERR403378:
0.00000100)0.0333:0.00000100,ERR386871:0.00000100)0.0700:0.00000100,ERR351940:0.000

00100)0.1044:0.00000100,ERR403368:0.00000100)0.1600:0.00000100,ERR403347:0.00000100)0.1953:0.00000100,ERR351939:0.00000100)0.2278:0.00000100,ERR403357:0.00000100)0.2590:0.00000100,ERR403358:0.00000100)0.2938:0.00000100,ERR403372:0.00010800)0.3111:0.00000100,(ERR386868:0.00000100,ERR351936:0.00000100)0.1800:0.00010800)0.3509:0.00000100,ERR403338:0.00000100)0.3781:0.00000100,ERR403328:0.00000100)0.4064:0.00173400,(((((((((ERR046834:0.00000100,ERR017798:0.00000100)0.1500:0.00000100,ERR046981:0.00000100)0.2283:0.00000100,ERR386937:0.00010800)0.3156:0.00010800,ERR403400:0.00000100)0.2867:0.00000100,ERR386887:0.00064900)0.2587:0.00000100,((ERR351912:0.00000100,ERR386844:0.00000100)0.2800:0.00010800,ERR017793:0.00000100)0.3500:0.00021600)0.2925:0.00000100,(ERR047004:0.00000100,ERR046996:0.00000100)0.2667:0.00021600)0.3460:0.00000100,ERR351929:0.00000100)0.3706:0.00000100,ERR386861:0.00000100)0.4011:0.00043300)0.2655:0.00000100,((((((ERR351882:0.00000100,ERR386814:0.00000100)0.3000:0.00043300,((ERR484743:0.00010800,ERR038738:0.00032500)0.2800:0.00021600,ERR403325:0.00054100)0.2733:0.00000100)0.4025:0.00010800,ERR386996:0.00054200)0.4180:0.00032500,(((((((ERR038739:0.00000100,ERR038748:0.00000100)0.2233:0.00010800,ERR040127:0.00000100)0.3233:0.00032400,ERR038740:0.00032500)0.3578:0.00010800,(ERR038749:0.00043300,ERR040125:0.00054100)0.1500:0.00000100)0.4027:0.00043300,(ERR038750:0.00021600,ERR040135:0.00000100)0.2933:0.00086600)0.3600:0.00000100,ERR040131:0.00064900)0.3767:0.00000100,ERR386875:0.00054100)0.4081:0.00054100)0.4069:0.00087000,ERR386911:0.00271400)0.3975:0.00010600,(ERR386896:0.00043300,(ERR386898:0.00000100,ERR387023:0.00000100)0.2833:0.00075800)0.3650:0.00162600)0.3433:0.00000100)0.2886:0.00000100,(ERR386967:0.00162500,(ERR046839:0.00000100,ERR046838:0.00000100)0.2433:0.00173400)0.1483:0.00000100)0.2955:0.00010800)0.1391:0.00000100,(ERR144604:0.00075800,((ERR144569:0.00010800,ERR067597:0.00000100)0.1833:0.00000100,ERR403280:0.00032500)0.3433:0.00021600)0.3211:0.00010800)0.1357:0.00000100,ERR229923:0.00000100)0.1349:0.00000100,(ERR067674:0.00010800,((((ERR403318:0.00000100,((ERR133936:0.00000100,ERR137259:0.00000100)0.1400:0.00000100,ERR067598:0.00000100)0.2383:0.00000100)0.3422:0.00010800,ERR108447:0.00000100)0.3292:0.00000100,ERR137242:0.00000100)0.3420:0.00000100,ERR137240:0.00010800)0.3728:0.00010800)0.3586:0.00010800)0.1289:0.00000100,((ERR386964:0.00336100,ERR387044:0.00379600)0.2067:0.00000100,(((ERR351915:0.00000100,ERR386847:0.00000100)0.2733:0.00249500,ERR386999:0.00260400)0.3733:0.00010500,((ERR351883:0.00000100,ERR386815:0.00000100)0.3133:0.00130000,ERR386891:0.00206000)0.3850:0.00021500)0.4100:0.00097400)0.4052:0.00043300)0.1234:0.00000100,(ERR387032:0.00075800,(ERR072027:0.00000100,ERR047003:0.00000100)0.2900:0.00021600)0.3633:0.00054200)0.1215:0.00000100,ERR403215:0.00000100)0.1208:0.00000100,ERR137205:0.00000100)0.1202:0.00000100,ERR108472:0.00000100)0.1195:0.00000100,(((ERR133894:0.00000100,ERR234624:0.00000100)0.1033:0.00000100,ERR133976:0.00000100)0.1750:0.00000100,ERR229935:0.00000100)0.2578:0.00010800)0.1172:0.00000100,ERR403324:0.00000100)0.1166:0.00000100,ERR108423:0.00000100)0.1160:0.00000100,((((ERR387049:0.00000100,ERR386959:0.00000100)0.1233:0.00000100,ERR072026:0.00000100)0.2117:0.00000100,ERR387031:0.00000100)0.2978:0.00000100,ERR072040:0.00000100)0.3833:0.00043300)0.1131:0.00000100,ERR108435:0.00000100)0.1126:0.00000100,ERR133842:0.00000100)0.1120:0.00000100,(ERR046872:0.00000100,ERR046873:0.00000100)0.2900:0.00032500)0.1109:0.00000100,(((ERR046743:0.00000100,(((((((((ERR046746:0.00000100,(((((((((((((ERR046737:0.00000100,ERR046971:0.00000100)0.0167:0.00000100,ERR046744:0.00000100)0.0383:0.000001

00,ERR039329:0.00000100)0.0667:0.00000100,ERR046964:0.00000100)0.0925:0.00000100,ER
R351889:0.00000100)0.1093:0.00000100,ERR046735:0.00000100)0.1322:0.00000100,ERR0467
45:0.00010800)0.1424:0.00000100,ERR386821:0.00000100)0.1542:0.00000100,ERR046986:0.0
0000100)0.1715:0.00000100,ERR046840:0.00000100)0.1973:0.00000100,ERR046943:0.000001
00)0.2179:0.00000100,ERR046825:0.00000100)0.2383:0.00000100,ERR046923:0.00000100)0.2
654:0.00000100,ERR039327:0.00000100)0.2890:0.00000100)0.3073:0.00000100,ERR046734:0.
00000100)0.3304:0.00000100,ERR046924:0.00000100)0.3508:0.00010800,ERR046960:0.00000
100)0.3456:0.00000100,ERR046738:0.00000100)0.3442:0.00000100,(((ERR039328:0.00000100,
ERR046841:0.00000100)0.1100:0.00000100,ERR046819:0.00000100)0.2083:0.00000100,ERR04
6950:0.00000100)0.2967:0.00010800)0.3536:0.00000100,ERR040109:0.00000100)0.3625:0.00
000100,ERR046842:0.00000100)0.3757:0.00000100,ERR046733:0.00000100)0.3897:0.0000010
0)0.4025:0.00021600,ERR046736:0.00000100)0.3974:0.00000100,ERR046741:0.00000100)0.40
36:0.00064900)0.0993:0.00000100,(((ERR234645:0.00000100,ERR019572:0.00000100)0.2667:0
.00010800,ERR067582:0.00000100)0.3667:0.00032500,ERR133861:0.00021600)0.3189:0.0001
0800)0.0976:0.00000100,((((ERR067646:0.00010800,ERR133946:0.00010800)0.0900:0.0000010
0,ERR137268:0.00000100)0.1467:0.00000100,ERR117458:0.00010800)0.2011:0.00000100,ERR
108501:0.00000100)0.2717:0.00010800)0.0957:0.00000100,(ERR403245:0.00010800,ERR1372
27:0.00021600)0.1800:0.00010800)0.0949:0.00000100,((((((((((((((((((((((ERR137195:0.0000010
0,ERR108486:0.00000100)0.0067:0.00000100,ERR228004:0.00000100)0.0217:0.00000100,ERR
234557:0.00000100)0.0433:0.00000100,ERR117465:0.00010800)0.0525:0.00000100,ERR13387
1:0.00000100)0.0687:0.00000100,ERR144552:0.00000100)0.0811:0.00000100,ERR108430:0.00
000100)0.0810:0.00000100,ERR067604:0.00000100)0.0917:0.00000100,ERR228010:0.0000010
0)0.1022:0.00000100,ERR144582:0.00021600)0.1060:0.00000100,ERR228018:0.00010800)0.11
97:0.00000100,ERR234642:0.00000100)0.1275:0.00000100,ERR067766:0.00000100)0.1451:0.0
0000100,ERR027445:0.00000100)0.1602:0.00000100,ERR117449:0.00000100)0.1742:0.000001
00,ERR137207:0.00000100)0.1898:0.00000100,ERR403275:0.00000100)0.2016:0.00000100,ER
R228015:0.00000100)0.2152:0.00000100,ERR234652:0.00000100)0.2282:0.00000100,ERR2280
60:0.00000100)0.2442:0.00000100,ERR133820:0.00000100)0.2560:0.00000100,ERR403304:0.0
0000100)0.2670:0.00010800)0.0871:0.00000100,(((((ERR067596:0.00000100,ERR227997:0.000
00100)0.1067:0.00000100,ERR144624:0.00000100)0.1750:0.00000100,ERR137233:0.00010800
)0.2600:0.00000100,ERR158614:0.00000100)0.3433:0.00000100,ERR234693:0.00000100)0.396
0:0.00043300)0.0853:0.00000100,(ERR067621:0.00000100,ERR067630:0.00000100)0.1567:0.0
0010800)0.0847:0.00000100,ERR229998:0.00000100)0.0844:0.00000100,ERR403233:0.000325
00)0.0841:0.00000100,(((((ERR386998:0.00000100,ERR351921:0.00000100)0.2200:0.00000100
,ERR386853:0.00000100)0.3783:0.00086600,(((ERR386961:0.00000100,ERR386913:0.00000100
)0.1533:0.00000100,ERR386904:0.00000100)0.2833:0.00000100,ERR386910:0.00000100)0.418
9:0.00108300)0.3828:0.00010800,(ERR403348:0.00043300,ERR386931:0.00064900)0.1833:0.0
0010800)0.3683:0.00000100,ERR403381:0.00043300)0.3800:0.00021600)0.0812:0.00000100,E
RR484733:0.00000100)0.0809:0.00000100,ERR403305:0.00000100)0.0807:0.00000100,(((ERR0
19862:0.00000100,ERR017788:0.00000100)0.2700:0.00010800,ERR158608:0.00000100)0.2967
:0.00010800,((ERR228032:0.00000100,(((ERR067593:0.00000100,ERR067722:0.00000100)0.11
67:0.00000100,ERR144631:0.00000100)0.2117:0.00000100,ERR133852:0.00000100)0.3100:0.0
0000100)0.3742:0.00021600,ERR158572:0.00000100)0.3847:0.00065000)0.2450:0.00000100)0
.0784:0.00000100,ERR133975:0.00000100)0.0782:0.00000100,ERR144556:0.00000100)0.0779:

0.00000100,ERR144635:0.00000100)0.0777:0.00000100,ERR137283:0.00000100)0.0774:0.000
00100,ERR228017:0.00000100)0.0771:0.00000100,((ERR133903:0.00000100,ERR137284:0.000
00100)0.2167:0.00010800,ERR137209:0.00000100)0.2550:0.00010800)0.0764:0.00000100,ERR
133956:0.00010800)0.0761:0.00000100,ERR158592:0.00000100)0.0759:0.00000100,ERR13394
4:0.00000100)0.0757:0.00000100)0.0924:0.00000100,ERR234663:0.00021600)0.0922:0.00000
100,((((((ERR228067:0.00162500,ERR387046:0.00184100)0.0567:0.00000100,((ERR046897:0.00
000100,ERR046901:0.00000100)0.3067:0.00075700,((ERR047013:0.00000100,ERR072037:0.00
000100)0.2500:0.00000100,ERR072038:0.00000100)0.3600:0.00054100)0.2892:0.00000100)0.
2933:0.00000100,(((ERR108481:0.00000100,ERR228025:0.00000100)0.3067:0.00054100,ERR13
3974:0.00032400)0.3167:0.00000100,SRR14508916:0.00054100)0.3889:0.00032500)0.3930:0.
00021600,(((ERR038754:0.00000100,ERR040130:0.00000100)0.1500:0.00000100,ERR040124:0.
00000100)0.2967:0.00000100,ERR040128:0.00000100)0.4111:0.00119200)0.4124:0.00065100,
(((ERR039343:0.00000100,ERR038282:0.00000100)0.2267:0.00000100,ERR046781:0.00000100)
0.3550:0.00108400,ERR386984:0.00054100)0.3722:0.00184500)0.3843:0.00010800,ERR04788
5:0.00271000)0.3642:0.00000100)0.0883:0.00000100,ERR234618:0.00000100)0.0881:0.00000
100,(((ERR144546:0.00000100,ERR133857:0.00000100)0.1933:0.00000100,ERR133905:0.00000
100)0.3717:0.00119200,ERR403377:0.00151700)0.3800:0.00021600)0.0874:0.00000100,((((ER
R017779:0.00140800,ERR403406:0.00108200)0.1933:0.00000100,(ERR351873:0.00000100,ERR
386805:0.00000100)0.3267:0.00064900)0.3367:0.00000100,ERR386906:0.00086600)0.4117:0.
00032500,ERR387014:0.00130000)0.3847:0.00010800)0.0862:0.00000100,((((((ERR067617:0.0
0000100,ERR403315:0.00010800)0.2700:0.00064900,(ERR108459:0.00032500,ERR067616:0.00
043300)0.1333:0.00000100)0.3300:0.00010800,((ERR108482:0.00000100,ERR229932:0.000001
00)0.2333:0.00032500,ERR230003:0.00032500)0.2300:0.00000100)0.3839:0.00032500,(ERR13
7228:0.00000100,ERR229925:0.00000100)0.2833:0.00064900)0.3167:0.00000100,ERR137256:
0.00064900)0.3063:0.00000100,((ERR133877:0.00021600,ERR133850:0.00010800)0.1567:0.00
000100,ERR144576:0.00075800)0.2783:0.00010800)0.3528:0.00000100,ERR228038:0.0002160
0)0.3654:0.00021600)0.0839:0.00000100,ERR158607:0.00010800)0.0837:0.00000100,((((((((ER
R387006:0.00000100,ERR387027:0.00000100)0.2433:0.00000100,ERR403403:0.00000100)0.37
33:0.00032400,ERR386919:0.00054100)0.4022:0.00108300,((((ERR386921:0.00000100,ERR433
208:0.00000100)0.1567:0.00000100,ERR403393:0.00000100)0.2233:0.00000100,ERR387022:0.
00000100)0.3233:0.00000100,ERR403365:0.00000100)0.4117:0.00184100)0.2917:0.00000100,
((((((ERR046941:0.00000100,ERR046939:0.00000100)0.0867:0.00000100,(ERR351871:0.000001
00,ERR386803:0.00000100)0.1933:0.00010800)0.2233:0.00000100,ERR072072:0.00000100)0.2
808:0.00000100,(ERR072029:0.00000100,ERR072025:0.00000100)0.2167:0.00010800)0.3333:0
.00000100,ERR046730:0.00010800)0.3800:0.00000100,ERR039324:0.00000100)0.4188:0.0007
5800)0.2888:0.00000100,(((ERR351898:0.00000100,ERR386830:0.00000100)0.2867:0.0007580
0,((ERR386801:0.00000100,ERR351869:0.00000100)0.3133:0.00075800,ERR403363:0.0004330
0)0.3117:0.00000100)0.4208:0.00064900,(ERR386813:0.00000100,ERR351881:0.00000100)0.2
367:0.00162500)0.3789:0.00010800)0.3429:0.00010800,(((((ERR386994:0.00054100,(ERR3868
35:0.00000100,ERR351903:0.00000100)0.2933:0.00075700)0.2550:0.00000100,((ERR038260:0.
00000100,ERR038261:0.00000100)0.2633:0.00086600,(ERR038263:0.00000100,ERR038262:0.0
0000100)0.3133:0.00097400)0.3022:0.00000100)0.4117:0.00021600,(((ERR038753:0.00000100
,(ERR040123:0.00000100,ERR038752:0.00010800)0.1467:0.00000100)0.2750:0.00000100,ERR0
40119:0.00000100)0.4011:0.00054100,ERR403380:0.00097400)0.3833:0.00010800)0.3527:0.0

0000100,(ERR040114:0.00054100,ERR403354:0.00054100)0.2100:0.00010800)0.3908:0.00000
100,ERR386945:0.00054100)0.4131:0.00032500)0.3238:0.00000100,(ERR403342:0.00097500,E
RR386947:0.00195100)0.2600:0.00032400)0.3289:0.00010800)0.0775:0.00000100,ERR067720:
0.00000100)0.0773:0.00000100,ERR234655:0.00010800)0.0772:0.00000100,((((ERR117466:0.0
0000100,ERR067664:0.00010800)0.0667:0.00000100,ERR229983:0.00043300)0.1117:0.000001
00,ERR108422:0.00000100)0.1900:0.00000100,(ERR228009:0.00054100,ERR227990:0.0002160
0)0.2033:0.00010800)0.2627:0.00010800)0.0764:0.00000100,ERR230008:0.00000100)0.0762:0
.00000100,(ERR067757:0.00043300,((ERR144603:0.00000100,ERR108437:0.00000100)0.2167:0
.00010800,(ERR046935:0.00000100,ERR046892:0.00000100)0.2733:0.00108300)0.1411:0.0000
0100)0.1067:0.00000100)0.0756:0.00000100,ERR108434:0.00000100)0.0754:0.00000100)0.06
98:0.00000100,(((ERR072023:0.00000100,ERR072024:0.00000100)0.3167:0.00108400,(ERR351
852:0.00032400,ERR386968:0.00000100)0.2500:0.00032400)0.3822:0.00140900,(ERR108490:0
.00000100,ERR117455:0.00000100)0.1967:0.00000100)0.3393:0.00010800)0.0692:0.00000100
,((((ERR229947:0.00010800,ERR229997:0.00010800)0.0767:0.00000100,ERR133935:0.0000010
0)0.1267:0.00000100,ERR117462:0.00010800)0.1933:0.00000100,ERR133867:0.00000100)0.23
42:0.00010800)0.0686:0.00000100,(ERR144616:0.00010800,ERR228002:0.00000100)0.1767:0.
00010800)0.0684:0.00000100,(((((((ERR234599:0.00000100,ERR403292:0.00010800)0.0800:0.0
0000100,ERR229990:0.00000100)0.1483:0.00000100,ERR067763:0.00021600)0.1656:0.000001
00,ERR403231:0.00000100)0.2200:0.00000100,ERR229937:0.00010800)0.2760:0.00000100,(ER
R133830:0.00000100,ERR229942:0.00000100)0.2133:0.00010800)0.3510:0.00000100,ERR0676
85:0.00000100)0.3925:0.00043300)0.0675:0.00000100,(((((ERR046848:0.00000100,ERR046898
:0.00000100)0.1667:0.00000100,ERR387009:0.00000100)0.3017:0.00000100,ERR386943:0.000
00100)0.4078:0.00108200,ERR403373:0.00173300)0.4050:0.00217200,((ERR038258:0.0000010
0,ERR038259:0.00000100)0.2700:0.00292600,(ERR046844:0.00000100,ERR046893:0.00000100
)0.2900:0.00325200)0.3111:0.00000100)0.3683:0.00032500)0.0666:0.00000100)0.0660:0.0000
0100,ERR227980:0.00000100)0.0659:0.00000100)0.0643:0.00000100,ERR067652:0.00000100)
0.0642:0.00000100,(((ERR067601:0.00010800,ERR067727:0.00010800)0.1333:0.00000100,ERR
137258:0.00000100)0.2333:0.00000100,ERR144621:0.00000100)0.3322:0.00021600)0.0639:0.
00000100,ERR133960:0.00000100)0.0638:0.00000100,ERR067721:0.00000100)0.0637:0.00000
100,ERR067691:0.00000100)0.0635:0.00000100,((ERR386941:0.00086600,(((ERR387042:0.000
75700,((ERR403397:0.00000100,ERR386899:0.00000100)0.2500:0.00043300,((ERR351849:0.00
000100,ERR386781:0.00000100)0.2600:0.00010800,ERR403394:0.00000100)0.3817:0.0007570
0)0.3475:0.00000100)0.3713:0.00010800,(ERR386780:0.00000100,ERR403421:0.00000100)0.3
133:0.00064900)0.3876:0.00010800,(ERR351872:0.00000100,ERR386804:0.00000100)0.3167:0
.00064900)0.4037:0.00021700)0.3800:0.00000100,((ERR387000:0.00000100,ERR386822:0.000
00100)0.2233:0.00000100,ERR351890:0.00000100)0.3567:0.00054100)0.3959:0.00021600)0.0
625:0.00000100,ERR403314:0.00000100)0.0624:0.00000100,((((ERR133798:0.00000100,ERR13
3863:0.00000100)0.1967:0.00010800,ERR133988:0.00032500)0.1833:0.00000100,(ERR234683:
0.00000100,ERR234657:0.00000100)0.2567:0.00010800)0.2842:0.00000100,ERR108505:0.000
00100)0.2920:0.00010800)0.0620:0.00000100,ERR386972:0.00054100)0.0619:0.00000100,ERR
158575:0.00000100)0.0618:0.00000100,(ERR072032:0.00000100,ERR072031:0.00000100)0.29
00:0.00064900)0.0617:0.00000100,((ERR351916:0.00000100,ERR386848:0.00000100)0.2767:0.
00021600,((ERR403335:0.00000100,ERR351907:0.00000100)0.2167:0.00000100,ERR386839:0.
00000100)0.3833:0.00054100)0.3442:0.00010800)0.0614:0.00000100,ERR117470:0.00000100)

0.0613:0.00000100,(ERR403289:0.00000100,ERR144571:0.00010800)0.1733:0.00010800)0.061
2:0.00000100,ERR133802:0.00000100)0.0611:0.00000100,ERR229977:0.00000100)0.0610:0.00
000100,((ERR108457:0.00064900,ERR133893:0.00021600)0.1500:0.00010800,ERR133833:0.00
075800)0.0767:0.00000100)0.0608:0.00000100,ERR234561:0.00021600)0.0607:0.00000100,ER
R133978:0.00064900)0.0606:0.00000100,((ERR067607:0.00000100,ERR133811:0.00000100)0.1
300:0.00000100,ERR228030:0.00000100)0.2750:0.00010800)0.0604:0.00000100,ERR403269:0.
00010800)0.0603:0.00000100,ERR229982:0.00000100)0.0602:0.00000100)0.0598:0.00000100,
ERR133937:0.00000100)0.0597:0.00000100,ERR229929:0.00010800)0.0597:0.00000100,((ERR3
86800:0.00000100,ERR351868:0.00000100)0.2333:0.00021600,(ERR351914:0.00000100,ERR38
6846:0.00000100)0.3033:0.00097400)0.3900:0.00043300)0.0595:0.00000100,(ERR133834:0.00
021600,ERR067615:0.00000100)0.1800:0.00010800)0.0593:0.00000100,ERR403295:0.0000010
0)0.0592:0.00000100,ERR067676:0.00000100)0.0592:0.00000100,ERR228005:0.00021600)0.05
91:0.00000100,(ERR351877:0.00000100,ERR386809:0.00000100)0.2800:0.00054100)0.0590:0.
00000100,(((ERR229958:0.00075800,ERR386973:0.00108300)0.2733:0.00054100,ERR144578:0.
00205900)0.3700:0.00021700,ERR133870:0.00151600)0.3444:0.00010800)0.0588:0.00000100)
0.0584:0.00000100,ERR144625:0.00000100)0.0583:0.00000100,ERR019564:0.00000100)0.058
2:0.00000100,ERR403250:0.00000100)0.0582:0.00000100,ERR137244:0.00000100)0.0581:0.00
000100,ERR234602:0.00000100)0.0580:0.00000100,ERR234690:0.00000100)0.0579:0.0000010
0,(ERR067576:0.00000100,ERR228029:0.00000100)0.1533:0.00010800)0.0578:0.00000100,ERR
017783:0.00000100)0.0577:0.00000100,(ERR108450:0.00010800,(ERR133829:0.00010800,ERR
228045:0.00000100)0.2833:0.00249300)0.1417:0.00000100)0.0576:0.00000100,ERR386890:0.
00000100)0.0575:0.00000100,ERR484734:0.00000100)0.0574:0.00000100,((ERR067753:0.0008
6600,ERR229972:0.00032500)0.1100:0.00000100,(ERR133809:0.00000100,ERR133804:0.00000
100)0.2600:0.00032500)0.3456:0.00021600)0.0572:0.00000100)0.0569:0.00000100,ERR23464
9:0.00010800)0.0569:0.00000100)0.0568:0.00000100,ERR137262:0.00000100)0.0567:0.00000
100,(((ERR351856:0.00000100,ERR386788:0.00000100)0.3067:0.00075800,(((ERR351876:0.000
00100,ERR386811:0.00000100)0.1633:0.00000100,ERR386808:0.00000100)0.2850:0.00000100
,ERR351879:0.00000100)0.4056:0.00130000)0.3907:0.00010800,(((((ERR403362:0.00086600,(((
ERR351887:0.00000100,ERR386819:0.00000100)0.2200:0.00000100,ERR387060:0.00000100)0.
3500:0.00108300,ERR386957:0.00054100)0.3989:0.00032500)0.3317:0.00000100,(ERR386963:
0.00086600,ERR387029:0.00119100)0.1967:0.00010800)0.3161:0.00000100,(ERR403361:0.000
00100,ERR387015:0.00000100)0.3233:0.00043300)0.3713:0.00010800,(ERR351874:0.0000010
0,ERR386806:0.00000100)0.3300:0.00151700)0.3717:0.00010800,ERR017802:0.00108300)0.36
06:0.00000100)0.3982:0.00032500)0.0560:0.00000100,ERR228061:0.00000100)0.0560:0.0000
0100,ERR234697:0.00064900)0.0559:0.00000100,ERR229984:0.00000100)0.0558:0.00000100,
ERR133945:0.00000100)0.0558:0.00000100,ERR403247:0.00010800)0.0557:0.00000100)0.055
6:0.00000100,ERR067707:0.00000100)0.0555:0.00000100,ERR230010:0.00000100)0.0554:0.00
000100,(ERR234631:0.00054100,((((((ERR025844:0.00000100,ERR025843:0.00000100)0.1300:0
.00000100,ERR046922:0.00000100)0.2033:0.00000100,ERR072095:0.00000100)0.2644:0.0000
0100,ERR025846:0.00000100)0.3175:0.00000100,ERR025842:0.00000100)0.3593:0.00000100,
ERR039345:0.00000100)0.4128:0.00216700)0.3538:0.00000100)0.0555:0.00000100,ERR13382
6:0.00000100)0.0554:0.00000100,ERR144596:0.00010800)0.0554:0.00000100,ERR067613:0.00
000100)0.0553:0.00000100,ERR234632:0.00000100)0.0552:0.00000100,ERR144574:0.0000010
0)0.0552:0.00000100,(((((ERR046995:0.00000100,ERR046999:0.00000100)0.3200:0.00021600,E

RR038747:0.00043300)0.3300:0.00000100,ERR038742:0.00021600)0.3889:0.00097400,ERR403294:0.00097400)0.3383:0.00000100,ERR108442:0.00119100)0.3300:0.00010800)0.0550:0.00000100,ERR067587:0.00000100)0.0549:0.00000100,(((ERR403390:0.00032500,ERR019565:0.00021600)0.0967:0.00000100,(ERR019573:0.00043300,(ERR351895:0.00000100,ERR386827:0.00000100)0.2167:0.00010800)0.3000:0.00010800)0.3392:0.00000100,ERR158609:0.00032500)0.3680:0.00021600)0.0547:0.00000100,ERR067609:0.00010800)0.0547:0.00000100,((ERR137245:0.00000100,ERR137239:0.00000100)0.2567:0.00021600,ERR108456:0.00010800)0.2733:0.00010800)0.0546:0.00000100,ERR133933:0.00000100)0.0545:0.00000100,ERR108428:0.00000100)0.0545:0.00000100,(ERR403366:0.00032500,ERR386986:0.00000100)0.2667:0.00032500)0.0544:0.00000100,ERR234586:0.00000100)0.0543:0.00000100,ERR133908:0.00010800)0.0543:0.00000100,ERR133855:0.00010800)0.0542:0.00000100,((((ERR351886:0.00000100,ERR386824:0.00000100)0.1667:0.00000100,ERR351892:0.00000100)0.2917:0.00000100,ERR386818:0.00000100)0.4011:0.00097500,ERR386942:0.00043300)0.4117:0.00075800)0.0542:0.00000100,ERR133958:0.00000100)0.0541:0.00000100,ERR067632:0.00032500)0.0541:0.00000100,(((ERR351928:0.00000100,ERR386860:0.00000100)0.2267:0.00054100,ERR403359:0.00043300)0.2800:0.00010800,ERR144577:0.00021600)0.2789:0.00010800)0.0540:0.00000100,((ERR144542:0.00000100,ERR133900:0.00000100)0.2067:0.00010800,(ERR133888:0.00000100,ERR133815:0.00000100)0.2633:0.00021600)0.2767:0.00010800)0.0539:0.00000100,ERR144590:0.00000100)0.0538:0.00000100,(ERR403276:0.00021600,((((ERR067647:0.00000100,(ERR027451:0.00000100,ERR158616:0.00000100)0.2300:0.00010800)0.2250:0.00000100,ERR234658:0.00000100)0.2900:0.00000100,ERR144567:0.00000100)0.3633:0.00000100,ERR228062:0.00000100)0.4080:0.00129900)0.3406:0.00000100)0.0539:0.00000100,ERR067719:0.00000100)0.0538:0.00000100)0.0537:0.00000100,ERR133942:0.00000100)0.0536:0.00000100,ERR387036:0.00021600)0.0536:0.00000100,ERR227988:0.00021600)0.0536:0.00000100,ERR228046:0.00000100)0.0535:0.00000100,ERR403286:0.00000100)0.0534:0.00000100,(((((ERR229940:0.00000100,ERR234569:0.00010800)0.0600:0.00000100,ERR137212:0.00000100)0.1350:0.00000100,ERR133814:0.00000100)0.1978:0.00000100,ERR117469:0.00010800)0.2417:0.00010800,ERR067600:0.00075800)0.1940:0.00000100)0.0533:0.00000100,ERR403264:0.00000100)0.0533:0.00000100,ERR019575:0.00000100)0.0532:0.00000100,ERR137267:0.00010800)0.0532:0.00000100,ERR108426:0.00000100)0.0531:0.00000100,ERR067618:0.00000100)0.0531:0.00000100,(ERR386915:0.00043300,ERR386936:0.00043300)0.2767:0.00032500)0.0531:0.00000100,((ERR386925:0.00075800,ERR387017:0.00075800)0.2767:0.00043300,ERR234689:0.00064900)0.2167:0.00000100)0.0530:0.00000100,ERR228043:0.00021600)0.0530:0.00000100,(ERR386940:0.00086600,((ERR046890:0.00000100,ERR046831:0.00000100)0.3100:0.00075800,(ERR039341:0.00000100,ERR039342:0.00000100)0.2800:0.00130000)0.3822:0.00010800)0.4000:0.00075800)0.0530:0.00000100,ERR227979:0.00000100)0.0530:0.00000100,ERR067755:0.00000100)0.0529:0.00000100,ERR234581:0.00000100)0.0529:0.00000100,ERR067689:0.00000100)0.0528:0.00000100,ERR133848:0.00010800)0.0528:0.00000100,ERR133904:0.00000100)0.0528:0.00000100,ERR403223:0.00000100)0.0527:0.00000100)0.0527:0.00000100,ERR234620:0.00000100)0.0526:0.00000100,((ERR027464:0.00043300,ERR386885:0.00054100)0.2867:0.00021600,ERR386902:0.00043300)0.3433:0.00032500)0.0526:0.00000100,((((ERR234614:0.00000100,ERR133962:0.00000100)0.2233:0.00000100,ERR229917:0.00000100)0.3500:0.00010800,ERR144544:0.00086600)0.3389:0.00000100,ERR403321:0.00000100)0.3750:0.00032500)0.0526:0.00000100,((ERR403251:0.00010800,(((ERR067661:0.00054100,ERR133858:0.00032500)0.2533:0.00010800,ERR133967:0.00075700)0.2950:0.00000

100,ERR117451:0.00043300)0.3689:0.00010800)0.3892:0.00010800,ERR137214:0.00097500)0.
3953:0.00021700)0.0526:0.00000100,(ERR229916:0.00000100,ERR067649:0.00000100)0.1900:
0.00010800)0.0526:0.00000100,ERR067739:0.00000100)0.0525:0.00000100,ERR403273:0.000
00100)0.0525:0.00000100,(ERR403364:0.00021600,ERR386917:0.00021600)0.1767:0.0001080
0)0.0525:0.00000100,ERR158611:0.00000100)0.0524:0.00000100,(ERR228007:0.00021600,((ER
R017794:0.00000100,ERR019868:0.00000100)0.2800:0.00097500,(ERR038292:0.00000100,ERR
038291:0.00000100)0.2567:0.00032400)0.3967:0.00097500)0.2975:0.00000100)0.0525:0.0000
0100,ERR144611:0.00000100)0.0525:0.00000100,ERR067698:0.00000100)0.0524:0.00000100,
ERR234627:0.00000100)0.0524:0.00000100,ERR144587:0.00010800)0.0524:0.00000100,ERR06
7714:0.00000100)0.0524:0.00000100,ERR117453:0.00000100)0.0524:0.00000100,((ERR067657
:0.00010800,ERR137196:0.00000100)0.2667:0.00043300,ERR386939:0.00140800)0.1333:0.000
00100)0.0524:0.00000100,ERR227977:0.00000100)0.0524:0.00000100)0.0524:0.00000100,ERR
133941:0.00000100)0.0524:0.00000100,(ERR108506:0.00000100,ERR229920:0.00000100)0.28
67:0.00054100)0.0524:0.00000100,ERR227975:0.00000100)0.0524:0.00000100,ERR234646:0.0
0000100)0.0524:0.00000100,ERR144636:0.00000100)0.0524:0.00000100,(ERR234637:0.00000
100,ERR137223:0.00000100)0.2100:0.00010800)0.0524:0.00000100,ERR228050:0.00000100)0.
0524:0.00000100,(ERR067670:0.00021600,(((ERR025848:0.00000100,ERR039344:0.00000100)0
.2100:0.00000100,ERR025847:0.00000100)0.3867:0.00043300,(ERR386932:0.00000100,ERR40
3370:0.00010800)0.2900:0.00021600)0.4208:0.00086700)0.3367:0.00000100)0.0526:0.000001
00,ERR234617:0.00000100)0.0526:0.00000100,ERR133851:0.00043300)0.0526:0.00000100,ER
R144615:0.00000100)0.0526:0.00000100,ERR133873:0.00000100)0.0525:0.00000100,(ERR351
885:0.00000100,ERR386817:0.00000100)0.3000:0.00140800)0.0527:0.00000100,ERR117456:0.
00010800)0.0527:0.00000100,ERR133880:0.00000100)0.0527:0.00000100,((((ERR108446:0.000
43300,(ERR067747:0.00000100,ERR108487:0.00010800)0.3200:0.00043300)0.2417:0.0000010
0,((((ERR144547:0.00021600,ERR108451:0.00010800)0.1100:0.00000100,ERR108483:0.000001
00)0.2150:0.00000100,ERR228031:0.00000100)0.3244:0.00000100,ERR067767:0.00000100)0.3
883:0.00021600)0.3538:0.00000100,ERR067729:0.00021600)0.3858:0.00021600,((ERR046997:
0.00000100,ERR072039:0.00000100)0.2867:0.00086600,ERR133897:0.00075800)0.3667:0.001
84300)0.2836:0.00000100)0.0532:0.00000100,ERR234559:0.00000100)0.0532:0.00000100,(ER
R234684:0.00052900,M_canettii_SNPs:0.19594400)0.0133:0.00001300)0.0533:0.00000100,(((E
RR046821:0.00000100,ERR047886:0.00000100)0.1467:0.00000100,ERR046903:0.00000100)0.2
717:0.00000100,ERR046796:0.00000100)0.3956:0.00065000)0.0535:0.00000100,ERR234560:0.
00010800)0.0535:0.00000100)0.0540:0.00000100,ERR027458:0.00000100)0.0539:0.00000100,
(ERR144573:0.00000100,ERR234696:0.00054100)0.2000:0.00010800)0.0541:0.00000100,ERR0
67590:0.00000100)0.0540:0.00000100,ERR137192:0.00010800)0.0540:0.00000100)0.0543:0.0
0000100,ERR108475:0.00000100)0.0543:0.00000100,(ERR038290:0.00000100,ERR038293:0.00
000100)0.2900:0.00086600)0.0544:0.00000100,ERR386948:0.00032500)0.0544:0.00000100,ER
R137224:0.00064900)0.0545:0.00000100,ERR067671:0.00021600)0.0545:0.00000100,(ERR117
450:0.00000100,ERR137280:0.00000100)0.2733:0.00032500)0.0546:0.00000100,ERR234687:0.
00043300)0.0547:0.00000100,ERR137246:0.00010800)0.0547:0.00000100,((ERR351855:0.0000
0100,ERR386787:0.00000100)0.3100:0.00054100,(ERR387024:0.00000100,ERR386979:0.00000
100)0.2667:0.00043300)0.3600:0.00021700)0.0550:0.00000100,ERR027450:0.00010800)0.055
0:0.00000100,ERR229919:0.00097500)0.0550:0.00000100,ERR403256:0.00000100)0.0550:0.00
000100,ERR067728:0.00000100)0.0550:0.00000100,ERR133886:0.00000100)0.0550:0.0000010

0,ERR227981:0.00000100)0.0551:0.00000100,((ERR067680:0.00097500,ERR067579:0.0005410
0)0.2367:0.00010800,(ERR133807:0.00043300,ERR137238:0.00054100)0.1633:0.00010800)0.3
478:0.00021600)0.0556:0.00000100,(ERR067580:0.00010800,ERR027449:0.00000100)0.1667:0
.00010800)0.0557:0.00000100,ERR230007:0.00000100)0.0557:0.00000100,ERR234698:0.0000
0100)0.0557:0.00000100,ERR403323:0.00032500)0.0558:0.00000100,(((ERR230004:0.0000010
0,ERR158593:0.00000100)0.1900:0.00000100,ERR133924:0.00021600)0.3017:0.00010800,ERR
108479:0.00054100)0.3033:0.00010800)0.0561:0.00000100)0.0566:0.00000100,ERR403293:0.
00000100)0.0566:0.00000100,ERR158578:0.00000100)0.0567:0.00000100,((ERR403409:0.0013
0000,ERR387021:0.00216900)0.3100:0.00075800,(ERR108497:0.00000100,ERR133921:0.00000
100)0.3033:0.00086600)0.1644:0.00000100)0.0573:0.00000100,(ERR133959:0.00021600,(ERR1
33952:0.00054100,(ERR351858:0.00000100,ERR386790:0.00000100)0.2967:0.00086600)0.150
0:0.00000100)0.1000:0.00000100)0.0578:0.00000100,ERR386862:0.00000100)0.0579:0.00000
100,ERR228028:0.00000100)0.0579:0.00000100,(((ERR133891:0.00000100,ERR133925:0.00000
100)0.1300:0.00000100,ERR234664:0.00000100)0.2267:0.00010800,ERR403396:0.00162500)0.
1511:0.00000100)0.0583:0.00000100,ERR403230:0.00000100)0.0584:0.00000100,(((ERR38679
5:0.00000100,ERR351863:0.00000100)0.2567:0.00021600,ERR387059:0.00043300)0.3183:0.00
010800,ERR158604:0.00119200)0.3444:0.00021700)0.0590:0.00000100,((ERR403310:0.000108
00,(ERR234638:0.00000100,ERR137241:0.00000100)0.2667:0.00021600)0.2850:0.00000100,ER
R067686:0.00000100)0.3900:0.00054100)0.0595:0.00000100,ERR234556:0.00000100)0.0596:0
.00000100,ERR403258:0.00043300)0.0597:0.00000100,ERR158615:0.00000100)0.0598:0.0000
0100,(((((((ERR403389:0.00000100,(ERR047012:0.00000100,ERR039335:0.00000100)0.0867:0.0
0000100)0.1550:0.00000100,ERR047010:0.00000100)0.2078:0.00000100,ERR039336:0.000001
00)0.2792:0.00000100,ERR039338:0.00000100)0.3387:0.00000100,ERR386977:0.00000100)0.3
811:0.00000100,ERR039337:0.00000100)0.4062:0.00151700,ERR067606:0.00097500)0.3888:0.
00010800)0.0615:0.00000100,ERR067641:0.00000100)0.0616:0.00000100,(ERR229943:0.0004
3300,(ERR067629:0.00000100,ERR067748:0.00000100)0.3133:0.00075800)0.1583:0.00000100)
0.0621:0.00000100,(ERR067752:0.00000100,ERR019562:0.00010800)0.1833:0.00010800)0.062
5:0.00000100,ERR067639:0.00000100)0.0626:0.00000100,ERR234600:0.00000100)0.0627:0.00
000100,ERR234635:0.00000100)0.0629:0.00000100,ERR133932:0.00000100)0.0630:0.0000010
0,ERR484740:0.00000100)0.0631:0.00000100,ERR144575:0.00010800)0.0633:0.00000100,ERR
144561:0.00010800)0.0635:0.00000100)0.0643:0.00000100,ERR234669:0.00000100)0.0645:0.
00000100,ERR067706:0.00000100)0.0646:0.00000100,((ERR387063:0.00097400,(((ERR386798:
0.00000100,ERR351902:0.00000100)0.1500:0.00000100,ERR386834:0.00000100)0.2433:0.000
00100,ERR351866:0.00000100)0.3811:0.00021600)0.3342:0.00000100,ERR386955:0.00043300
)0.3373:0.00010800)0.0663:0.00000100,ERR108453:0.00032500)0.0666:0.00000100,ERR40326
8:0.00000100)0.0669:0.00000100,ERR108498:0.00000100)0.0672:0.00000100,ERR229964:0.00
000100)0.0674:0.00000100)0.0687:0.00000100,ERR229922:0.00000100)0.0690:0.00000100,((((
(ERR017801:0.00000100,ERR019875:0.00000100)0.2900:0.00043200,ERR386988:0.00075700)0
.3567:0.00021600,ERR386983:0.00064900)0.3767:0.00010800,(ERR046933:0.00000100,ERR04
6918:0.00000100)0.2733:0.00097300)0.4113:0.00456300,ERR019570:0.00075900)0.3856:0.00
010700)0.0719:0.00000100,ERR067581:0.00000100)0.0723:0.00000100,ERR067638:0.0000010
0)0.0727:0.00000100,ERR133837:0.00000100)0.0730:0.00000100,ERR137236:0.00000100)0.07
33:0.00000100,ERR229927:0.00000100)0.0737:0.00000100,ERR067693:0.00000100)0.0741:0.0
0000100,ERR234619:0.00021600)0.0745:0.00000100,ERR403240:0.00000100)0.0750:0.000001

00,ERR386970:0.00086600)0.0755:0.00000100,ERR144548:0.00000100)0.0759:0.00000100,ER
R137270:0.00054100)0.0764:0.00000100,ERR387039:0.00065000)0.0768:0.00000100,ERR1445
92:0.00064900)0.0774:0.00000100,ERR144545:0.00000100)0.0778:0.00000100,(ERR403254:0.
00021600,ERR108433:0.00054100)0.2300:0.00021600)0.0789:0.00000100,(((ERR234692:0.000
00100,ERR403235:0.00000100)0.1000:0.00000100,ERR133896:0.00000100)0.2117:0.00000100
,ERR403232:0.00000100)0.2567:0.00010800)0.0809:0.00000100,(((((ERR017787:0.00043300,E
RR386881:0.00000100)0.1133:0.00000100,(ERR351920:0.00000100,ERR386852:0.00000100)0.
2933:0.00021600)0.3022:0.00000100,ERR038746:0.00021600)0.3317:0.00000100,ERR387011:
0.00000100)0.4167:0.00054100,ERR386952:0.00086600)0.3833:0.00010800)0.0848:0.0000010
0,(ERR137282:0.00000100,ERR067696:0.00000100)0.2067:0.00010800)0.0860:0.00000100,((((E
RR067735:0.00000100,ERR108478:0.00000100)0.2033:0.00000100,ERR108504:0.00000100)0.3
650:0.00075800,((((ERR234699:0.00000100,ERR067678:0.00000100)0.1767:0.00000100,ERR06
7602:0.00010800)0.2900:0.00000100,ERR227994:0.00000100)0.4167:0.00064900,ERR108508:
0.00054100)0.4083:0.00021600)0.4029:0.00043300,ERR229950:0.00064900)0.3529:0.0000010
0)0.0913:0.00000100,ERR137220:0.00010800)0.0919:0.00000100,(ERR403367:0.00010800,ERR
403418:0.00000100)0.2833:0.00032500)0.0933:0.00000100,((ERR229954:0.00043300,ERR1372
02:0.00032400)0.3100:0.00032500,ERR403331:0.00010800)0.3150:0.00010800)0.0952:0.0000
0100,((ERR386923:0.00097400,ERR386990:0.00119100)0.2600:0.00043300,ERR386892:0.0015
1700)0.3300:0.00032500)0.0972:0.00000100,ERR133800:0.00000100)0.0978:0.00000100,ERR0
19553:0.00010800)0.0985:0.00000100,((ERR040132:0.00065000,ERR403398:0.00010800)0.116
7:0.00000100,ERR386880:0.00119200)0.2233:0.00010800)0.1005:0.00000100,(ERR133827:0.0
0000100,ERR133866:0.00000100)0.1733:0.00010800)0.1018:0.00000100,ERR229933:0.000001
00)0.1025:0.00000100,ERR234633:0.00000100)0.1031:0.00000100,ERR403248:0.00032500)0.1
037:0.00000100,((ERR386992:0.00000100,ERR403327:0.00021600)0.2067:0.00010800,((ERR02
5840:0.00000100,ERR025839:0.00000100)0.2300:0.00010800,ERR019571:0.00000100)0.3233:
0.00021600)0.3258:0.00010800)0.1071:0.00000100,ERR137271:0.00000100)0.1077:0.0000010
0,(ERR229957:0.00000100,ERR158570:0.00000100)0.1867:0.00010800)0.1092:0.00000100,ERR
144618:0.00000100)0.1099:0.00000100,ERR228012:0.00010800)0.1105:0.00000100)0.1133:0.
00000100,ERR108421:0.00000100)0.1140:0.00000100,(ERR133882:0.00000100,ERR158569:0.0
0000100)0.2467:0.00021600)0.1153:0.00000100,ERR158577:0.00000100)0.1159:0.00000100,E
RR144553:0.00000100)0.1165:0.00000100,ERR158597:0.00000100)0.1172:0.00000100,ERR229
978:0.00000100)0.1179:0.00000100,ERR067754:0.00000100)0.1186:0.00000100,ERR067645:0.
00000100)0.1193:0.00000100,ERR403300:0.00000100)0.1200:0.00000100,((((ERR108463:0.000
64900,(ERR229963:0.00000100,ERR229967:0.00010800)0.2667:0.00032500)0.3117:0.0001080
0,(ERR108468:0.00043300,ERR108466:0.00086600)0.0733:0.00000100)0.3425:0.00000100,ERR
234694:0.00043300)0.3960:0.00043300,ERR403330:0.00108300)0.3639:0.00010800)0.1248:0.
00000100,ERR137229:0.00000100)0.1254:0.00000100,(ERR234625:0.00021600,((ERR046875:0.
00000100,ERR046874:0.00000100)0.2400:0.00021600,ERR234588:0.00173400)0.1250:0.00000
100)0.0833:0.00000100)0.1280:0.00000100,ERR108441:0.00000100)0.1286:0.00000100,ERR14
4584:0.00010800)0.1292:0.00000100,(ERR228048:0.00000100,ERR067608:0.00000100)0.1900:
0.00010800)0.1305:0.00000100,ERR227991:0.00032500)0.1312:0.00000100,ERR158589:0.000
00100)0.1319:0.00000100,((ERR067745:0.00000100,ERR133970:0.00000100)0.2333:0.0000010
0,ERR117459:0.00000100)0.3617:0.00064900)0.1339:0.00000100,ERR067605:0.00000100)0.13
45:0.00000100,ERR137225:0.00000100)0.1351:0.00000100,ERR144585:0.00108300)0.1358:0.0

0000100,ERR133838:0.00000100)0.1365:0.00000100,((ERR072030:0.00000100,ERR047014:0.0
0000100)0.1833:0.00000100,ERR047007:0.00000100)0.3667:0.00065000)0.1384:0.00000100,E
RR403290:0.00000100)0.1391:0.00000100,ERR133939:0.00000100)0.1398:0.00000100,ERR144
613:0.00000100)0.1405:0.00000100,ERR158591:0.00000100)0.1412:0.00000100,(ERR229985:0
.00021600,(((((((ERR039339:0.00000100,ERR046929:0.00000100)0.0933:0.00000100,ERR0469
52:0.00000100)0.1283:0.00000100,ERR046951:0.00000100)0.1711:0.00000100,ERR046948:0.0
0000100)0.2425:0.00000100,ERR046930:0.00000100)0.3120:0.00000100,ERR046932:0.000001
00)0.3383:0.00000100,ERR039340:0.00000100)0.3690:0.00000100,ERR046953:0.00000100)0.3
954:0.00119100)0.3515:0.00000100)0.1481:0.00000100,ERR137235:0.00000100)0.1488:0.000
00100,ERR234630:0.00000100)0.1494:0.00000100,ERR484742:0.00000100)0.1500:0.00000100
,ERR228054:0.00000100)0.1507:0.00000100,ERR117467:0.00000100)0.1514:0.00000100,ERR1
37218:0.00010800)0.1520:0.00000100,ERR234583:0.00000100)0.1527:0.00000100,ERR403278
:0.00010800)0.1534:0.00000100,ERR403267:0.00000100)0.1541:0.00000100,ERR067733:0.000
00100)0.1548:0.00000100,ERR227996:0.00000100)0.1554:0.00000100,ERR144581:0.00010800
)0.1561:0.00000100,ERR403283:0.00000100)0.1567:0.00000100)0.1616:0.00000100,ERR15858
5:0.00000100)0.1622:0.00000100,ERR144580:0.00000100)0.1628:0.00000100,ERR228024:0.00
000100)0.1635:0.00000100,((ERR386863:0.00000100,ERR351931:0.00000100)0.2833:0.000649
00,ERR403407:0.00032500)0.2783:0.00010800)0.1654:0.00000100,ERR133968:0.00000100)0.1
660:0.00000100,((ERR403322:0.00000100,ERR133846:0.00000100)0.1733:0.00000100,ERR067
585:0.00000100)0.3233:0.00021600)0.1679:0.00000100,ERR144627:0.00000100)0.1686:0.000
00100,ERR403303:0.00000100)0.1693:0.00000100,ERR133926:0.00000100)0.1699:0.00000100
,ERR234593:0.00000100)0.1705:0.00000100,(((ERR386929:0.00000100,ERR386966:0.00000100
)0.3733:0.00032400,ERR047881:0.00021700)0.4083:0.00108300,ERR040138:0.00075700)0.343
3:0.00000100)0.1732:0.00000100,ERR067717:0.00000100)0.1739:0.00000100,ERR108496:0.00
064900)0.1745:0.00000100,ERR229924:0.00000100)0.1752:0.00000100,ERR067762:0.0000010
0)0.1758:0.00000100,ERR117464:0.00000100)0.1765:0.00000100)0.1777:0.00000100,ERR0677
13:0.00000100)0.1784:0.00000100,ERR229980:0.00000100)0.1791:0.00000100,ERR108480:0.0
0000100)0.1798:0.00000100,(ERR403344:0.00303600,ERR144634:0.00010800)0.0000:0.00000
100)0.1810:0.00000100,ERR386934:0.00000100)0.1817:0.00000100)0.1830:0.00000100,ERR22
7987:0.00000100)0.1836:0.00000100,ERR067692:0.00000100)0.1843:0.00000100,ERR228044:
0.00054100)0.1849:0.00000100,ERR108500:0.00000100)0.1856:0.00000100,((ERR137193:0.00
000100,ERR234695:0.00000100)0.3133:0.00108300,ERR108493:0.00010800)0.1567:0.0000010
0)0.1876:0.00000100,ERR133983:0.00000100)0.1882:0.00000100,ERR137219:0.00000100)0.18
89:0.00000100,ERR067737:0.00065000)0.1896:0.00000100,ERR403311:0.00000100)0.1902:0.0
0000100,ERR158586:0.00000100)0.1909:0.00000100,ERR227999:0.00000100)0.1916:0.000001
00,ERR137272:0.00000100)0.1922:0.00000100,ERR144600:0.00000100)0.1928:0.00000100,ER
R108488:0.00000100)0.1935:0.00000100,ERR144606:0.00000100)0.1941:0.00000100,ERR4032
91:0.00043300)0.1948:0.00000100,ERR067636:0.00000100)0.1954:0.00000100,ERR351880:0.0
0000100)0.1961:0.00000100,ERR403225:0.00021600)0.1968:0.00000100,ERR158590:0.000001
00)0.1974:0.00000100,ERR067626:0.00000100)0.1981:0.00000100,ERR137204:0.00000100)0.1
987:0.00000100,((ERR158584:0.00032500,ERR229944:0.00021600)0.1033:0.00000100,(ERR137
201:0.00000100,ERR158595:0.00000100)0.2067:0.00010800)0.2622:0.00010800)0.2012:0.000
00100,ERR108491:0.00000100)0.2019:0.00000100,ERR229970:0.00000100)0.2025:0.00000100
,ERR386928:0.00021600)0.2032:0.00000100,ERR230000:0.00000100)0.2038:0.00000100,ERR4

03411:0.00000100)0.2045:0.00000100)0.2076:0.00000100,ERR387053:0.00000100)0.2083:0.0
0000100,ERR228027:0.00021600)0.2089:0.00000100,ERR067594:0.00000100)0.2095:0.000001
00,ERR484748:0.00000100)0.2101:0.00000100,(ERR019858:0.00000100,ERR017784:0.0000010
0)0.1800:0.00010800)0.2113:0.00000100,ERR133806:0.00000100)0.2120:0.00000100,ERR1084
73:0.00010800)0.2127:0.00000100,ERR108452:0.00000100)0.2133:0.00000100,ERR137254:0.0
0000100)0.2139:0.00000100,(ERR234603:0.00043300,ERR137253:0.00021600)0.2267:0.00021
600)0.2151:0.00000100,ERR067644:0.00000100)0.2157:0.00000100,((ERR025836:0.00000100,
ERR025835:0.00000100)0.2833:0.00043300,(ERR351867:0.00000100,ERR386799:0.00000100)0
.3267:0.00075800)0.3911:0.00032500)0.2183:0.00000100,ERR137237:0.00010800)0.2190:0.00
000100,ERR234639:0.00000100)0.2196:0.00000100,ERR133810:0.00000100)0.2202:0.0000010
0,ERR144593:0.00000100)0.2208:0.00000100,ERR234570:0.00000100)0.2214:0.00000100,ERR
133920:0.00064900)0.2220:0.00000100,ERR403262:0.00000100)0.2226:0.00000100,((ERR0468
88:0.00000100,ERR046833:0.00000100)0.2867:0.00140900,ERR133808:0.00075800)0.3217:0.0
0021600)0.2243:0.00000100,(ERR228003:0.00010800,ERR108461:0.00097400)0.0000:0.00000
100)0.2256:0.00000100,ERR067635:0.00000100)0.2262:0.00000100,ERR158602:0.00000100)0.
2268:0.00000100,((ERR386791:0.00000100,ERR351859:0.00000100)0.2500:0.00140800,ERR38
7058:0.00086600)0.3500:0.00054100)0.2286:0.00000100,ERR234596:0.00000100)0.2292:0.00
000100,(ERR067660:0.00021600,ERR386989:0.00195000)0.0033:0.00000100)0.2304:0.000001
00,ERR108474:0.00000100)0.2310:0.00000100,ERR403316:0.00032500)0.2316:0.00000100,ER
R234573:0.00010800)0.2322:0.00000100,(ERR234647:0.00000100,ERR234640:0.00000100)0.2
767:0.00054100)0.2334:0.00000100,ERR144607:0.00000100)0.2340:0.00000100,((ERR144614:
0.00010800,ERR137273:0.00021600)0.3167:0.00065000,ERR228022:0.00032500)0.2883:0.000
10800)0.2358:0.00000100,ERR144597:0.00000100)0.2364:0.00000100,ERR144594:0.00043300
)0.2369:0.00000100,ERR144557:0.00000100)0.2375:0.00000100,ERR108499:0.00021600)0.238
2:0.00000100,ERR108462:0.00010800)0.2388:0.00000100,ERR067583:0.00000100)0.2394:0.00
000100,ERR108455:0.00000100)0.2399:0.00000100,ERR403306:0.00000100)0.2405:0.0000010
0,ERR067760:0.00000100)0.2411:0.00000100,ERR158576:0.00000100)0.2417:0.00000100,(ERR
403224:0.00000100,ERR229961:0.00000100)0.1533:0.00010800)0.2429:0.00000100,ERR11745
7:0.00000100)0.2435:0.00000100,ERR229921:0.00000100)0.2440:0.00000100,(ERR228026:0.0
0054100,ERR228063:0.00064900)0.0033:0.00000100)0.2451:0.00000100,ERR229949:0.000001
00)0.2457:0.00000100,ERR108485:0.00010800)0.2464:0.00000100,ERR228042:0.00000100)0.2
470:0.00000100,ERR403216:0.00000100)0.2476:0.00000100,ERR067611:0.00010800)0.2482:0.
00000100,ERR133862:0.00000100)0.2488:0.00000100,ERR117454:0.00010800)0.2494:0.00000
100,ERR133918:0.00000100)0.2500:0.00000100,ERR144595:0.00000100)0.2505:0.00000100,E
RR137243:0.00000100)0.2511:0.00000100,ERR133839:0.00000100)0.2517:0.00000100,ERR403
296:0.00000100)0.2523:0.00000100)0.2540:0.00000100,ERR067592:0.00010800)0.2545:0.000
00100,ERR403218:0.00000100)0.2552:0.00000100,ERR234660:0.00000100)0.2557:0.00000100
,ERR067577:0.00000100)0.2563:0.00000100,ERR229939:0.00010800)0.2569:0.00000100,ERR4
03272:0.00000100)0.2575:0.00000100,ERR137234:0.00000100)0.2581:0.00000100,ERR067743
:0.00021600)0.2586:0.00000100,ERR229960:0.00010800)0.2592:0.00000100,ERR067603:0.000
00100)0.2598:0.00000100,ERR234590:0.00010800)0.2603:0.00000100,ERR046882:0.00000100
)0.2609:0.00000100,ERR144562:0.00010800)0.2615:0.00000100,ERR234584:0.00000100)0.262
0:0.00000100,ERR144559:0.00010800)0.2625:0.00000100,ERR067595:0.00000100)0.2632:0.00
000100,ERR133911:0.00000100)0.2636:0.00000100,ERR227984:0.00010800)0.2641:0.0000010

0,ERR234666:0.00000100)0.2647:0.00000100,ERR108432:0.00043300)0.2652:0.00000100,ERR
229931:0.00000100)0.2658:0.00000100,(ERR403308:0.00000100,ERR228011:0.00010800)0.21
33:0.00010800)0.2669:0.00000100,ERR133854:0.00010800)0.2675:0.00000100,(ERR133883:0.
00000100,ERR067730:0.00000100)0.2967:0.00043300)0.2687:0.00000100,(ERR133989:0.0011
9100,ERR230001:0.00075800)0.1433:0.00010800)0.2698:0.00000100,(ERR228059:0.00000100,
ERR158580:0.00010800)0.1367:0.00010800)0.2709:0.00000100,ERR234653:0.00010800)0.271
4:0.00000100)0.2725:0.00000100,ERR133973:0.00000100)0.2731:0.00000100,ERR403320:0.00
000100)0.2736:0.00000100,ERR403238:0.00000100)0.2742:0.00000100,ERR234565:0.0000010
0)0.2747:0.00000100,ERR230006:0.00000100)0.2753:0.00000100,ERR234607:0.00000100)0.27
58:0.00000100,(ERR067708:0.00064900,ERR108427:0.00054100)0.2767:0.00054100)0.2767:0.
00000100,ERR067697:0.00000100)0.2773:0.00000100,ERR144632:0.00000100)0.2778:0.00000
100,ERR403319:0.00054100)0.2784:0.00000100,(ERR351899:0.00000100,ERR386831:0.000001
00)0.2967:0.00065000)0.2795:0.00000100,ERR067715:0.00000100)0.2800:0.00000100,((ERR14
4572:0.00086600,ERR403237:0.00010800)0.1867:0.00010800,ERR158574:0.00032500)0.2583:
0.00010800)0.2816:0.00000100,ERR117452:0.00000100)0.2821:0.00000100,(ERR067612:0.000
86700,(ERR046894:0.00000100,ERR046887:0.00000100)0.2833:0.00119100)0.3450:0.0003250
0)0.2838:0.00000100,ERR144623:0.00000100)0.2844:0.00000100,(ERR386779:0.00000100,ERR
351847:0.00000100)0.2500:0.00064900)0.2854:0.00000100,ERR144570:0.00000100)0.2860:0.
00000100,ERR144565:0.00021600)0.2865:0.00000100,ERR158573:0.00010800)0.2871:0.00000
100,ERR137230:0.00000100)0.2877:0.00000100,ERR067687:0.00010800)0.2882:0.00000100,E
RR067711:0.00000100)0.2888:0.00000100,ERR403299:0.00000100)0.2893:0.00000100,(ERR04
6863:0.00000100,ERR046862:0.00000100)0.3100:0.00075800)0.2904:0.00000100,ERR228001:
0.00000100)0.2909:0.00000100,ERR144555:0.00086600)0.2915:0.00000100,ERR067667:0.000
00100)0.2919:0.00000100,ERR067659:0.00000100)0.2925:0.00000100,ERR067654:0.00000100
)0.2930:0.00000100,ERR137216:0.00010800)0.2935:0.00000100,ERR403255:0.00054100)0.294
0:0.00000100,ERR133951:0.00000100)0.2946:0.00000100,(ERR234662:0.00000100,ERR234610
:0.00000100)0.1867:0.00010800)0.2957:0.00000100,ERR234575:0.00000100)0.2962:0.000001
00,ERR229999:0.00000100)0.2968:0.00000100,ERR046934:0.00130000)0.2973:0.00000100,ER
R229915:0.00010800)0.2978:0.00000100,ERR133869:0.00000100)0.2983:0.00000100,ERR1338
98:0.00000100)0.2988:0.00000100,ERR403353:0.00097500)0.2993:0.00000100,ERR137275:0.0
0000100)0.2998:0.00000100,ERR403244:0.00000100)0.3003:0.00000100,ERR158581:0.000108
00)0.3008:0.00000100,(ERR133892:0.00021600,ERR144599:0.00010800)0.2800:0.00054100)0.
3018:0.00000100,ERR137264:0.00000100)0.3023:0.00000100,ERR228039:0.00000100)0.3028:
0.00000100,ERR067619:0.00000100)0.3033:0.00000100,ERR133915:0.00000100)0.3039:0.000
00100,ERR108502:0.00000100)0.3044:0.00000100,ERR017785:0.00000100)0.3049:0.00000100
,ERR137247:0.00000100)0.3053:0.00000100,ERR133953:0.00000100)0.3058:0.00000100,ERR1
08512:0.00010800)0.3063:0.00000100,ERR133950:0.00000100)0.3068:0.00000100,ERR234623
:0.00000100)0.3073:0.00000100,ERR108515:0.00000100)0.3078:0.00000100,ERR387007:0.000
32500)0.3084:0.00000100,ERR229993:0.00000100)0.3090:0.00000100,ERR228064:0.00010800
)0.3095:0.00000100,ERR117463:0.00000100)0.3100:0.00000100,ERR228068:0.00043300)0.310
5:0.00000100,ERR133902:0.00000100)0.3111:0.00000100,ERR234648:0.00000100)0.3116:0.00
000100,ERR133884:0.00000100)0.3121:0.00000100,ERR108425:0.00000100)0.3127:0.0000010
0)0.3137:0.00000100,ERR108439:0.00000100)0.3141:0.00000100,(ERR351938:0.00000100,ERR
386870:0.00000100)0.2133:0.00021600)0.3151:0.00000100,ERR403263:0.00000100)0.3156:0.

00000100,ERR403242:0.00021600)0.3161:0.00000100,ERR403287:0.00000100)0.3166:0.00000
100,ERR108513:0.00000100)0.3171:0.00000100,ERR234608:0.00000100)0.3176:0.00000100,E
RR484739:0.00000100)0.3180:0.00000100,ERR137217:0.00000100)0.3185:0.00000100,ERR133
805:0.00000100)0.3190:0.00000100,ERR158601:0.00000100)0.3196:0.00000100,ERR133901:0.
00000100)0.3200:0.00000100,ERR386958:0.00000100)0.3204:0.00000100,(ERR133865:0.0000
0100,ERR234585:0.00000100)0.2633:0.00021600)0.3214:0.00000100,ERR133947:0.00000100)
0.3219:0.00000100,ERR484741:0.00000100)0.3224:0.00000100,(ERR229981:0.00000100,(ERR1
44605:0.00000100,ERR234616:0.00000100)0.2500:0.00010800)0.2867:0.00010800)0.3238:0.0
0000100,ERR117460:0.00000100)0.3243:0.00000100,ERR108467:0.00000100)0.3248:0.000001
00,ERR137248:0.00010800)0.3253:0.00000100,ERR133969:0.00021600)0.3257:0.00000100,ER
R351925:0.00000100)0.3262:0.00000100,ERR228052:0.00010800)0.3267:0.00000100,ERR1338
89:0.00000100)0.3272:0.00000100,ERR484745:0.00000100)0.3277:0.00000100,ERR017789:0.0
0075800)0.3283:0.00000100,ERR144558:0.00000100)0.3287:0.00000100,ERR227976:0.000001
00)0.3292:0.00000100,ERR067746:0.00000100)0.3296:0.00000100,(ERR386916:0.00043300,((E
RR386841:0.00000100,ERR387030:0.00000100)0.2000:0.00000100,ERR351909:0.00000100)0.3
767:0.00021600)0.3944:0.00021600)0.3317:0.00000100,ERR108494:0.00000100)0.3322:0.000
00100,ERR144619:0.00000100)0.3326:0.00000100,ERR133878:0.00000100)0.3331:0.00000100
,ERR387043:0.00108200)0.3335:0.00000100,ERR234597:0.00000100)0.3340:0.00000100,ERR2
34579:0.00032500)0.3346:0.00000100,ERR229968:0.00000100)0.3351:0.00000100,ERR234643
:0.00000100)0.3355:0.00000100,ERR158579:0.00000100)0.3360:0.00000100,(ERR403417:0.00
010800,(ERR067677:0.00000100,ERR144598:0.00000100)0.2600:0.00108300)0.1317:0.000001
00)0.3374:0.00000100,ERR067684:0.00000100)0.3378:0.00000100,ERR144602:0.00021600)0.3
383:0.00000100,ERR234609:0.00000100)0.3388:0.00000100,ERR067634:0.00086600)0.3392:0.
00000100,ERR158596:0.00000100)0.3397:0.00000100,ERR137279:0.00010800)0.3402:0.00000
100,ERR133917:0.00000100)0.3408:0.00000100,ERR228023:0.00000100)0.3413:0.00000100,E
RR386971:0.00075800)0.3418:0.00000100,ERR133982:0.00000100)0.3422:0.00000100,ERR133
910:0.00000100)0.3427:0.00000100,ERR403284:0.00000100)0.3432:0.00000100,ERR027459:0.
00000100)0.3436:0.00000100,ERR133879:0.00000100)0.3441:0.00000100)0.3450:0.00000100,
ERR234582:0.00000100)0.3455:0.00000100,ERR403301:0.00000100)0.3459:0.00000100,ERR13
3949:0.00000100)0.3464:0.00000100,ERR387018:0.00097400)0.3469:0.00000100,ERR144588:
0.00000100)0.3473:0.00000100,ERR229994:0.00000100)0.3478:0.00000100,ERR067653:0.000
10800)0.3483:0.00000100,ERR234644:0.00000100)0.3488:0.00000100,ERR137274:0.00000100
)0.3492:0.00000100,ERR403241:0.00021600)0.3497:0.00000100,ERR403219:0.00000100)0.350
1:0.00000100,ERR229953:0.00010800)0.3506:0.00000100,ERR403416:0.00054100)0.3511:0.00
000100,ERR228020:0.00000100)0.3515:0.00000100,ERR067695:0.00010800)0.3520:0.0000010
0,ERR229969:0.00000100)0.3524:0.00000100,ERR067625:0.00000100)0.3528:0.00000100,ERR
067704:0.00021600)0.3533:0.00000100,ERR067650:0.00010800)0.3538:0.00000100,ERR22992
6:0.00000100)0.3542:0.00000100,ERR403410:0.00000100)0.3546:0.00000100,ERR133885:0.00
000100)0.3551:0.00000100,ERR067765:0.00000100)0.3556:0.00000100,ERR067631:0.0000010
0)0.3561:0.00000100,(ERR386826:0.00000100,ERR351894:0.00000100)0.2167:0.00010800)0.3
570:0.00000100,ERR234564:0.00000100)0.3575:0.00000100,ERR386857:0.00000100)0.3580:0.
00000100,ERR228037:0.00000100)0.3584:0.00000100,ERR403229:0.00000100)0.3588:0.00000
100,ERR108436:0.00010800)0.3592:0.00000100,ERR027455:0.00000100)0.3598:0.00000100,E
RR229918:0.00000100)0.3602:0.00000100,ERR067759:0.00000100)0.3607:0.00000100,ERR067

663:0.00010800)0.3612:0.00000100,ERR234568:0.00000100)0.3616:0.00000100,ERR133914:0.
00000100)0.3620:0.00000100)0.3629:0.00000100,ERR234628:0.00000100)0.3633:0.00000100,
((ERR158598:0.00000100,ERR137265:0.00000100)0.2033:0.00000100,ERR133966:0.00010800)
0.3300:0.00021600)0.3646:0.00000100,ERR144560:0.00000100)0.3651:0.00000100,ERR15858
8:0.00000100)0.3655:0.00000100,ERR144551:0.00000100)0.3660:0.00000100,ERR229955:0.00
000100)0.3664:0.00000100,ERR133890:0.00000100)0.3669:0.00000100,ERR403252:0.0001080
0)0.3674:0.00000100,ERR403243:0.00010800)0.3678:0.00000100,ERR227978:0.00010800)0.36
82:0.00000100,ERR144609:0.00000100)0.3687:0.00000100,ERR067710:0.00000100)0.3691:0.0
0000100,ERR234598:0.00010800)0.3695:0.00000100,ERR403285:0.00000100)0.3700:0.000001
00,ERR229966:0.00000100)0.3704:0.00000100,ERR108469:0.00000100)0.3709:0.00000100,ER
R067672:0.00000100)0.3714:0.00000100)0.3726:0.00000100,ERR067662:0.00010800)0.3730:0
.00000100,((ERR227992:0.00000100,ERR158582:0.00000100)0.2967:0.00054100,ERR387054:0.
00043300)0.1483:0.00000100)0.3743:0.00000100,ERR133864:0.00010800)0.3747:0.00000100,
(ERR229988:0.00000100,ERR234654:0.00000100)0.1667:0.00010800)0.3755:0.00000100)0.376
8:0.00000100,ERR229934:0.00000100)0.3773:0.00000100,ERR108484:0.00000100)0.3777:0.00
000100,(ERR228035:0.00000100,ERR228055:0.00010800)0.2133:0.00021600)0.3785:0.000001
00,ERR229975:0.00000100)0.3790:0.00000100,ERR229936:0.00000100)0.3794:0.00000100,ER
R019574:0.00086600)0.3798:0.00000100,ERR133859:0.00000100)0.3803:0.00000100,ERR2346
56:0.00000100)0.3807:0.00000100,ERR386901:0.00054100)0.3811:0.00000100,ERR108509:0.0
0032500)0.3816:0.00000100,ERR137231:0.00000100)0.3820:0.00000100,ERR133799:0.000001
00)0.3824:0.00000100,ERR133984:0.00000100)0.3828:0.00000100,ERR228019:0.00000100)0.3
833:0.00000100,ERR067651:0.00010800)0.3837:0.00000100,ERR144550:0.00000100)0.3841:0.
00000100,ERR133845:0.00032500)0.3846:0.00000100,ERR230005:0.00000100)0.3850:0.00000
100,ERR234572:0.00010800)0.3855:0.00000100,ERR137277:0.00000100)0.3859:0.00000100,E
RR234578:0.00075800)0.3864:0.00000100,ERR108429:0.00000100)0.3867:0.00000100,ERR158
613:0.00000100)0.3872:0.00000100,ERR158587:0.00000100)0.3875:0.00000100,ERR403266:0.
00010800)0.3879:0.00000100,ERR144566:0.00010800)0.3883:0.00000100,ERR403214:0.00000
100)0.3887:0.00000100,ERR403336:0.00032500)0.3891:0.00000100,ERR137222:0.00000100)0.
3896:0.00000100,ERR067731:0.00021600)0.3900:0.00000100,(ERR133913:0.00000100,ERR133
955:0.00000100)0.1467:0.00010800)0.3908:0.00000100,ERR067673:0.00010800)0.3912:0.000
00100,ERR403281:0.00000100)0.3916:0.00000100,ERR403302:0.00000100)0.3921:0.00000100
,ERR234641:0.00000100)0.3926:0.00000100,ERR403222:0.00010800)0.3930:0.00000100,ERR1
08514:0.00000100)0.3934:0.00000100,ERR227995:0.00000100)0.3938:0.00000100,ERR386962
:0.00032500)0.3942:0.00000100,ERR133961:0.00000100)0.3945:0.00000100,ERR234562:0.000
10800)0.3949:0.00000100,ERR133824:0.00054100)0.3953:0.00000100,ERR067723:0.00010800
)0.3957:0.00000100,(ERR386802:0.00000100,ERR351870:0.00000100)0.2800:0.00108300)0.39
66:0.00000100,ERR133979:0.00010800)0.3969:0.00000100,ERR108443:0.00000100)0.3973:0.0
0000100,ERR067637:0.00000100)0.3977:0.00000100,ERR108495:0.00010800)0.3981:0.000001
00,ERR403270:0.00000100)0.3985:0.00000100,ERR067732:0.00010800)0.3989:0.00000100,ER
R228065:0.00108300)0.3993:0.00000100,ERR403282:0.00000100)0.3998:0.00000100,ERR1339
85:0.00043300)0.4001:0.00000100,ERR117468:0.00000100)0.4005:0.00000100,ERR234563:0.0
0021600)0.4009:0.00000100,ERR144630:0.00032500)0.4013:0.00000100,ERR228036:0.000108
00)0.4017:0.00000100,ERR228014:0.00000100)0.4021:0.00000100,ERR386985:0.00129900)0.4
025:0.00000100,ERR234595:0.00000100)0.4029:0.00000100,ERR403391:0.00054100)0.4033:0.

00000100,ERR234577:0.00021600)0.4037:0.00000100,ERR133954:0.00000100)0.4041:0.00000
100,ERR133977:0.00032500)0.4044:0.00000100,ERR067683:0.00010800)0.4048:0.00000100,E
RR108510:0.00000100)0.4052:0.00000100,ERR403415:0.00021600)0.4056:0.00000100,ERR403
217:0.00000100)0.4060:0.00000100)0.4067:0.00000100,ERR067588:0.00000100)0.4071:0.000
00100,ERR133841:0.00000100)0.4075:0.00000100,ERR234674:0.00000100)0.4078:0.00000100
,ERR137260:0.00010800)0.4082:0.00000100,ERR137206:0.00000100)0.4086:0.00000100,ERR0
67622:0.00000100)0.4090:0.00000100,ERR133840:0.00000100)0.4094:0.00000100,ERR144554
:0.00000100)0.4098:0.00000100,(ERR403288:0.00000100,ERR229992:0.00000100)0.1867:0.00
021600)0.4107:0.00000100,ERR067750:0.00010800)0.4110:0.00000100,ERR228051:0.0000010
0)0.4114:0.00000100,ERR067741:0.00000100)0.4118:0.00000100,ERR228013:0.00000100)0.41
22:0.00000100,ERR144608:0.00000100)0.4126:0.00000100,ERR387008:0.00065000)0.4129:0.0
0000100,ERR234622:0.00000100)0.4133:0.00000100,ERR137215:0.00000100)0.4137:0.000001
00,ERR228006:0.00000100)0.4140:0.00000100,ERR133927:0.00000100)0.4145:0.00000100,ER
R133874:0.00032500)0.4148:0.00000100,ERR228066:0.00000100)0.4152:0.00000100,ERR1445
43:0.00000100)0.4156:0.00000100,ERR234667:0.00000100)0.4160:0.00000100,ERR133931:0.0
0000100)0.4165:0.00000100,ERR403259:0.00000100)0.4168:0.00000100,ERR137249:0.000001
00)0.4172:0.00000100,ERR403376:0.00151700)0.4176:0.00000100,ERR108420:0.00000100)0.4
180:0.00000100,ERR137263:0.00032500)0.4184:0.00000100,ERR027462:0.00032500)0.4188:0.
00000100,ERR067694:0.00000100)0.4192:0.00000100,ERR108507:0.00000100)0.4195:0.00000
100,ERR403313:0.00010800)0.4199:0.00000100,ERR144628:0.00000100)0.4203:0.00000100,E
RR234594:0.00000100)0.4207:0.00000100,ERR228058:0.00000100)0.4211:0.00000100,ERR067
761:0.00000100)0.4214:0.00000100,ERR227982:0.00021600)0.4217:0.00000100,ERR234688:0.
00000100)0.4221:0.00000100,(ERR386993:0.00032500,(ERR386829:0.00000100,ERR351897:0.
00000100)0.3100:0.00119100)0.1583:0.00000100)0.4232:0.00000100,(ERR133844:0.00000100
,ERR027446:0.00000100)0.1733:0.00010800)0.4239:0.00000100,ERR067578:0.00000100)0.424
3:0.00000100,ERR234651:0.00000100)0.4247:0.00000100,ERR137199:0.00000100)0.4251:0.00
000100,ERR234615:0.00000100)0.4255:0.00000100,ERR227985:0.00000100)0.4259:0.0000010
0,ERR351853:0.00000100)0.4262:0.00000100,ERR137285:0.00000100)0.4266:0.00000100,ERR
234606:0.00010800)0.4270:0.00000100,ERR144564:0.00010800)0.4273:0.00000100,ERR13727
8:0.00000100)0.4277:0.00000100)0.4285:0.00000100,ERR027452:0.00000100)0.4288:0.00000
100,ERR067742:0.00000100)0.4292:0.00000100,ERR137255:0.00000100)0.4296:0.00000100,E
RR133860:0.00000100)0.4299:0.00000100,ERR108458:0.00000100)0.4303:0.00000100,ERR484
746:0.00000100)0.4306:0.00000100,ERR228049:0.00010800)0.4310:0.00000100,ERR386785:0.
00000100)0.4313:0.00000100,ERR067736:0.00000100)0.4317:0.00000100,ERR137252:0.00000
100)0.4321:0.00000100,ERR133938:0.00000100)0.4325:0.00000100,ERR403345:0.00043300)0.
4328:0.00000100,ERR027456:0.00000100)0.4332:0.00000100,ERR234686:0.00000100)0.4335:
0.00000100,ERR403339:0.00021600)0.4339:0.00000100,ERR229979:0.00010800)0.4342:0.000
00100,ERR133819:0.00000100)0.4346:0.00000100,ERR067744:0.00000100)0.4350:0.00000100
,ERR133876:0.00010800)0.4354:0.00000100,ERR144633:0.00000100)0.4357:0.00000100,ERR1
37281:0.00000100)0.4361:0.00000100,ERR067599:0.00000100)0.4364:0.00000100,ERR403279
:0.00000100)0.4368:0.00000100,ERR144563:0.00010800)0.4372:0.00000100,ERR046883:0.000
00100)0.4376:0.00000100,(ERR038278:0.00000100,ERR038277:0.00000100)0.2933:0.0020580
0)0.4383:0.00000100,ERR386858:0.00000100)0.4387:0.00000100,ERR067705:0.00000100)0.43
90:0.00000100,ERR067623:0.00000100)0.4394:0.00000100,ERR108470:0.00000100)0.4398:0.0

0000100,ERR229976:0.00010800)0.4401:0.00000100,ERR137210:0.00000100)0.4404:0.000001
00,ERR386944:0.00032500)0.4407:0.00000100,ERR144610:0.00032500)0.4411:0.00000100,ER
R234576:0.00000100)0.4414:0.00000100,ERR067624:0.00032500)0.4418:0.00000100,ERR2280
56:0.00010800)0.4421:0.00000100,ERR403307:0.00000100)0.4425:0.00000100,ERR133847:0.0
0000100)0.4428:0.00000100,ERR137211:0.00000100)0.4431:0.00000100,ERR403271:0.000001
00)0.4435:0.00000100,ERR067700:0.00000100)0.4438:0.00000100,ERR137221:0.00000100)0.4
441:0.00000100,ERR229986:0.00010800)0.4446:0.00000100,ERR228040:0.00000100)0.4449:0.
00000100,ERR234574:0.00000100)0.4453:0.00000100,ERR234671:0.00000100)0.4457:0.00000
100,ERR403253:0.00000100)0.4460:0.00000100,ERR403277:0.00000100)0.4463:0.00000100,E
RR067643:0.00000100)0.4467:0.00000100,ERR137250:0.00000100)0.4471:0.00000100,ERR067
610:0.00054100)0.4475:0.00000100,ERR027448:0.00000100)0.4478:0.00000100,ERR067586:0.
00000100)0.4482:0.00000100,ERR133930:0.00000100)0.4485:0.00000100,ERR133831:0.00000
100)0.4489:0.00000100,ERR351930:0.00000100)0.4493:0.00000100,ERR230002:0.00064900)0.
4496:0.00000100,ERR158605:0.00054100)0.4500:0.00000100,ERR133907:0.00000100)0.4503:
0.00000100,ERR403419:0.00097400)0.4507:0.00000100,ERR067620:0.00000100)0.4511:0.000
00100,ERR046937:0.00097400)0.4514:0.00000100,ERR067656:0.00000100)0.4518:0.00000100
,ERR067709:0.00000100)0.4521:0.00000100,ERR229989:0.00010800)0.4525:0.00000100,ERR1
33868:0.00010800)0.4529:0.00000100,ERR133823:0.00086600)0.4532:0.00000100,ERR144549
:0.00000100)0.4536:0.00000100,ERR067668:0.00000100)0.4539:0.00000100,ERR403392:0.000
54100)0.4542:0.00000100,ERR133895:0.00000100)0.4545:0.00000100,(ERR133887:0.0003250
0,ERR386956:0.00184200)0.0000:0.00000100)0.4551:0.00000100,ERR403236:0.00000100)0.45
55:0.00000100,ERR234591:0.00000100)0.4559:0.00000100,ERR403234:0.00021600)0.4562:0.0
0000100,ERR067688:0.00000100)0.4566:0.00000100,ERR133849:0.00000100)0.4569:0.000001
00,ERR067758:0.00000100)0.4572:0.00000100,ERR137203:0.00000100)0.4575:0.00000100,ER
R133919:0.00000100)0.4579:0.00000100,ERR133948:0.00010800)0.4582:0.00000100,ERR1372
69:0.00000100)0.4585:0.00000100,ERR137257:0.00000100)0.4589:0.00000100,ERR108503:0.0
0000100)0.4592:0.00000100,ERR067718:0.00000100)0.4596:0.00000100,ERR403227:0.000001
00)0.4599:0.00000100,ERR019552:0.00021600)0.4604:0.00000100,ERR403226:0.00000100)0.4
607:0.00000100,ERR067666:0.00000100)0.4610:0.00000100,ERR133957:0.00010800)0.4614:0.
00000100,ERR229962:0.00000100)0.4618:0.00000100,(((((ERR228069:0.00000100,ERR229928:
0.00000100)0.1767:0.00000100,ERR133912:0.00000100)0.3283:0.00010800,ERR067716:0.000
43300)0.3100:0.00000100,ERR137200:0.00043300)0.3150:0.00010800,ERR228021:0.00162500
)0.2527:0.00000100)0.4638:0.00000100,ERR351926:0.00000100)0.4641:0.00000100,ERR40322
1:0.00000100)0.4644:0.00000100)0.4648:0.00000100,ERR403228:0.00000100)0.4651:0.00000
100,(((((ERR046984:0.00000100,ERR046859:0.00000100)0.3067:0.00086600,(ERR046868:0.000
00100,ERR046869:0.00000100)0.2733:0.00043300)0.1833:0.00000100,(((((ERR046917:0.00000
100,((((((((((((((((((((((((((((((((((((ERR039346:0.00000100,ERR038253:0.00000100)0.0667:0.00000
100,ERR046910:0.00000100)0.1200:0.00000100,ERR046904:0.00000100)0.1633:0.00000100,E
RR038257:0.00000100)0.2217:0.00000100,ERR038256:0.00000100)0.2573:0.00000100,ERR046
919:0.00000100)0.2983:0.00010800,ERR038286:0.00000100)0.2624:0.00000100,(((((((((((((((((((
(((ERR386856:0.00000100,(ERR038296:0.00000100,ERR038297:0.00000100)0.1533:0.00010800
)0.0950:0.00000100,ERR046793:0.00000100)0.0867:0.00000100,ERR046794:0.00000100)0.088
3:0.00000100,ERR046800:0.00010800)0.0867:0.00000100,ERR351924:0.00000100)0.0911:0.00
000100,ERR046795:0.00000100)0.1029:0.00000100,ERR386895:0.00000100)0.1154:0.0000010

0,ERR038294:0.00000100)0.1226:0.00000100,ERR038295:0.00000100)0.1360:0.00000100,ERR
046913:0.00000100)0.1506:0.00000100,ERR046792:0.00000100)0.1642:0.00000100,ERR04679
0:0.00000100)0.1838:0.00000100,ERR046787:0.00000100)0.2079:0.00000100,ERR403383:0.00
000100)0.2304:0.00000100,ERR046784:0.00000100)0.2481:0.00000100,ERR046785:0.0000010
0)0.2696:0.00000100,ERR046789:0.00000100)0.2907:0.00000100,ERR046788:0.00000100)0.31
16:0.00000100,ERR386878:0.00000100)0.3285:0.00000100,ERR046786:0.00000100)0.3468:0.0
0000100,ERR046911:0.00000100)0.3591:0.00000100,ERR387052:0.00000100)0.3746:0.000216
00)0.2938:0.00000100,ERR038285:0.00000100)0.2869:0.00000100,ERR387010:0.00000100)0.2
807:0.00000100,ERR046853:0.00000100)0.2757:0.00000100,ERR046852:0.00000100)0.2709:0.
00000100,ERR046822:0.00000100)0.2676:0.00000100,ERR046782:0.00000100)0.2654:0.00000
100,ERR038279:0.00000100)0.2625:0.00000100,ERR038275:0.00000100)0.2616:0.00000100,E
RR386969:0.00000100)0.2628:0.00000100,ERR046791:0.00000100)0.2655:0.00000100,ERR046
908:0.00000100)0.2698:0.00000100,(ERR351932:0.00000100,ERR386864:0.00000100)0.2000:0
.00010800)0.2798:0.00000100,ERR038274:0.00000100)0.2879:0.00000100,(ERR386865:0.0000
0100,ERR351933:0.00000100)0.2000:0.00010800)0.3017:0.00000100,ERR046851:0.00000100)
0.3090:0.00000100,ERR386907:0.00000100)0.3171:0.00000100,ERR046797:0.00000100)0.325
0:0.00000100,ERR046799:0.00000100)0.3335:0.00000100,ERR072087:0.00010800)0.3422:0.00
000100,ERR038276:0.00000100)0.3496:0.00000100,ERR403346:0.00000100)0.3558:0.0000010
0,ERR072096:0.00000100)0.3632:0.00000100,ERR046798:0.00000100)0.3706:0.00000100,ERR
046783:0.00000100)0.3781:0.00000100,ERR386922:0.00000100)0.3851:0.00000100,ERR04684
3:0.00000100)0.3918:0.00000100,ERR038281:0.00000100)0.3991:0.00000100,ERR386918:0.00
000100)0.4034:0.00000100)0.4098:0.00097400,(ERR040115:0.00000100,ERR040137:0.000325
00)0.3400:0.00108200)0.3993:0.00000100,((ERR403326:0.00021600,ERR387061:0.00021600)0.
2233:0.00000100,(ERR038755:0.00000100,ERR038751:0.00000100)0.3133:0.00043300)0.4100:
0.00054100)0.3796:0.00000100,(ERR403333:0.00054100,(ERR072028:0.00000100,ERR047000:
0.00000100)0.3000:0.00043300)0.3783:0.00032500)0.3701:0.00000100,((((ERR351917:0.00000
100,ERR351893:0.00000100)0.2167:0.00000100,ERR386825:0.00000100)0.2967:0.00000100,E
RR386849:0.00000100)0.4033:0.00119100,(ERR386845:0.00000100,ERR351913:0.00000100)0.
3133:0.00075800)0.3520:0.00010800)0.3729:0.00000100)0.3873:0.00000100,(((ERR072090:0.0
0000100,ERR072088:0.00000100)0.2133:0.00000100,ERR072089:0.00000100)0.3717:0.000649
00,ERR403420:0.00064900)0.2711:0.00000100)0.4039:0.00000100,ERR386976:0.00097400)0.4
081:0.00075800)0.4928:0.00000100,ERR229946:0.00000100)0.4931:0.00000100,(ERR137251:0
.00032500,((((((ERR387003:0.00129900,(SRR8375619:0.00000100,SRR8375620:0.00000100)0.2
800:0.00108200)0.2867:0.00000100,((((((ERR386978:0.00054100,ERR403385:0.00086500)0.12
67:0.00000100,(ERR403351:0.00075700,ERR386879:0.00054100)0.2467:0.00010800)0.2511:0.
00000100,SRR8375615:0.00064900)0.3292:0.00000100,(SRR8375623:0.00010800,SRR8375614:
0.00000100)0.2600:0.00054100)0.4172:0.00043300,(SRR8375617:0.00000100,SRR8375618:0.0
0000100)0.2467:0.00097400)0.3583:0.00000100,(((ERR046832:0.00000100,ERR046900:0.0000
0100)0.2767:0.00097400,SRR8375621:0.00064900)0.3250:0.00010800,ERR158603:0.00184100
)0.3611:0.00010800)0.3911:0.00010800)0.4158:0.00075700,(((((ERR046907:0.00000100,((ERR0
46915:0.00000100,ERR046905:0.00000100)0.1500:0.00000100,ERR386995:0.00000100)0.3083
:0.00000100)0.4144:0.00173100,((ERR047002:0.00000100,ERR047001:0.00000100)0.2767:0.00
108200,ERR403371:0.00043200)0.3750:0.00097400)0.3822:0.00000100,ERR387040:0.0015150
0)0.4152:0.00108200,ERR386949:0.00162400)0.3833:0.00000100,(((ERR386843:0.00000100,ER

R351911:0.00000100)0.2700:0.00151500,SRR8375622:0.00097400)0.3150:0.00010800,((ERR38
6797:0.00000100,ERR351865:0.00000100)0.2733:0.00119000,ERR387035:0.00184000)0.3217:
0.00000100)0.3833:0.00021600)0.2943:0.00000100)0.4023:0.00032400,(((ERR386877:0.00000
100,((ERR403379:0.00000100,ERR386873:0.00000100)0.1567:0.00000100,ERR403399:0.00000
100)0.2900:0.00000100)0.4344:0.00064900,ERR386920:0.00032400)0.4317:0.00119300,ERR38
6946:0.00303600)0.3993:0.00010600)0.3589:0.00000100,((((ERR017792:0.00097400,ERR38695
1:0.00054100)0.3100:0.00043300,ERR403329:0.00075800)0.3867:0.00086600,(SRR8375616:0.
00108200,((ERR351888:0.00000100,ERR386820:0.00000100)0.2700:0.00043200,ERR386965:0.
00000100)0.3700:0.00032400)0.4111:0.00064900)0.3883:0.00021700,((ERR040121:0.0030360
0,ERR386927:0.00217000)0.2067:0.00010500,ERR019568:0.00194900)0.1550:0.00000100)0.27
67:0.00000100)0.3839:0.00000100,((ERR046849:0.00000100,ERR046891:0.00000100)0.3467:0.
00271200,(ERR046855:0.00000100,ERR046856:0.00000100)0.2633:0.00249500)0.3556:0.0001
0500)0.4081:0.00097400)0.4001:0.00000100)0.5088:0.00000100,((ERR387056:0.00086600,ERR
403404:0.00075800)0.2767:0.00054200,ERR387045:0.00075800)0.3033:0.00010800)0.5097:0.
00000100,ERR144620:0.00010800)0.5099:0.00000100,(((((ERR234672:0.00010800,ERR403297:
0.00000100)0.1433:0.00000100,ERR067665:0.00000100)0.2600:0.00000100,ERR234589:0.000
00100)0.3344:0.00032500,((((ERR133963:0.00000100,(((ERR144622:0.00021600,ERR234612:0.
00000100)0.1833:0.00010800,ERR108492:0.00000100)0.1733:0.00000100,ERR158594:0.00000
100)0.2222:0.00000100)0.2942:0.00000100,ERR133856:0.00010800)0.3547:0.00000100,ERR13
7198:0.00000100)0.3917:0.00021600,(ERR137232:0.00021600,ERR403309:0.00010800)0.1900:
0.00010800)0.3963:0.00043300)0.2664:0.00000100,((((((((((ERR403220:0.00010800,ERR23458
7:0.00010800)0.0700:0.00000100,ERR228053:0.00000100)0.1050:0.00000100,ERR067703:0.00
000100)0.1233:0.00000100,((((ERR067675:0.00000100,ERR067734:0.00000100)0.1067:0.00000
100,ERR067751:0.00000100)0.2000:0.00000100,ERR229987:0.00000100)0.2778:0.00000100,E
RR108445:0.00010800)0.3117:0.00010800)0.2817:0.00000100,ERR133822:0.00000100)0.3004:
0.00000100,ERR234634:0.00010800)0.3170:0.00000100,ERR133832:0.00000100)0.3515:0.000
00100,ERR229959:0.00000100)0.3728:0.00000100,ERR234558:0.00000100)0.4036:0.00032500
,((((((ERR133801:0.00000100,ERR227998:0.00000100)0.1033:0.00000100,ERR234636:0.000001
00)0.1683:0.00000100,ERR108444:0.00000100)0.2200:0.00000100,ERR403239:0.00000100)0.2
950:0.00000100,ERR228008:0.00000100)0.3447:0.00000100,ERR228016:0.00000100)0.3950:0.
00054100)0.3793:0.00021600,(((ERR108460:0.00000100,ERR133803:0.00000100)0.1467:0.000
00100,ERR158571:0.00000100)0.3050:0.00000100,ERR234592:0.00000100)0.3889:0.00140800
)0.3165:0.00000100)0.2083:0.00000100)0.5207:0.00000100,(((ERR386974:0.00021600,(ERR04
6881:0.00000100,ERR046880:0.00000100)0.2833:0.00043300)0.3650:0.00086700,((ERR046824
:0.00000100,((ERR046945:0.00000100,ERR046850:0.00000100)0.1800:0.00000100,ERR046823:
0.00000100)0.3000:0.00000100)0.4078:0.00032500,ERR386981:0.00054100)0.3983:0.0005410
0)0.3986:0.00065000,(ERR386866:0.00000100,ERR351934:0.00000100)0.2967:0.00205900)0.3
644:0.00010800)0.5234:0.00000100,ERR067658:0.00000100)0.5237:0.00000100,ERR234665:0.
00032500)0.5240:0.00000100,(ERR403401:0.00032500,ERR019569:0.00086600)0.1567:0.0001
0800)0.5245:0.00000100,((ERR046962:0.00000100,(((((((ERR046991:0.00000100,ERR046946:0
.00000100)0.0467:0.00000100,ERR072036:0.00000100)0.1083:0.00000100,ERR072034:0.0000
0100)0.1700:0.00000100,ERR046988:0.00000100)0.2200:0.00000100,ERR072020:0.00010800)
0.2667:0.00000100,ERR046980:0.00000100)0.3022:0.00000100,ERR046739:0.00000100)0.351
9:0.00000100,ERR046975:0.00010800)0.3767:0.00000100)0.4070:0.00119100,(((ERR046906:0.

00000100,((ERR046916:0.00000100,ERR039325:0.00000100)0.1500:0.00000100,ERR039326:0.
00000100)0.2417:0.00000100)0.3300:0.00000100,ERR046912:0.00000100)0.4192:0.00065000,
ERR386882:0.00043300)0.4120:0.00043300)0.3720:0.00021700)0.5290:0.00000100,ERR13385
3:0.00000100)0.5292:0.00000100,((((ERR351878:0.00000100,ERR386810:0.00000100)0.2733:0.
00021600,((ERR038288:0.00000100,ERR038287:0.00000100)0.2633:0.00010800,ERR038280:0.
00000100)0.3833:0.00043300)0.3208:0.00000100,(ERR386807:0.00000100,ERR351875:0.0000
0100)0.3000:0.00064900)0.3844:0.00010800,(ERR039331:0.00000100,ERR039332:0.00000100)
0.3000:0.00054100)0.3913:0.00021700)0.5316:0.00085300,((((ERR6375116:0.00000100,ERR63
75115:0.00000100)0.3267:0.00043300,(((((ERR6375117:0.00000100,ERR6375125:0.00000100)
0.1200:0.00000100,ERR6375120:0.00000100)0.2133:0.00000100,ERR6375118:0.00000100)0.3
133:0.00000100,ERR6375126:0.00000100)0.3983:0.00000100,ERR6375119:0.00000100)0.4540
:0.00043300)0.4186:0.00000100,ERR017782:0.00043300)0.4463:0.00021500,(ERR386816:0.00
000100,ERR351884:0.00000100)0.3433:0.00108400)0.4493:0.00392900)0.5327:0.00854400,((((
((((((((((((((((((((((((((((((((((((ERR1462622:0.00000100,ERR1462605:0.00000100)0.0167:0.000001
00,ERR1462625:0.00000100)0.0283:0.00000100,ERR1462585:0.00000100)0.0300:0.00000100,
ERR1462604:0.00010800)0.0267:0.00000100,ERR1462628:0.00000100)0.0473:0.00000100,ERR
1462633:0.00000100)0.0550:0.00000100,ERR1462590:0.00000100)0.0629:0.00000100,ERR146
2607:0.00000100)0.0696:0.00000100,ERR1462624:0.00000100)0.0789:0.00000100,ERR146258
7:0.00000100)0.0900:0.00000100,ERR1462593:0.00000100)0.0985:0.00000100,ERR1462589:0.
00000100)0.1058:0.00000100,ERR1462606:0.00000100)0.1123:0.00000100,ERR1462592:0.000
00100)0.1202:0.00000100,ERR1462626:0.00000100)0.1258:0.00000100,ERR1462597:0.000001
00)0.1375:0.00000100,ERR1462594:0.00010800)0.1429:0.00000100,ERR1462596:0.00000100)
0.1578:0.00000100,ERR1462630:0.00010800)0.1675:0.00000100,ERR1462632:0.00000100)0.1
890:0.00000100,ERR1462598:0.00000100)0.2114:0.00000100,ERR1462586:0.00000100)0.2382
:0.00000100,ERR1462629:0.00000100)0.2612:0.00000100,ERR1462623:0.00000100)0.2863:0.0
0000100,ERR1462591:0.00000100)0.3143:0.00000100,ERR1462634:0.00000100)0.3388:0.0000
0100,ERR1462631:0.00000100)0.3606:0.00000100,ERR1462599:0.00000100)0.3829:0.0000010
0,ERR1462584:0.00000100)0.4048:0.00000100,ERR1462595:0.00000100)0.4234:0.00000100,E
RR1462588:0.00000100)0.4420:0.00000100,ERR1462600:0.00000100)0.4558:0.00000100,ERR1
462603:0.00000100)0.4724:0.00000100,ERR1462627:0.00000100)0.4896:0.00000100,ERR1462
601:0.00000100)0.5048:0.00000100,ERR1462602:0.00000100)0.5185:0.00000100,ERR1462608
:0.00000100)0.5331:0.00173000,((((SRR13888795:0.00010800,((((((((((SRR17111235:0.0001080
0,((((((((((((((((SRR17111226:0.00000100,SRR17111248:0.00000100)0.0233:0.00000100,SRR171
11231:0.00010800)0.0300:0.00000100,SRR17111236:0.00000100)0.0478:0.00000100,SRR1711
1224:0.00000100)0.0592:0.00000100,SRR17111250:0.00010800)0.0720:0.00000100,SRR17111
241:0.00000100)0.0894:0.00000100,SRR17111237:0.00000100)0.1057:0.00000100,SRR171112
28:0.00000100)0.1200:0.00000100,SRR17111232:0.00000100)0.1359:0.00000100,SRR1711124
9:0.00000100)0.1507:0.00000100,SRR17111220:0.00000100)0.1667:0.00000100,SRR17111247:
0.00000100)0.1825:0.00000100,SRR17111243:0.00000100)0.2026:0.00000100,SRR17111244:0.
00000100)0.2262:0.00000100,SRR17111221:0.00000100)0.2531:0.00000100,SRR17111246:0.0
0000100)0.2810:0.00000100)0.3084:0.00000100,(SRR17111252:0.00000100,SRR17111230:0.0
0000100)0.2867:0.00010800)0.3642:0.00000100,(SRR17111223:0.00000100,SRR17111233:0.0
0000100)0.3233:0.00010800)0.4198:0.00000100,SRR17111245:0.00000100)0.4502:0.00000100
,SRR17111227:0.00000100)0.4746:0.00000100,SRR17111239:0.00000100)0.4942:0.00000100,S

RR17111234:0.00000100)0.5160:0.00000100,SRR17111222:0.00000100)0.5331:0.00032400,SR
R13888789:0.00064800)0.5243:0.00000100,SRR13888797:0.00021600)0.5214:0.00000100)0.5
323:0.00021600,(((SRR13888750:0.00021600,SRR13888778:0.00000100)0.4100:0.00075700,SR
R17111242:0.00097300)0.4550:0.00010800,SRR13888776:0.00118900)0.3811:0.00000100)0.5
265:0.00021600,((((((ERR1462612:0.00000100,((((((((SRR13888783:0.00000100,ERR1462615:0.
00000100)0.0533:0.00000100,ERR1462619:0.00000100)0.0583:0.00000100,ERR1462621:0.000
00100)0.0800:0.00000100,ERR1462616:0.00000100)0.0992:0.00000100,ERR1462613:0.000001
00)0.1287:0.00000100,ERR1462611:0.00000100)0.1611:0.00000100,ERR1462578:0.00000100)
0.1819:0.00000100,ERR1462620:0.00000100)0.2133:0.00000100,ERR1462618:0.00000100)0.2
396:0.00000100)0.2723:0.00000100,(((ERR1462582:0.00000100,ERR1462583:0.00173100)0.02
00:0.00000100,(((((ERR1462574:0.00000100,ERR1462577:0.00000100)0.0733:0.00000100,ERR
1462581:0.00000100)0.1367:0.00000100,ERR1462580:0.00000100)0.1800:0.00000100,ERR146
2576:0.00000100)0.2250:0.00000100,ERR1462575:0.00000100)0.2793:0.00000100)0.3462:0.0
0000100,ERR1462579:0.00000100)0.3917:0.00010800)0.4384:0.00000100,ERR1462617:0.0000
0100)0.4658:0.00000100,ERR1462610:0.00000100)0.4873:0.00000100,ERR1462614:0.0000010
0)0.5126:0.00000100,ERR1462609:0.00000100)0.5372:0.00108100)0.3545:0.00000100,((SRR13
888781:0.00129900,(SRR13888784:0.00021600,SRR13888774:0.00010800)0.4400:0.00108200)
0.4067:0.00010700,SRR17156532:0.00140600)0.3078:0.00000100)0.3514:0.00000100)0.5339:
0.00173300,((((((((SRR13888749:0.00000100,SRR13888798:0.00010800)0.0767:0.00000100,SR
R13888751:0.00000100)0.1483:0.00000100,SRR13888754:0.00010800)0.1933:0.00000100,(SR
R13888753:0.00000100,SRR13888755:0.00000100)0.2733:0.00010800)0.3093:0.00000100,SRR
13888790:0.00000100)0.3911:0.00000100,SRR13888765:0.00000100)0.4786:0.00000100,SRR1
3888752:0.00000100)0.5500:0.00075700,((((SRR13888782:0.00010800,(((((((((((SRR13888793:
0.00000100,SRR11418319:0.00000100)0.3433:0.00021600,SRR20551054:0.00000100)0.1950:0.
00000100,(((((SRR20551046:0.00000100,SRR20551025:0.00000100)0.0833:0.00000100,SRR205
51027:0.00000100)0.1533:0.00000100,SRR13888758:0.00000100)0.2300:0.00000100,SRR2055
1028:0.00000100)0.3017:0.00000100,SRR20551026:0.00000100)0.3820:0.00010800)0.2946:0.
00000100,(SRR13888785:0.00000100,SRR13888786:0.00000100)0.2833:0.00010800)0.2700:0.
00000100,SRR13888772:0.00010800)0.2773:0.00000100,SRR20551047:0.00010800)0.2853:0.0
0000100,SRR20551053:0.00000100)0.3051:0.00000100,SRR13888780:0.00021600)0.3310:0.00
000100,(SRR20551024:0.00000100,SRR20551023:0.00000100)0.3733:0.00021600)0.3871:0.00
000100,SRR13888799:0.00000100)0.4171:0.00000100,SRR13888760:0.00000100)0.4446:0.000
00100,SRR13888761:0.00010800)0.4667:0.00000100)0.4925:0.00000100,SRR13888769:0.0000
0100)0.5110:0.00021600,(((SRR13888756:0.00000100,SRR13888767:0.00000100)0.1933:0.000
00100,SRR13888757:0.00000100)0.3717:0.00000100,SRR13888759:0.00010800)0.5311:0.0003
2400)0.5196:0.00010800,SRR13888792:0.00043200)0.5226:0.00064900)0.4304:0.00000100,((S
RR13888796:0.00010800,((((((SRR13888768:0.00010800,SRR13888777:0.00010800)0.2767:0.0
0010800,(((SRR13888779:0.00000100,SRR13888764:0.00000100)0.1467:0.00000100,SRR13888
766:0.00000100)0.2750:0.00000100,SRR13888775:0.00000100)0.3889:0.00010800)0.3187:0.0
0000100,SRR13888788:0.00010800)0.3300:0.00000100,SRR13888794:0.00000100)0.3710:0.00
000100,SRR13888787:0.00000100)0.4242:0.00000100,SRR13888791:0.00010800)0.4700:0.000
10800)0.4670:0.00000100,((((SRR13888762:0.00010800,SRR13888763:0.00032400)0.0700:0.00
000100,SRR13888770:0.00000100)0.1833:0.00000100,SRR13888773:0.00000100)0.3156:0.000
00100,SRR13888771:0.00000100)0.4325:0.00010800)0.5449:0.00064900)0.5324:0.00184200)0

.5325:0.00097400,(SRR1792164:0.00000100,SRR1792165:0.00000100)0.4433:0.00369000)0.53
24:0.00074600)0.5347:0.00273000,((SRR8065077:0.00000100,SRR8065075:0.00000100)0.4000
:0.00032500,(SRR8065072:0.00010800,(SRR8065083:0.00000100,SRR8065076:0.00000100)0.3
767:0.00010800)0.5100:0.00021600)0.5467:0.00456800)0.5347:0.00434400,((((((((((SRR114183
16:0.00205400,((SRR11418320:0.00000100,SRR11418324:0.00000100)0.4233:0.00054000,((((((
((((((((SRR1791860:0.00000100,SRR1791875:0.00000100)0.0467:0.00000100,SRR14296114:0.0
0000100)0.0783:0.00000100,SRR5657358:0.00000100)0.1078:0.00000100,SRR5657512:0.0000
0100)0.1417:0.00000100,SRR1791869:0.00000100)0.1673:0.00000100,SRR5657359:0.0001080
0)0.1800:0.00000100,SRR5657513:0.00000100)0.2100:0.00000100,SRR14296108:0.00000100)
0.2550:0.00000100,SRR1791867:0.00000100)0.3100:0.00000100,SRR1791876:0.00000100)0.35
90:0.00000100,SRR1791880:0.00000100)0.4115:0.00000100,SRR14296090:0.00000100)0.4622:
0.00000100,SRR14296113:0.00021600)0.4887:0.00000100,SRR1791868:0.00000100)0.5231:0.0
0000100,SRR14296109:0.00000100)0.5642:0.00043200)0.5608:0.00064800)0.5369:0.00000100
,(((SRR15296973:0.00000100,SRR15296943:0.00010800)0.4733:0.00086400,(SRR15296947:0.0
0000100,SRR15296962:0.00000100)0.4433:0.00129700)0.5100:0.00021600,(((SRR15296940:0.
00000100,SRR15296936:0.00000100)0.2600:0.00000100,SRR15296959:0.00000100)0.5650:0.0
0075600,(SRR15296924:0.00000100,SRR15296923:0.00000100)0.4300:0.00140500)0.3383:0.0
0000100)0.2704:0.00000100)0.4143:0.00000100,(((((((((((((((((((((((SRR11418326:0.00010800,S
RR20551043:0.00000100)0.0300:0.00000100,(SRR15296968:0.00000100,SRR15296970:0.0000
0100)0.3900:0.00021600)0.1589:0.00000100,(SRR11418318:0.00010800,SRR15296971:0.0001
0800)0.2933:0.00010800)0.1527:0.00000100,SRR15296983:0.00000100)0.1344:0.00000100,SR
R15296914:0.00000100)0.1219:0.00000100,SRR20551037:0.00000100)0.1179:0.00000100,(((((
(SRR15296966:0.00000100,SRR15296910:0.00010800)0.0567:0.00000100,SRR15296977:0.0000
0100)0.1100:0.00000100,SRR15296933:0.00000100)0.1711:0.00000100,SRR20551041:0.00000
100)0.2325:0.00000100,SRR20551034:0.00000100)0.2900:0.00000100,SRR20551032:0.000001
00)0.3478:0.00010800)0.2189:0.00000100,SRR20551035:0.00000100)0.2260:0.00000100,SRR1
5296922:0.00000100)0.2355:0.00000100,SRR20551036:0.00000100)0.2520:0.00000100,SRR15
296955:0.00000100)0.2735:0.00000100,SRR20551042:0.00000100)0.2997:0.00000100,SRR152
96982:0.00000100)0.3314:0.00000100,SRR20551045:0.00010800)0.3615:0.00000100,SRR1529
6915:0.00000100)0.3897:0.00000100,SRR15296911:0.00000100)0.4156:0.00000100,SRR20551
040:0.00000100)0.4368:0.00000100,SRR15296980:0.00021600)0.4594:0.00000100,SRR152969
13:0.00000100)0.4837:0.00000100,SRR15296912:0.00000100)0.5069:0.00000100,SRR2055103
9:0.00000100)0.5248:0.00000100,SRR20551038:0.00000100)0.5418:0.00000100,SRR15296944:
0.00000100)0.5565:0.00054000)0.5343:0.00010800,((((SRR1792449:0.00010800,SRR1791891:0
.00000100)0.4100:0.00021600,(SRR1791830:0.00000100,SRR1791841:0.00000100)0.4267:0.00
032400)0.4378:0.00000100,SRR1791793:0.00010800)0.5333:0.00021600,SRR11418330:0.0006
4800)0.5500:0.00043200)0.5451:0.00021600,(((SRR1792284:0.00000100,((((((((((((((((((((((((((((
((((((((((((((SRR1792223:0.00000100,((((((((((((((((((((((((((((SRR1792173:0.00000100,SRR17924
44:0.00000100)0.0167:0.00000100,SRR1792210:0.00000100)0.0250:0.00000100,SRR1792213:0
.00000100)0.0333:0.00000100,SRR1792208:0.00000100)0.0467:0.00000100,SRR1792216:0.000
00100)0.0567:0.00000100,SRR1792184:0.00000100)0.0683:0.00000100,SRR1792221:0.000001
00)0.0748:0.00000100,SRR1792218:0.00000100)0.0788:0.00000100,SRR1792188:0.00000100)
0.0844:0.00000100,SRR1792209:0.00000100)0.0897:0.00000100,SRR1792225:0.00000100)0.09
91:0.00000100,SRR1792219:0.00000100)0.1047:0.00000100,SRR1792222:0.00000100)0.1121:0

.00000100,SRR1792228:0.00000100)0.1193:0.00000100,SRR1792189:0.00000100)0.1340:0.000
00100,SRR1792220:0.00000100)0.1463:0.00000100,SRR1792226:0.00010800)0.1610:0.000001
00,SRR1792215:0.00000100)0.1793:0.00000100,SRR1792229:0.00000100)0.1974:0.00000100,S
RR1792190:0.00000100)0.2185:0.00000100,SRR1792246:0.00000100)0.2394:0.00000100,SRR1
792224:0.00000100)0.2580:0.00000100,SRR1792227:0.00000100)0.2765:0.00000100,SRR1792
249:0.00000100)0.2933:0.00000100,SRR1792247:0.00000100)0.3111:0.00000100,SRR1792214:
0.00000100)0.3281:0.00000100,SRR1792211:0.00000100)0.3432:0.00000100,SRR1792217:0.00
000100)0.3576:0.00000100,SRR1792212:0.00000100)0.3695:0.00000100,SRR1792230:0.00000
100)0.3841:0.00000100)0.3985:0.00010800,SRR1791776:0.00000100)0.3892:0.00000100,SRR1
792200:0.00000100)0.3803:0.00000100,SRR1792484:0.00000100)0.3714:0.00000100,SRR1792
472:0.00000100)0.3631:0.00000100,SRR1792234:0.00010800)0.3577:0.00000100,SRR1792500:
0.00000100)0.3516:0.00000100,SRR1792505:0.00000100)0.3476:0.00000100,SRR1791708:0.00
010800)0.3436:0.00000100,SRR4019491:0.00000100)0.3408:0.00000100,SRR1792239:0.00000
100)0.3394:0.00000100,SRR1792233:0.00000100)0.3379:0.00000100,SRR1792235:0.00000100
)0.3372:0.00000100,SRR1792498:0.00010800)0.3361:0.00000100,SRR1792002:0.00000100)0.3
358:0.00000100,SRR1792293:0.00000100)0.3362:0.00000100,SRR1792251:0.00000100)0.3372:
0.00000100,SRR1792476:0.00000100)0.3389:0.00000100,SRR1792499:0.00000100)0.3420:0.00
000100,SRR1792238:0.00000100)0.3449:0.00000100,SRR1792205:0.00010800)0.3490:0.00000
100,SRR1792232:0.00000100)0.3540:0.00000100,SRR1792237:0.00000100)0.3595:0.00000100,
SRR1791692:0.00000100)0.3681:0.00000100,SRR1792207:0.00000100)0.3776:0.00000100,SRR
1791832:0.00000100)0.3863:0.00000100,SRR1792486:0.00000100)0.3931:0.00000100,SRR179
2236:0.00000100)0.4044:0.00000100,SRR1792295:0.00000100)0.4137:0.00000100,SRR179216
3:0.00010800)0.4241:0.00000100,SRR1792201:0.00000100)0.4344:0.00000100,SRR1792475:0.
00010800)0.4424:0.00000100,SRR1792473:0.00000100)0.4523:0.00000100,SRR1792258:0.000
00100)0.4628:0.00000100,SRR4019488:0.00000100)0.4724:0.00000100,SRR1792478:0.000001
00)0.4808:0.00000100,SRR1792480:0.00000100)0.4893:0.00000100,SRR1792285:0.00000100)
0.4980:0.00000100,SRR4019485:0.00000100)0.5078:0.00000100,SRR1791768:0.00000100)0.51
57:0.00000100,SRR1792292:0.00000100)0.5227:0.00000100,SRR1792496:0.00000100)0.5297:0
.00000100,SRR1792206:0.00000100)0.5363:0.00000100,SRR1792495:0.00000100)0.5441:0.000
00100)0.5519:0.00043200,SRR11418321:0.00054000)0.5528:0.00064800,SRR11418315:0.0006
4800)0.5467:0.00000100)0.3581:0.00000100,(((((((((ERR4450963:0.00043200,(((((((ERR445097
4:0.00000100,ERR4450973:0.00000100)0.0800:0.00000100,ERR4450975:0.00000100)0.1383:0.
00000100,ERR4450971:0.00000100)0.2078:0.00000100,ERR4450972:0.00000100)0.3117:0.000
00100,ERR4450970:0.00000100)0.3827:0.00000100,ERR4450976:0.00000100)0.4639:0.000108
00,((((((SRR13015802:0.00000100,SRR13015807:0.00000100)0.0967:0.00000100,SRR13015801:
0.00000100)0.2033:0.00000100,SRR13015804:0.00000100)0.3256:0.00000100,SRR13015805:0.
00021600)0.4050:0.00000100,SRR13015803:0.00000100)0.4893:0.00021600,((((((((((SRR13015
798:0.00000100,SRR13015808:0.00000100)0.0633:0.00000100,SRR13015794:0.00000100)0.09
83:0.00000100,SRR13015797:0.00000100)0.1444:0.00000100,SRR13015800:0.00000100)0.186
7:0.00000100,SRR13015795:0.00000100)0.2420:0.00000100,SRR13015809:0.00000100)0.3072:
0.00000100,SRR13015810:0.00000100)0.3600:0.00000100,SRR9850824:0.00000100)0.4167:0.0
0000100,SRR13015796:0.00000100)0.4719:0.00000100,SRR13015799:0.00000100)0.5210:0.00
021600,(((ERR4451184:0.00000100,ERR4451187:0.00000100)0.1500:0.00000100,ERR4451186:
0.00000100)0.3267:0.00000100,ERR4451185:0.00000100)0.4422:0.00010800)0.4917:0.000108

00)0.4162:0.00000100)0.4595:0.00010800)0.4482:0.00000100,(((SRR13046687:0.00010800,SR
R13046671:0.00010800)0.4500:0.00086400,ERR4450935:0.00075600)0.2433:0.00000100,((((SR
R11462863:0.00021600,SRR11462885:0.00000100)0.2233:0.00000100,SRR11462928:0.000108
00)0.3750:0.00000100,SRR11462882:0.00010800)0.5289:0.00054000,((ERR4451192:0.0000010
0,ERR4451193:0.00010800)0.3000:0.00000100,ERR4451194:0.00000100)0.5333:0.00032400)0.
3233:0.00000100)0.2615:0.00000100)0.4325:0.00000100,(((((((ERR4450933:0.00000100,ERR4
450927:0.00000100)0.0967:0.00000100,ERR4450926:0.00000100)0.1350:0.00000100,ERR4450
928:0.00000100)0.1933:0.00000100,ERR4450934:0.00000100)0.2550:0.00000100,ERR4450930
:0.00000100)0.3307:0.00000100,ERR4450931:0.00000100)0.4000:0.00000100,ERR4450929:0.0
0000100)0.4648:0.00000100,ERR4450932:0.00000100)0.5288:0.00021600)0.5129:0.00010800,
(((((SRR13046674:0.00000100,SRR13046673:0.00000100)0.0867:0.00000100,SRR13046686:0.0
0000100)0.2267:0.00000100,SRR13046680:0.00000100)0.3422:0.00000100,SRR13046669:0.00
000100)0.4333:0.00000100,SRR13046677:0.00000100)0.5393:0.00032400)0.5260:0.00000100,
((SRR14001969:0.00021600,SRR14001975:0.00000100)0.4733:0.00086400,SRR14001976:0.000
54000)0.5117:0.00032400)0.5458:0.00032400,((((((ERR4451180:0.00000100,ERR4451183:0.00
000100)0.1967:0.00000100,ERR4451181:0.00000100)0.3700:0.00000100,ERR4451182:0.00000
100)0.5444:0.00021600,(((((ERR4450922:0.00000100,ERR4450919:0.00000100)0.1200:0.00000
100,ERR4450923:0.00000100)0.1950:0.00000100,ERR4450925:0.00000100)0.3233:0.00000100
,ERR4450920:0.00000100)0.4458:0.00000100,ERR4450924:0.00000100)0.5393:0.00043200)0.3
963:0.00000100,(ERR4451195:0.00054000,SRR13046683:0.00000100)0.4433:0.00054000)0.42
70:0.00000100,(ERR4451189:0.00000100,ERR4451188:0.00000100)0.4767:0.00075600)0.4721:
0.00010800)0.5452:0.00021600,(((((((ERR4450958:0.00000100,(ERR4450921:0.00021600,ERR4
450956:0.00000100)0.2233:0.00000100)0.4967:0.00021600,(((ERR4450950:0.00000100,(((ERR
4450937:0.00000100,ERR4450938:0.00000100)0.1900:0.00000100,ERR4450957:0.00000100)0.
3600:0.00000100,ERR4450936:0.00000100)0.5411:0.00043200)0.4283:0.00000100,ERR445095
1:0.00000100)0.3600:0.00000100,ERR4450952:0.00010800)0.3378:0.00000100)0.3781:0.0000
0100,(((ERR4450945:0.00000100,ERR4450947:0.00000100)0.3133:0.00010800,ERR4450939:0.0
0000100)0.3300:0.00000100,ERR4450946:0.00000100)0.4256:0.00010800)0.5126:0.00021600,
(ERR4450959:0.00000100,ERR4450962:0.00000100)0.4800:0.00054000)0.4751:0.00000100,((((
(((ERR4450967:0.00000100,ERR4450964:0.00000100)0.1067:0.00000100,ERR4450961:0.00000
100)0.1567:0.00000100,ERR4450960:0.00000100)0.2200:0.00000100,ERR4450965:0.00000100
)0.3000:0.00000100,ERR4450968:0.00000100)0.3993:0.00000100,ERR4450966:0.00000100)0.4
833:0.00000100,ERR4450969:0.00000100)0.5448:0.00032400)0.4922:0.00000100,SRR1301580
6:0.00032400)0.5089:0.00000100,(ERR4451190:0.00000100,ERR4451191:0.00000100)0.4567:0
.00054000)0.5394:0.00032400)0.5297:0.00010800,((((((SRR14001962:0.00000100,SRR1400196
1:0.00000100)0.3000:0.00010800,((((((SRR20551055:0.00000100,SRR14001968:0.00010800)0.
0667:0.00000100,(SRR20551056:0.00010800,SRR20551033:0.00000100)0.2667:0.00010800)0.
1778:0.00000100,SRR14001977:0.00000100)0.2108:0.00000100,SRR14001963:0.00000100)0.2
620:0.00000100,SRR14001972:0.00010800)0.3233:0.00000100,SRR20551044:0.00021600)0.38
95:0.00000100)0.5119:0.00000100,SRR14001965:0.00043200)0.5493:0.00043200,SRR1400197
0:0.00021600)0.5179:0.00000100,SRR14001967:0.00032400)0.5067:0.00000100,SRR14001971:
0.00032400)0.5197:0.00010800)0.5447:0.00010800,(SRR14001973:0.00000100,SRR14001964:
0.00021600)0.4467:0.00064800)0.5439:0.00000100,((SRR14001966:0.00064800,SRR1792073:0
.00075600)0.4300:0.00032400,SRR1792068:0.00064800)0.5467:0.00043200)0.5521:0.0005400

0)0.4553:0.00000100,((((((((SRR1791736:0.00000100,(((SRR17077299:0.00000100,(SRR170772
92:0.00000100,SRR17077295:0.00000100)0.4367:0.00021600)0.2950:0.00000100,SRR1707729
6:0.00000100)0.2944:0.00000100,SRR17077293:0.00000100)0.3425:0.00000100)0.4160:0.000
00100,SRR17077294:0.00000100)0.5017:0.00021600,((SRR1791802:0.00000100,SRR1791946:0
.00000100)0.3800:0.00010800,SRR1792245:0.00010800)0.4317:0.00010800)0.3911:0.0000010
0,(SRR17077291:0.00021600,SRR17077290:0.00000100)0.2700:0.00010800)0.4130:0.0000010
0,SRR1791950:0.00010800)0.4489:0.00000100,(SRR1791960:0.00010800,SRR1792445:0.00000
100)0.3267:0.00010800)0.5176:0.00000100,SRR1792155:0.00010800)0.5549:0.00097200,((SRR
11418333:0.00000100,SRR11418327:0.00000100)0.2367:0.00000100,SRR11418322:0.0000010
0)0.5300:0.00086400)0.5237:0.00010800)0.4935:0.00000100,(((((((SRR1791909:0.00010800,(((
(((((((((((SRR1792261:0.00000100,SRR1792122:0.00000100)0.3100:0.00010800,((((SRR179220
4:0.00010800,SRR1792193:0.00000100)0.2000:0.00000100,SRR1792203:0.00000100)0.3833:0.
00010800,SRR1791969:0.00010800)0.3256:0.00000100,SRR1792502:0.00010800)0.3250:0.000
00100)0.4122:0.00010800,SRR1792109:0.00000100)0.3576:0.00000100,(SRR1792409:0.00000
100,SRR1792410:0.00000100)0.2933:0.00010800)0.2956:0.00000100,((SRR1792041:0.0002160
0,SRR1792491:0.00000100)0.2067:0.00000100,SRR1792477:0.00021600)0.3500:0.00010800)0.
2681:0.00000100,SRR1791959:0.00000100)0.2603:0.00000100,SRR1792101:0.00000100)0.263
3:0.00000100,SRR1792110:0.00000100)0.2689:0.00000100,SRR1792108:0.00000100)0.2819:0.
00000100,SRR1791797:0.00032400)0.3031:0.00000100,SRR1791722:0.00021600)0.3252:0.000
00100,SRR1792005:0.00000100)0.3537:0.00000100,SRR1792147:0.00000100)0.3847:0.000001
00,(SRR1791804:0.00010800,SRR1792171:0.00021600)0.3033:0.00010800)0.4415:0.00000100,
SRR1792118:0.00000100)0.4659:0.00000100,SRR1791824:0.00010800)0.4881:0.00000100)0.5
089:0.00000100,SRR1792027:0.00000100)0.5322:0.00000100,SRR1792111:0.00000100)0.5516:
0.00021600,(SRR11418325:0.00032400,SRR11418338:0.00021600)0.4700:0.00032400)0.5518:
0.00021600,(SRR11418339:0.00064800,(ERR4352201:0.00032400,ERR4352202:0.00000100)0.3
833:0.00010800)0.4650:0.00021600)0.5346:0.00000100,(SRR18053779:0.00021600,SRR15296
981:0.00043200)0.4133:0.00021600)0.5549:0.00021600,SRR11418334:0.00032400)0.5560:0.0
0043200)0.5531:0.00054000,((SRR17156530:0.00086400,SRR17156539:0.00075600)0.4167:0.0
0086600,((((((SRR17156545:0.00000100,SRR17156531:0.00000100)0.2067:0.00000100,SRR171
56547:0.00000100)0.3667:0.00000100,SRR17156533:0.00000100)0.5156:0.00010800,(((SRR17
156540:0.00000100,SRR17156543:0.00010800)0.1767:0.00000100,SRR17156549:0.00000100)0
.3683:0.00000100,SRR17156535:0.00000100)0.5644:0.00054000)0.5471:0.00021600,SRR17156
548:0.00086400)0.5354:0.00010800,(((((SRR17111240:0.00000100,SRR17156542:0.00021600)0
.0600:0.00000100,SRR17111238:0.00010800)0.1150:0.00000100,SRR17111229:0.00000100)0.2
778:0.00000100,SRR17111225:0.00000100)0.4417:0.00000100,SRR17111251:0.00000100)0.54
80:0.00129600)0.5383:0.00010700)0.5571:0.00140600)0.5312:0.00000100,((((SRR13046668:0.
00010800,(((SRR13046672:0.00000100,(SRR13046676:0.00000100,SRR13046681:0.00000100)0
.2867:0.00010800)0.2800:0.00000100,SRR13046685:0.00021600)0.3222:0.00000100,SRR13046
670:0.00000100)0.4300:0.00000100)0.5053:0.00021600,(SRR13046678:0.00010800,(SRR13046
684:0.00000100,SRR13046689:0.00000100)0.3967:0.00010800)0.4283:0.00010800)0.5008:0.0
0000100,SRR13046679:0.00032400)0.5493:0.00097200,((((SRR8064855:0.00000100,(SRR80648
58:0.00010800,SRR8064859:0.00000100)0.3233:0.00010800)0.3150:0.00000100,(SRR8064860:
0.00000100,SRR8064856:0.00000100)0.4300:0.00032400)0.4292:0.00000100,SRR8064857:0.00
021600)0.5060:0.00000100,SRR8064853:0.00010800)0.5628:0.00183800)0.3617:0.00000100)0

.5340:0.00010800)0.4878:0.00000100)0.5042:0.00000100,ERR6140818:0.00000100)0.5044:0.0
0000100,(((SRR13986564:0.00010800,(((((SRR5486090:0.00000100,SRR5486091:0.00000100)0.
1067:0.00000100,SRR5486093:0.00000100)0.2000:0.00000100,SRR15296969:0.00000100)0.31
44:0.00000100,SRR5486092:0.00000100)0.4350:0.00000100,SRR5486089:0.00000100)0.5307:0
.00010800)0.5450:0.00021600,SRR13986559:0.00032400)0.5400:0.00021600,(((((SRR15296974
:0.00000100,(((((SRR15296965:0.00000100,SRR20551030:0.00000100)0.0967:0.00000100,SRR5
486088:0.00000100)0.1617:0.00000100,SRR5486087:0.00010800)0.2111:0.00000100,SRR2055
1029:0.00000100)0.3100:0.00000100,SRR11418337:0.00000100)0.4040:0.00000100)0.4794:0.
00000100,SRR20551031:0.00000100)0.5471:0.00010800,SRR11418314:0.00043200)0.5567:0.0
0043200,(((SRR5486075:0.00000100,SRR5486073:0.00000100)0.3767:0.00010800,(SRR548607
2:0.00000100,SRR5486071:0.00000100)0.3800:0.00010800)0.5300:0.00054100,SRR15296964:0
.00075700)0.5033:0.00010800)0.4733:0.00000100,SRR13986562:0.00054000)0.4907:0.000108
00)0.5065:0.00021600)0.5080:0.00000100,((((((((((((ERR6146471:0.00000100,ERR6149920:0.0
0000100)0.0367:0.00000100,ERR6140651:0.00000100)0.0517:0.00000100,ERR6140671:0.0000
0100)0.0778:0.00000100,ERR6149927:0.00000100)0.0958:0.00000100,ERR6149407:0.0000010
0)0.1287:0.00000100,ERR6146479:0.00000100)0.1433:0.00000100,ERR6140825:0.00000100)0.
1876:0.00000100,ERR6149928:0.00000100)0.2217:0.00000100,ERR6148926:0.00000100)0.262
2:0.00000100,ERR6143851:0.00000100)0.2957:0.00000100,ERR6149408:0.00000100)0.3279:0.
00000100,ERR6144471:0.00000100)0.3583:0.00010800,((((((((SRR10993950:0.00000100,SRR10
993951:0.00000100)0.4067:0.00021600,SRR10993942:0.00064800)0.3200:0.00000100,SRR109
93949:0.00000100)0.3644:0.00000100,SRR10993948:0.00021600)0.4675:0.00000100,SRR1099
3944:0.00021600)0.5493:0.00043200,SRR10993946:0.00140600)0.5322:0.00010800,SRR10993
947:0.00086400)0.5152:0.00000100,SRR10993943:0.00129700)0.5600:0.00097200)0.2873:0.0
0000100)0.5117:0.00000100,ERR6149960:0.00000100)0.5118:0.00000100,ERR6149867:0.0000
0100)0.5120:0.00000100,ERR6150014:0.00000100)0.5121:0.00000100,ERR6149987:0.0000010
0)0.5123:0.00000100,(ERR6149409:0.00000100,ERR6148923:0.00000100)0.4233:0.00021600)0
.5126:0.00000100)0.5186:0.00000100)0.5280:0.00000100,ERR6140093:0.00000100)0.5282:0.0
0000100,ERR6149870:0.00010800)0.5284:0.00000100,ERR6149921:0.00000100)0.5286:0.0000
0100,((ERR4188062:0.00000100,(((ERR4187956:0.00000100,ERR4187817:0.00000100)0.1800:0
.00000100,ERR4188133:0.00000100)0.3333:0.00000100,ERR4187955:0.00000100)0.4633:0.00
010800)0.5233:0.00097300,(((SRR11418317:0.00086500,SRR11418323:0.00032400)0.4367:0.0
0086500,SRR13986558:0.00097200)0.4133:0.00000100,SRR14508917:0.00118800)0.5000:0.00
021600)0.4513:0.00010800)0.5301:0.00000100,ERR6149467:0.00000100)0.5303:0.00000100,E
RR6150050:0.00000100)0.5305:0.00000100,ERR6149412:0.00000100)0.5306:0.00000100,(((((((
(((((SRR15296950:0.00000100,SRR15296948:0.00000100)0.0733:0.00000100,SRR15296938:0.0
0000100)0.1150:0.00000100,SRR15296963:0.00000100)0.1756:0.00000100,SRR15296917:0.00
000100)0.2192:0.00000100,SRR15296956:0.00000100)0.2800:0.00000100,SRR15296949:0.000
00100)0.3406:0.00000100,SRR15296941:0.00000100)0.4029:0.00000100,SRR15296972:0.0004
3200)0.4204:0.00000100,SRR15296951:0.00054000)0.4511:0.00000100,SRR15296945:0.00000
100)0.5130:0.00000100,SRR15296978:0.00000100)0.5612:0.00151300,(SRR13046682:0.00000
100,SRR13046675:0.00000100)0.3833:0.00216300)0.4785:0.00000100)0.5333:0.00000100,ERR
6149989:0.00000100)0.5334:0.00000100,ERR6149473:0.00000100)0.5336:0.00000100,ERR613
9645:0.00000100)0.5338:0.00000100,ERR6139834:0.00000100)0.5340:0.00000100,ERR614999
0:0.00000100)0.5341:0.00000100,ERR6140814:0.00000100)0.5343:0.00000100,ERR6146011:0.

00000100)0.5345:0.00000100,ERR6149924:0.00000100)0.5347:0.00000100,ERR6149418:0.000
00100)0.5349:0.00000100,ERR6149871:0.00000100)0.5351:0.00000100,ERR6140810:0.000001
00)0.5353:0.00000100,(SRR5485729:0.00000100,SRR1792485:0.00000100)0.4667:0.00108000)
0.5357:0.00000100,ERR6149923:0.00000100)0.5359:0.00000100,ERR6149416:0.00000100)0.5
361:0.00000100,ERR6149877:0.00000100)0.5363:0.00000100,ERR6143841:0.00000100)0.5365
:0.00000100,ERR6145974:0.00000100)0.5366:0.00000100,ERR6149889:0.00000100)0.5368:0.0
0000100,((((SRR15296930:0.00000100,SRR15296935:0.00000100)0.2467:0.00000100,SRR1529
6931:0.00000100)0.5183:0.00043200,((((SRR15296928:0.00000100,(SRR15296953:0.00000100,
SRR15296957:0.00000100)0.3533:0.00010800)0.2917:0.00000100,SRR15296976:0.00000100)0
.3633:0.00000100,SRR15296979:0.00000100)0.4792:0.00010800,SRR15296927:0.00043200)0.4
480:0.00000100)0.5092:0.00010800,((SRR15296926:0.00000100,SRR15296952:0.00000100)0.2
600:0.00000100,SRR15296975:0.00000100)0.5233:0.00021600)0.5388:0.00032400)0.5391:0.0
0000100,ERR6140813:0.00000100)0.5392:0.00000100,ERR6140466:0.00000100)0.5394:0.0000
0100,ERR6140672:0.00000100)0.5396:0.00000100,ERR6140815:0.00000100)0.5398:0.0000010
0,ERR6150051:0.00000100)0.5400:0.00000100,ERR6150052:0.00000100)0.5401:0.00000100,E
RR6145973:0.00000100)0.5403:0.00000100,ERR6149480:0.00000100)0.5406:0.00000100,ERR6
149885:0.00000100)0.5407:0.00000100,ERR6149478:0.00000100)0.5409:0.00000100,ERR6149
876:0.00000100)0.5411:0.00000100,ERR6149866:0.00000100)0.5413:0.00000100,ERR6140760
:0.00000100)0.5415:0.00000100,ERR6149930:0.00000100)0.5417:0.00000100,ERR6149421:0.0
0010800)0.5416:0.00000100,ERR6149878:0.00000100)0.5415:0.00000100,ERR6149883:0.0000
0100)0.5414:0.00000100,ERR6142310:0.00000100)0.5413:0.00000100,ERR6140661:0.0000010
0)0.5412:0.00000100,ERR6149410:0.00000100)0.5411:0.00000100,ERR6149424:0.00000100)0.
5410:0.00000100,ERR6143842:0.00000100)0.5409:0.00000100,(SRR17156541:0.00000100,SRR
17156537:0.00010800)0.4533:0.00151400)0.5407:0.00000100,ERR6149925:0.00000100)0.540
7:0.00000100,ERR6149468:0.00000100)0.5406:0.00000100,ERR6140776:0.00000100)0.5405:0.
00000100,ERR6149411:0.00000100)0.5404:0.00000100,ERR6140807:0.00000100)0.5403:0.000
00100,ERR6140821:0.00000100)0.5402:0.00000100,ERR6149417:0.00000100)0.5402:0.000001
00,ERR6149875:0.00000100)0.5401:0.00000100,ERR6149886:0.00000100)0.5400:0.00000100,
ERR6150048:0.00000100)0.5399:0.00000100,ERR6149918:0.00000100)0.5398:0.00000100,ERR
6149477:0.00000100)0.5398:0.00000100,ERR6139642:0.00000100)0.5397:0.00000100,ERR614
0809:0.00000100)0.5396:0.00000100,ERR6149926:0.00000100)0.5395:0.00000100,ERR614947
2:0.00000100)0.5394:0.00000100,ERR6149919:0.00000100)0.5394:0.00000100,ERR6140772:0.
00000100)0.5392:0.00000100,ERR6149880:0.00000100)0.5392:0.00000100,ERR6149874:0.000
00100)0.5391:0.00000100,ERR6140817:0.00010800)0.5390:0.00000100,ERR6149868:0.000001
00)0.5390:0.00000100,ERR6149888:0.00000100)0.5389:0.00000100,ERR387001:0.00000100)0.
5388:0.00000100,ERR6148925:0.00000100)0.5387:0.00000100,SRR13986557:0.00000100)0.53
87:0.00000100,ERR6153606:0.00000100)0.5386:0.00000100,ERR6149873:0.00000100)0.5386:
0.00000100,ERR6150015:0.00000100)0.5385:0.00000100,ERR6149988:0.00000100)0.5384:0.0
0000100,ERR6140762:0.00000100)0.5383:0.00000100,SRR16278275:0.00021600)0.5382:0.000
00100,ERR6150047:0.00000100)0.5381:0.00000100,ERR6149887:0.00000100)0.5381:0.000001
00,ERR6149869:0.00000100)0.5380:0.00000100,ERR6149872:0.00000100)0.5379:0.00000100,S
RR15296954:0.00086400)0.5378:0.00000100,ERR6140763:0.00000100)0.5377:0.00000100,ERR
6139636:0.00000100)0.5377:0.00000100,ERR6140242:0.00000100)0.5376:0.00000100,ERR614
9932:0.00000100)0.5375:0.00000100,ERR6149931:0.00000100)0.5374:0.00000100,ERR614948

2:0.00000100)0.5374:0.00000100,ERR6140812:0.00000100)0.5373:0.00000100,ERR6149476:0.00000100)0.5372:0.00000100,ERR6149481:0.00000100)0.5371:0.00000100,ERR6149884:0.00000100)0.5370:0.00000100,ERR6149426:0.00000100)0.5370:0.00000100,ERR6149474:0.00000100)0.5369:0.00000100,ERR6149483:0.00000100)0.5369:0.00000100,ERR6140774:0.00000100)0.5368:0.00000100,ERR6149423:0.00000100)0.5367:0.00000100,ERR6140654:0.00000100)0.5366:0.00000100,ERR6149881:0.00000100)0.5366:0.00000100,ERR6144470:0.00000100)0.5365:0.00000100,ERR6150053:0.00000100)0.5365:0.00000100,ERR6149420:0.00000100)0.5364:0.00000100,ERR6149414:0.00000100)0.5364:0.00000100,ERR6146482:0.00000100)0.5363:0.00000100,ERR6139981:0.00000100)0.5362:0.00000100,ERR6140823:0.00000100)0.5361:0.00000100,ERR6149929:0.00000100)0.5361:0.00000100,ERR6149419:0.00000100)0.5360:0.00000100,ERR6149475:0.00000100)0.5360:0.00000100,ERR6149865:0.00000100)0.5359:0.00000100,ERR6149922:0.00000100);
