# Supplementary material for: Global-scale GWAS associates a subset of SNPs with animal-adapted variants in M. tuberculosis complex
Source: BMC Med Genomics. 2023 Oct 24;16:260. doi: 10.1186/s12920-023-01695-5 (PMC10598944; doi:10.1186/s12920-023-01695-5)

Phenotype *Sus* GWAS Output

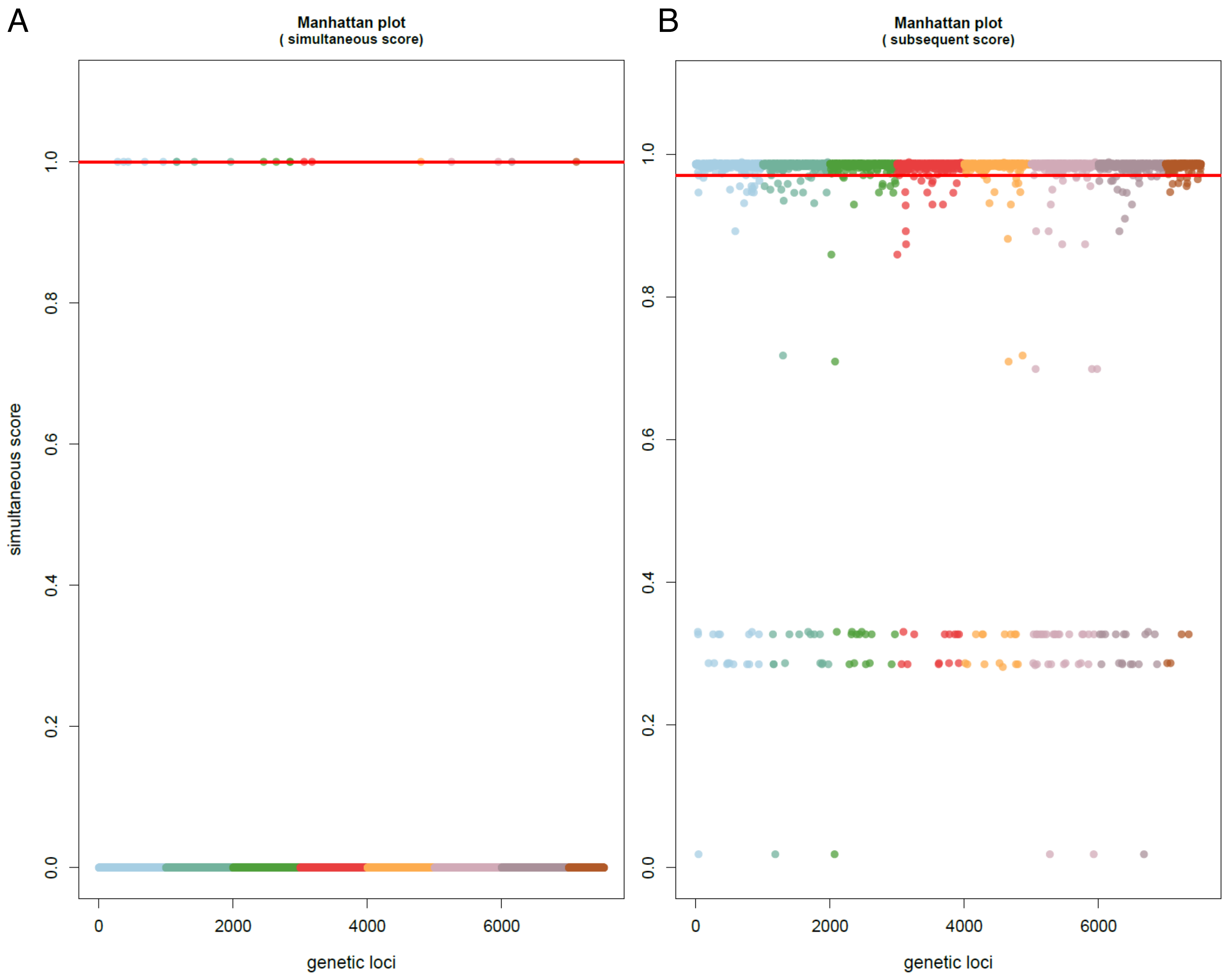

Supplement: Supplementary file 7 — Additional file 7. [file 12920_2023_1695_MOESM7_ESM.pdf]
