# Supplementary figures and images for: Global-scale GWAS associates a subset of SNPs with animal-adapted variants in M. tuberculosis complex
Source: BMC Med Genomics. 2023 Oct 24;16:260. doi: 10.1186/s12920-023-01695-5 (PMC10598944; doi:10.1186/s12920-023-01695-5)

# Phenotype UK Origin GWAS Output

A

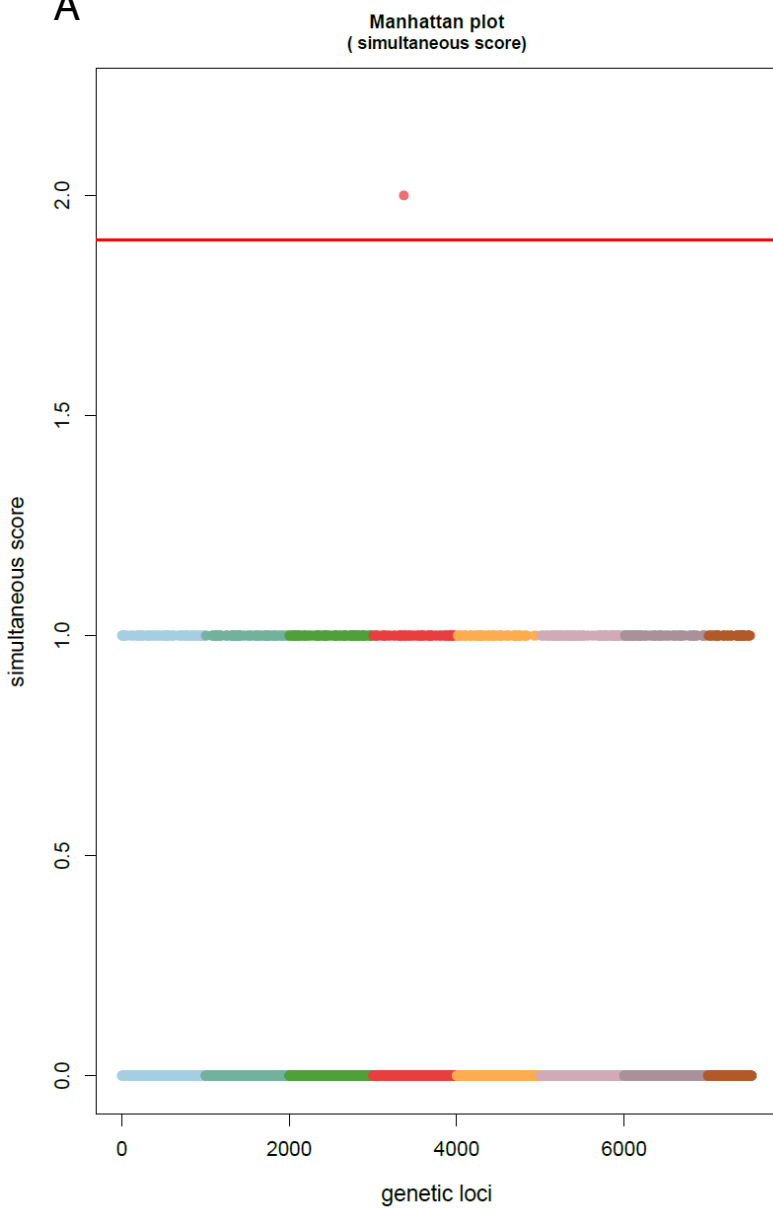

B

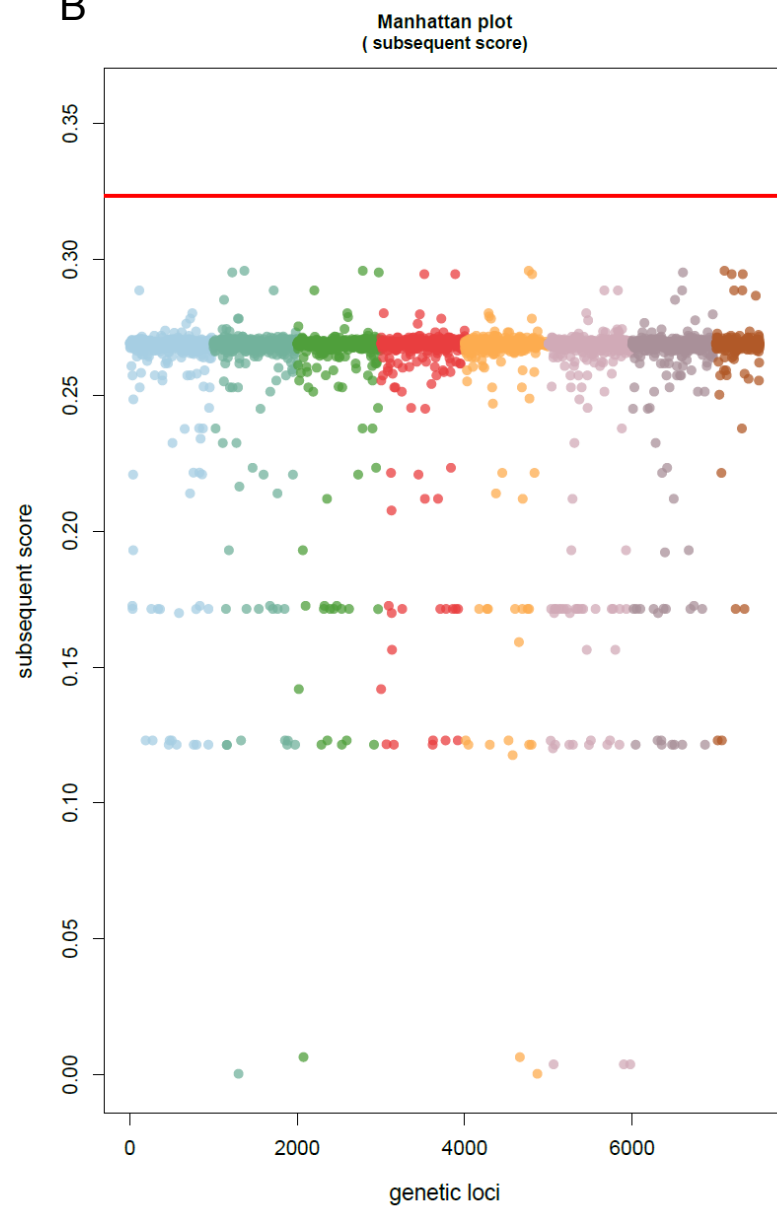

Supplement: Supplementary file 8 — Additional file 8. [file 12920_2023_1695_MOESM8_ESM.pdf]

# Phenotype MCP GWAS Output

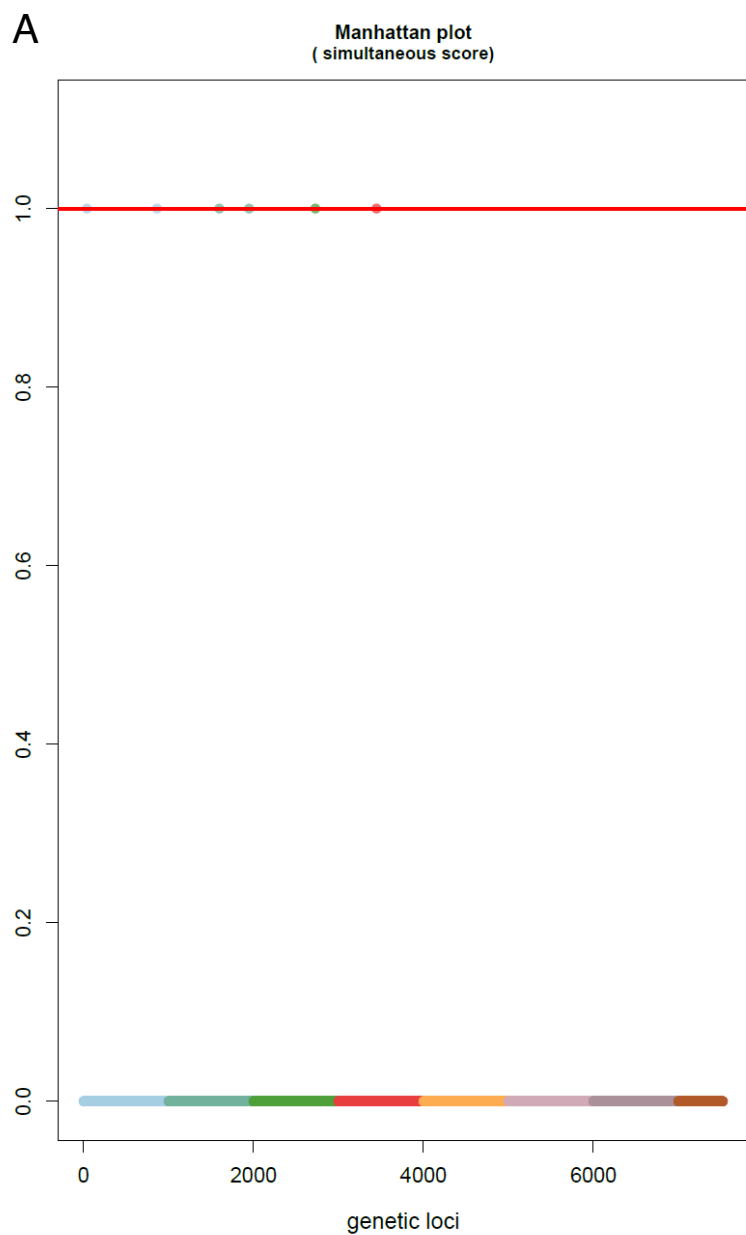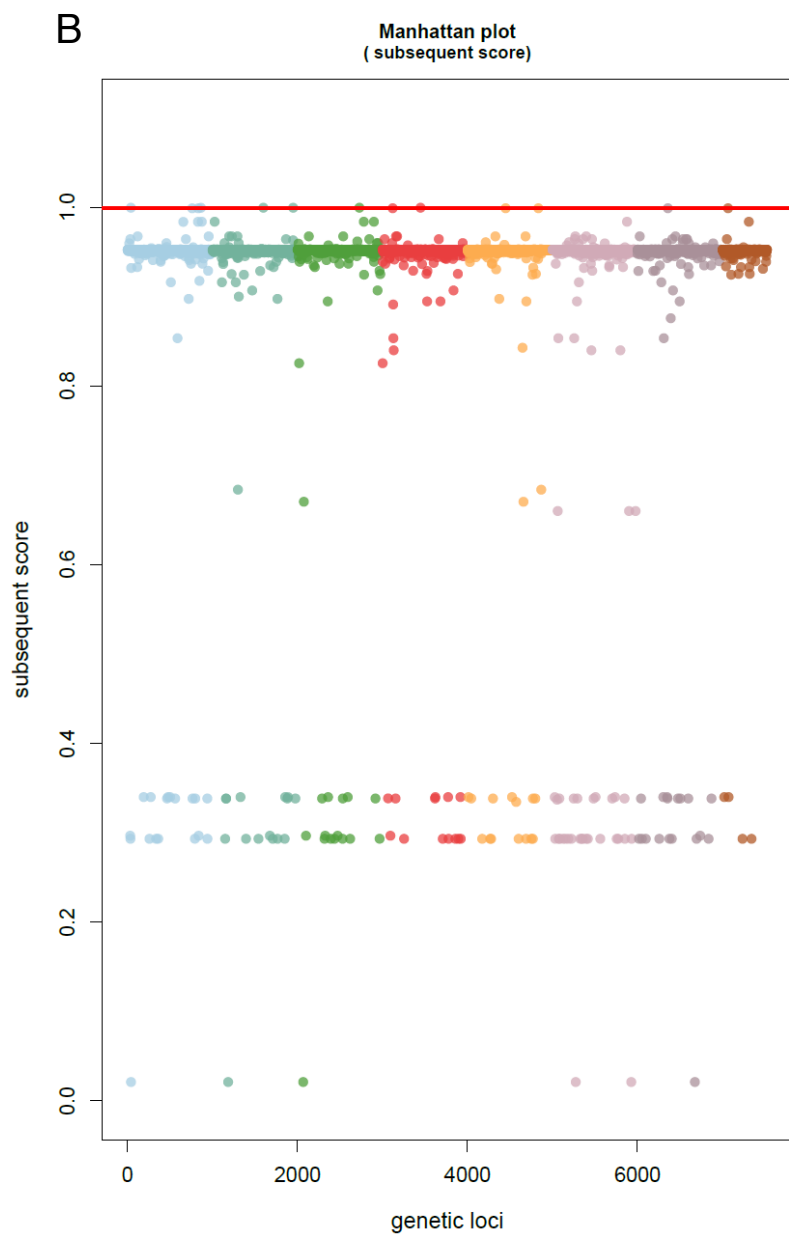

Supplement: Supplementary file 9 — Additional file 9. [file 12920_2023_1695_MOESM9_ESM.pdf]
